# Plant breeding can be made more efficient by having fewer, better crosses

**DOI:** 10.1186/1471-2229-13-22

**Published:** 2013-02-07

**Authors:** John R Witcombe, Sanjaya Gyawali, Madhu Subedi, Daljit S Virk, Krishna D Joshi

**Affiliations:** 1 Centre for Advanced Research in International Agricultural Development (CARIAD), Bangor University, Bangor, Gwynedd, United Kingdom; 2 Local Initiatives for Biodiversity, Research and Development (LI-BIRD), P.O. Box 324, Pokhara, Nepal; 3 LI-BIRD, P.O. Box 324, Pokhara, Nepal; 4 CARIAD, Bangor University, c/o CIMMYT South Asia Regional Office, P.O. Box 5186, Kathmandu, Nepal; 5 Present address: Agriculture and Agri-Food Canada, 107 Science Place, SK S7N0X2, Saskatoon, Canada; 6 Present address: The James Hutton Institute, Craigiebuckler, Aberdeen, UK

## Abstract

**Background:**

Crop yields have to increase to provide food security for the world’s growing population. To achieve these yield increases there will have to be a significant contribution from genetic gains made by conventional plant breeding. However, the breeding process is not efficient because crosses made between parental combinations that fail to produce useful varieties consume over 99% of the resources.

**Results:**

We tested in a rice-breeding programme if its efficiency could be improved by using many fewer, but more judiciously chosen crosses than usual. In a 15-year programme in Nepal, with varietal testing also in India and Bangladesh, we made only six crosses that were stringently chosen on complementary parental performance. We evaluated their success by the adoption and official release of the varieties they produced. We then modelled optimum cross number using assumptions based on our experimental results.

Four of the six crosses succeeded. This was a fifty-fold improvement over breeding programmes that employ many crosses where only about one, or fewer, crosses in 200 succeed. Based on these results, we modelled the optimum number of crosses by assuming there would be a decline in the reliability of the breeder’s prediction of the value of each cross as more crosses were made (because there is progressively less information on the traits of the parents). Fewer-cross programmes were more likely to succeed and did so using fewer resources. Making more crosses reduced the overall probability of success of the breeding programme.

**Conclusions:**

The efficiency of national and international breeding programmes would be increased by making fewer crosses among more carefully chosen parents. This would increase the number of higher yielding varieties that are delivered to farmers and hence help to improve food security.

## Background

Every year, cereal breeders in international agricultural research centres that helped produce the Green Revolution make hundreds, or thousands, of crosses and derive only small populations from each one. This breeding strategy is perpetuated because high-volume crossing, despite the inevitable inefficiency of having many failed crosses, produced the iconic Green Revolution varieties that were successful in dramatically increasing yields from the 1960s to the present day, and because it continues to produce genetic gains in crop yield. Hence there has been little momentum for change even though a high-volume cross approach is very difficult to employ in more modestly funded national programmes. However, changes ought to be considered because making so many crosses that most must fail is not justified either by theory or comparative experiment.

Experimental data for breeding programmes with contrasting cross numbers have not been reported in the literature, which is unsurprising given the size of the experiments required. For example, to test varying combinations of *m* (number of crosses) and *n* (population size derived from each cross), an ideal experiment is two breeding programmes having the same total number of plants (*K*), the same selection methods, but contrasting values for *m* and *n.* This demands huge resources but will still only test one possible strategy for selecting the crosses in the low *m* set. Other experimental approaches such as combining ability tests (e.g., diallel crosses) do not resolve the problem; although they can provide estimates of the relative values of different crosses they cannot predict the probabilities of favourable genotypes occurring as population size varies.

We tested whether a few cross strategy could succeed in rice and compared it with contemporaneous rice-breeding programmes that used the conventional many-cross approach. Within realistic limits of resources, this was the best possible comparison to test the hypothesis inherent in the many-cross strategy that a programme based on few crosses, however chosen, will inevitably fail. We report on only the first six of our stringently chosen crosses because these are the only ones for which there has been sufficient time for the outcomes to be known.

The justification for using fewer crosses was that breeders could predict the better crosses using existing information on the performance *per se* of parents. Combining ability tests are a resource-demanding alternative. Such tests determine means and variances, but they involve the growing and measuring more than three generations of plants as F_2_-derived lines must be tested [1] and spending the time and resources appears unwarranted. However, genotypic performance *per se* provides an indication of parental value [2] and an abundance of information is already available for genotypes that have already been adopted by farmers or have been officially released.

The theory on how many crosses to make has helped little in deciding the optimum number. Models are based on either minimising the risk of excluding superior genotypes [3,4] or maximising the response to selection. We consider only the first of these two approaches in this paper. These models determine the optimum number of crosses (*m)* and population size (*n)* per cross, given a limit of *K* plants. However, contrasting assumptions on how well breeders can predict the value of crosses result in very different optima. Only one cross is needed if a successful cross can be predicted with certainty. However, many are required if there is little power of prediction and this was assumed by Yonezawa and Yamagata because they had a constant value for the probability of success of each cross no matter how many crosses were made [3].

We dropped this assumption because it is based on an extraordinary premise - that the breeder has no ability to choose crosses that are more likely to succeed. It is more realistic for the probability of success of each cross combination to differ according to the information available to the breeder for choosing possible cross combinations. The reliability of the prediction will decline as the number of crosses increase based on a law of diminishing returns; as the number of crosses increases the quality and quantity of available information on the parents diminishes. Weber [4] did consider crosses having a greater chance of good genotypes than in others, and concluded it may be better to enlarge the size of the progenies from more favourable crosses and to reduce the whole number of crosses. However, he concluded that it is usually not optimal to exclude less favourable crosses but did not generalise on what was the optimum number.

## Results

### Success rate of the six crosses – evidence from performance of the rice varieties on farm and on station

Four of the total of six crosses we made were successful as they produced in replicated trials significantly higher-yielding varieties than the best available alternatives in three rice ecosystems both on farm [5-8], Additional files 1, 2] (Table 1) as well as on station (see below). These higher yields were achieved without farmers having to apply more inputs and were accompanied by improvements in other traits that had been targeted in the breeding programme to meet the needs of the client farmers.

**Table 1:** Yield advantage over checks of some of the new varieties from the four successful cross combinations under local farming conditions

**Country and cross number (see Table**4)	**Comparison made**: **new variety *versus *check**	**Yield advantage on**-**farm**^**a**^	**Yield advantage on**-**farm over local varieties**^**b**^
**Nepal** (**cross 1**)	Barkhe 3004 *versus* Mansuli	2003 to 2005: 19% more: 0.65 ± 0.46 t ha^-1^ (n=18) [5]	2005: 44% more: 1.5 ± 0.20 t ha^-1^ (n=23) [5], Additional file 1]
**India** (**cross 1**)	Ashoka 200F, Ashoka 228 *versus* Birsa Gora 102	2000 to 2001: 54% more: 0.5 ± 0.1 t ha^-1^ (n=40) [6]	2001: 35% more: 0.41 ± 0.15 t ha^-1^ (n=198) [6]
**Bangladesh** (**cross 2**)	Judi 582 *versus* Swarna, BRRIdhan 32, BRRIdhan 39	2002 to 2004: 44% more: 1.0 ± 0.2 t ha^-1^ (n=22) [7]	2003 to 2005: 21% more: 0.49 ± 0.14 t ha^-1^ (n=61) [7]
**Nepal** (**cross 3**)	Sunaulo Sugandha (aromatic) *versus* Mansuli (non aromatic)	2002 to 2004: 6% more: 0.16 t ha^-1^ (n.s.) (n=36). 1.07 t ha^-1^ more than aromatic varieties (n = 9) [8]	2004 to 2006: 15% more: 0.54 t ha^-1^ (n = 101) [8], Additional file 2]
**India** (**cross 3**)	Sugandha 1 *versus* IR64	2003 to 2007: 14% more: 0.42 ± 0.14 t ha^-1^ (n=69)	2007: 26% more: 1.1 ± 0.14 t ha^-1^ (n=4)
**Nepal** (**cross 5**)	Madhyam Dhan 0742 *versus* Mansuli	2008 to 2011: 26% more: 1.0 ± 0.2 t ha^-1^ (n=101)	Data not yet available

^a^In multiple-entry trials where each on-farm trial is a replicate of a randomized complete block design (plot size > 10 m^2^) and n = number of trials. All differences are significant from ANOVA except Sunaulo Sugandha versus non-aromatic controls.

^b^In single-intervention trials i.e., paired plots with one new variety versus farmer’s best check (plot size > 50 m^2^) where n=number of trials. All yield differences are significant from ANOVA.

± = standard error.

In India, the on-station yield advantage in Jharkhand from 1999 to 2001 of Ashoka 200F and Ashoka 228 averaged 28% more grain, an additional 0.56 ± 0.1 t ha^-1^. However, in much higher yielding All-India Coordinated Rice Research Project (AICRRP) Trials these early-duration varieties did not yield as much as later maturing entries. This was a reflection on the inappropriateness of the trials that had a mean grain yield more than treble that achieved by farmers in the difficult target environments, rather than any shortcomings of the varieties [6]. The advantages of their early duration were not considered because the AICRRP trials had no provision for considering yield per day or trading off yield against duration. In Nepal, in on-station trials in 2003 to 2005, Barkhe 3004 yielded 12% more grain than Mansuli, an extra 0.37 ± 0.1 t ha^-1^ in seven four-replicate trials of the National Rice Research Programme (NRRP) of the Nepal Agricultural Research Council (2 in 2003, 2 in 2004 and 3 in 2005). Judi 582 was not tested on-station in Bangladesh. Sunaulo Sugandha was tested against Mansuli in only three on-station trials and these were included in the randomised complete block design data in Table 1. Sugandha1 was tested from 2004 to 2007 in 18 on-station trials and it yielded 0.5 ± 0.09 t ha^-1^, 12% more than IR64.

### Success rate of the six crosses – evidence from official recognition and adoption by farmers

Several varieties have received official recognition in Nepal and India (Table 2) reflecting their performance on station and on farm. The first cross produced Ashoka 200F and Ashoka 228 and these were released in 2003 as Birsa Vikas Dhan 109 and 110 for the rainfed uplands of Jharkhand state, India. Later, Ashoka 200F was officially recommended for cultivation in Gujarat in 2006, and Madhya Pradesh and Rajasthan in 2005. In 2006, Barkhe 3004 was officially released in Nepal followed by the release of Barkhe 3010 in India and the registration of Barkhe 1027 in Nepal. The third cross produced one variety that has been released in Nepal and one in India. Several varieties have performed well in Bangladesh, but the operation of the seeds act makes it impossible to get these released. Various outcome assessment studies have shown that many more varieties than those that have been released have been adopted [10-15], Additional files 3- 7] (Table 2) and that some of the varieties, such as Ashoka 200F and Ashoka 228, are better accepted than any previously released modern variety as they rapidly replace them [9].

**Table 2:** Varieties from the six crosses that have been officially released or registered or identified as having significant adoption

**Cross**	**Country**	**Released or registered varieties**	**Year**	**Adopted varieties**
KIII/IR64†	India	Ashoka 200F	2003	The 2 Ashoka varieties
		Ashoka 228	2003	[10-13]^a^
		Barkhe 3010	2009	
	Nepal	Barkhe 3004	2006	The 3 released varieties
		Barkhe 1027	2011	[14] and
		Barkhe 2014	2011	- Barkhe 3019 [14]^b^
				- Super 3004 [14]
	Bangladesh			- Judi 567 [15]^c^
				- Barkhe 3004 [15]
Radha 32/KIII	Nepal			- Judi 572 [14]
				- Judi 582 [14]
	Bangladesh			- Judi 572 [15]
Pusa Basmati 1	Nepal	Sunaulo Sugandha	2008	-Sunaulo Sugandha [14]
				- Sugandha 1 [14]
				- Barkhe 2024 [14]
				- Barkhe 2001 [14]
	India	Sugandha 1	2009	Sugandha 1
CH45/MT1	Nepal			None
Mansuli/MT4	Nepal	One proposed	2012	Madhyam Dhan 0742
Mansuli/IR64	Nepal			None

^a^References [11-13] in full in Additional files 3, 4, 5.

^b^Reference [14] in full in Additional files 6.

^c^Reference [15] in full in Additional files 7.

### Probabilities that the success rate of the six crosses significantly exceeded those in many cross breeding programmes in Nepal, Bangladesh and IRRI

We calculated how frequently the success rates we had would occur by chance given the same success rates of the breeding programmes in Nepal, Bangladesh and the International Rice Research Institute (IRRI) (Table 3). We determined the number of crosses made by these three programmes in a defined period and the number of releases in an equivalent but later period to allow for about an 8-year lag phase.

**Table 3:** **The probabilities of having two**, **three or four successful crosses after selecting six crosses at random when 1 in 200 are successful**

**Number of successful crosses in the six randomly selected crosses**	**Probability of occurrence of this number of successful crosses**	**Equals one chance in:**
Four	1 x 10^-8^	100,000,000
Three	2 x 10^-6^	404,533
Two	0.0004	2,702

In Nepal, from 1972 to 2003, the national research system made 1951 crosses for the low altitude regions and 1011 crosses for the hills [unpublished data obtained from the Nepal Agricultural Research Council]. In the 32-year period from 1980 to 2011, 37 rice varieties were released in Nepal. Of these, 13 were from crosses made in Nepal [16,17] (3 for low altitudes and 10 for the hills). This was an overall success rate of about 1 cross in 228 although for the terai it was only 1 in 650 and for the hills 1 in 101. At the Bangladesh Rice Research Institute (BRRI) 5840 crosses were made from 1970 to 1997 [18]. From 1978 to 2007, 38 rice varieties were released. At least 6 of them were not from crosses made at BRRI, so about 1 cross in 183 resulted in a released variety but farmers have not adopted all of these. Unpublished information indicates similar success rates in India. IRRI made more than two thousand crosses a year [19,20] from approximately 1965–1995 (because precise dates of the crosses are not documented). By 2005, 328 breeding lines from IRRI had been released as 643 varieties (because many are released in more than on country) in 75 countries [21] giving an estimated success rate of about 1 in 213.

Was our high success rate of 4 in 6 a significant improvement over that in a many-cross breeding programme or due to chance? We determined the likelihood of producing a success rate of 4 in 6 if six crosses were randomly selected from a breeding programme where 1 in 200 crosses succeed. It was extremely improbable that the random selection would include four successful crosses, and unlikely that it would include three (Table 3). Hence, our high success rate was not by chance but because the stringent crosses were more likely to succeed. If we apply the most rigorous definition of success – that a variety also has to be released as well as adopted - then the success rate would fall to 2 in 6 (with a possible increase to 3 in 6 if the pending release proposal of a variety from the fifth cross is approved). Even with a success of 2 in 6 the results remain highly significant, P<0.001 (Table 3).

We can also determine how high the success rate needs to be in a conventional programme for our results to become non-significant. For 3 from 6 crosses to be successful by chance the successful crosses in a conventional programme would need to rise to 4 per 100 (for P to be above 0.001) or 9 per 100 (for P to be above 0.01). Such increases of 8 to 18 fold over the overall reported rates of about 0.5 per 100, and four fold more than the highest rate for the hills in Nepal, and are beyond any errors that might be expected in the documented success rate of the three conventional breeding programmes in Nepal, Bangladesh and IRRI.

By country, the success rate was 2 out of 3 for Bangladesh (66%), 2 out of 2 for India (100%) and 4 out of 6 for Nepal (66%). The standard error associated with a proportion of success of 0.66 is ± 0.19 and we have conservatively used a value of 0.4, lower than the lower range of the SE, to model the optimum cross number.

### Optimum number of crosses when the probability of success of each cross declines as more crosses are made

We have shown that using performance *per se* as an indicator of parental value allowed cross combinations to be selected that had a higher probability of success than more random ones. Hence, to model optimum cross number *P*1 (the probability of success in a cross) should not be a constant, but decline as more crosses are made, because the quantity and quality of relevant information for choosing crosses on performance *per se* also declines.

Because there are no experimental data on how the quantity and quality of knowledge on performance *per se* of potential parents declines with the number of crosses that are made*,* we modelled the rate that fits best with our experience (an S-shaped curve). We also examined other, contrasting, feasible relationships: a linear decline in knowledge of the parents the more crosses are made (and hence the same decline in the probability of success) or an exponential decline. The initial levels of *P*1 were conservatively assumed to be lower than those we found experimentally (see above), and it was assumed *P*1 would decline as more crosses were made to fall the level of 0.005 typically found in high cross breeding programmes.

**Figure 1:**
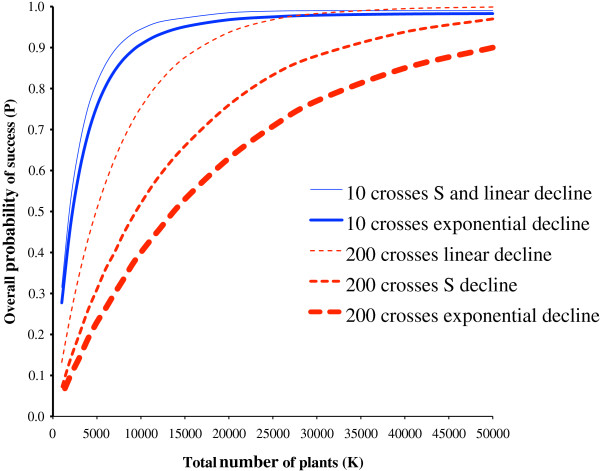
**The overall probability of a programme succeeding when *P*1 declines with cross number.** A comparison of either 10 or 200 crosses with *K* varying from 1,000 to 50,000 and with the probability of plant succeeding within a cross constant at 0.001. The probability of an individual cross succeeding is initially 0.4 and declines to 0.005 in an S-shape (S), or linearly (L), or exponentially (E) (the exact shapes are shown in Figure 3). Using 10 crosses with an S-shape or a linear decline give practically identical results, so only the mean of the two is shown.

No matter what particular rate of decline is assumed, it always results in many crosses (200) being less efficient than a few (10) across a range of *K* (Figure 1). Only when there is a linear decline in *P*1 and values of *K* are high is there a tiny advantage (measured by overall probability of success) in using 200 crosses over 10. However, making fewer crosses remains the most efficient option as the insignificant gain in overall probability is outweighed by the increased cost of making more crosses.

With 10 crosses, *K* can be reduced substantially (from 50 000 to 6 000) with only a small reduction in the probability of overall success (Figure 1).

We modelled three further scenarios: (1) having lower initial values of *P*1, (2) different values for *P*2, and (3) co-varying *P*2 with *P*1.

(1) When the initial values of *P*1 are lower than 0.4 making fewer crosses is still advantageous. For the S-shaped decline, 200 crosses only become superior to 10 when *P*1 falls to 0.075 (when *K* = 50 000), while 10 crosses are always superior to 200 for the exponential decline no matter how small is *P*1. For the linear decline the initial value of *P*1 had to fall to about 0.2 before 200 crosses had any advantage over 10 (and then only for when *K* > 30 000).

(2) The value of *P*2 (assuming for simplicity it is constant) is important – smaller values of *P*2 greatly increase the advantage of using fewer crosses, albeit to differing extents, in the three rates of decline of *P*1. For example, with *P*1 initially equal to 0.4 and *K* = 30,000 we compared the overall probabilities of success when *P*2 was equal to 0.001 or 0.0005. With the lower value of *P*2, the advantage of 10 crosses over 200 is 3.7 times larger in the case of an S-shaped decline, 24 times larger for a linear decline, and 2.9 times larger for an exponential decline.

 Moreover, the Yonezawa and Yamagata model uses high values of *P*2 since they are based on the recovery of favourable heterozygous or homozygous genotypes in the F_2_[3], and hence ignores the required population sizes in subsequent generations to obtain homozygous segregants. It is more realistic to model the probabilities of finding a desirable homozygote in an advanced selfing generation, and this can easily be applied to single seed descent where population size remains constant from the F_2_ to the advanced selfing generations. *P*2 is then very small because, for example, the probability of recovering a transgressive segregant favourable at 10 loci is 0.0001, and favourable at 14 loci it is 0.00006. The larger populations possible with fewer crosses are then most helpful in increasing the possibility of finding favourable genotypes.

(3) It is a reasonable assumption that the frequency of favourable plants in a cross is positively related to the probability that a cross will succeed, i.e., *P*2 should be higher when *P*1 is higher. This increases the advantage of having fewer crosses but not by a large percentage.

### Optimum number of crosses when the probability of success of each cross is a constant

**Figure 2:**
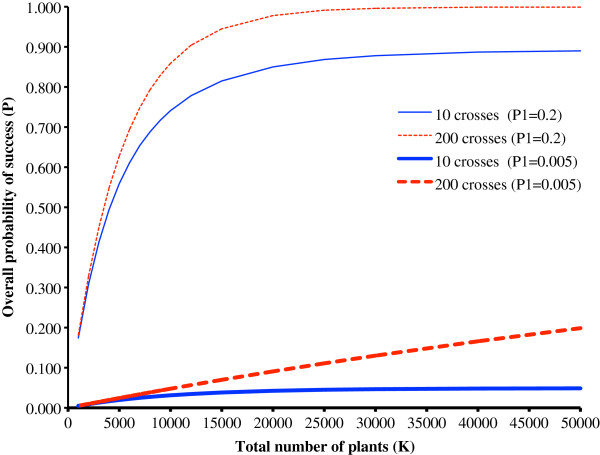
**The overall probability of a programme succeeding when P1 is constant.** A comparison of either 10 or 200 crosses and *K* varying from 1,000 to 50,000 when the probability of an individual cross succeeding (*P*1) is a constant 0.005 or a constant 0.2 and the probability of a plant succeeding (*P*2) is a constant 0.001.

In great contrast to Figure 1, more crosses always increase the probability of success when *P*1 is assumed to be constant (Figure 2).

However, despite this there are counter arguments that favour fewer crosses. The higher the value of *P*1 the smaller the relative advantage of more crosses becomes (Figure 2). For example, when *P*1 = 0.005 then 200 crosses gives a 179% increase in the overall probability of success compared with 10 (with *K* = 30 000). However, this falls to 13% when *P*1 = 0.2, and to <1% when *P*1 = 0.4 and such small advantages will not justify the cost of more crosses.

As discussed above, *P*2 becomes much smaller when the probability of recovering homozygotes in an advanced selfing generation is considered. The smaller *P*2 becomes, the smaller is the advantage of making more crosses. However, a smaller *P*2 has much less effect when *P*1 is constant compared with when it declines as more crosses are made (see above).

Fewer crosses allow an increase in population size but the Yonezawa and Yamagata model does not take the advantage of this into account, because it assumes that the probability of a cross being favourable, *P*1, remains constant no matter how many plants are derived from it. This is because the model produces a ceiling for the number of plants beyond which an increase is of no benefit. For example, having more than 3,000 plants in a cross when *P*2 = 0.001 is of no benefit, as the risk of failing to find a favourable plant in an F_2_ population of this size has already fallen to zero. However, the favourable plant has advantageous alleles at a defined number of loci (when *P*2 = 0.001 this is at 25 loci) [3]. Increasing the population size to give a high probability of recovering a plant with advantageous alleles at, say, 26 loci ought to increase *P*1 over having a smaller population size where 25 can be expected. Because the model does not account for this improvement in *P*1 with increased population size, it is biased towards favouring more crosses rather than more plants per cross.

## Discussion

If *P*1 and *P*2 are assumed to be constant it is mathematically impossible using the equation of Yonezawa and Yamagata [3] for fewer crosses to have a higher overall probability success than many. However, if *P*1 is 0.2 or above (considerably below the 0.66 we found experimentally) making more crosses does not give sufficient increases in the overall probability of success to compensate for the additional costs. Increasing cross number is resource demanding since it is both easier and cheaper to grow, for example, 5,000 plants from each of ten crosses than 250 plants from each of 200 crosses. Apart from the cost of having to make more crosses, it involves growing more field plots, having more labels and packets, gathering more data, and having more parental checks. A similar argument has previously been made even for when *P*1 was at more modest levels [3]. For example, when *P*1 is 0.1 or more and *K* is 50,000 or more, the costs associated with having more crosses would be more decisive in deciding the optimal crossing scheme [3].

An alternative to the labour-intensive option of increasing *m*, is to increase *K* – although no more expensive, a few crosses with a large *K* is less risky than many crosses with a smaller *K*[22].

Our model on optimum cross number is the first that shows that making more crosses can reduce plant breeding efficiency and has done so by making more realistic assumptions. Our experiment showed that by using existing information on the performance *per se* of the parents we could predict, with a high probability, the best crosses to make. We did this by using the abundance of information already available on the traits of varieties adopted by farmers or officially released. We hence assumed that *P*1 must decline as more crosses are made because the available information on the performance *per se* of possible parents also declines. This equates to a law of diminishing returns as more crosses are made. The manner in which the diminishing returns occurred was not critical. All the three relationships of the decline between *P*1 and *n* we modelled, S-shape, linear and exponential, resulted in more crosses being less efficient than fewer. What does make a difference is how quickly the probability of success of each cross falls to that of more randomly made crosses. The longer it takes to fall to this level the less the disadvantage of making more crosses and *vice versa*.

From our experience, an S-shaped relationship between *P*1 and *n* is the most realistic assumption. We found it difficult to rank the best cross combinations among the relatively few genotypes for which there was well-proven performance *per se* in the target population of environments (TPEs) over many years. Hence, there was little to choose between the first and the next few crosses. After this, the quality of prediction would decline, as we would have to use parents for which there was less information on their performance in the TPEs over fewer years. Finally, there were many more possible parents for which we knew very little, may not even have been tested in the TPEs, may only have been tested in a single season, and hence were only as likely to succeed as those in a conventional, many-cross programme.

Experimentally, there is already much evidence on the success of using many crosses but little evidence has been provided on either the success or failure of the alternative approach of making only a few. We have positively answered the key question: can a few stringently selected crosses succeed by producing improved crop varieties that farmers adopt and, thereby, increase breeding efficiency? A few crosses succeeded because we were able to significantly increase the probability of success (*P*1) of each cross over the low level found in conventional programmes. We then made the most practicable experimental comparison possible: determining if the *P*1 we found in our experiment was significantly better than that of many-cross breeding programmes from the same region and crop. It is the most comprehensive test, as far as we know, on the feasibility of the few cross approach for inbreeding crops.

For fewer crosses to succeed an increased likelihood of success of each cross has to be achieved by making greater efforts than normal to choose complementary parents. Our crossing strategy was to cross parents that have been widely grown in the TPEs over many seasons (e.g., Kalinga III, IR64, Mansuli) either with new material from our breeding programme or with varieties identified using participatory varietal selection [10]. More recently, we have been able cross the best lines that have emerged from our breeding programme (e.g., Barkhe 1027, Sunaulo Sugandha, Judi 582) with the most popular, well-established local variety (no matter what its original source). Breeding programmes with a global reach can also more carefully choose parents, and hence reduce the number of crosses, by making crosses with germplasm targeted at individual countries and domains [10].

A fewer-cross strategy greatly simplified the breeding scheme and saved resources. Much larger F_2_ populations were possible with fewer crosses and they could be easily handled by using early-generation bulk populations [10]. Although larger population sizes were used for each cross - to ensure that a well-chosen cross has sufficient plants in its progeny to succeed - the overall size of the breeding programme can be reduced. We had about 1 ha of breeding material in each season. This was a smaller area than the smallest of the three rice breeding programmes to which we made comparisons, i.e., that of NRRP, but our programme produced released varieties at more than twice the rate. Hence, a few-cross programme could achieve on about half the land area (and with a much simpler layout as there are fewer entries) the same release rate as a many-cross programme.

There is some other evidence that the few-cross approach is effective. Another rice breeding programme in Nepal by collaborators of the International Plant Genetic Resources Institute (IPGRI now Bioversity International) has also relied on only a few crosses. One parent was always a local landrace because landrace utilization was an objective of the programme. Even with this constraint on the choice of parents of only eight crosses, four have resulted in varieties that are in the release-, or pre-release, stage (Gyawali, unpublished). At the West Africa Rice Development Association (WARDA) many crosses are made each year. However, because the crosses were so difficult to make in a ‘wide-cross’ breeding programme between *Oryza sativa* and *O. glaberrima* considerable effort was placed on choosing the parents of the crosses [23]. Only eight parents of *glaberrima* and five of *sativa* were chosen on the basis of their best combination of traits and only seven of the crosses set seed. All of the seven ‘New Rice for Africa’ (NERICA) varieties that were released in 2000 [24] were from just one of these crosses, a success rate of 14%. As was the case for our crosses, this is a considerable improvement over normal success rates and our experience suggests that this was due to the great attention paid to choosing parents necessitated by the high cost of making these wide crosses. In maize, the parallel of a few-cross approach is to make only a single composite population and we tested this in western and eastern India. Two populations were made, one for each region, and both have produced a released variety [25,26].

A breeding programme can be safely based on making very few crosses each year. The six crosses we analysed were made over a period of four years (1.5 per year on average). With one cross a year, a success rate of 50% would be more than sufficient for a very successful programme. With two a year, a rate as low as 20% will give nearly a 90% probability of breeding a successful new variety every 5 years, which compares well with many national breeding programmes. These probabilities would be higher if, as we found, some of the crosses produced more than one released variety.

What if all breeders used only a few crosses? This would restrict the amount of germplasm used in crosses but not restrict the amount used in successful crosses. In conventional programmes, although many crosses are made most neither produce released varieties nor progeny that would be used in crosses to eventually produce a released variety. However, the exceptions are valuable, for example IR64 has an extremely complex parentage with 20 original farmer varieties from 8 countries as parents [19]. Clearly, not all of them would have previously been released varieties or parents of released varieties so many of the crosses were actually used for pre-breeding i.e., the creation of parents. Hence, to deliberately broaden the genetic base of crops the range of parents has to extend beyond successful cultivars, resulting in an increase in the number of crosses (but probably still many fewer than those currently made). Fewer crosses are highly appropriate for breeding programmes that have limited resources and that are targeted at specific environments, an apt description of most national, public-sector breeding programmes.

## Conclusions

Given the strong evidence that the chances of a cross succeeding can be considerably improved by the careful choice of cross combinations, we conclude that reducing the number of crosses increases plant breeding efficiency. This is contrary to the current practice in most breeding programmes. Many public-sector national programmes cannot replicate the high volume crossing strategy of better-funded international breeding programmes. They can easily adopt the strategy of making fewer, more carefully chosen crosses to increase the efficiency of their breeding programmes and deliver more new varieties to their client farmers and thereby improve their food security. Large international breeding programmes are inefficient in that the vast majority of crosses fail and would be more effective if fewer, better targeted crosses were made.

## Methods

### Hypothesis and strategy

We tested the effectiveness of a low *m* strategy, with stringent selection of the crosses based on complementary parental performance in the target environments in a fifteen-year international breeding programme in Nepal (with varietal testing in India, and Bangladesh). There has been sufficient time to produce quantitative evidence on success or failure for the first four crosses and strong indications on the success of the last two. We compared the success rate of these six crosses with that in conventional breeding where success was defined as when a cross had produced at least one officially released variety – a conservative measure for the comparison as it overestimates success in the conventional programmes because a significant proportion of released varieties are never adopted [27,28]. For our breeding programme, success was defined as when a cross had produced a variety that farmers adopted (most adopted varieties were also released). To estimate adoption a series of outcome assessments were conducted (see below ‘measuring the success of the crosses’).

By using fewer crosses, the number of plants from each cross (*n*), could be increased substantially and still have fewer plants in total than in a conventional high-cross programmes. To use so few crosses, the parents were stringently chosen (for methods see [10,22]). We also attempted to reduce the risk of failure from poor grain quality and the untoward effects of genotype x environment interaction by testing grain quality with end-users before yield trials and selecting for agronomic traits in the target environments, i.e. the farmers’ fields in Nepal, India and Bangladesh.

Our breeding programme could be expected to have a lower than average success rate because the environments we targeted were more difficult than average. They were environments where either the green revolution had never taken off (landraces constitute the majority of current cultivation) or, after initial success, there had been little further progress (the majority of modern varieties that farmers were growing were decades old).

### Comparison to other breeding programmes

We compared the success rate of crosses in our programmes to those in public sector rice breeding programmes in Bangladesh and Nepal using published data for Bangladesh [18] and Nepal [16,17]. We also made a comparison to the breeding programme of IRRI using published data on crosses [19,20] and the number of breeding lines released up to 2005 [21].

### Choosing the crosses

We have made six crosses targeted at the main season in Nepal for which there has been sufficient time to evaluate the progeny (Table 4). Stringent selection criteria were applied to choose these crosses. We defined the breeding objective by market research with farmers and then determined the most likely cross combination that could meet this objective using information already available on the possible parents.

**Table 4:** The first six crosses in the breeding programme in Nepal

**Cross number and parentage**	**Year**	**Tested in**
1. Kalinga III/IR64†	1997	India, Nepal, Bangladesh
2. Radha 32/Kalinga III	1998	Nepal, Bangladesh
3. Pusa Basmati 1 population	1998	Nepal, Bangladesh, India
4. CH45/Medium Tall Bulk 1	1999	Nepal
5. Mansuli/Medium Tall Bulk 4	1999	Nepal
6. Mansuli/IR64	2000	Nepal

†Cross for breeding programme in India (commenced with F_3_ in Nepal). All other crosses made in Nepal.

The first cross was between Kalinga III and IR64. IR64 was from a high-cross breeding programme from IRRI and was at one time the most widely grown rice genotype in the world and has excellent roots for a lowland variety [29]. This cross was targeted at two countries, India and Nepal. For Nepal, the target domains were intermediate and semi-deep rainfed environments, and for India the rainfed uplands. Kalinga III was bred by the Central Rice Research Institute, Cuttack, India, and was one of the most widely adopted upland rice varieties in India, but has poorly developed roots for an upland variety [30]. It could contribute early maturity, the tall plant height that most farmers in the target regions require for high fodder yields, and good grain quality. For breeding for upland environments in India, IR64 could contribute improved rooting, and for both countries it contributed better lodging resistance, high yield and resistance to multiple pests and diseases.

The second cross was between IR44595, a variety bred at IRRI, and named as Radha 32 in Nepal, and Kalinga III. The former parent was chosen for its exceptional high yield in Nepal in the early (*Chaite*) season [31]. However, farmers rejected it because of its poor market price due to poor cooking quality so it was crossed with Kalinga III, that farmers had liked in the *Chaite* season in Nepal [32] to contribute improved grain quality and earliness.

The third cross was derived from a randomly outbred population of Pusa Basmati 1, a variety bred at the Indian Agricultural Research Institute (IARI), that naturally outcrosses because of poor pollen production (partial male-sterility). Farmers liked Pusa Basmati 1 in on-farm trials in Chitwan, Nepal, for its high yield and grain quality but they reported two disadvantages – awned florets that made milling more difficult and dwarf plant height that reduced straw yield that is economically important in Nepal as fodder. The removal of these two traits would make the variety highly acceptable to farmers. Although this was strictly not a single cross it was treated as such because the progeny from the population were advanced as if from a single cross.

The fourth cross was targeted at the *Chaite* season only and the most popular variety for this season, CH45, was crossed with a medium tall, early bulk designated as MT1 from the cross Kalinga III/IR64. Farmers regarded CH45 as being later to mature than optimal, and MT1 could impart earliness as well as improved grain quality.

The fifth and sixth crosses were with Mansuli, a highly popular variety with farmers in Nepal (and particularly in Chitwan) because of its adaptability, premium grain market price and high straw yield from tall plant height. However, it was susceptible to lodging and increasingly susceptible to disease. The objective was to retain the quality characteristics, tall plant height, and phenotype of Mansuli (the same grain type, golden glume colour and maturity) while improving lodging resistance, disease resistance and yield. It was crossed with an F_4_ bulk from the cross Kalinga III/IR64 that had been selected in Chitwan for uniformity of medium-tall plant height, later maturity, lodging resistance and fine grains (designated as MT4). For the sixth cross, Mansuli was crossed to IR64 as a back-up of the fifth cross as it had multiple diseases resistance but – because dwarf plant height gave it its lodging resistance – there was little indication of its value as a donor of lodging resistance in a tall progeny.

### Measuring the success of crosses

We surveyed the preferences of farmers by interviewing those that had tried the new varieties using structured questionnaires and focus group discussions. For the products from Kalinga III/IR64 in eastern India (Ashoka 200F and Ashoka 228) 159 farmers were surveyed in 2002 and 150 in 2004 [9] while 11 whole villages were surveyed in 2005 that first had access to seed in 2002 [11]. In western India a survey (total of 165 farmers) in Gujarat was conducted in 2005 by the Gramin Vikas Trust (GVT) and Anand Agricultural University (AAU); in Madhya Pradesh by GVT and Jawaharlal Nehru Krishi Vishwa Vidhyalaya (JNKVV); and Rajasthan by GVT and Maharana Pratap University of Agriculture & Technology, Udaipur (MPUAT) [12]. A survey was also made in 2008, funded by the Monitoring Impact and Learning (MIL) component of the Department for International Development (DFID)-funded Research into Use Programme (RiUP). Based on a larger sample size, it confirmed the results of the previous studies [13]. A similar MIL study was done for the uptake of rice varieties in Nepal [14] and the success of rice varieties from the Nepal breeding programme was also documented in Bangladesh [15].

### Statistical analysis – measuring the probability of the success rates found

The probabilities were calculated of how frequently our observed success rates would occur by chance given the same success rates of the breeding programmes in Nepal and Bangladesh. This was done by expanding the binomial: (x + y)^m^ where x was the probability of success and y the probability of failure (=1-x) and *m* = number of crosses. For example, when *m* = 3 then the probability of 3 successes = x^3^, 2 successes = 3x^2^y, 1 success = 3y^2^x, and 0 success = y^3^.

### Modelling optimum cross number – varying the probability of success of crosses

We used the model of Yonezawa and Yamagata [3] to examine in more detail the relationship between cross number, population size and probability of success. In the 1978 paper, the model assumes no prior information on the genetic potential of the parents (i.e., the breeder is not able to have any influence on the probability of a cross succeeding by choosing the parents that are crossed) and computes the probability of minimizing the risk of not selecting any favourable genotype in the total breeding population (*K*) of *m* crosses each with *n* plants. The risk (R) is presented as R = [(1-*P*1)+*P*1(1-*P*2)^n^]^m^ where *P*1 is a constant and = the probability of an individual cross succeeding, i.e., having a favourable plant, while *P*2 is a constant and = the probability of any plant being favourable within a cross. This probability model is also based on the binomial theorem.

**Figure 3:**
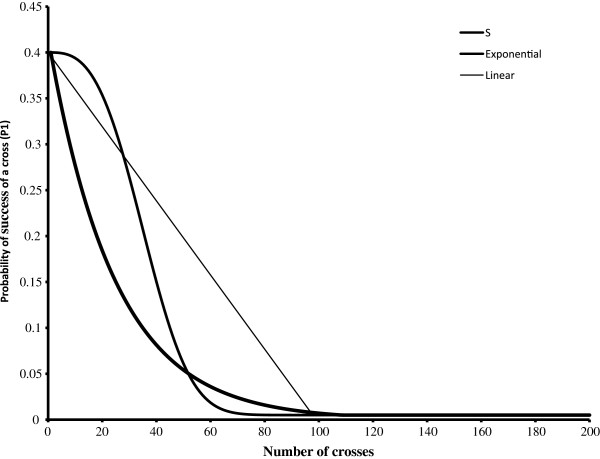
**The assumed rates of decline in the probability of a cross succeeding** (**P1**) **with increasing number of crosses.**

It is an unrealistic assumption that breeder’s knowledge of parents cannot be used to choose crosses that are more likely to succeed. We assumed cross combinations could be chosen that used existing data on performance *per se* of the parents and that such combinations were more likely to succeed than a random choice. The information available on parents, particularly that relating to performance in the target population of environments, will decline as more crosses are made. We modelled three different rates (S-shape, linear and exponential) at which the knowledge of the traits declined, and hence the likelihood of the choice being successful. Figure 3 shows the exact shape of the curves used and in all cases the probability of a cross succeeding fell to 0.005 at, or close to, 100 crosses. Once the calculated value of Px, the probability of cross x succeeding, fell below 0.005 the value of Px was taken as 0.005.

## Competing interests

The authors declare they have no competing interests.

## Authors’ contributions

JRW drafted the paper, developed the models in Excel, was manager of the UK Department for International Development (DFID) Plant Sciences Research Programme (PSRP) that funded most of this work, and was a collaborator on the breeding and varietal testing programmes in India, Nepal and Bangladesh. SG and MS were principal investigators on PSRP funded projects on rice breeding in Nepal. DSV was principal investigator on PSRP-funded projects in India. KDJ was initially principal investigator on PSRP funded projects in Nepal and then regional co-ordinator for PSRP projects in Nepal, Bangladesh, and India. All authors read and approved the final manuscript.

## Supplementary Material

Additional file 1 ** A proposal for the release of rice variety Barkhe 3004 (a rice variety developed using client-oriented breeding [COB]) approaches.** Pokhara, Nepal: LI-BIRD; Hardinath, Nepal: NRRP; Chitwan, Nepal: Jaskelo Youth Club; Bangor, UK: CAZS-NR, University of Wales; 2006. Click here for file

Additional file 2 ** A proposal for the release of an aromatic rice variety Suanulo Sugandha.** Application to be made for approval and release of crop varieties to Government of Nepal. Ministry of Agriculture and Cooperatives, National Seed Board; 2008. Click here for file

Additional file 3 ** Impact of New Upland Rice Varieties in Eastern India from Client-Oriented Breeding: Evidence from Whole Village Surveys.** Bangor, UK: Centre for Arid Zone Studies (CAZS), University of Wales; 2004. Click here for file

Additional file 4 ** The impact of new maize and rice varieties on the livelihoods of poor farmers in marginal agricultural areas of western India.** In Livelihoods Summit. Udaipur, Rajasthan: Department for International Development (DFID) and Indian Farm Forestry Development Corporation (IFFDC) 27th-30th September; 2005. Click here for file

Additional file 5 ** New Upland Rice Varieties for India.** Rainfed Agriculture Impact Study No. 1. Monitoring Impact Assessment and Learning Component (MIL) of the Research into Use Programme (RiU); 2008. Click here for file

Additional file 6 ** New Rice Varieties for Nepal.** Rainfed Agriculture Impact Assessment Study No. 2. Monitoring Impact Assessment and Learning Component (MIL) of the Research into Use Programme (RiU); 2008. Click here for file

Additional file 7 ** New Rice Varieties for Bangladesh from Client-Oriented Breeding.** Rainfed Agriculture Impact Assessment Study No.3. Monitoring Impact and Learning (MIL) Component of the Research into Use (RiU) Programme; 2008. Click here for file
