# Supplementary material for: Plant breeding can be made more efficient by having fewer, better crosses
Source: BMC Plant Biol. 2013 Feb 7;13:22. doi: 10.1186/1471-2229-13-22 (PMC3621587; doi:10.1186/1471-2229-13-22)
Supplement: Additional file 1 — A proposal for the release of rice variety Barkhe 3004 (a rice variety developed using client-oriented breeding [COB]) approaches. Pokhara, Nepal: LI-BIRD; Hardinath, Nepal: NRRP; Chitwan, Nepal: Jaskelo Youth Club; Bangor, UK: CAZS-NR, University of Wales; 2006. [file 1471-2229-13-22-S1.pdf]

Monitoring Impact and Learning (MIL) Component of the Research into Use (RiU) Programme

New Rice Varieties for Bangladesh from Client-Oriented Breeding.

Rainfed Agriculture Impact Assessment Study No.3

Table of Contents

Executive summary 4

Background to the study 5

Objectives of the study 7

Methodology of the study 8

Group discussion 9

Results and discussion 11

Results from the first set of group discussion 11

Results from the second set of group discussion 13

Benefits to households 15

What has been learned about the use of COB varieties? 16

Environmental dimension of the innovation 16

What has been learned that will be valuable in any future Bangladesh work? 16

References 17

Appendix 1 *Addressing social exclusion and poverty through rice varieties bred using Client-oriented breeding*. 18

Appendix 2 *Sampling frame for 65 villages in three districts of HBT Bangladesh* 19

Appendix 3 *Checklist for group discussion* 21

ACRONYMS

CAZS NR	CAZS Natural Resources
CBOs	Community Based Organizations
COB	Client-Oriented Breeding
DAE	Department of Agriculture Extension
DFID	Department for International Development (of the UK)
GD	Group Discussion
GOs	Government Organizations
HBT	High Barind Tract, Bangladesh
IRD	Informal Research and Development
LI-BIRD	Local Initiatives for Biodiversity, Research and Development
MIL	Monitoring Impact and Learning
NGOs	Non-government Organisations
PCI	Participatory Crop Improvement
PPB	Participatory Plant Breeding
PROVA	Peoples Resource Oriented Voluntary Association
PSP	Plant Sciences Research Programme
PVS	Participatory Varietal Selection
RiU	Research into Use
RNRRS	Renewable Natural Resources Research Strategy

New rice varieties for Bangladesh from client-oriented breeding (COB)

Executive Summary

- A rapid impact assessment was done in the High Barind Tract (HBT) of Bangladesh on three rice varieties Judi 567, Judi 582 and Barkhe 3004 bred using client-oriented breeding (COB) in RNRRS projects. The study was implemented in 24 villages of 3 districts of the HBT, i.e. Rajshahi, Chapai Nawabganj and Naogaon, using group discussion.
- Of the three varieties evaluated, Judi 567 and Barkhe 3004 had been adopted both during the t. *aman* and the *boro* season.
- Judi 567 was adopted for upland and medium lands while Barkhe 3004 was adopted for medium and lowland rice ecosystems. These adoption patterns were mainly determined by the crop duration of a particular variety fitting to a particular domain.
- Adoption of Judi 582 had been declining due to lack of any obvious advantage either with the existing farmers varieties or with Judi 567 and Barkhe 3004.
- In around one third of the group discussions, the participants reported increases in rice yield of about 30%. Increased availability of food was reported in almost all the discussions and participants also mentioned better nutrition during most group discussions.
- Participants in about a quarter of the group discussions reported that increased integration of *rabi* crops, made possible by the introduction of short duration rice varieties, contributed to a 100% increase in cropping intensity in more than 50% of previously fallow land.

New rice varieties for Bangladesh from client-oriented breeding (COB)

INTRODUCTION

The Barind tract, situated in the greater Rajshahi region of Bangladesh, consists of plain and undulated areas covering three districts; Rajshahi, Chapai Nawabganj and Naogaon. The undulated Barind, called the high Barind tract (HBT), comprises roughly 21% of the whole Barind with a total area of about 1600 km^2 (160, 000 ha). This is the most marginal area of the Barind Tract for rainfed cropping, accounting for 21% of the drought-prone lowland rice land in Bangladesh (Saha, 2002, Riches, 2008).The HBT area is situated above normal flood level. In most situations sloping land has been terraced to hold rainwater for rice cultivation.

The HBT is the driest part of the country with an annual rainfall ranging from 1000 -1400 mm, mainly concentrated during June to October but there is great variability in total rainfall, timing of onset and cessation of the rains as well as in-season drought periods. In general, with the cessation of the monsoon rains, the moisture quickly dries out and the soil turns very hard, making sowing and establishment of rainfed rabi crops extremely difficult.

The topsoil consists of grey silt loam to silty-clay loam and has a compact 6-8 cm thick plough pan and low organic matter (0.8-1.2%). Permeability and moisture-holding capacity is also low. The soil becomes soft and sticky in the rainy season and after that quickly dries out and soon cracks (Idris, 1990). The topsoil is strongly acidic (pH 4.5 - 5.5) and the subsoil is slightly acidic to neutral (pH 6.0-7.0).

Rainfed transplanted *aman* (T. *Aman*) rice during the rainy season is the predominant crop with the fields remaining fallow during the following *Rabi* season. Rice is the staple food in the area and farmers are largely dependent on this crop. Some farmers grow broadcast or transplanted *Aus* rice with the onset of pre-monsoon rains and after harvesting *Aus*, they grow mainly local varieties of T. *aman* rice (Joshi, et. al., 2005). Attempts to increase the productivity of the High Barind needs to be focused on rainfed lands by improving the reliability and yields of *aman* rice while increasing total system productivity by increasing the total area planted to rabi crops (Riches, 2008).

A number of rice varieties that were either identified by the PSP-funded Participatory Crop Improvement (PCI) Project in Nepal or bred by client-oriented breeding (COB) in Nepal were introduced into Bangladesh. The objective of this initiative was to offer a wider choice of short- and medium duration rice varieties to the farmers in the HBT.

The HBT area of Bangladesh, whose population exceeds 2.5 million, is one of the poorest regions in Bangladesh (Appendix 1). Around 90% of people in the HBT live on less than $2 a day. Only 10% of people, particularly absentee landlords, are rich. At least 90% of the poor depend on agriculture for their livelihoods, either from subsistence farming on small land holdings and/or from service as agricultural labourers. There is an increasing trend of males temporarily leaving the area seeking employment. Substantial increases in rice grain yields or large improvements in rice grain quality that result in a considerably higher market price can significantly contribute to improvement in the livelihoods of poor people. The urban poor, rural landless and those with small land holding are also highly vulnerable to increases in the price of rice so greater production and stability of production helps them greatly. Due to low probability of significant rainfall

after mid-October, some 80% of the area currently lies fallow in the *rabi* season (Riches, 2008).

The NGO, the Peoples Resource Oriented Voluntary Association (PROVA), that was based in the High Barind Tract implemented with CAZS-NR two RNRRS/PSP funded projects for the intensification of cropping in rice fallow areas of the HBT (Table 1). This involved identification of new chickpea varieties suitable for the region, developing agronomic practices including seed priming with plain water and some micro-nutrients, seed production and preservation technologies. These projects also looked at other alternative winter crops that can be grown in place of chickpeas, e.g. barley, linseed, lentil, and lupin.

Integrating *rabi* crops after rice is not easy for a number of reasons. For example, rice paddies are unsuitable for crops requiring aerobic environments, long duration rice varieties mature beyond the optimum time for planting of rabi crops and delayed planting of rabi crops results in reduced yields due to poor stand establishment and/or due to limited biomass formation.

In this context, rice varieties developed using client-oriented breeding (COB) in RNRRS/PSRP funded projects in Nepal were introduced (all of which were short- to medium duration varieties) into Bangladesh for participatory evaluation and scaling out to improve overall returns to farmers from rice as well as winter crops (Table 1). This initiative on rice COB was carried out by PROVA and CAZS-NR within the funding framework of the *rabi* cropping projects without any additional funding for these activities. The Department of Agriculture Extension (DAE) and a few NGOs also collaborated in conducting demonstrations and Informal Research and Development (IRD) i.e., the distribution of packets of seed to farmers for evaluation and farmer-to-farmer dissemination while minimising the transaction costs to the distributing agency.

Table 1. Timeline of major events in PVS and COB in Bangladesh

Year	Event
1999	• RNRRS project R7450 on 'Promotion of chickpea following rainfed rice in the Barind area of Bangladesh' started jointly implemented by PROVA and CAZS NR
2002	• Follow-on project R8269 on 'Improvement of rainfed cropping systems in the High Barind Tract of Bangladesh' started.<br>• First COB variety from Nepal tested in PVS trials
2004	• Number of PVS and IRD trials conducted on COB varieties<br>• Orienting various government line agencies and NGOs on the concepts, approaches and benefits of participatory approaches and discussion of possible collaboration for PVS, IRD and demonstrations
2005	• A stakeholders' meeting held in Dhaka to plan and arrange collaboration for testing and promotion of COB lines<br>• Scaling up of first promising varieties from COB began on a small scale through DAE and a few NGOs
2006	• DAE started Demonstrations on COB varieties in the *Boro* season 2006-07
2007	• DAE organised demonstrations of Barkhe 3004 in a number of locations I the T. *aman* season

This report examines from the total of 19 varieties that were introduced from Nepal (Table 2) the uptake and impact of three COB rice varieties (Table 3). These three rice varieties were all from the COB breeding programme in Nepal and were the three varieties that performed best in the trials. One of them, Judi 582, was from the cross

Kalinga III/Radha 32 while the remaining two, Judi 567 and Barkhe 3004, were from the cross Kailnga III/IR64. This was later restricted just to Judi 567 and Barkhe 3004 as these two were the most widely accepted varieties. Other COB varieties, such as Judi 572 and Barkhe 2001, which may have been used to some extent, were not included in the study.

Table 2. Short- and medium-duration rice varieties introduced into Bangladesh and tested in Mother and Baby trials, informal research and development (IRD), and seed production in 2003 and 2004.

**PVS identified Nepal**	**Year introduced**	**COB varieties Nepal**	**Year introduced**	**COB varieties India**	**Year introduced**
BG 1442	2002	Sugandha 1	2002	Ashoka 200F	2004
Ekahattar	2002	Barkhe 2001	2003	Ashoka 228	2004
Judi 582	2002	Judi 565	2003		
Pant Dhan 10	2002	Judi 566	2003		
PNR-381	2002	Judi 567	2003		
Sarwati	2002	Barkhe 1006	2004		
		Judi 141F	2004		
		Judi 572	2004		
		Barkhe 3004	2004		
		Super 3004	2004		
		Sunaulo Sugandha	2004		

Table 3. Major traits of the three COB rice varieties, Judi 567, Judi 582 and Barkhe 3004 included in the study

**Varietal traits**	**Judi 582**	**Judi 567**	**Barkhe 3004**
Seasonal adaptation	T. *aman* and *boro*	T. *aman*, *boro*, *aus*	T. *aman* and *boro*
Toposequence† adaptation	Upper and middle	Upper	Middle and lower
Yield advantage over best check T. *aman*	33% (additional 0.9 t ha^{-1}) over mean of Swarna and BRRIdhan 32 (n=22) during T. *aman*	48% (additional 1.13 t ha^{-1}) over mean of Swarna, BRRIdhan 32 and BRRIdhan 39 (n=8)	5.0-5.5 t ha^{-1}
Yield advantage over best check *boro*	37% (additional 1.49 t ha^{-1}) yield over the mean of check varieties BRRIdhan 28 and Miniget (n=29)	20% (additional 0.81 t ha^{-1}) yield over the mean of check varieties BRRIdhan 28 and Miniget (n=29)	5.50-6.0 t ha^{-1}
Time to maturity during T. *aman*	120 days<br>Swarna: 150 days	115 days<br>Swarna: 150 days	130 days<br>Swarna: 150 days
Time to maturity during *boro*	155 days<br>BRRIdhan 28= 145 days	145days<br>BRRIdhan 28:145 days 125days at *Aus* Pariza: 120 days	155 days<br>Swarna not grown in *boro*

†Toposequence is the continuum of rice cultivation from the upper to the lower land
Sources: Joshi et.al, 2005, 2007, 2008

The study looked at technological innovations that were the outcomes of RNRRS projects. The overall objective was to estimate the extent of adoption of COB rice varieties in the t. *aman*, *aus* and *boro* seasons in selected villages of the HBT of Bangladesh.

METHODS

The sampling frame was prepared during early December 2008 and comprised 65 villages from three districts of the HBT (Appendix 2). These villages were identified using the records maintained by PROVA as these villages that either had one-time Informal Research and Development (IRD) from 2004 to 2005 or seed multiplication activities from 2003 to 2005 or both, involving at least one rice variety from COB. A few of these villages also had mother trials (a single replicate of all the entries grown in a farmer's field) and baby trials (a single entry of the mother trial grown by a farmer alongside the currently best variety that the farmer grows) or both (data not shown). These villages are suitable for growing rice crops in all three seasons (T. *aman*, *boro* and *aus*) except for three villages which were not suitable for growing *boro* rice because of lack of irrigation (Appendix 2). For conducting group discussions (GD), eighteen villages out of 65 were randomly selected by the RiUP Cluster Study Team Manager. The objective of the group discussions was to determine the current COB rice users and non-users for the year 2008 (Figure 1).

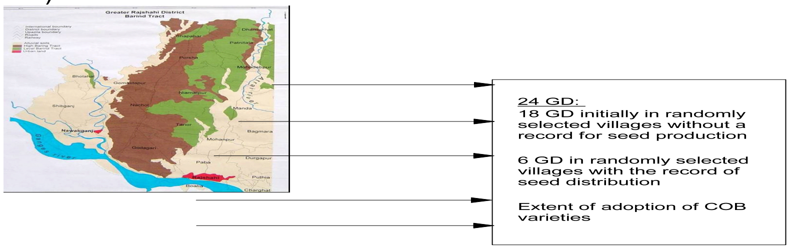

Figure 1. Locations in High Barind Tract where group discussions (GD) were conducted during impact assessment of COB rice varieties.

The first set of 18 villages (Table 4) were randomly selected from a total list of 65 villages where PROVA had activities in the High Barind Tract without any consideration of seed supply of the COB varieties. No seed of Barkhe 3004 had been distributed at all in these villages and seed of the other two varieties was limited. Following the discussion of the results of these initial group discussions it was decided to conduct additional group discussions in the villages for which seed distribution records existed

although the volume of seeds given out to any villages were very limited at this early stage of what was a research project. Hence, six further group discussions were held in six new villages randomly selected by the RiUP Cluster Study Team Manager out of the 29 villages, from the total of 65, where seeds of either of the two COB varieties, e.g. Judi 567 or Barkhe 3004 or both had been distributed (Table 5). All of the six villages had land suitable for growing both T. *aman* and *boro* rice (Appendix 2).

Table 4. Seed quantities (kg) of three COB varieties distributed in 18 randomly selected villages from 2003 up to 2005.

		Quantity (kg)		
**District**	**Village**	**Barkhe 3004**	**Judi 567**	**Judi 582**
Rajshahi	Baksuil	0	0	5
Rajshahi	Banglakandor	0	135	400
Rajshahi	Chandlai	0	1	204
Rajshahi	Daknipara	0	0	10
Rajshahi	Dangpara	0	0	5
Rajshahi	Gikrapara	0	0	10
Nawabganj	Borenda	0	1	0
Nawabganj	Dhinagor	0	0	20
Nawabganj	Kanpara	0	0	10
Nawabganj	Palpara	0	0	0
Nawabganj	Paul	0	5	0
Nawabganj	Rahimpara	0	0	5
Naogaon	Ghatnagro	0	10	0
Naogaon	Manikora	0	0	10
Naogaon	Nimdighe	1.5	5	0
Naogaon	Sharandopur	0	6	0
Naogaon	Tajpur	1.5	27	10
Naogaon	Vioil	0	4	0
	Grand Total	3	194	689

Table 5. Amount of seed (kg) of Judi 567 and Barkhe 3004 distributed from 2003 to 2005. Randomly selected villages for the six further group discussions indicated in bold.

District	Village	Judi 567 in 2004 (kg)	Judi 567 in 2005 (kg)	Barkhe 3004 in 2005 (kg)
Naogoan	**Chakla**		9	0
	**Gangoir**		10.5	0
	Shuhati		6	0
	Ghatnagor		15	0
	Nimdighe		7.5	1.5
	Paul		6	0
	Sharandopur		9	0
	Tajpur		33	1.5
	Viol		6	0
Nawabgonj	**Adda**		13.5	0
	**Gonoer**		7.5	0
	Seerpur		24	0
	Barenda		2	0
	Chandlai		2	0
Rajshahi	**Chhabbisnagor**	15	293	15
	**Bijoynagor**		180	50
	Banglakandor		140	0
	Dangpara		0	0
	Birail		200	0
	Dhamdum		1.5	0
	Digram		0	50
	Hajipara		10	0
	Hotathpara		86.5†	0
	Malo para		11††	0
	Mondomala		8	0
	Rishikul		50	0
	Sishapara		7.5	0
	Telopara		10.5	3
	Tilahari	16.5	10.5	0
	Total	31.5	1159.5	121

†From 2003 to 2005
†† distributed 4.5 kg in t. amnan and 7.5 kg in *boro* season

Group discussions were conducted in the 24 sampled villages; 18 for the first time and 6 for the second set of group discussions. The survey was implemented by the PROVA team and locally recruited enumerators. At each GD, 10-15 farmers (mostly males) participated, including key informants. Participants were drawn from poor and rich categories. Since, the GDs were conducted during the time of peak rice threshing and winter crop planting and participation of farmers in all the GDs was not very satisfactory as in some instances some of the participants left the meeting half way through to attend some of their own pressing work, and at times the FGD were done with the participants from a small portion of a village rather than the entire village. The GDs were conducted using a checklist (Appendix 3). A social map of each village was drawn with the help of key informants and participants to demarcate village boundaries and indicate the

position of individual households (Figure 2). This list of households so prepared contained both farming and non-farming families. From this list, households not involved in farming were excluded to leave the framework for determining users or non-users of COB rice varieties.

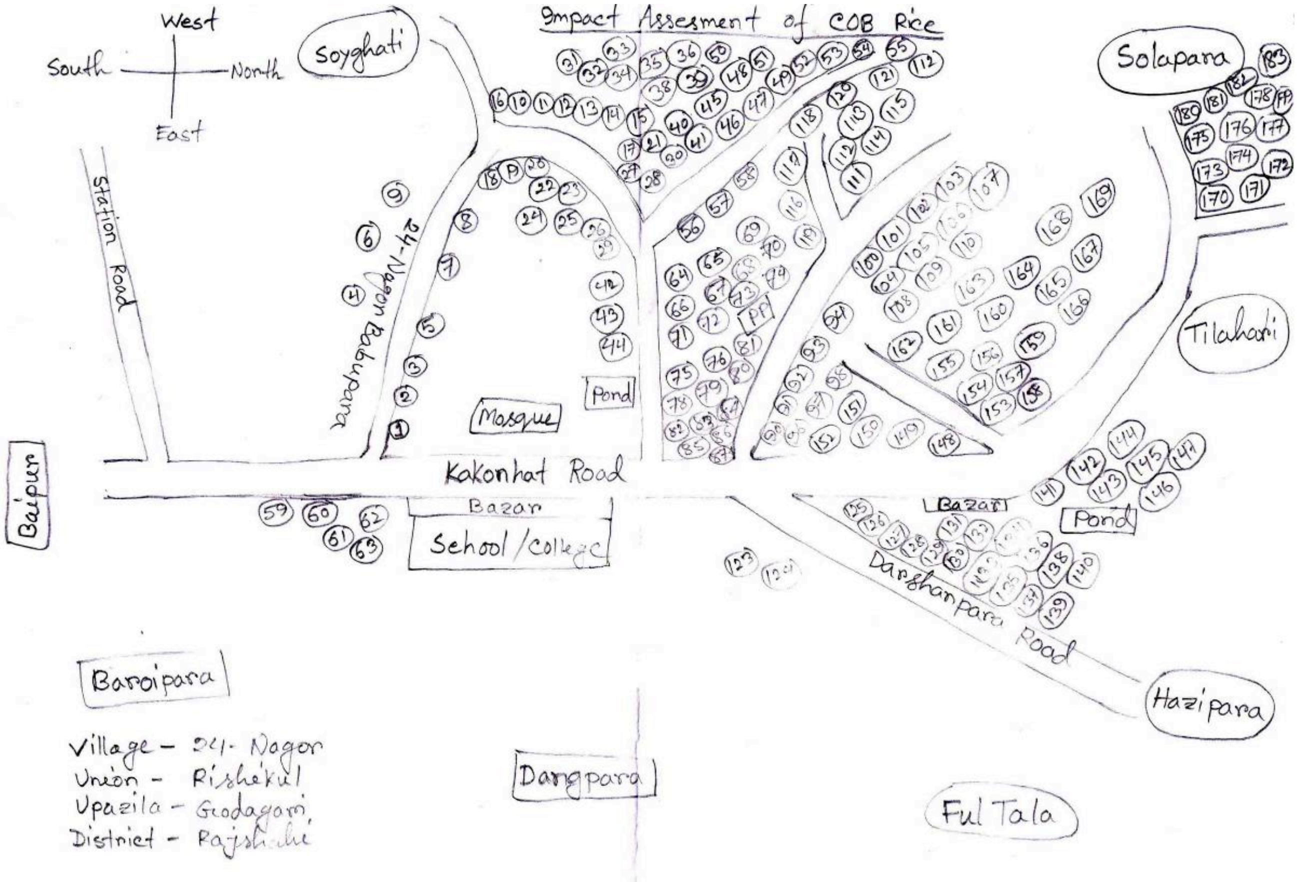

Figure 2. A social map of Chhabisnagor, Rajshahi showing the village boundary and settlement

Following this, a list for farmers who were users or non users of COB was prepared using the knowledge of the participants about the farmers, their rice area and rice varieties. A current user was defined as a farmer who grew at least one COB variety during one of the three rice seasons of 2008.

RESULTS AND DISCUSSION

The first group discussions in 18 villages

Of the 18 villages where initial group discussions were conducted, very limited quantities of seed of Barkhe 3004 were distributed by PROVA in only two villages (Table 6), Judi 567 seeds were distributed in 50% of these villages (Table 7), while seeds of Judi 582 were distributed to 12 out of 18 villages (Table 8).

Barkhe 3004 was grown in two villages (Table 6) where only 3 kg seeds of this variety had been distributed. However, adoption of this variety was reported from two villages where seed had not been distributed. This demonstrates that farmer-to farmer spread had taken place as during the initial stage of testing as there was no other source of

seed. Barkhe 3004 was used most in medium land as its maturity period of 130 days (Table 3) fits well for this situation.

Table 6. Amount of seed distributed and household use (%) of Barkhe 3004 by households in upland, medium and lowland in eighteen villages in 2008

Village	Number of farming households (N)	Seed quantity (kg)	User households (%)		
			Upland	Medium land	Lowland
Banglakandor	153	0	5	5	3
Dangpara	95	0	0	10	0
Nimdhige	37	1.5	0	0	0
Tajpur	62	1.5	0	0	0
Remaining 14 villages†		0	0	0	0

†Baksuil, Borenda, Chandlai, Daknipara, Dhinagor, Ghatnagro, Gikrapara, Kanpara, Manikora, Palpara, Paul, Rahimpara, Sharandopur, Vioil

Judi 567 was reported to be grown in three out of the 18 villages (Table 7) and its use was not correlated with original seed supply, e.g., a high proportion of households grew it in Dangpara where farmers had not originally received seed from the project. Judi 567 was grown only on medium and upland conditions due to its early maturity and relative drought tolerance and is unsuitable for lowlands. If this short-duration variety were to be grown in the lowlands it would have to be harvested, dried and threshed while there was still water in the fields.

Table 7. Amount of seed distributed and household use (%) of Judi 567 by households in upland and medium land in eighteen villages in 2008.

Village	Number of farming households (N)	Seed distributed (year)	Seed quantity (kg)	User households (%)	
				Upland	Medium land
Banglakandor	153	2005	140	40	35
Dangpara	95	§	0	40	20
Barenda	52	2005	2	5	0
Chandlai	40	2005	2	0	0
Ghatnagro	74	2005	15	0	0
Nimdighe	37	2005	7.5	0	0
Paul	36	2005	6	0	0
Sharandopur	73	2005	9	0	0
Tajpur	62	2005	33	0	0
Vioil	30	2005	6	0	0
Remaining 8 villages†		§	0	0	0
Grand Total			220.5		

†Baksuil, Daknipara, Dhinagor, Gikrapara, Kanpara, Manikora, Palpara, and Rahimpara
§ Seed not distributed

Judi 582 was the least grown of the three COB varieties despite its seed being distributed in 12 out of the 18 villages (Table 8). The reasons reported by the participants for the decline in use compared with those reported by Pandit (2008) were:

- This is about 10 days later than both Judi 567 and BRRIdhan 28 but does not give an adequate extra yield (compared with these two varieties) to justify this

additional time. Unfavourable weather affects the harvesting and threshing of any longer duration variety and farmers are not prepared to take the risk for limited potential gains.

- During the t. *aman* season it has too short a duration for the lowlands as it matures nearly three weeks earlier than the most widely grown lowland varieties Swarna and BR 11.
- Judi 582 yields nearly 20% less than Barkhe 3004 with similar maturity. In *boro* season maintaining a rice variety for 10 more days (compared with Judi 567 and BRRIdhan 28) means at least two extra irrigations at considerable cost.
- If seeds of Judi 582 from the *boro* season are used in the following *boro*, the germination is really low-only about 20% seeds germinate and Judi 582 is not one of the preferred varieties for *Aus* due to its longer maturity. Conversely, Judi 567 is suitable to grow in *aus* season (needs to be stored for less time) so germination is not a problem. Seeds of Barkhe 3004 from t. *aman* can be easily used for *boro* season as the crop matures in good time for using seed for *boro* planting and there is no problem of seed dormancy in this variety.
- Grain quality of Judi 582 is not as good as that of Judi 567 or Barkhe 3004.

Table 8. Amount of seed distributed and household use (%) of Judi 582 by households in upland and medium land in eighteen villages in 2008.

Village	Number of farming households (N)	Seed distributed (year)	Seed quantity (kg)	User households (%)	
				Upland	Medium land
Vioil	30	§	0	5	10
Baksuil	140	2004	5	0	0
Banglakandor	153	2004	110	0	0
Chandlai	40	2004	3	0	0
Daknipara	47	2005	10	0	0
Dangpara	96	2004	5	0	0
Dhinagor	79	2003	20	0	0
Gikrapara	115	2005	10	0	0
Kanpara	23	2005	10	0	0
Manikora	56	2005	10	0	0
Rahimpara	25	2005	5	0	0
Sharandopur	73	2005	9	0	0
Tajpur	62	2005	9	0	0
Remaining 5 villages†		§	0	0	0
Grand Total			206		

†Barenda, Ghatnagro, Nimdighe, Palpara, Paul
§ Seed not distributed

The second set of group discussions in six villages

Out of the six randomly selected villages, seeds of Barkhe 3004 were distributed only in two villages and the quantities of seed distributed were low (Table 9). Its adoption was considerable both in the T. *aman* and *boro* seasons in these two villages, Chhaubisnagor and Bijoynagor, considering the modest quantities of seeds that were distributed.

The highest adoption of this variety was in Chhaubisnagor during T. *aman*. According to the key informants of GDs, this variety is gradually becoming popular during the t. *aman* season as a replacement for BR 11 and Swarna, both of which are currently the most widely grown varieties in t. *Aman* in the area. In addition, it is liked for cultivation in the *boro* season in both of the villages.

High use of this variety in the both the villages could be explained by three factors:

- Most of the research and seed production of COB rice by PROVA were done in Chhaubisnagor and Bijoynagor .It is likely that seed of Barkhe 3004 spread in the villages through the research activities, although it was distributed in a limited quantity as a part of promotional activities, e.g. IRD and to some extent, seed production;
- Farmer-to-farmer spread through rapid multiplication of seeds in both T. *aman* and *boro* season;
- Possible, overestimates by the participants of the discussion group as they may not have been selected to represent the entire village

Table 9. Amount of seed distributed and household use (%) of Barkhe 3004 by households iin the T. *aman* and *boro* seasons in six villages in 2008.

**Village**	**Number of farming households (N)**	**Seed distributed (year)**	**Seed quantity (kg)**	**Farmers in GD (N)**	**User households (%)**	
					**T. *aman***	***Boro***
Chhaubisnagor	167	2005	15	14	64	50
Bijoynagor	35	2005	50	15	33	20
Adda	33	§	0	11	0	0
Gonoer	60	§	`0	11	0	0
Chakla	42	§	0	10	0	0
Gangoir	46	§	0	14	0	0

§ seed not distributed

Judi 567 was adopted in all the three rice growing seasons (Table 10) and was adopted in four out of the six villages in the t. *aman* and *boro* seasons despite the small amounts of seed distributed in all villages apart from Chhabisnagor and Bijoynagor.

Key informants during the discussion revealed that Judi 567 is the most widely used of the COB varieties. Earlier studies done on this variety revealed that it is preferred for its high yield, early maturity and lower water requirements.

Table 10. Amount of seed distributed and household use (%) of Judi 567 by households in the *aus,* T. *aman* and *boro* seasons in six villages in 2008.

Village	Number of farming households (N)	Seed distributed (year)	Seed quantity (kg)	Farmers in GD (N)	User households (%)		
					Aus	**T. *aman***	***Boro***
Chhaubisnagor	167	2004, 2005	308	14	57	57	50
Bijoynagor	35	2005	180	15	0	20	7
Adda	33	2005	13.5	11	0	18	18
Gonoer	60	2005	7.5	11	0	0	0
Chakla	42	2005	9	10	0	0	0
Gangoir	46	2005	10.5	14	0	14	14

Judi 582 was reported from the small sample of respondents in only one village (Chhaubisnagor) where it was grown in *aus* and t. *aman* seasons (Table 11). During *boro*, wherever it is grown, It has a similar maturity to Barkhe 3004 during the *Boro* season but yields less.

Table 11. Household adoption percentage for Judi 582 in *Aus*, t. *aman* and *boro* seasons in 2008

Village	Number of farming households (N)	Seed distributed (year)	Seed quantity (kg)	Farmers in GD (N)	User households (%)		
					Aus	**t. *aman***	***Boro***
Chhaubisnagor	167	2003 to 2005	741	14	14	21	0
Bijoynagor	35	§	170	15	0	0	0
Adda	33	§	9	11	0	0	0
Gonoer	60	2005	7.5	11	0	0	0
Chakla	42	2005	3	10	0	0	0
Gangoir	46	2005	33	14	0	0	0

Benefits

To households

The following responses were most common from the participants of the group discussion in all the villages.

- In almost one third of the group discussions, the participants reported an increase in rice yield of about a third on average.
- Increased integration of *rabi* crops due to the introduction of short duration rice varieties contributed to a 100% increase in cropping intensity in >50% of previously fallow land was reported by the participants in about a quarter of the group discussions.
- Increased availability of food was reported in almost all the discussions and participants also mentioned better nutrition during most GDs.
- Increased income from increased grain sales resulting in improvements to the living conditions and education level of children.
- Increased employment due to increase in cropping intensity was also reported in all the GDs.
- Reduced production costs

To the environment

Use of short- to medium duration COB varieties resulted in significant savings on irrigation water due to their drought tolerance which contributed in preserving natural resources, e.g. ground water to some extent and also reducing the environmental pollution that may accrue from the use of diesel engines to pump ground water. The insect-pest tolerance and the higher yields under unchanged levels of inputs of these COB varieties are also of great import (Pandit, 2008).

What has been learnt about the use of COB varieties?

- Adoption and uptake of varieties in Bangladesh in all the three seasons and across three rice domains that were developed in Nepal using COB approaches suggests that COB is successful in developing widely adapted rice varieties.
- Use of PVS is cost effective in testing and promoting rice varieties rapidly.
- Adoption is at a very early stage so evaluating the extent of adoption and spread of COB rice varieties in Bangladesh can only indicate their potential. This is particularly true because of the very limited efforts that have gone into seed production and promotion.
- However, villages that have had no formal access to seed can also have high levels of adoption if farmers learn about the varieties and get access to seed through their own innovation system.
- Of the three varieties, the two that have been adopted most can be grown in two or more seasons:
    - Judi 567 in the *Aus*, *Aman* and *Boro*
    - Barkhe 3004 in the *Aman* and *Boro*
- Focus on large scale seed production and marketing is essential if the benefits of new COB rice varieties are to be scaled up. But this has some important considerations for the seed regulatory framework of Bangladesh. Efforts are needed to initiate the registration process of at least two COB rice varieties, e.g. Judi 567, Barkhe 3004.
- Judi 567 and Barkhe 3004 are both highly suitable candidates for further dissemination including their formal registration or even release through the formal process
- The adoption of Judi 567 and Barkhe 3004 in more than one rice growing seasons in a year means that their dissemination can be a far more rapid process in Bangladesh than in Nepal where these varieties can, except in exceptional circumstances, only be grown in a single season per year.

References

Ahmed, A.U., Hill, R.V., Smith, L.C., Weismann, DM and Frankenberger, T (2007). The World's Most Deprived. Characteristics and causes of extreme poverty and hunger. Washington DC: International Food Policy Research Institute. www.ifpri.org

Idris, K.M. (1990). Barind Tract: Problems and potentials. Publication No 33. Soil Resource Development Institute, Rajshahi, Bangladesh

IFAD (2001). Rural Poverty Report 2001: The Challenges of Ending Rural Poverty. Oxford University Press.

Joshi, K.D., Musa, A.M., Johansen, C., Harris, D., Devkota, K.P., Gyawali, S. and Witcombe, J.R. (2005). Short duration rice varieties for the high Barind tract of Bangladesh: The initial impact of varieties from client oriented breeding and selection in Nepal. Version 2. Rajshahi, Bangladesh: PROVA and Bangor, Wales, UK: CAZS Natural Resources

Joshi, K.D. Musa, A.M. Johansen, C., Gyawali, S., Harris, D and Witcombe, J.R. (2007). Highly client-oriented breeding, using local preferences and selection, produces widely adapted rice varieties Field Crops Research 100: 107-116

Joshi, K.D., Musa, A., Johansen, C., Harris, D., Devkota, K.P., Gyawali, S. and Witcombe, J.R. (2008). Identifying rice varieties for the High Barind Tract of Bangladesh with farmers. In: CR Riches, D Harris, DE Johnson and B Brady (Editors) *Improving Agricultural Productivity in Rice-Based Systems of the High Barind Tract of Bangladesh*. Los Banos (Philippines): International rice Research Institute. 215 p.

Riches, C.R. (2008). Setting the scene. The High Barind Tract: A challenging drought-prone agricultural environment. In: CR Riches, D Harris, DE Johnson and B Brady (Editors) *Improving Agricultural Productivity in Rice-Based Systems of the High Barind Tract of Bangladesh*. Los Banos (Philippines): International rice Research Institute. 215 p.

Saha, A.K. (2002). Impact Assessment Study for the DFID Funded Project R7540, Promotion of Chickpea Following Rain fed Rice in the Barind Areas of Bangladesh.

Pandit, D.B. (2008). Adoption and Spread of Rice Varieties Bred by Client Oriented Breeding in High Barind Tract of Bangladesh in 2005 and 2006. Nashipur, Dinajpur: Wheat Research Centre, Bangladesh Agricultural Research Institute.

UNDP (2008). Bangladesh Human Development Report 2007/2008. http://hdrstats.undp.org/countries/country_fact_sheets/cty_fs_BGD.html

Appendix 1. *Addressing social exclusion and poverty through rice varieties bred using client- oriented breeding*

Bangladesh is one of the poorest countries in the world. Nearly 83% people live below $2 a day. The economy of Bangladesh is predominantly agrarian with agriculture sector accounting for 35% of Gross Domestic Products (GDP). Over 80% of the Bangladesh, roughly over 15 million households live in rural areas and agriculture sector employs over 62% of labour force.

One of the greatest challenges remains to be addressed in Bangladesh is the structural poverty of the country – the main factors of which are an unequal distribution of productive assets, inequitable distribution of income, massive under-employment and low levels of human resource development. In addition, a low level of law and order, endemic corruption and a lack of good governance also add to poverty and deprivation of common people (UNDP, 2008).

The characteristics of sharecroppers and landless people living in HBT Bangladesh (which is also true for other South Asian countries) are: being landless or limited access to land and other productive resources, larger families, higher dependency ratios, lower educational attainment and higher underemployment. The poor also lack basic amenities e.g. piped water supply, sanitation and electricity. They live in rural remote areas located furthest from roads, markets, schools and health services. These are food insecure households with limited access to credit and inputs and technology is severely limited and certain constraints such as lack of information about markets, lack of business and negotiating experiences and a lack of collective organisation deprive them of the power needed to interact on equal terms with others (IFAD, 2001, Ahmed et al., 2007).

In the absence of household survey, it is difficult to make any comparison between the growers and non-growers in terms of poverty characteristics. However, the following socio-economic characteristics apply to the majority of the farmers in the area and would also apply to the farmers adopting the COB varieties:

- A high proportion of sharecroppers
- Small land holdings
- Low inherent soil fertility with no or very low organic matter
- Limited access to available modern technologies
- Low education levels
- Little access to forests and an acute shortage of fuel

Women's Perspective

Increased yields from COB varieties can generate more on farm employment in different seasons for landless women particularly from *Adibashis* during harvesting, threshing, winnowing, drying and seed preservation. Muslim women contribute in post harvest operation and also in the evaluation of technologies

Appendix 2. *Sampling frame for 65 villages in three districts of HBT, Bangladesh, 2008*
(Villages had IRD (2004-05) or Seed Multiplication Activities (2003-05) or both (1= activity, 0 = no activity

S .No	District	Village	IRD	Seed	Total	T. *aus*	T. *aman*	*Boro*
1	Naogoan	Amda	1	0	1	√	√	
2	Naogoan	Chakla	1	0	1	√	√	√
3	Naogoan	Gangoir	1	0	1	√	√	√
4	Naogoan	Ghatnagor	1	0	1	√	√	√
5	Naogoan	Manikora	1	0	1	√	√	
6	Naogoan	Nimdighee	1	0	1	√	√	√
7	Naogoan	Sharandhapur	1	1	2	√	√	√
8	Naogoan	Shuhati	1	0	1	√	√	√
9	Naogoan	Tajpur	1	0	1	√	√	√
10	Naogoan	Telipara	1	0	1	√	√	
11	Naogoan	Vioil	1	0	1	√	√	√
12	Nawabgonj	Adda	1	0	1	√	√	√
13	Nawabgonj	Azipur	1	0	1	√	√	√
14	Nawabgonj	Baipur	0	1	1	√	√	√
15	Nawabgonj	Barenda	1	0	1	√	√	√
16	Nawabgonj	Bhabuk	1	0	1	√	√	√
17	Nawabgonj	Bongpara	1	0	1	√	√	√
18	Nawabgonj	Dhinagor	1	0	1	√	√	√
19	Nawabgonj	Gonoer	1	0	1	√	√	√
20	Nawabgonj	Kanpara	1	0	1	√	√	√
21	Nawabgonj	Nasirabad	1	0	1	√	√	√
22	Nawabgonj	Palpara	1	0	1	√	√	√
23	Nawabgonj	Paul	1	0	1	√	√	√
24	Nawabgonj	Rahimpara	1	0	1	√	√	√
25	Nawabgonj	Sherpur	1	0	1	√	√	√
26	Rajshahi	Chabbish Nagor	1	1	2	√	√	√
27	Rajshahi	Agrampur	0	1	1	√	√	√
28	Rajshahi	Baksuil	1	0	1	√	√	√
29	Rajshahi	Banglakandar	1	1	2	√	√	√
30	Rajshahi	Baroipata	1	0	1	√	√	√
31	Rajshahi	Bautia	0	1	1	√	√	√
32	Rajshahi	Bejoy Nagor	1	0	1	√	√	√
33	Rajshahi	Bipur	1	1	2	√	√	√
34	Rajshahi	Birail	0	1	1	√	√	√
35	Rajshahi	Bordhama	0	1	1	√	√	√
36	Rajshahi	Chandlai	1	1	2	√	√	√
37	Rajshahi	Chrisnopur	1	0	1	√	√	√
38	Rajshahi	Daknipara	0	1	1	√	√	√
39	Rajshahi	Dangpara	1	1	2	√	√	√
40	Rajshahi	Dhamdum	1	0	1	√	√	√
41	Rajshahi	Digram	1	1	2	√	√	√
42	Rajshahi	Gikrapara	0	1	1	√	√	√
43	Rajshahi	Hajipara	0	1	1	√	√	√
44	Rajshahi	Hotathpara	1	1	2	√	√	√
45	Rajshahi	Kadmapara	1	1	2	√	√	√

46	Rajshahi	Kalidaspur	0	1	1	√	√	√
47	Rajshahi	Kochia para	1	0	1	√	√	√
48	Rajshahi	Malo para	1	0	1	√	√	√
49	Rajshahi	Mirpur	1	0	1	√	√	√
50	Rajshahi	Mohadebpur	1	1	2	√	√	√
51	Rajshahi	Mondomala	1	0	1	√	√	√
52	Rajshahi	Murgidanga	0	1	1	√	√	√
53	Rajshahi	Nobogram	1	1	2	√	√	√
54	Rajshahi	Pachondo	1	0	1	√	√	√
55	Rajshahi	Rajabari	1	0	1	√	√	√
56	Rajshahi	Rishikul	0	1	1	√	√	√
57	Rajshahi	Sholapara	0	1	1	√	√	√
58	Rajshahi	Sishapara	1	1	2	√	√	√
59	Rajshahi	Soighati	1	1	2	√	√	√
60	Rajshahi	Tatulia	1	1	2	√	√	√
61	Rajshahi	Tatuliadanga	0	1	1	√	√	√
62	Rajshahi	Telopara	1	0	1	√	√	√
63	Rajshahi	Tilahari	1	1	2	√	√	√
64	Rajshahi	Vimpara	1	0	1	√	√	√
65	Rajshahi	Zani	0	1	1	√	√	√
	Grand Total		51	29	80			

Appendix 3. Check list of the group discussion

1. ***Setting the context for group discussion***

- **Demarcate the village boundary involving Key Informants from the locality**
- **Prepare rough village map (also indicate the position of the houses in the map)**
- **Prepare the list of households then disaggregate them by adopter and non-adopter (use table given on page 5)**

Document following information:

Demography

Land type

Soil type

Irrigation facility

Major crops grown

2. ***What rice varieties are grown in your village?***

Season	Upper topo sequence		Medium topo sequence		Lower topo sequence	
	Varieties	% Area coverage	Varieties	% Area coverage	Varieties	% Area coverage

3. Impact assessment of COB rice varieties

3. 1. Where have you distributed COB rice seeds?

Village Variety Seed quantity (kg)

2 .Where are the COB varieties currently grown?

Village	Variety	Area (Bigha)§

§ = 1 Bigha = 1330 m^2

3. How are the villages connected where seeds have been distributed and COB varieties are currently grown?

a) Kinship
b) Ethnic groups
c) Historical factors
d) Ease and speed of access
