# Supplementary material for: Plant breeding can be made more efficient by having fewer, better crosses
Source: BMC Plant Biol. 2013 Feb 7;13:22. doi: 10.1186/1471-2229-13-22 (PMC3621587; doi:10.1186/1471-2229-13-22)
Supplement: Additional file 2 — A proposal for the release of an aromatic rice variety Suanulo Sugandha. Application to be made for approval and release of crop varieties to Government of Nepal. Ministry of Agriculture and Cooperatives, National Seed Board; 2008. [file 1471-2229-13-22-S2.pdf]

New Upland Rice Varieties for India

Rainfed Agriculture Impact Study No. 1

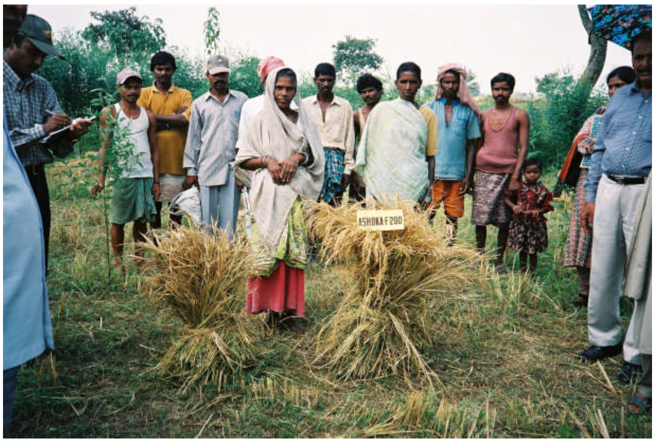

Monitoring Impact Assessment and Learning Component (MIL) 2.2 of the Research into Use Programme (RiU)

Contents

Acknowledgements ... i
Abbreviations ... ii
PART A SYNTHESIS OF STUDY FINDINGS ... 1
INTRODUCTION ... 1
GENERAL OVERVIEW OF THE METHODOLOGY ... 1
CASE STUDY FINDINGS ... 2
INSIGHTS ... 7
The innovation process ... 7
Seed dissemination and extension ... 7
The nature of the benefits ... 8
ANNEX A1 The Study Methodology ... 9
PART B REPORT ON THE STRUCTURED SURVEY ... 1
EXECUTIVE SUMMARY ... 1
INTRODUCTION ... 4
METHODS ... 9
Selection of states and districts ... 9
Selection of villages ... 10
Selection of households for interview ... 11
Group discussions ... 11
Household survey ... 12
Selection of households ... 12
Poverty index ... 13
Administration of questionnaires ... 14
RESULTS AND DISCUSSION ... 15
Seed spread ... 15
Persistence of use ... 21
Extent of Use in the Survey Areas ... 23
Estimated Extent of Use Beyond the Survey Areas ... 28
What rice varieties do the Ashoka varieties replace? ... 34
Environmental impact ... 37
Benefits to Users ... 37
Characteristics of Users and non-Users ... 40
REFERENCES ... 42
ANNEXES ... 43
Annex B1 Group Discussion in primary villages ... 44
Annex B2 RFA Household Survey Questionnaire: Ashoka Rice Growers ... 46
Annex B3 RFA Household Survey Questionnaire: Non-Ashoka Rice Growers ... 53
Annex B4 Poverty index ... 58
PART C: REPORT ON THE QUALITATIVE STUDY ... 60
INTRODUCTION ... 60
METHODOLOGY ... 60
FINDINGS ... 63
Extent of innovation within survey villages ... 63
Who are the innovators? ... 64
Impact of innovations ... 65
What are factors influencing the growing of Ashoka? ... 65
INSIGHTS ... 67

ANNEXES - FIELD REPORTS by Dr. M. BASU and Dr A. DEY 69

Acknowledgements

In addition to the authors of the different parts of this case study, a large number of staff members of Gramin Vikas Trust were involved in conducting the fieldwork for the structured survey. The fieldwork was coordinated by Dr JP Yadavendra of GVT. In eastern India the survey team was led by Dr. SC Prasad, and western India by Shri HK Tomar. Senior managers of GVT, Shri Amar Prasad and Shri KS Sandhu, attended a 10-day survey planning workshop in June 2008 and gave their general support to the study. We are extremely grateful to them all.

The fieldwork for the qualitative study was carried out under the supervision of the Study Team's social development advisor, Dr Marlene Buchy. The Indian researchers hired for this study were Dr Malika Basu and Dr Aniruddha Dey. Dr Dey was assisted by Mr Albert Xalxo, and logistical support (locating villages and introducing researchers) was provided by GVT local staff.

We are grateful to Dr Sheelagh O'Reilly of IOD for her continual support, advice and encouragement throughout the study.

Abbreviations

AAU	Anand Agricultural University (Gujarat)
AICRP	All India Coordinated Research Project
BAU	Birsa Agricultural University
BVD	Birsa Vikas Dhan (release suffixes of upland rice varieties
CAZS NR	CAZS-Natural Resources
CBO	Community-based organisation
COB	Client-oriented breeding
CRI	Central research institutes
CRURRS	Central Rainfed Upland Rice Research Station, Hazaribag
CRS	Catholic Relief Services
CSC	Central Sub-Committee *or* Central Seed Committee
CVRC	Central Variety Release Committee
DFID	Department for International Development
DoA	Department of Agriculture in a state
EIRFP	Eastern India Rainfed Farming Projects
FGD	Focus group discussion
GAU	Gujarat Agricultural University
GD	Group discussion
GOI	Government of India
GVT	Gramin Vikas Trust
HH	Household
ICAR	Indian Council for Agricultural Research
IRRI	International Rice Research Institute
JNKVV	Jawaharlal Nehru Krishi Vishwa Vidyalaya (Madhya Pradesh)
KVK	Krishi Vigyan Kendra
MP	Madhya Pradesh
MPRLP	Madhya Pradesh Rural Livelihoods Project
MPUAT	Maharana Pratap University of Agriculture & Technology (Rajasthan)
MV	Modern variety
NGO	Non-governmental organisation
NREGA	National Rural Employment Guarantee Act
NSC	National Seed Corporation Limited
PPB	Participatory plant breeding
PSP	Plant Sciences Research Programme of DFID
PTD	Participatory technology development
PVS	Participatory varietal selection
RF	Rockefeller Foundation
RIUP	Research into Use Programme
RNRRS	Renewable Natural Resource Research Strategy of DFID
SAU	State Agricultural University
SHG	Self-help groups
SSCA	State Seed Certification Agency
SSSC	State Seed Sub-Committee
SVRC	State Variety Release Committee
WIRFP	Western India Rainfed Farming Project

PART A SYNTHESIS OF STUDY FINDINGS

Prepared by:

Czech Conroy
Natural Resources Institute
University of Greenwich, ME4 4TB
UK

INTRODUCTION

This case study is an impact assessment of two rice varieties, Ashoka 200F and Ashoka 228. These varieties were bred through client-oriented breeding in DFID Renewable Natural Resource Strategy (RNRRS) supported projects of the Plant Sciences Research Programme.

This study used a variety of structured and semi-structured methods. Part B of the case study describes the methodology and findings of fieldwork undertaken by the University of Bangor's CAZS-Natural Resources, in collaboration with the Indian NGO, Gramin Vikas Trust. Part C describes a subsequent semi-structured and more qualitative piece of fieldwork undertaken by Dr Marlene Buchy, a social development consultant, and two Indian consultants, Dr Malika Basu and Dr Aniruddha Dey. This part of the case study (part A):

- describes the overall methodology used in this case study;
- synthesizes the key findings from the two field studies, identifying both similarities and differences; and also
- draws lessons/insights from the findings relating to technology development and innovation processes.

This is one of seven case studies on rainfed agriculture innovations in south Asia, which are part of a broader 'cluster study'. Each case study, and the broader cluster study, aims to obtain information regarding:

1. *extent* of use of the innovation
2. *who the innovators are* and are not
3. *impact* (including benefits and disbenefits) of the innovation
4. factors *explaining extent* of innovation (factors influencing use)
5. factors *explaining differential impact* among potential users/innovators
6. *Sustainability* of use of innovations by farmers.

GENERAL OVERVIEW OF THE METHODOLOGY

The impact assessment was done in five districts located in four states of India, Banswara, Rajasthan; Mayurbhanj, Orissa; West Midnapur, West Bengal; and, Ranchi and Hazaribag, Jharkhand. The study covered 20 'project' villages (also called 'primary' villages) where activities related to the Ashoka varieties had been implemented – 4 villages in each district; a larger number of 'secondary' non-project villages to which cultivation of the Ashoka varieties had spread from the primary villages; and a few 'tertiary' villages to which Ashoka cultivation had spread from the secondary villages.

All four states were covered: (a) in case differences in innovation systems between the states influenced the impact of the technology; and (b) to cover a wide geographical area in

case differences in agro-ecological conditions influence impact. Two districts were selected in Jharkhand state because that is where most of the Ashoka work had been done. The survey methods are described below.

The *structured survey* comprised three methods (see Annex A1 for further details). First, village-level group discussions to determine the varietal composition of rice among upland and/or medium land rice growers, and the extent to which the Ashoka varieties were being grown. Second, group discussions in primary villages to identify the main secondary villages to which primary village rice growers had supplied Ashoka seed. Third, a questionnaire-based household survey of current upland/medium land rice growers in the primary villages, comprising 10 current Ashoka users and five non-users per village.

In the *qualitative (semi-structured) study* all the 'primary' villages surveyed during the structured work, as well as a selection of 'secondary' villages were visited. Three methods were used in this study:

- group discussions, including farmers growing Ashoka varieties, those who might have grown them and stopped and those who might want to grow it in the future;
- in depth interviews of 'interesting' individual households if any, such as households having experienced dramatic changes in their livelihoods or farming systems or households that have tested the seeds for a few years and decided to stop;
- cross-checking some data with other actors such as: primary health care nurse or health visitor, trader, extension worker, nearby research station.

CASE STUDY FINDINGS

This section synthesizes the findings from the structured survey (found in Part B) and the semi-structured, qualitative survey (Part C). By and large the two sets of findings are consistent and complementary: where discrepancies occur they have been noted.

1. Extent of Use

Within a household's rice-growing area The household survey found that farmers who grew the varieties adopted them on a high proportion of their suitable land (on average 75% of their upland and over 50% of their medium land). In contrast, the qualitative survey authors concluded that: " ..though the number of farmers who grow Ashoka has increased, the proportion of the potentially suitable land allocated by households to growing Ashoka remains small. In Kud (Hazaribagh district) for example only 25% of the suitable land is under Ashoka and in Benjara (Ranchi district) it is 20%". This study found that in all the FGDs across the 5 districts, farmers maintained that they will continue to grow their local *Desi* rice varieties. Farmers want to spread the risks by maintaining a variety diversity but also *Desi* rice still has some qualities valued by local people such as for example, those presented in Table C (in Annex C11) in the case of Benjara village.

Within and between survey villages In the primary and secondary villages, 14 to 53% of households grew the Ashoka varieties on 2 to 24% of the total rice area, depending on the district and village. In the survey of individual farmers, over one third of those who grew an Ashoka variety distributed seed to other households from the 2007 harvest. In the structured group discussions farmers reported that, on average, farmers in every study village (i.e. primary village) had distributed seed to about two new villages (secondary villages). This process extended further as farmers had distributed seed to new (tertiary) villages from

these secondary villages. The primary and secondary villages had more or less equivalent levels of use despite there having been no formal seed supply in the latter case.

Within and beyond survey districts Given that there was high village-to-village spread and a large number of villages had been supplied with seed by NGOs and other organisations, adoption was extrapolated from the sample to the district and state levels. It was estimated that about 177,000 farmers were growing about 26,600 ha of Ashoka varieties in the five study districts.

If farmers adopt these varieties to a similar extent across the four states that were studied then about 420,000 ha would be devoted to them by the 2,800,000 households. Moreover, the varieties have already been widely distributed in the states of Madhya Pradesh, Gujarat, Chhattisgarh, and Uttar Pradesh, and they will have spread across borders to neighbouring states with important upland rice growing areas such as Bihar.

2. Factors explaining extent of innovation

Several factors influencing the extent of use of the varieties were identified. These can be grouped into two categories: (a) those preventing or discouraging use, and (b) those leading farmers to choose other crop-related options. The most important of the former is difficulty in accessing seed. The latter include: agro-ecological conditions for which other rice varieties are more appropriate; and crop options that give better returns. Each of these will now be discussed.

Access to seed Ashoka seed spread in the survey villages was almost entirely farmer to farmer and there was no commercial marketing of the seed. Farmers with Ashoka seed apparently gave it primarily to relatives, rather than to neighbours or friends. Thus, in the short-term at least, these factors may have prevented some farmers in survey blocks from obtaining seed.

Agro-ecological conditions The household survey found that in one district, West Midnapur, Ashoka varieties were not grown in the **medium lands**. This appears to be because farmers had better alternative rice varieties for this situation. In the **uplands** of this district Ashoka was widely used, but the fact that other modern varieties (MVs) were also widely grown here may have discouraged even wider use. The growth of other MVs was apparently due to the fact that it is possible to grow longer-duration, medium-land varieties in this situation as the duration of the monsoon is longer. There are five months of significant rainfall here compared with 3 to 4 months in Jharkhand and other more westerly districts.

Alternative crop options In the study districts of Rajasthan and Orissa, where the structured group discussions had revealed that there was no upland rice, the Ashoka varieties would only be grown if they replaced other crops. These were reported to be: predominantly maize in Rajasthan; mainly Sabai grass, *Ischaemum augustifolium*, grown for paper making, in Orissa; and, in Hazaribag, a wide variety of crops with Niger, *Guizotia abyssinica*, the most important. The qualitative study found that in Jaria village (Ranchi district) an NGO has been supporting different livelihoods activities, such as flower growing, which provide further opportunities; vegetable farming has been developed on upland plots in the kharif season as well. In Hutkona (Hazaribagh district) people reported stopping growing Ashoka rice because growing vegetables upland was more profitable and provided a longer stream of income. In Baripada – also found in the structured survey - many farmers use their upland to grow sabai grass for rope making, which secures a good market price, and Ashoka is not competitive.

3. Sustainability - continuity of Ashoka use

The household survey found that the Ashoka varieties were still being grown by the great majority of farmers (95%) who were first given seed 4 to 6 years earlier. This was despite little, or no, subsequent outside interventions for these varieties in their villages. Similarly, farmers that obtained seed from other farmers continued to grow the varieties once they had gained access to the seed.

The household survey found that the great majority of farmers who discontinued their use of Ashoka rice said it was because of lack of access to seed. This constraint was confirmed by the qualitative study. For example, in Bahja and Huktona villages (Hazaribagh district), farmers reported losing all their seeds after the drought in 2006. Not all farmers kept seeds for the following year, consuming what they had; and when they ran out there was no alternative seed supply.

The qualitative survey identified many cases of discontinuers and several other factors as being responsible. These included *Bad early experience due to inappropriate practice,* which led some farmers to stop growing Ashoka rice. In *primary villages* this happened because technical advice from GVT on the cultivation of Ashoka was limited: furthermore, some of the GVT staff employed for this work were non-agriculturists, hence they may not have always give appropriate advice. Farmers in *secondary villages* sometimes planted the seeds on the wrong type of land or followed inappropriate practices, leading to failure of the crop.

Other reasons given by farmers for ceasing to grow Ashoka varieties, included:

- damage by elephants, which prefer Ashoka varieties to all others
- prone to flies (*Gandhi bug*), one type of grain borer which destroys the crops.

According to farmers in Hulsi, Ranchi district, possible reasons why elephants prefer Ashoka varieties and why Ashoka fields tend to be the first point of attack by the elephants were:

a) as the plants are tall it was easier to remove with their trunks
b) softness and taste of the fodder and grains
c) sweet smell from the matured plants.

4. Who are the innovators?

There was no significant difference in the poverty status of users and non users of the Ashoka varieties when they were compared using the poverty index developed for the study. Of the 200 respondents in the household survey, 198 (99%) fell in the poor[1] class (defined as having a poverty score of less than 12.5): the highest score of any household was 14. Using other poverty indicators, i.e. BPL status and NREGA card holder, slightly higher percentages of users than non-users would be classified as poor (see Table A?): however, as noted earlier, these indicators are not entirely reliable. Regarding ethnicity, a lower proportion of users belonged to scheduled tribes (see Table), and a higher proportion belonged to ‘other backward castes’.

Overall, the findings suggest that the varieties are very well suited to the needs of resource-poor farmers. The qualitative survey also identified some Ashoka users who would probably have been classified as better off if they had been scored against the poverty index.

[1] This was not surprising given that the villages to which the seed was distributed were all selected by GVT (in the DFID-supported EIRFP and WIRFP bilateral projects) on the basis of being very poor.

Table A1 Characteristics of users of Ashoka varieties and non-users

Characteristic	Users	Non-Users
Poverty index	6.54	6.33
Below poverty line (%)	68	54
NREGA card holder (%)	82	76
Tribal (%)	63	74

5. Differential impact

Neither poverty/wealth status nor ethnicity have a differential effect on a household's capacity to grow the Ashoka varieties. The extent to which a household can benefit from them seems to depend primarily on the area of suitable land that it has for cultivating them; and also perhaps on the prevailing agro-ecological conditions, which may affect the yield.

6. Benefits and Impact of Ashoka Rice Varieties

Benefits

The household survey found that better quality grain was the benefit ranked highest by most farmers: there was a consistent high ranking of grain quality in all districts (Part B,Table 33). This was followed by earlier harvest and then better quality fodder, both of which were frequently mentioned in the qualitative survey. In addition, about half of the farmers identified 'more rice' (i.e. higher yield) as a benefit.

The vast majority of Ashoka grain was kept for home consumption, apparently due to:

1. the perceived high quality of Ashoka grain; and
2. because the early harvesting of the Ashoka grain takes place at a time when households are short of food grains generally.

The first point was mentioned in many FGDs in the qualitative survey, with repeated references to the tastiness of Ashoka rice. According to a FGD with women growers in Bhuyan Tola: "Even if they don't have any vegetables or pulses only rice can be consumed with a little salt. It is so tasty that even rice with soup can be taken without any side dish".

The second point was also reported in various villages covered by the qualitative survey, for example: Ashoka can be harvested "during the usual lean season… During *Dusherra (the main festival of the Hindus),* now at least they have some food to eat" (Kud village, Hazaribagh); the fact that it is an early maturing variety "ensure[s] food security during lean season" (Benjara, Ranchi).

In the household survey 83% of Ashoka users reported increases in rice availability, with a mean increase in rice self-sufficiency of almost one month, or 17%. The findings of the qualitative survey were similar:

> "The direct positive result of the introduction of Ashoka is an increase in grain production which extends the grain self-sufficiency of the household by 2 weeks to 1 month in Banswara district, for example ; and for up to 2 months even for marginalized farmers in Hazaribagh district, depending on the size of the land available".

The higher food availability reported by the majority of the farmers was reflected in the higher reported grain yield in a typical growing year of the Ashoka varieties compared with

popular local ones (Part B,Table 32). In the questionnaire-based household survey farmers were only able to make this comparison for a *typical* growing year. The yield advantage would probably be greater in a drought year, due to the shorter growing period of the Ashoka varieties.

Respondents in the household survey were not able to make the comparison for a *drought* year, as the last one was 2002 and they had not experienced a drought year since they started growing Ashoka varieties. However, in the qualitative survey villages in Hazaribagh farmers *were* able to make this comparison. There was less rainfall here in 2006 and a drought was declared in the area. In Kherika village all crops were affected, and only those farmers who grew Ashoka got some paddy, which at least could be used as seed for the next season: but for other rice varieties there was no production. This village also experienced lower rainfall than usual in 2008: farmers reported that Ashoka's production was not affected, whereas production was less than usual for other varieties. In Hutkona village of Hazaribagh a drought was reported as having occurred in 2004, but in this case even the Ashoka crop failed.

The qualitative survey identified the short duration and the resistance to drought of the Ashoka varieties as two positive factors mentioned by all groups in the 5 districts. This survey also identified some other commonly perceived benefits of Ashoka varieties that were not picked up in the household survey, including:

- Earlier maturity enables growing of a second crop
- Requires less fertiliser
- Requires less water
- Requires less labour
- Easier to cook and needs less fuel.

Impacts

The increase in yield, and hence grain self-sufficiency, has resulted in a decrease in grain related expenditure: this frees up funds for other uses. For example, one woman (in Kud village, Hazaribagh district) reported that this had enabled her to continue sending her children to school.

One positive impact women mentioned is that, as Ashoka matures earlier than other varieties, the period of harvesting and threshing rice is less intensive as it is more spread out. Women said that they felt less under pressure. Women in one village (Bhuyan Tola, Katkamsandi block) also reported that there had been an increase of agriculture wage labour by 15-20 days due to the longer duration of the rice harvest.

Improved nutritional status was also reported in some villages. However, it would be difficult to establish whether any such improvement was due to growing Ashoka rice or to other factors.

7. Grassroots innovation processes

Farmer to farmer spread This has been extensive and some details were given earlier in section 1.

Gender dimension The qualitative study found that both men and women have been involved in innovating Ashoka varieties in their households and villages. In a number of the

primary villages the seeds were distributed by GVT through women's self-help groups. In each of two *secondary* villages in Banswara district, the seeds were initially introduced through a woman. The two women both initially got seeds from relatives and after their first harvest distributed some seeds through their SHG. Another woman (in Kud, Hazaribagh district) convinced her in-laws of the benefits of Ashoka and they had been growing it since 2003; she said this success had improved her status within the family.

Growing Ashoka as a summer crop Farmers sometimes modify technological 'inventions' and come up with a different innovation, and this is one example. The qualitative survey brought to light the fact that some farmers were also growing Ashoka rice as a summer crop, planting it in February. The structured household survey also found cases of summer cultivation of Ashoka rice in the survey districts in Orissa and W. Bengal. Whereas the two varieties were developed by the plant breeders purely as a *kharif* crop.

INSIGHTS

The innovation process

The development and dissemination of the Ashoka varieties was made possible primarily by the contributions (financial and in-kind) made by DFID bilateral development projects; and subsequently by supplementary funding from PSP programme development (PD) funds. Participatory rice breeding in India began (in 1998) under the auspices of the EIRFP and WIRFP, with CAZS-NR researchers (Dr Virk and Professor Witcombe) working as consultants to the projects. The development of the varieties and their distribution to the 'primary' villages surveyed in this study was done during the period up to and including 2003; but it was only in 1999 that a related and fully funded PSP research project started, combining participatory breeding with the use of molecular markers.

The level of impact shown in this case study could not have been achieved with the level of funds (e.g. £150,000-200,000) typically provided for a RNRRS research project; nor in the period of time for which RNRRS funding was typically provided, i.e. three years, with possible extensions of 1-3 years.

The involvement of Dr Virk and Professor Witcombe as consultants to the projects stemmed from the fact that the University of Bangor was part of consortia to which DFID awarded the contract to manage consultancy inputs to the WIRFP from its inception in 1993 to 2005.

The PD funds were available to the CAZS-NR researchers because Professor John Witcombe was the manager of the PSP and therefore had considerable discretion over what these funds were used for.

Seed dissemination and extension

New varieties of an existing crop are probably the easiest new technology for farmers to start using, and there are many cases of new varieties spreading from farmer to farmers without any official extension campaign, and sometimes without them having been officially released. This case study has provided another example, documenting quite extensive and rapid farmer-to-farmer spread of Ashoka varieties.

Nevertheless, the fact that some farmers grew Ashoka varieties on inappropriate lands and had problems growing Ashoka rice points to weaknesses in the way in which seeds of the Ashoka varieties were disseminated - or conversely ways in which it could have been strengthened. GVT staff did not always give proper instructions to farmers while giving the seed for testing in the early (PVS) stages. This is partly/largely because the GVT was mostly targeting social issues in the EIRFP and WIRFP and most of its community workers (who distributed the seed to the farmers) had a social science background. There was a lack of literature with the Ashoka variety seed in the latter stages when dissemination started.

Several farmers or groups of farmers interviewed in the qualitative survey, including ones positive about Ashoka, argued that Ashoka use could have been more widespread and/or faster if seed distribution had been accompanied by, or followed up with, an extension campaign. One example is Litu Oraon (30), a very dynamic and highly respected individual in Hulsi village, Ranchi, through whom Ashoka seed was first introduced in the village in 2002: he was still growing Ashoka rice when interviewed in March 2009. He believed the villagers should have been given proper orientation on the process of growing Ashoka and said that in absence of it many farmers incurred a loss in the first year. He also felt that some follow up from GVT would have helped the farmers to gain confidence and the spread of Ashoka varieties in the village. Even after growing it for many years he felt there was scope for improvement with guidance; and even in 2009 he was not very comfortable in saying how much seed was required for 1 acre of land. According to him had there been some hand outs or pamphlets mentioning the selection of land, amount of seed required for a specific unit, methods to be followed, requirements of fertilisers and pesticides, sowing and harvest time, etc. the situation would have been much better and more people would have been benefitted from the variety.

Similar points were made by others, such as a group of farmers in Bajha village, Katkhamsandi, who suggested that "local farmers needed more counseling and advocacy and then only they would have shifted from hybrid varieties to Ashoka … There should be more campaign on benefits of Ashoka". The group pointed out that, in contrast, "There are local agents for hybrid varieties and even the dealers campaign for hybrid varieties".

The nature of the benefits

Conventional plant breeding tends to attach greater weight to increased yield than to any other crop trait. It is interesting, therefore, that, although Ashoka has this trait, the majority of Ashoka growers ranked other traits, particularly grain quality and taste, more highly… It is also interesting that Ashoka users identified many other positive characteristics of these varieties. This reinforces the case made repeatedly by advocates of PPB and COB that plant breeding should take account of multiple farmer-preferred crop traits.

ANNEX A1 The Study Methodology

What kind of comparison? – 'before and after' versus 'with and without'

Assessing the impact of any particular development intervention – be it a technology, policy or institutional change – is seldom straightforward. The context in which the intervention takes place is real life – dynamic, complex, uncontrolled and often spatially heterogeneous – and this makes it difficult to separate the effects (if any) of the intervention from any other changes in people's lives that may have been taking place. There may be other contextual changes in rural study areas - such as electrification, development and roads and schools - that could have contributed to any general positive changes in local livelihoods that have taken place since the intervention was implemented or initiated.

In controlled experiments scientific researchers often make *'with and without'* comparisons involving two or more very similar groups of people, livestock, crops or whatever is being studied and is expected to be affected by the 'intervention' or 'treatment'. Any differences that develop between the groups during the period of the experiment can then be plausibly attributed to the 'intervention'.

Use of a *'with and without'* approach was considered in this impact assessment, and would have involved selecting 'project' villages to which seed of Ashoka varieties had been distributed and very similar non-project villages. This approach was not adopted for two reasons. First, it would have been difficult to ensure that any two villages involved in a comparison were sufficiently similar, and hence be confident that any differences subsequently identified between rice-growing farmers in the two villages were due to the Ashoka varieties. Second, the primary or project villages where the seed was distributed in 2002/2003 were ones that had previously been purposively selected by development projects as among the poorest in their block or district – hence other villages in the same block would probably not have been as poor.

For the above reasons the 'before and after' approach was considered to be more appropriate and was used. This approach has its own problems – for example, if we had been measuring impact in terms of changes in household income since Ashoka varieties had been 'adopted' by farmers then any increase in income could have been due to many other factors. Thus, the study team used a kind of 'results chain' approach to minimise this kind of problem. The before and after comparisons were based on farmer recall and focused on changes in variables like rice yield and self-sufficiency in rice (in months per year). The team examined the linkages between area of land planted to Ashoka rice, the yield obtained, the ways in which the rice was used and any benefits that farmers said they had experienced as a result of growing Ashoka rice.

Minimising bias

The senior staff of RIUP's MIL component decided that the structured survey would be implemented by staff of the organisations that had been involved in developing and distributing the Ashoka varieties, as they were familiar with the technology and with the locations where the seed had been distributed and the villagers living there. An independent Cluster Study Team Manager (Czech Conroy) was appointed to oversee the design and implementation of this case study and several others. He had the final say on survey methods, wording of questionnaires etc., and also carried out the (stratified) random selection of study districts and villages. In the structured survey household selection was also random (after stratification), but for practical reasons the selection was done by the field teams implementing the survey.

In the questionnaire used in the structured household survey almost all of the questions were closed (e.g. Yes/No) rather than open in order to avoid bias (sub-conscious as well as conscious) in the way that answers were recorded. The qualitative study, on the other hand, would be inherently semi-structured or unstructured, which would have increased the likelihood of bias among survey team members creeping in. For this reason, and also because of the special skills required to undertake qualitative work of this nature, it was decided that this study would be done by qualified independent researchers, under the supervision of the Study Team's social development advisor, Dr Marlene Buchy. The Indian researchers hired for this study were Dr Malika Basu and Dr Aniruddha Dey.

The use of two different sets of study methods by two different teams was also seen as advantageous in terms of triangulation – cross-checking – of survey findings.

Details of methods used in the structured survey

These are summarised in the table below.

Method 1 A Group Discussion to generate information about users and non-users of the Ashoka varieties, and information about the percentage of users and an approximate idea of the percentage of the land area on which the innovation was being practised[2]. The GD aimed to generate lists of users and non-users. Where possible the survey team would obtain Patwari records and use these to triangulate the GD lists (or consider alternative approaches to triangulation, such as facilitating villagers to prepare a social map showing all households), to avoid any households being omitted from the GD list. The concern here was that there might have been a possibility of systematic bias towards any omissions being primarily scheduled caste or other marginalised households.

Method 2 The purpose of this method was to find out how many 'secondary' villages had the new varieties and how distant they were from the primary ('project') village.

Method 3 In each project village 10 current users of Ashoka varieties were interviewed, of whom 5 were original (2002 or 2003) targeted receivers (TRs) of Ashoka seed and 5 non–TRs (i.e. farmers who did not receive seed directly from GVT). Thus, there were 200 respondents who were current growers or users of Ashoka varieties, 100 of whom would be TRs. The number of current non-users of Ashoka varieties was 5 per village, giving 100 altogether.

---

2 (Method 3 also generated information on extent of adoption per grower household (EXTENT by household rice area); and by multiplying the average % adoption of rice land per HH by total number of adopters from Method 1, it would be possible to triangulate the information from Method 1 about the total area of adoption per village.)

Table Summary of Structured Survey Methods for Ashoka Rice Varieties

Survey Method	To measure	Where?	What villages?
Method 1. Group discussion<br>Among upland and/or medium land rice growers: Ashoka and non-Ashoka rice growers	EXTENT<br>Adoption level within village<br>(i) varietal composition of rice among upland and/or medium rice growers,<br>(ii) adoption of Ashokas by area (%) and HH (%); and<br>(iii) numbers of growers and non-growers (& caste/ethnicity)	Primary villages in:<br>1. GVT West – Banswara (4).<br>2. GVT East – Bero (4), Katkamsadi (4), Jhargram (4). Baripada (4)	20 villages (primary) that received seed in 2002 or 2003
Method 2. Inter-village seed spread mapping<br>**Method 2a**. Group discussions (GD) in Primary villages to identify secondary villages (done at same time as method 1).<br><br>**Method 2b**. GD in Secondary villages.	EXTENT (Spread)<br>**Method 2a** Main nearby or connected villages in which Ashoka is grown, and linkages between those (secondary) villages and the primary village.<br><br>**Method 2b**. Extent within selected secondary villages measuring same as Method 1 parts (i) and (ii) above	Method 2a: Same as Method 1.<br><br>Method 2b:<br>Likely to be in the same blocks as primary villages but could be across block borders.	Method 2a in the same 20 Primary villages above.<br><br>Method 2b in connected or nearby secondary villages identified in method 2a
Method 3. Structured HH surveys of current upland/medium land rice growers<br><br>**3 (a) Short Q'aire of:**<br>100 current non-users of Ashoka<br>(may include discontinuers)<br><br>**3 (b) Long Q'aire of:**<br>200 current Ashoka users	Characteristics of HH and farming/cropping system, and **comparison of non-users (Method 3a) and users (3b)** to enable analysis of factors influencing:<br>1. ADOPTION (or not) &<br>2. SUSTAINABILITY/ continued use (or not)<br><br>**Short Q'aire**<br>Factors influencing ADOPTION or not<br><br>**Long Q'aire:**<br>* Factors influencing ADOPTION or not<br>* EXTENT of Ashoka adoption by HH<br>* Benefit/HH<br>* IMPACT on livelihoods	**Regions** same as 1.<br>**Households** Sampling of 300 with **15 per primary village** stratified into 3 groups of 5 hh each<br>1. **Short Q'aire:** Random sample of 5 non-users per village<br>2. **Long Q'aire:** Random sample of 5 non-target users<br>3. **Long Q'aire:** Random sample of 5 targeted users	Same as 1 (a)
Method 4. Qualitative in-depth survey of:<br>(a) individual (♀ & ♂) receivers/users<br>(b)individual non-users<br>(c) focus groups	IMPACT<br>Including related emerging issues, e.g. migration patterns; village labour markets; degree of indebtedness; how they use extra income.	In each of the four states	- Sample of seed recipient villages covered by Methods 1 & 3<br>- Maybe other villages too

PART B REPORT ON THE STRUCTURED SURVEY

Prepared by:

John R Witcombe and D.S. Virk
CAZS-Natural Resources,
Bangor University LL57 2UW,
UK

Acknowledgements

A large number of staff members of Gramin Vikas Trust were involved in conducting the fieldwork for the structured survey. The fieldwork was coordinated by Dr JP Yadavendra. In eastern India the survey team was led by Dr. SC Prasad, and western India by Shri HK Tomar. Senior managers of GVT, Shri Amar Prasad and Shri KS Sandhu, attended a 10-day survey planning workshop in June 2008 and gave their general support to the study. We are extremely grateful to them all.

EXECUTIVE SUMMARY

- This report describes an assessment made on the impact of two rice varieties, Ashoka 200F and Ashoka 228. These varieties were bred through client-oriented breeding in DFID Renewable Natural Resource Strategy (RNRRS) supported projects of the Plant Sciences Research Programme. The history of the breeding of these varieties in an institutional context is described.
- The impact assessment was done in five districts located in four states of India, Banswara, Rajasthan; Mayurbhanj, Orissa; W Midnapur, West Bengal; and, Ranchi and Hazaribag, Jharkhand. In twenty villages that received seed of these varieties (primary villages) group discussions were held and a total of nearly 300 farmers were interviewed who were randomly selected from users and non users of the varieties. Group discussions were held in 37 villages to which seed had spread from them (secondary or tertiary villages).

Benefits

- When specifically asked about benefits about half of the 200 farmers who grew an Ashoka rice variety reported increases in rice grain self sufficiency (by 17% or one month) or increases in grain sales (by 46% or 150 kg).
- A much higher proportion reported increases in grain yield; most farmers reported from recall yield advantages over local alternatives, although average increases were lower than those recorded in actual participatory, on-farm experiments.
- Farmers reported other benefits such as better eating quality and higher straw yield and earlier harvests.

Acceptability and adaptation to specific rice ecosystems

- In 2007, the Ashoka varieties were still grown by the great majority of farmers (95%) who were first given seed 4 to 6 years earlier despite little, or no, subsequent outside interventions for these varieties in their villages. Similarly, farmers that obtained seed from other farmers continued to grow the varieties once they had gained access to the seed. The great majority of farmers who discontinued their adoption said it was because of lack of access to seed.
- Farmers who grew the varieties adopted them on a high proportion of their suitable land (on average 75% of their upland and over 50% of their medium land).

- Adoption in upland and medium land differed greatly from district to district as in Rajasthan and Orissa little or no rice was grown in the uplands. Hence, in these two districts they were cultivated only in medium lands, but almost only in uplands in West Bengal and mostly in the uplands in Ranchi district, Jharkhand. Only in Hazaribag, Jharkhand, were they grown in both upland and medium land.
- What varieties the Ashoka varieties replaced varied greatly by district and was influenced by the agro-ecological situation and by the extent of private-sector marketing of hybrid rice.
    - The Ashoka varieties were the only modern upland varieties with significant adoption in Hazaribag district, Jharkhand and Rajasthan so they replaced landraces.
    - In higher rainfall uplands of West Bengal and medium lands in Orissa, they partially replaced modern varieties that were of longer-duration.
    - In Ranchi district, Jharkhand, the only varieties in the uplands were private-sector hybrids and public-sector medium land varieties and over 90% of the area under them was replaced by the Ashoka varieties.
- Only in Hazaribag district were public-sector upland varieties found to any significant extent and this was the district where the Central Upland Rice Research Station was located. Vandana was used on less than 5% of the area 16 years after its release compared with 40% of the area for the much more recently released Ashoka varieties. In W Midnapur Kalinga III was grown (16%) and could have led to the underestimation of the area of Ashoka varieties as it is easily confused with the Ashoka varieties. Kalinga III was introduced by GVT in nineties but no seed is now available in the market and farmers growing Ashoka varieties could be still calling it Kalinga III.

Seed distribution by farmers

- In the survey of individual farmers, over one third of those who grew an Ashoka variety distributed seed to other households from the 2007 harvest.
- In the group discussions farmers reported that, on average, farmers in every study village (i.e. primary village) had distributed seed to about two new villages (secondary villages). This process extended further as farmers had distributed seed to new (tertiary) villages from these secondary villages.
- The primary and secondary villages had more or less equivalent levels of use despite there having been no formal seed supply in the latter case. In Hazaribag district of Jharkhand, adoption in tertiary villages was examined and was found to be higher than in the primary villages.

Extent of use

- In the primary and secondary villages, 14 to 53% of households grew the Ashoka varieties on 2 to 24% of the total rice area, depending on the district and village. Given that there was high village-to-village spread and a large number of villages had been supplied with seed by NGOs and other organisations, adoption was extrapolated from the sample to the district and state levels. It was estimated that about 177,000 farmers were growing about 26,600 ha of Ashoka varieties in the five study districts.
- If farmers adopt these varieties to a similar extent across the four states that were studied then about 420,000 ha would be devoted to them by the 2,800,000 households. Moreover, the varieties have already been widely distributed in the states of Madhya Pradesh, Gujarat, Chhattisgarh, and Uttar Pradesh, and they will have spread across borders to neighbouring states with important upland rice growing areas such as Bihar.

Poverty focus

- There were no significant differences in how poor the users and non users of the Ashoka varieties were when compared using a poverty index (Table 6). Over 99% of the households were poor which confirms the strategy of the EIRFP and WIRFP of

targeting villages having a high proportion of indigenous tribal people and with poor access to markets and other facilities. There were three socio-economic traits with small but significant differences between the users and non-users of the Ashoka varieties. The households that used the varieties had a higher number of farm workers per household, a higher proportion of heads holding a below the poverty line card, and a lower proportion of scheduled tribes. These differences were too small to suggest that social factors are a major factor in the upland rice innovation system.

INTRODUCTION

This report examines the uptake and impact of Ashoka 200F and Ashoka 228 varieties of upland rice that were developed and disseminated in the DFID Plant Sciences Research Programme RNRRS projects in India. These two varieties were produced using client-oriented breeding (COB) methods[3] that included participatory varietal selection (PVS). This was carried out jointly by Gramin Vikas Trust (GVT) and Birsa Agricultural University (BAU), Ranchi and CAZS-NR.

Ashoka 200F was released as Birsa Vikas Dhan 109 and Ashoka 228 was released as Birsa Vikas Dhan 110)[2].

Before the initiation of the RNRRS project to breed the Ashoka varieties PVS had been used to identify parental varieties; Kalinga III in eastern and western India and IR 64 in eastern India.

During the development of the Ashoka varieties institutional arrangements varied greatly and many different partners were involved (Tables 1 and 2).

Plant breeding is a long-term process and the rice breeding programme was funded in a number of consecutive projects involving multiple partners (Tables 1, 2).

Client-oriented breeding can build on the results of PVS as it can identify suitable parental varieties for crossing. PVS in upland rice was carried out in eastern and western India by GVT in DFID-funded bilateral projects (EIRFP and WIRFP) with CAZS-NR as DFID consultants from 1994. These participatory trials identified Kalinga III to be the most farmer-preferred variety in the marginal rainfed uplands as it had a higher grain quality than all other varieties released for the uplands. Subsequently Kalinga III was disseminated by GVT in the WIRFP and EIRFP. Despite its popularity with farmers, Kalinga III has weak stems that make it prone to lodging under heavy rains and high fertility. Its early maturation allows it to escape terminal drought but its inherent drought tolerance is low because of its poorly developed root system. Therefore, a breeding programme was started to improve Kalinga III and to develop earlier maturing, high yielding, good grain quality variety with high adaptation to drought and low fertility soils of the rainfed uplands. COB methods were used in this breeding programme as they fitted both with the participatory development approach of the bilateral projects as well as the research objectives of the PSP. Participatory approaches were considered to be more efficient for rapidly delivering sustainable benefits to farmers.

---

[3] Participatory plant breeding (PPB) is still in common use but describes the activity of involving farmers rather than its purpose of improving client orientation. Hence, the term client-oriented breeding (COB) has been suggested as more appropriate and less divisive and is used here (Witcombe *et al.*, 2005).

[2] It is common to change the name of a variety from that under which it is tested at the time of release. The names of Ashoka varieties were changed by the Research Evaluation Council of BAU to reflect Birsa from BAU and Vikas from GVT. It should also be noted that only BAU could propose these varieties for release since NGOs are not allowed to do so for state releases in India. The GVT used both names during seed dissemination since farmers were more familiar with Ashoka names. We use 'Ashoka varieties' to refer to both Ashoka 200F and Ashoka 228 in this document.

Table 1. Timeline of institutional collaboration in breeding and dissemination of the Ashoka varieties by major projects.

Institutional start years	Project	Institutions
1996-05	GVT Western India Rainfed Farming Project (WIRFP) – identification of Kalinga III	GVT<br>CAZS-NR[3] as DFID consultants
	Eastern India rainfed farming project (EIRFP)– breeding of Ashoka varieties	GVT<br>CAZS-NR as DFID consultants<br>BAU
1998-01	Cross (Kalinga III/IR64) was made for the EIRFP and the Plant Sciences Research Programme (PSP) project R7122 'Participatory plant breeding in rice for high-potential production systems in the terai and low hills of Nepal'.	CAZS-NR<br>LI-BIRD
1999-02	Plant Sciences Research Programme project R7434 'Innovative methods of rice breeding – combining participatory plant breeding (PPB) with molecular markers'	CAZS-NR<br>GVT<br>BAU<br>LI-BIRD<br>UAS, Bangalore<br>MPUAT
2002-06	Plant Sciences Research Programme project R8099: Participatory plant breeding project in rice and maize for eastern India	GVT<br>CAZS-NR<br>BAU
2003-05	Western India rainfed farming project – testing and dissemination of the Ashoka varieties	GVT<br>CAZS-NR as DFID consultants<br>Three State Agricultural Universities, one each in Gujarat, Madhya Pradesh and Rajasthan
2005-09	Rockefeller Foundation funded project on Ashoka dissemination and upland rice breeding	GVT<br>CAZS-NR<br>BAU
2006-09	Madhya Pradesh Rural Livelihoods Project (MPRLP) – dissemination of Ashoka varieties	GVT and MP government

[3] CAZS changed its name during the course of this work to CAZS-NR. It is referred to throughout as its current name of CAZS-NR.

Table 2. Key events in the development, release and recommendation of the Ashoka varieties.

Year	Key event in breeding	Institutional details
1997	Cross of Kalinga III/IR64	Cross proposed by CAZS-NR and agreed in a training course run by CAZS-NR in Bangor for participants from EIRFP and WIRFP. Subsequently made at IRRI at the request of the Manager of Plant Sciences Research Programme, CAZS-NR, during a visit to IRRI for review of PSP rice projects. Rice breeding in India started in bilateral development projects (EIRFP, WIRFP) with State Agricultural Universities (BAU, Jharkhand, AAU, Gujarat, MPUAT, Rajasthan, and JNKVV, Madhya Pradesh) with support from CA|ZS-NR scientists initially funded from PSP programme development.
1998	First generation of selection in Ranchi, Jharkhand, India	In EIRFP with support from CAZS-NR scientists one as consultant to the EIRFP (DS Virk) and one funded under programme development from the PSP (JR Witcombe).
1999	Ashoka varieties tested in trials; PVS and research station trials	By GVT in the EIRFP and research trials by BAU with collaboration from CAZS-NR (reported in Virk et al., 2003).
1999	Entered in All India Rice Improvement Project trials of ICAR	Through BAU as only they were able to do so.
2001	First dissemination of Ashoka varieties	By GVT in EIRFP villages with CAZS-NR as consultants.
2003	Ashoka varieties released	By Jharkhand State Variety Release Committee for cultivation in Jharkhand state (Ashoka 200F as BVD 109 and Ashoka 228 as BVD 110)
2003	Entered in State Agricultural University trials in western India.	In Gujarat by AAU, in MP by JNKVV, and in Rajasthan by MPUAT all through the WIRFP with CAZS-NR as consultants.
2005	Ashoka 200F recommended for cultivation in Gujarat and Rajasthan .	In Gujarat by AAU and Rajasthan by MPUAT
2005	Scaling up in eastern India	Through Rockefeller Foundation (RF) funding for scaling up and research in Jharkhand, Orissa and West Bengal
2005	Notification of Ashoka varieties for certified seed production	By Ministry of Agriculture, Government of India in response to application by BAU
2006	Distribution of over 160 t seed	By GVT
2006	Both Ashoka 200F and Ashoka 228 recommended for cultivation in MP	By JNKVV
2006	Dissemination of Ashoka varieties in MP	Facilitated by GVT in in Madhya Pradesh Rural Livelihood Project
2007	First year over 200 t seed supplied	Under RF funding including seed produced in this project and sold to CRS for distribution in many states

A training course funded under the WIRFP and EIRFP was led by CAZS-NR in Bangor in the spring of 1997. GVT and BAU staff participated and widely accepted the suggested strategy of CAZS-NR of using a large population size breeding programme that used only a few crosses made with PVS-identified varieties as parents. Selection was delayed to the later generations in the target environment of farmers' fields or conditions similar to farmers' management (Table 3; Witcombe and Virk, 2001). To hasten the breeding process generations were grown in the main seasons and in the off-seasons and BAU were allowed, under the ICAR system, to pay for use of the necessary facilities for off season growing at

the Central Rice Research Institute, Cuttack. Farmers were involved in selection either in a collaborative or consultative mode in the segregating generations and immediately tested new varieties in PVS trials (Virk *et al.,* 2003; Table 3). The collaborative method produced Ashoka 200F and the consultative method Ashoka 228 (Table 3).

Seed of these two Ashoka varieties have been widely distributed to farmers in eastern and western states of India (Fig. 1). Apart from NGO seed distribution over 300 t was distributed to villages in Jharkhand by the Department of Agriculture from 2005 to 2007 and both varieties have been distributed to farmers in on farm trials in Tamil Nadu.

.

Table 3. Breeding of Ashoka 200F and Ashoka 228 from the cross Kalinga III/IR64

**Year/ season**	**Generation**	**Ashoka 200F (Collaborative COB)**	**Ashoka 228 (Consultative COB)**
1996-97	P1/P2, F_1 -F_2	Cross made and advanced to $_{F2}$	
RS[a] 97	F_2 –F_3	10300 plants raised at GVT-BAU farm Ranchi	
DS[b] 97-98	F_3-F_4	600 F_3 plant-progenies raised in the off-season generation advance at CRRI[c]	
RS 98	F_4-F_5	Equal quantities of F_4 seed from all of the 600 progenies harvested in the DS were bulked and 2 kg provided to four farmers .	177 selected F_4 lines, each derived from all of the plants in an F_3 line, were grown in 10 m^2 plots.
DS 98-99	F_5-F_6	Generation advance of farmer-selected bulk at CRRI.	F_5 lines from 12 most-selected F_4 lines grown at CRRI.
RS 99	F_6-F_7	Testing in AICRIP IVT-VE, and BAU and GVT station trials, and farmers' fields	
DS 99-00	F_7-F_8	Seed increase and roguing	
RS 00	F_8-F_9	Testing in BAU and GVT station trials and on-farm trials	
DS 00-01	F_9-F_{10}	Seed increase and roguing.	
RS 01	F_{10}-F_{11}	Testing in BAU and GVT station trials and on-farm trials	
RS 02		Testing in BAU and GVT station trials and on-farm trials	
RS 03		Released by State Variety Release Committee, Jharkhand on basis of trial data from 1999 to 2002	

[a]RS = Rainy season or *kharif* season.
[b]DS = dry season or *rabi* season.
[c]CRRI = Central Rice Research Institute, Cuttack, Orissa.

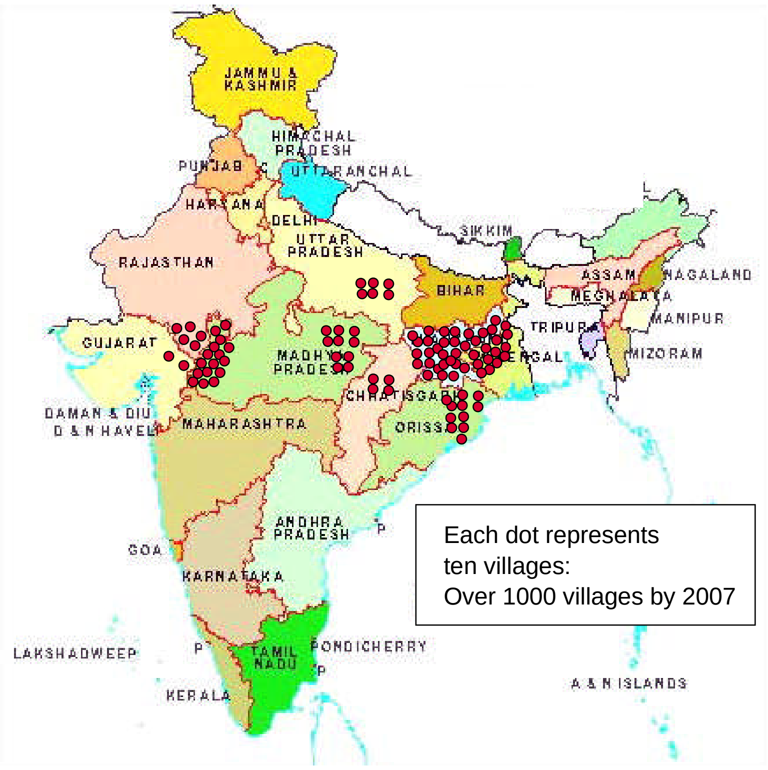

Fig. 1. The extent of documented distribution of the Ashoka varieties from 2001 to 2007 by non governmental organisations.

METHODS

In July to September/October 2008, field surveys were carried out by the Gramin Vikas Trist (GVT) in 20 primary villages (Table 4) with assistance from CAZS-NR and the Case Study Teams of MIL component 2.2. The surveys were conducted in villages selected from those where GVT had worked in either the Western or the Eastern Rainfed Farming Projects. The selection procedure is described below and can be summarised as:

- Districts were selected on the basis of the earliest seed distribution.
- Primary villages within districts were randomly selected from those where seed distribution had taken place in 2002 or 2003

Table 4. The secondary and tertiary villages sampled for group discussions and their relation to the primary villages.

			Secondary villages		Tertiary villages	
State District	State Block	Primary village	1	2	1	2
Rajasthan	Rajasthan					
Banswara	Bagidora	Bhojapra	Jeharpada	-		
	Kushalgarh	Bhuripada	Kundiya,	Suryan		
		Sundaripara	Haldupada,	Khanapada		
	Sajjangarh	Rohaniamanna	Bhadvel (Machlipada)	Machharasath		
Orissa	Orissa					
Mayurbhanj	Baripada	Badbilla	Badputka	Kainfulia		
		Bhudurbani	Chandwa	Mundripal		
		Mohiniganj	Tetulisol	Gardunesa		
		Patharchukli	Bramanmara	Sunputuka		
W Bengal	W Bengal					
W Midnapur	Jhargram	Amakuli†	Kherikabad	Medini		
		Bhagabandh	Banstola,	Kulshibanga		
		Chhota Purulia	Khairbani	Lohamalia		
		Jainagar	Nuniakundri	Lalbona		
Jharkhand	Jharkhand					
Ranchi	Bero	Hulsi	Bhandra	Tutlo		
		Katarmali	Deghia	Kamalpur		
		Muramo	Jamtoli	Mukunda		
		Nehalu	Benjara	Katpali		
Hazaribagh	Katkamsandi	Bajha	Beed	Kud		
		Hatkauna	-	-		
		Kherika	Uridiri	-	Borogora	Lakhnu
		Rajhar	Jhalaria	-		

†Found to be in adjacent Binpur-2 block when GD was conducted.

Selection of states and districts

The focus of DFID funded projects was states, districts and villages with extreme poverty and where the people were largely resource-poor tribal smallholders.

The EIRFP worked in Jharkhand (previously southern Bihar), West Bengal and Orissa states. The village surveys by GVT in the beginning of EIRFP in 1995 reported that rice was the most important crop of farmers of rainfed uplands and that resource-poor tribal farmers had smallholdings (0.4 to 0.8 ha) of sloping, poor-fertility, drought-prone land comprising of some 40% of largely degraded upland. Similarly, the WIRFP worked in Rajasthan, Gujarat and Madhya Pradesh predominantly with smallholder tribal farmers.

For the study in EIRFP states all three were selected but in the WIRFP only district Banswara in Rajasthan was selected because this was considered to have more upland rice than any of the other districts in which the WIRFP worked.

Blocks within states were selected where seed of the Ashoka varieties had been distributed in 2002 or 2003 by GVT as this allowed enough time for any possible impact to have taken place. These were three blocks in Banswara district and villages were randomly selected from all three blocks, Rajasthan (Table 4), one block in Mayurbhanj district, Orissa, one block in West Midnapur district, W. Bengal, and two blocks in Ranchi district (from which one was selected) and one in Hazaribag district in Jharkhand. Two districts were selected in Jharkhand because this was the state for which the Ashoka varieties had been released and where most seed distribution had been made. Ranchi was near the GVT headquarters and Hazaribag near a station of ICAR's Central Rice Research Institute, Cuttack. i.e., the Central Rainfed Upland Rice Research Station (CRURRS), Hazaribag. This was intentionally selected to enable a comparison of the extent of use of the CRURRS upland rice variety Vandana (released in 1992) with that of the Ashoka varieties.

Selection of villages

To select the villages GVT and CAZS-NR compiled a complete inventory of seed distribution of the Ashoka varieties by organisation, state, district and village in eastern and western India. From this inventory lists for seed distribution made in 2002-03 were drawn up. The Case Study Team Leader then selected blocks within states where there was a sufficiently large number of farmers who had been given seed. Villages within blocks were selected at random from among all those where seed had been supplied. Two subsequent changes to the selection of villages were required because in village Masu in Bero block of Ranchi district the inhabitants were not cooperative so this was changed to village Nihalu in Bero block) and Ambakul in Baripada block of Mayurbhanj district could not be found (perhaps it was actually the name of a self-help group in another village) and was changed to village Barbilla).

The location of the districts is shown in Fig 2.

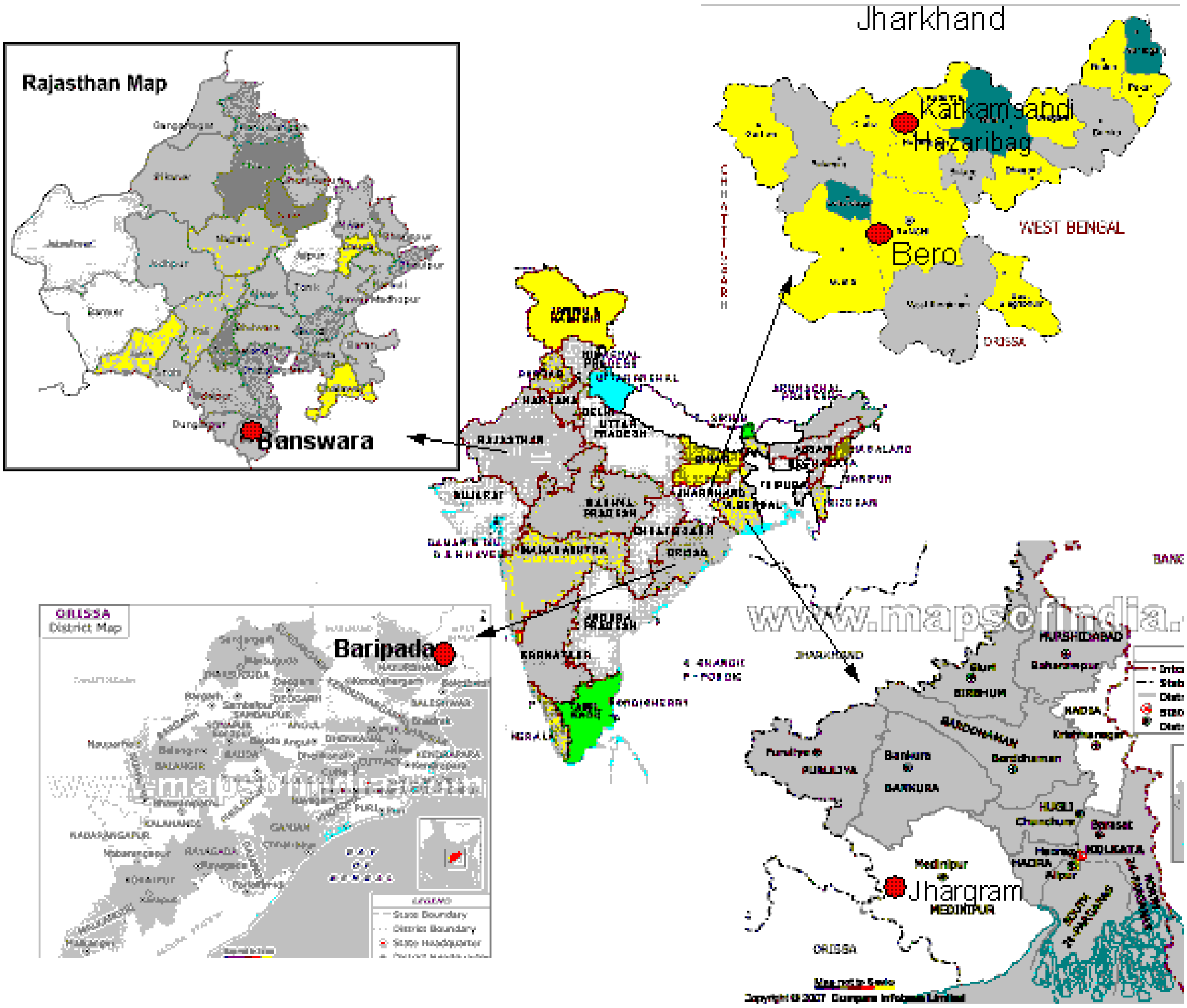

Fig. 2. The location of the study districts/blocks in four states.

Selection of households for interview

Households in the selected villages were selected randomly from list of rice growers that was prepared in group discussions (see below).

Group discussions

In all villages the same structure for the group discussion was followed:

1. Identifying Ashoka users and non-users among the rice users in the village that allowed an estimate of the proportion of households in the village using Ashoka varieties.
2. In the primary villages only identifying Ashoka variety users who were target and non-target (these terms are defined below).
3. Identifying villages to which farmers had distributed seed.
4. Estimating the proportion of the rice land in the upland and medium land devoted to the Ashoka varieties on a village basis.
5. Learning how farmers came to know about Ashoka varieties.
6. Finding how they got the seed of them e.g. from relatives or friends or market
7. What was the situation regarding accessibility of seed.

For full details of the group discussion see Annex 1.

A list of rice growers was compiled which was divided into: current Ashoka variety users, who were either 'target' (i.e., who received seed from GVT) or 'non-target' (who received seed from some other source); and current non-users of Ashoka varieties. In each of the 20 initial, randomly selected villages, termed 'primary villages', farmers were asked the most important villages to which they had distributed seed to others and these were termed 'secondary villages' (Table 4). All villages which had received seed of the Ashoka varieties were identified and marked on a block map. Two secondary villages per primary village were selected, if possible, though in some cases there were less than two (Table 4), for a group discussion. In Hazaribag district, only four secondary villages were identified. To increase the sample size from only four, a group discussion was held in two villages, termed tertiary villages, that were identified in a group discussion in one of the four secondary villages as having received seed from that village (Table 4).

Household survey

The objectives of the household surveys included:

1. Compare the household characteristics of user and non-users of the Ashoka varieties.
2. Estimate the benefits of the Ashoka varieties and impact on food availability of users
3. Estimate the trends and extent of use by area and household numbers
4. Varietal replacement trends by Ashoka varieties
5. Persistence of use of Ashoka varieties
6. Seed exchange dynamics

Selection of households

The group discussion provided the list of current Ashoka users and in each village 15 farmers were interviewed; 5 target and 5 non-target users and 5 non-users (Table 5). A random selection was made from the list because it allowed generalisation of results – it gives confidence that they are representative. However, across all districts four non-user respondents were not available during the visits of the enumerators and so could not be interviewed. To avoid bias (available replacements could be systematically different to those not available depending on the specific reasons for being available or not) no replacements were made.

To randomly select farmers the random function was used in the relevant Excel spreadsheets to produce lists of selected farmers in each category i.e. target user, non-target user and non-user.

Table 5. The sampling frame for the household surveys in the primary villages.

				Farmer type		
**State**	**District**	**Block**	**Village**	**Non-user**	**Target user**	**Non-target user**
Rajasthan	Banswara	Bagidora	Bojapada	5	5	5
		Kushalgarh	Bhuripada	5	5	5
			Sundaripada	5	5	5
		Sajjangarh	Rohaniamanna	5	5	5
Jharkhand	Hazaribag	Katamsandi	Bajha	5	5	5
			Hatkauna	5	5	5
			Kherika	5	5	5
			Rajhar	4†	5	5
Jharkhand	Ranchi	Bero	Hulsi	5	5	5
			Katarmali	5	5	5
			Muramu	5	5	5
			Nehalu	4†	5	5
Orissa	Mayurbhanj	Baripada	Badbila	4†	5	5
			Bhudurbani	5	5	5
			Mohaniganj	5	5	5
			Patharchakuli	5	5	5
W Bengal	West Midnapur	Binpur-2	Amakuli	5	5	5
		Jhargram	Bhagabandh	5	5	5
			Chota Parulia	4†	5	5
			Jainagar	5	5	5
	Grand Total			96	100	100

† One farmer was not available for interview during visits to the village.

Format of questionnaire

Two household level questionnaires were prepared for structured household survey. A meeting of Case Study Team (CST), Indian partners, Nepalese partners and CAZS-NR was organised by MIL component of RiU at the National Livelihood Resource Institute (NLRI), Ratlam, MP (India) from 3 to 9 June 2008 to develop the questionnaires from drafts prepared by CST and CAZS-NR based on a previous study conducted by CAZS-NR in 2004 (Virk and Witcombe, 2007). The questionnaire for users of Ashoka varieties had 52 questions and the questionnaire for non users of Ashoka varieties but growers of rice had 39 (Annexes 2 and 3). The information common to both questionnaires concerned: basic information about the household; agricultural information including livestock, crops and varieties grown for the last three years; and livelihood information including job holders, government scheme employment, number of farm workers and migration. The user questionnaire asked additional information about the Ashoka varieties.

Poverty index

Poverty index was calculated according to the method decided in Dhulikhel, Nepal meeting from 23 to 29 January 2009 (Annex 4). The overall poverty score of a household was the sum of six component scores (Table 6). The maximum possible total score was 25, and the poverty line was drawn at 12.5.

Table 6. The indicators and scores used for the poverty index.

	Score						
**Indicator**	**0**	**1**	**2**	**3**	**4**	**5**	**6**
Livestock units	<1	1-<3	3-<5	5-<10	>=10		
Food production per capita (kg)	<180	180-<365	365-<730		>=730		
Roof material	Thatch	Tile	Tin	Pucca/ concrete			
Jobholders in the family	No job		1 job		2 jobs		3 or more
Tractor ownership	No tractor					Own tractor	
Seasonal unskilled labour Migration	Wife migrates	Husband migrates		Neither migrates			

Administration of questionnaires

The questionnaires were piloted with farmers in Komboi and Jada villages in Dahod district (Gujarat) in WIRFP and in Mehru village of Ranchi district (Jharkhand) in EIRFP between last week of February and early March 2008. The aim of piloting was to assess the time taken to fill-up a questionnaire, the clarity of the wording and to identify any other limitations. The questionnaires were subsequently revised in June in Ratlam meeting.

Four teams of two enumerators each were formed by the GVT. All four teams worked together in one primary village in Banswara from 16 to 24 July 2008, under the supervision of Dr J.P. Yadvendra of GVT and Dr D.S. Virk of CAZS-NR, to pilot the revised questionnaires and to ensure that the group discussion methods and use of the household questionnaires would be consistently applied across the teams. One team continued until the end of August 2008 in Banswara district to complete the group discussions and household surveys in all of the villages there.

The three other teams undertook group discussions and household surveys from 1 August to 15 September with one team per district: Mayurbhanj (Orissa), W. Midnapur (W. Bengal) and Ranchi (Jharkhand). Work in Hazaribag district (Jharkhand) was undertaken by one of these three teams from 15-30 September 2008.

After the data collection each team entered the data of the group discussions and the household surveys in Excel sheets. The data were checked for discrepancies by GVT and CAZS-NR staff and corrected.

RESULTS AND DISCUSSION

Seed spread

Passing of seed from farmer to farmer is the most important mechanism in the spread of a new variety when formal seed supply is limited, as is the case with the Ashoka varieties. Hence, we first examine the extent of this process.

Seed spread estimated from group discussions

In the group discussions the total seed transactions, from the previous harvest in 2007, for both within the village and to other villages were recorded (Table 7). In many cases all of the transactions reported by the group were with farmers from other villages. However, at least from harvests prior to 2007, there must have been many within-village transactions; by definition, non-target users of the Ashoka varieties had to get seed from sources other than GVT; and neighbours in the same village are the most obvious source of seed. Hence, the very high proportion of transactions to other villages is either a result of under-recording of within-village transactions - perhaps because the key informants took more notice of distribution to outsiders – or the process of seed dissemination by within-village transactions was complete. However, the household survey revealed much higher within-village transactions than were found in the group discussions (see below “seed spread estimated from survey of households”) so under reporting by key informants seems the correct explanation.

Table 7. Seed dissemination from the 2007 harvest by users in the primary villages of Ashoka varieties within their primary villages and to secondary villages as recorded in the group discussions in 2008.

District **Primary village**	**Total seed transactions (no.)**	**Within-village transactions (no.)**	**Outside village transactions (no.)**	**Transactions to secondary villages (%)**	**Maximum distance (km)**
Banswara					
Sundripara	7	1	6	86	35
Bhuripara	6	1	5	83	24
Rahaniya	5	0	5	100	10
Manna					
Bhojapara	1	0	1	100	6
Mayurbhanj					
Patharchukli	2	0	2	100	3
Bhudurbani	2	0	2	100	4
Badbilla	2	0	2	100	3
Mohiniganj	2	0	2	100	3.5
W. Midnapur					
Jainagar	3	0	3	100	4
Bhagabandh	4	0	4	100	3.5
Chhota Purulia	4	0	4	100	6
Amarkali	3	0	3	100	9
Ranchi					
Hulsi	3	0	3	100	18
Katarmali	3	0	3	100	7
Muramo	3	0	3	100	16
Nehalu	3	0	3	100	9
Hazaribag					
Hatkauna	0	0	0	0	0
Kherika	1	0	1	100	2
Bajha	3	0	3	100	4
Rajhar	1	0	1	100	1.5

The group discussions collected data on the number of villages to which the seed was distributed from each of the four primary villages in each district. In the study districts of Mayurbhanj, W. Midnapur, and Ranchi, farmers in the primary villages distributed seed of the Ashoka varieties to a total of 8, 14 and 12 secondary villages, respectively; and in Banswara to 7 and in Hazaribag to 5 secondary villages (Table 7). The number of secondary villages will be underestimates of the total number of villages as the group was only asked about the **main** villages to which seed was distributed (Annex 1) and not for details of all of them. Moreover, the members of the group would not have been aware of all of the seed transactions by all of the households in the village.

Only one secondary village was identified in Bhojapra primary village, Banswara: in this village rice was little grown and farmers preferred to cultivate maize or soybean. Another exception was in Hazaribag district where three villages had either no secondary village (one case) or only one secondary village (two cases). As was the case in Rajasthan, they were also located in an area where upland rice is little grown.

Farmers in the secondary villages distributed seed to yet more villages that we term as tertiary villages (Table 8). As for the primary villages, the majority of seed transactions from farmers in the secondary villages were reported as being to outside villages and the reasons for this were discussed above. Farmers in the primary village of Kherika, Hazaribag distributed seed to the secondary village Uridiri. It was found that farmers in Uridiri had distributed seed to two quite-distant tertiary villages where upland cultivation was more important. One of these was village Lakhnu. Farmers were interviewed there and included Mr Tousif Khan who received seed from Uridiri village in 2007 and produced a harvest of 1.4 t some of which he distributed to other farmers in the village. In 2008, he produced 2.4 t and intends to sell 1.8 t as seed through a local seed trader who will sell to yet more villages. Hence the process is an ever expanding pattern but for practical reasons this study was designed not to go beyond the tertiary level.

As has been recorded in previous studies in India, e.g. Witcombe *et al.* (1999), some of the seed transactions were to distant villages, in this case up to 60 km away from the secondary villages and up to 35 km away from the primary villages. Nonetheless, there would be a greater likelihood of seed being distributed to nearby villages, as was the case for Banswara (Figs. 3 and 4).

Table 8. Seed dissemination of Ashoka variety by users from primary to secondary and secondary to tertiary villages, as recorded in group discussions in 2008.

	**Seed source for secondary village (primary village number)**	**Secondary villages †**	**Seed source for tertiary village (secondary village number)**	**Tertiary Villages**
**Banswara**				
01 Bhojapra	1	**01 Jeharpara**	3, 4	Mokampura (2ndry also)
02 Bhuripara	2	**02 Suryan**	4	Kalinjra
03 Sundaripara	2	**03 Kundiya**	5	Badrapara
04 Rohania Manna	3	**04 Haldupara**	6	Bhadvel (2ndry also)
	3, 2	**05 Khanapara**	6	Kushalipara (2ndry also)
	4	**06 Macharasath**	6	Mahudi
	4	**07 Bhadvel**	7	Merana
	2, 3	08 Mokampura		
	2, 3	09 Nalpara		
	3	10 Nalwai		

	3	11 Badi Sarva		
	4	12 Kushlipara		
	4	13 Dungra		
	4	14 Sajjangarh		
**Mayurbhanj**				
01 Patharchakli	1	**01 Sunputuka**	3	Damodarpura
02 Bhudurbani	1	**02 Brahamanmara**	4	Bhurikumari
03 Badbilla	2	**03 Chandwa**	6	Padurisole
04 Mohiniganj	2	**04 Mundripal**		
	3	**05 Badputka**		
	3	**06 Kainfulia**		
	4	**07 Tetulisol**		
	4	**08 Gardunesa**		
**W. Midnapur**				
01 Jainagar	**1**	**01 Nuniakundri**	1	Hudhudi
02 Bhagaband	1, 2	**02 Lalbona**	1	Nischintapur (2ndry also)
03 Chhota Parulia	2	**03 Banstola**	2, 3	Chapasol
04 Amakuli	2	**04 Kulshibanga**	2	Bhakra
	3	**05 Khairbani**	3	Jambadia
	3	**06 Lohamalia**	5	Lodhasuli
	4	**07 Kherikabad**	7	Ergada
	4	**08 Medini**	7	Kulbadia
	2	09 Nischintapur		
	3	10 Bara Purulia		
	3	11 Asanshila		
	4	12 Parihat		
**Ranchi**				
01 Hulsi	1	**01 Bhandra**	1	Baridihi
02 Katarmali	1	**02 Tutlo**	3	Karanjia
03 Muramo	2	**03 Deghia**	4	Dumri
04 Nehalu	2	**04 Kamalpur**	5	Khatri-Khatanga
	3	**05 Jamtoli**	6	Boda
	3	**06 Mukunda**	7	Sakarpur
	4	**07 Benjara**	8	Chhopari
	4	**08 Katpali**		
	1	09 Masu		
	2	10 Karanjia		
	3	11 Dola		
	4	12 Khirda		
**Hazaribag**				
01 Hatkauna	2	**01 Uridiri**	1	**Borogora**
02 Kherika	3	**02 Kud**	1	**Lakhnu**
03 Bajha	3	**03 Beed**	1	Sakarja
04 Rajhar	4	**04 Jhalaria**	1	Nachle
	3	05 Nainadhor	1	Bajha (Pry also)
			1	Khera
			2	Danto
			4	Barikola

† Villages in bold were surveyed by FGD.

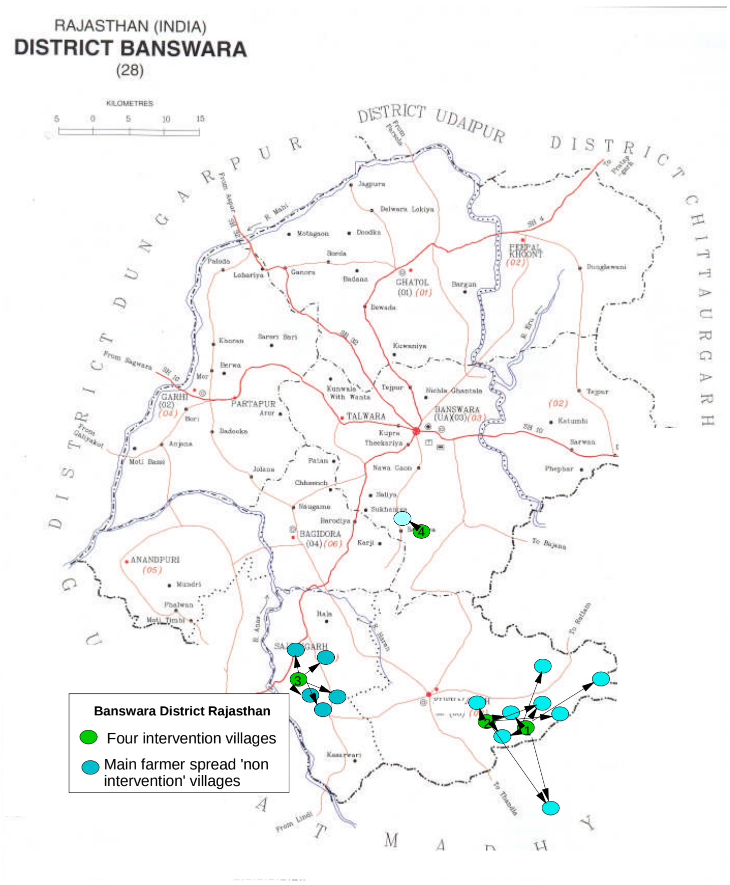

Fig 3. Distribution of seed from four primary villages (green circles) to secondary villages (blue circles) in Banswara as identified in the group discussions in the primary villages in 2008.

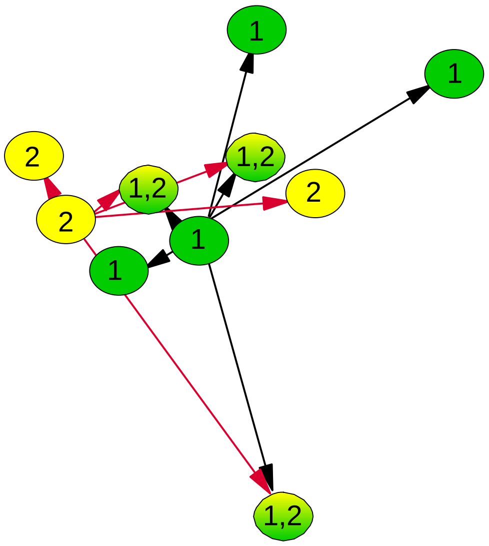

Fig 4. Details of spread from two primary villages, Sundaripada (1) and Bhuripada (2). From these two villages seed is distributed to eight new ones. Three of the villages (1,2) are in common.

Seed spread estimated from survey of households

The household surveys confirmed that farmers were distributing seed and, given that only 10 users were interviewed per primary village and the number of transactions (Table 9) was higher than in the group discussions (Tables 7, 8), the seed distribution appears to be under estimated in the latter case. Apart from the case of Ranchi district, at least a third of farmers that grew the Ashoka varieties distributed seed of them with at least half of the transactions

being to outside villages (Table 9). In three of the districts the surprising result was that 100% of the farmers only distributed seed to one recipient. This seems unlikely in this relatively large sample (37 households), as in the remaining two districts, and other studies (Witcombe *et al.*, 1999), the frequency of multiple distribution was high.

Seed quantities distributed were sufficient, on average, to plant over a tenth of a hectare (assuming 100 kg ha^{-1} for broadcast upland sowing). This represents a reasonable proportion of the rice land (Table 10).

Most of the seed was distributed as an exchange or a gift with only 12% of transactions being by sale (Table 11). Most recipients were relatives or neighbouring farmers illustrating the reliance on the informal sector in the farmer rice innovation system.

Table 9. Distribution of seed of the Ashoka varieties by users according to number, amount and distance of distribution, from a household survey of 200 users in 5 districts and 4 states 2008.

	Seed distributors		Seed recipients				
**District**	**HH (No)**	**HH (%)**	**Total HH (No.)**	**Within village (%)**	**In secondary villages (%)**	**Average distance (km)**	**Average quantity (kg)**
Ranchi	3	8	3	0	100	8	14
Hazaribag	13	32	13	46	54	9	13
Mayurbhanj	21	52	21	62	38	3	11
W. Midnapur	15	38	17	53	47	8	36†
Banswara	21	52	38	47	53	7	9
All 100 target	53	53	69	58	42	5	18
All 100 non target	20	20	23	26	74	9	11

† One farmer sold 400 kg to a trader. It is not known if this was as seed or grain.

Table 10. Average rice landholding by district of the surveyed 100 target and 100 non-target users, from a household survey of 200 Ashoka rice users in 2008.

District	Non-target users (ha)	Target users (ha)	Average (ha)
Banswara	0.31	0.43	0.33
Hazaribag	1.47	1.27	1.31
Mayurbhanj	0.83	0.72	0.73
Ranchi	0.78	0.69	0.90
W. Midnapur	0.94	1.09	0.85
Average over all districts	0.87	0.84	0.82

Table 11. Seed distribution of the Ashoka varieties by type of recipient and transaction, from a survey of 200 users in primary villages in 5 districts and 4 states in 2008.

District/	Recipients	Type of seed recipient (%)				Mode of transaction (%)		
**Block**	**(number)**	**Farmer**	**Trader**	**Relative**	**Friend**	**Exchange**	**Gift**	**Sold**
Ranchi	3	33	0	67	0	0	67	33
Hazaribag	13	23	0	62	15	38	54	8
Mayurbhanj	21	67	0	23	10	86	14	0
Jhargram	17	18	6	53	24	53	35	12
Banswara	38	37	8	55	0	76	16	8
District mean	18	36	3	52	10	51	37	12

Seed spread estimated from non-target users in household survey

In each primary village non-target users were identified. By definition, they would not have received seed from GVT and must have obtained the seed from other farmers within their village, a neighbouring village or an informal commercial source[4]. All of these farmers (apart from three in Ranchi district) grew the Ashoka varieties one or more years after their first introduction to target farmers (Table 12). Summarising these data across all the districts the adoption curve for the non-target users approximates to a classic S-shaped adoption curve (Fig. 5) indicating that adoption levels in the primary villages were reaching their ceiling of maximum adoption.

Table 12. Year in which the farmers who grew an Ashoka variety first grew it shown by the two classes of farmers, ‘target’ and ‘non-target’, from a survey of 100 target and 100 non target users in primary villages in 5 districts and 4 states in 2008.

		**Year farmer first grew Ashoka (number of farmers)**								
**District (state)**	**User**	**01**	**02**	**03**	**04**	**05**	**06**	**07**	**08**	**Total**
Ranchi	Target			17	2	1				20
	Non target			3	11	6				20
Hazaribag	Target			20						20
	Non target				7	13				20
Mayurbhanj	Target		20							20
	Non target				5	1	3	4	7	20
W. Midnapur	Target			20						20
	Non target				12	8				20
Banswara	Target			20						20
	Non target				11	6		3		20

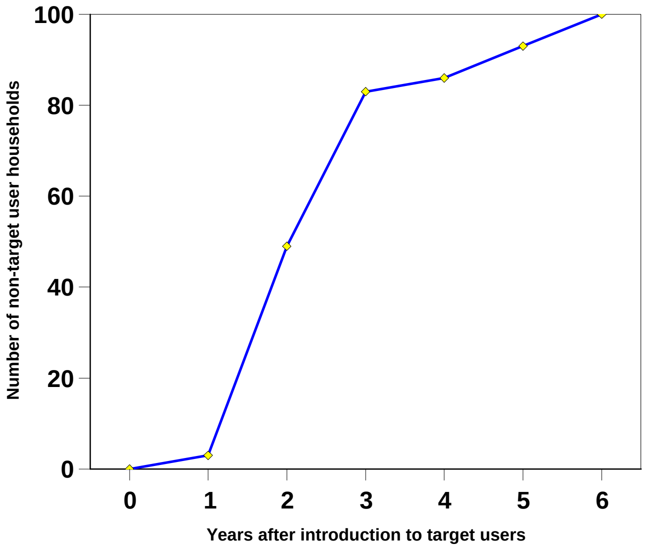

Fig 5. The rate of adoption by new non-target users following the introduction of the Ashoka varieties to target farmers in the villages. One hundred non-target users were randomly sampled in 2008 and asked in which year they first grew an Ashoka variety.

[4] No private-sector seed company has taken up the production and sale of these varieties.

Persistence of use

Group discussions

Having considered the extensive spread from farmer to farmer both within and across villages, the next indicator of the acceptability of this agricultural technology is whether farmers, once they gain access to seed, continue to use it or not. In the group discussions (for preparing the list of Ashoka users) in the primary villages in four of the five districts all of the target farmers that first had access to seed of an Ashoka variety in 2002 or 2003 were still growing it in the *kharif* of 2007 (Table 13). Only in Ranchi district, Jharkhand, had some farmers discontinued: 77% of those that received seed of an Ashoka variety continued to grow it, and all farmers that had discontinued cited the non-availability of seed of Ashoka varieties as the reason for not growing them. However, we have no explanation as to why this should apply to Ranchi district and not to other districts other than the speculation that there is greater competition from private-sector hybrids. However, in Muramo primary village, Ranchi district, and its secondary villages, farmers also mentioned damage to the Ashoka varieties from elephants. They said these varieties attract elephants more than other varieties and landraces because of their earlier growth and more palatable straw.

Table 13. Proportion of target farmers in primary villages who had continued to grow an Ashoka variety up to *kharif* 2007 who had originally received seed of an Ashoka variety from GVT in 2002 or 2003, from the list of users prepared in group discussions in 4 primary villages in 5 districts and 4 states in 2008.

**District (State)**	**Households in primary villages who received seed in 2002-2003 of Ashoka variety (number)**	**Households who were still growing an Ashoka variety in *kharif* 2007 (number)**	**Proportion of continuing users (%)**
Banswara (Rajasthan)	23	23	100
Mayurbhanj (Orissa)	28	28	100
Ranchi (Jharkhand)	35	27	77
Hazaribag (Jharkhand)	28	28	100
W. Midnapur (W. Bengal)	29	29	100
Mean			95

Household surveys – users

In all districts, except Hazaribag and one case in Mayurbhanj, the farmers identified in the group discussions as current users, who were randomly selected for individual interview, were confirmed by the household survey interviews as users. However, in Hazaribag, 35% of those identified as users and subsequently interviewed were found to be discontinuers. All had been users in 2006 and many had been users in 2007, but had been misidentified as current users in 2008 in the group discussions. Two of the discontinuing users were target farmers while 6 were non-target farmers (Fig. 6). As these farmers were interviewed using the user questionnaire no questions were asked about the reasons as to why they were no longer growing an Ashoka variety.

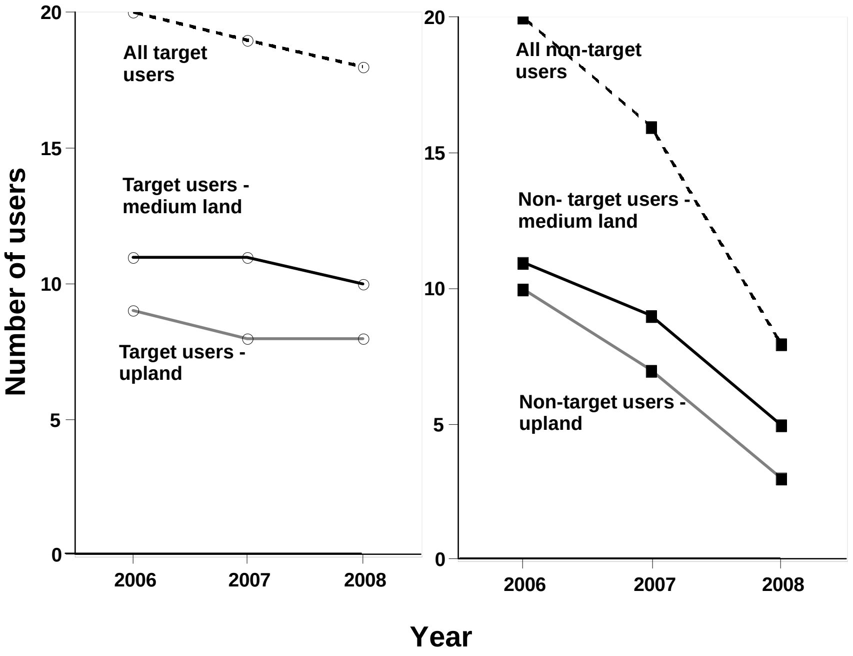

Fig. 6. Use of Ashoka varieties by target and non-target users in Hazaribag from 2006 to 2008 from the household survey in 2008.

Household survey – current non-users

In the household survey in primary villages the non-users were asked why they were not growing the Ashoka varieties. Overall most of the non users (60%) had not heard of them and this was particularly true in three districts - Ranchi and Hazaribag in Jharkhand and Mayurbhanj, Orissa (Table 14). This lack of knowledge applied even though they were in the same villages as users. In some villages the households are widely dispersed and there may be less than what might be expected to be high communication between hamlets in the same village. In two districts, Banswara and W. Midnapur, about 60% of the non-users of the Ashoka varieties had heard of them (Table 14).

Overall, about 23% of those that had heard of an Ashoka variety had actually grown one of them previously (Table 14) a result that differs from the target farmers in the group discussion where 95% of target farmers were reported to have continued adoption (Table 13). The current non-users would mainly be non-target farmers and, hence, they may find it more difficult to procure seed than target users who were always members of a self help group and always knew other farmers from whom they might get seed. Difficulty in obtaining seed was supported by 73% of the non-users that had grown an Ashoka variety responding that they would grow it if they could gain access to seed (Table 15).

Whether those that had heard of the Ashoka varieties had previously tried them or not the reasons for currently not using them remained the same. The great majority (80%) said they could not get seed, while the remainder either said that they did not cultivate upland or did not have suitable land (Table 15). This raises the question as to why the majority had not asked for seed from the users of these varieties in their village; but it implies that improved access to seed could significantly increase adoption levels.

Table 14. Responses (%) of 96 non-user farmers to the questions 'Have they heard about the Ashoka varieties' and 'Have they ever grown an Ashoka variety', from a survey of 96 non-user farmers in 4 primary villages in 5 districts and 4 states in 2008.

	**Have heard about an Ashoka variety?**		**Have ever grown an Ashoka variety?**	
**District**	**Yes responses (no)**	**Proportion (%)**	**Yes responses (no)**	**Proportion of total (%)**
Banswara	13	65	1	5
Hazaribag	6	32	6	32
Mayurbhanj	2	11	1	5
Ranchi	4	21	4	21
W. Midnapur	12	63	10	53
Total	*37*	*39*	*22*	*23*
Sample size (no)	96		96	

Table 15. Reasons for not growing the Ashoka varieties given by the 37 farmers who had heard of them and either had never grown them or had tried them previously, from a survey of 96 non-users in 5 districts and 4 states in 2008.

	**Knows about an Ashoka variety and never grown**		**Grown Ashoka variety previously and discontinued**	
**Reason**	**Responses (no)**	**Proportion (%)**	**Responses (no)**	**Proportion (%)**
Non-availability of seed	12	80	16	73
Not the best variety for land	1	7	2	9
Do not cultivate upland	2	13	3	14
Seed got mixed	0		1	4
Total (no)	15		22	

Extent of Use in the Survey Areas

Use by land type and by area within the study villages

The breeding programme was planned in the RNRRS project to specifically target upland rice farmers, as EIRFP and WIRFP DFID bilateral projects had shown that these more often belonged to disadvantaged communities compared with farmers that only cultivated medium and lowlands. The varieties were bred to be of short duration to make them ideally suited to rainfed uplands. However, the survey showed that upland rice was not grown in all of the districts (Table 16) even though there was cultivated upland. There was even a large difference between the proportion of upland devoted to rice between the tertiary villages in Hazaribag compared with both the primary and secondary villages. It appears as if through village-to-village spread the Ashoka varieties eventually arrive in favourable areas where there is more suitable area for them.

In the study districts of Rajasthan and Orissa where the group discussions had revealed that there was no upland rice, the Ashoka varieties would only be grown if they replaced other crops. These were reported to be: predominantly maize in Rajasthan, mainly Sabai grass, *Ischaemum augustifolium*, grown for paper making, in Orissa; and, in Hazaribag, a wide variety of crops with Niger, *Guizotia abyssinica*, the most important.

All districts, however, had medium rice land where the Ashoka varieties could be grown as a transplanted crop (Table 17).

Table 16. Total rice area (ha) disaggregated into upland, medium land and lowland in all the sampled primary, secondary and tertiary villages in group discussions.

**State** *District* **Village type**	**Total upland area (ha)**	**Upland rice area (ha)**	**Proportion upland area under upland rice (%)**	**Medium land rice area (ha)**	**Lowland rice area (ha)**	**Upland rice area (% rice land)**	**Medium land rice area (% rice land)**	**Lowland rice area (% rice land)**
**Rajasthan**								
Banswara								
4 Primary	102	0	0	26	31	0	46	54
7 Secondary	104	0	0	33	77	0	30	70
**Orissa**								
Mayurbhanj								
4 Primary	190	0	0	77	42	0	65	35
8 Secondary	341	0	0	160	88	0	65	35
**W Bengal**								
W. Midnapur								
4 Primary	126	67	53	56	29	44	37	19
8 Secondary	656	273	42	327	467	25	31	44
**Jharkhand**								
Ranchi								
4 Primary	160	32	20	102	179	10	33	57
8 Secondary	360	110	31	377	301	14	48	38
**Jharkhand**								
Hazaribag								
4 Primary	157	11	7	106	82	6	53	41
4 Secondary	82	4	5	49	56	4	44	52
2 Tertiary	91	44	48	40	37	36	33	31
**Jharkhand**								
Two districts								
8 Primary	317	43	14	208	261	8	41	51
12 Secondary	442	113	26	425	357	14	52	44

Table 17. Rice area disaggregated into upland, medium land and lowland as a proportion of all cultivated land in all the sampled primary, secondary and tertiary villages in the group discussions in 2008.

District Village type	Upland rice area (% all land)	Medium land rice area (% all land)	Lowland rice area (% all land)	Rice land (% all land)
Banswara				
4 Primary	0	8	10	18
7 Secondary	0	9	20	29
Mayurbhanj				
4 Primary	0	25	14	39
8 Secondary	0	27	15	42
W. Midnapur				
4 Primary	32	26	14	72
8 Secondary	19	23	32	74
Ranchi				
4 Primary	6	18	31	54
8 Secondary	8	29	23	60
Hazaribag				
4 Primary	3	31	24	58
4 Secondary	2	26	30	58
2 Tertiary	26	24	22	72
Jharkhand				
8 Primary	5	22	28	55
12 Secondary	8	29	24	60

In the three districts where farmers grew the varieties in the upland many chose to do so and the use of the Ashoka varieties was by 19 to 53% of the households (Table 18). In the four districts where they were used in the medium lands they were grown by 11 to 54% of the households. The only district where it was not grown in the medium lands was W Midnapur, where perhaps farmers had better alternatives for this situation.

When farmers chose to grow Ashoka they did so on a high proportion of their available rice land (Table 19, Figs. 7 and 8). In the upland the minimum average area in the household survey ranged from 57% of the rice upland in W Midnapur to 91% in Ranchi district (Table 19, Fig. 7). In the medium land farmers devoted 9 to 46% of what were larger areas of medium land (Table 19, Fig. 8). Hence, on average, farmers grew the Ashoka varieties on equivalent areas of about 0.15 ha in both the upland and the medium land.

The use of the Ashoka varieties did not change over the three years from 2006 to 2008, presumably as these farmers had already managed by 2006 to get enough seed to grow them on high proportions of their rice land (Figs. 7, 8).

Table 18. Use of Ashoka varieties in upland and medium land by households (HH) in primary and secondary villages from group discussions in 2008.

District	Village class	Ashoka using HH in upland			Ashoka using HH in medium land		
		No.	%	SE	No.	%	SE
Banswara	Primary				104	54	5
	Secondary				118	37	4
Mayurbhanj	Primary				223	27	3
	Secondary				404	31	4
W. Midnapur	Primary	132	53	2			
	Secondary	818	19	6			
Ranchi	Primary	174	33	8			
	Secondary	350	44	10	845	11	5
Hazaribag	Primary	44	21	12	122	39	6
	Secondary	18	39	11	79	41	6

Table 19. Use of Ashoka varieties in *kharif* 2008 by average area per household (HH), shown separately for upland and medium land, among the 40 current users in each of 5 districts in the primary villages in 4 states.

District	Upland			Medium			All
	HH (No.)	Average Ashoka area per HH (ha)	Proportion of upland rice area per HH (%)	HH (No.)	Average Ashoka area per HH (ha)	Proportion of medium land rice area (%)	Proportion of all rice area (%)
Banswara	-	-	-	40	0.15+0.01	79	46
Mayurbhanj	-	-	-	39	0.15+0.01	39	20
W. Midnapur	39	0.14+0.01	57	1	0.04+0.00	5	13
Ranchi	32	0.14+0.02	91	10	0.12+0.02	65	19
Hazaribag	11	0.19+0.04	79	15	0.18+0.04	49	9
District mean	82	0.15+0.01	73	105	0.15+0.01	58	17

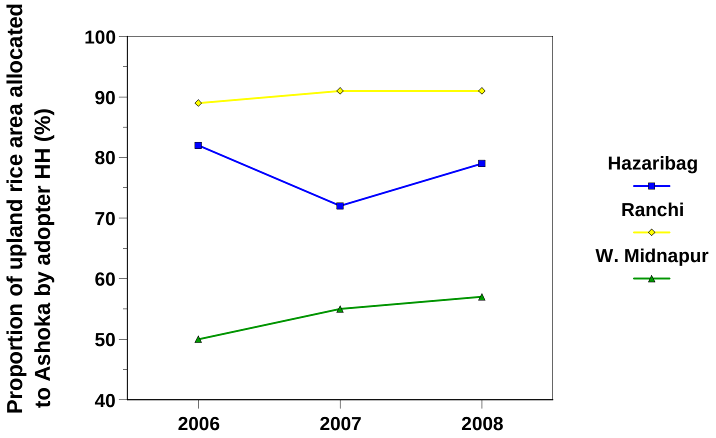

Fig 7. Overall proportion (%) of upland rice area allocated to Ashoka varieties by target and non-target users over three years based on households sampled who grew Ashoka in the upland in Hazaribag and Ranchi districts of Jharkhand state, and West Midnapur district in W Bengal state.

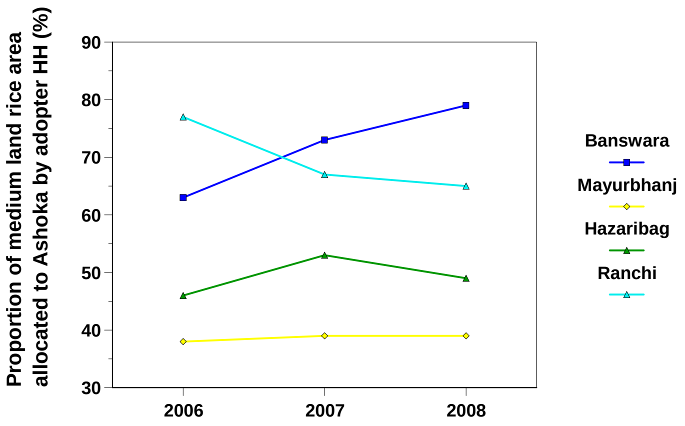

Fig. 8. Overall proportion (%) of medium rice area allocated to Ashoka varieties by target and non-target users over three years based on households sampled who grew Ashoka in the medium lands in four districts and three states, i.e., Banswara (Rajasthan), Mayurbhanj (Orissa), Hazaribag and Ranchi (Jharkhand).

Estimated use by area and households in the study villages by combining group discussion and household survey data

The proportion of total rice area devoted to the Ashoka varieties in the primary and secondary villages was estimated both from the data collected in the group discussions and the data collected in the household survey using the method described in Box 1. The use of the Ashoka varieties by area varied from 1.8 to 24% of total rice area depending on the category of village and the district (Table 20). A combination of group discussion and household survey data also allowed the areas under the Ashoka varieties in each village to be estimated (Table 21). The villages in many cases were not villages as such but hamlets known as *tollas* (in eastern India) or *falias* (in western India). Hence, when extrapolating to the village level we have used somewhat higher values.

Estimated Extent of Use Beyond the Survey Areas

The data in Tables 20 and 21 were then used to extrapolate adoption at different levels:

- the blocks where the study villages were located,
- Jharkhand state as a whole,
- blocks outside of Jharkhand where seed had been distributed and,
- finally, to estimate the potential area that the Ashoka varieties could cover.

Box 1. Extrapolating use in the nine states from the group discussions and the survey data four states

The best overall estimate of the use of Ashoka varieties in the whole of the 3355 rice growing households identified in the group discussions in all 53 villages (16 primary, 35 secondary and 2 tertiary) can be obtained using a combination of the group discussion and the household survey data. The latter is considered more reliable as each sampled household reported on their own experiences.

Group discussion determined that 24% of households (i.e. 806 out of 3355 rice growing HH) were adopters of Ashoka varieties

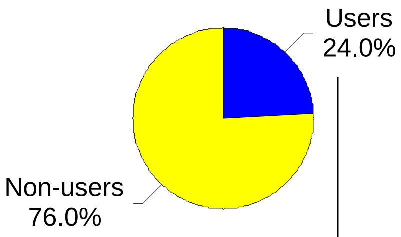

100 non user households in primary villages randomly sampled

200 user households from the 24% in the primary villages randomly sampled and interviewed

Interview shows that on average they allocate 17% of rice land to Ashoka varieties (survey result)

Best estimate of overall adoption by area of Ashoka variety by 3355 households = 24% x 17% i.e. 4.1% of all their rice land is under Ashoka varieties

Table 20. Extrapolated proportion of area of use (%) of the Ashoka varieties at the village level. Estimated from the proportion of households growing Ashoka varieties in the group discussions in 2008 and the proportion of total rice land (upland, medium and lowland) on which they were grown from a household survey in 2008.

District	Villages	Total HH in GD (no)	Total HH that use Ashoka variety in GD (no)	Proportion of HH that use Ashoka variety in GD (%)	Proportion of rice land under Ashoka varieties by users in household survey (%)	Ashoka variety use as proportion of total rice area (%)
Banswara	4 primary	104	55	53	46	24.4
	7 secondary	118	44	37	-	17.0
	7 tertiary	-	-	-	-	-
Mayurbhanj	4 primary	223	58	26	20	5.2
	8 secondary	404	113	28	-	5.6
	3 tertiary	-	-	-	-	-
W. Midnapur	4 primary	125	70	56	13	7.3
	8 secondary	861	119	14	-	1.8
	8 tertiary	-	-	-	-	-
Ranchi	4 primary	275	46	17	19	3.2
	8 secondary	957	184	19	-	3.6
	7 tertiary	-	-	-	-	-
Hazaribag	4 primary	122	34†	29	9	2.6
	4 secondary	101	42	42	-	3.8
	2 tertiary	65	22	34	-	-
Total		3355	787	23	17	4.0

- = no data.

†Reduced by 35% to account for the 35% of users who were found to be current non-users in the household survey.

Table 21. Extrapolated area of use (ha) of the Ashoka varieties at the village level. Estimated from the number of households growing Ashoka varieties in the group discussions in 2008 and the average area of rice land in which they were grown from a household survey in 2008.

District	Villages	Total HH that use Ashoka variety in GD (no)	Average area per HH under Ashoka variety in household survey (ha)	Estimated Ashoka area per village† (ha)
Banswara	4 primary	55	0.15	2.1
Mayurbhanj	4 primary	58	0.15	2.2
W. Midnapur	4 primary	70	0.14	2.5
Ranchi	4 primary	46	0.14	3.2
Hazaribag	4 primary	34	0.19	1.6

†For example, primary villages in Banswara had 55 households growing them on an average of 0.15 ha = 2.1 ha in all village

Estimated use by area and households in the blocks of the study villages

The area under the Ashoka varieties was estimated in the blocks where the study took place (Table 22). About 30,000 ha of rice were estimated to be grown in these blocks by dividing the available data for the rice area of the district by the number of blocks in the district. In the absence of block-level data this was the best approximation possible. Adoption in the blocks of the study villages was assumed to be similar to the study villages themselves. This is a reasonable assumption given that about 10% of the villages in each block were sampled, the high village-to-village spread, and the fact that non-study villages where seed had been distributed in later years received more seed than the early-interaction villages. This was because in 2002, seed was distributed in limited quantities mainly for trial purposes, whereas from 2006 seed was distributed in the Rockefeller Foundation project for scaling up. Given the above assumptions, over 1.5 thousand hectares were under the Ashoka varieties in these six blocks and they would be grown by over 11 thousand households.

Table 22. Extrapolated area (ha) of use in the six blocks in which the surveys were done using the lowest estimates obtained for the proportion of area devoted to the Ashoka varieties in the study villages.

District	Total rice land in district ('000 ha)	Number of blocks in district	Average rice land per block (ha)	Proportion of Ashoka area (lowest % in Table 20)	Total area of Ashoka in block (ha)
Banswara	36	8	4500	17.0	770
Mayurbhanj	210	26	8100	5.2	420
W. Midnapur	110	29	3800	1.8	68
Ranchi	197	21	9400	3.2	300
Hazaribag	69	15	4600	2.6	120
Total (ha)	622		30400		1,670
Total (households)†					11,100

†Assuming average area per user household of 0.15 ha.

Estimated use by area and households in Jharkhand

Jharkhand has had by far the greatest activity in seed dissemination of the Ashoka varieties of all the states, and is the one where it is most reasonable to extrapolate from the block to the state level. We have used the lowest adoption level that was found (2.6% of the area) to be conservative even though later villages received more seed (Table 23). Over 30,000 ha and over 200,000 households grew the Ashoka varieties in Jharkhand if these assumptions are correct.

Table 23. Extrapolated household use and area (ha) in Jharkhand using the lowest estimates obtained for the proportion of households and area devoted to the Ashoka varieties.

State	Total rice area in state ('000 ha)	Proportion of adopters' area (%)	Area under Ashoka (ha)	Number of HH††
Jharkhand	1420	2.6†	36900	217,000

†Lowest of all estimates in villages in Ranchi and Hazaribag (Table 20).
††Assuming average area per user household of 0.17 ha.

For triangulation, another set of assumptions was used based on the data that were available for the number of villages to which seed was distributed. The Ashoka variety areas per village in Jharkhand varied from 1.6 ha in Hazaribag to 3.2 ha in Ranchi district (Table

24) but these survey areas excluded the neighbouring areas within the same village to which spread could reasonably assume to have occurred. Hence, for extrapolation in the primary villages an area of 4 ha is used as a reasonable estimate. We assumed lower use than this in the secondary villages and even lower use in the tertiary ones. The number of primary villages reached by the DoA was derived from the known distribution of a total of 300 t over a period of three years by assuming that an average of 120 kg was distributed per village. This fits with the general practice of the DoA. If seed distribution was repeatedly made to the same villages over years this would result in an estimate of seed distribution to 1000 villages. If the distribution was not repeated in any village but was always to new villages, 3000 villages would have received seed. The numbers of secondary and tertiary villages were extrapolated from the group discussions (which were known to be underestimates as each group was only asked to identify the principal secondary villages). Hence, this method may be an underestimate, particularly as an estimate of only one tertiary village per secondary village is used. Using these various assumptions the estimated use of the Ashoka varieties in Jharkhand varied from 13,000 to 33,000 ha using this method (Table 24); and was in agreement with the other, whole-district, method (Table 23).

Table 24. Extrapolated household use and area (ha) in Jharkhand using the estimated number of villages to which Ashoka seed has been distributed and a conservative and realistic range for the estimate of the number of secondary and tertiary villages and estimates for the area (ha) of Ashoka varieties in each village.

Village type	Estimated number of villages	Assumed area of Ashoka varieties per village (ha)	Estimate of total area under Ashoka varieties (ha) Conservative	Realistic
Primary (GVT)	300	4	1200	1200
Primary (Dept of Agric)	1000-3000	4	4000	12000
Secondary (GVT)	600	2	1200	1200
Secondary (Dept of Agric)	2000 to 6000	2	4000	12000
Tertiary (total)	2600 to 6600	1	2600	6600
Total (ha)			13000	33000
Total households†			76,500	194,000

†Assuming average area per user household of 0.17 ha.

Estimated use by area and households in blocks outside of Jharkhand where seed of the Ashoka varieties had been distributed

The combined seed distribution of the Ashoka varieties by GVT and CRS outside of Jharkhand covered 94 blocks (Table 25). If we assume that these blocks have an average of around 6000 ha of rice (this is the mean of the blocks in the five study districts) and the lowest use found in the study of 2% of the area then there are 11,280 ha of Ashoka varieties in these blocks grown by 75,200 households. This disregards seed distribution to other villages outside of these blocks.

Whatever the errors in the estimates for Jharkhand, when all of the states of India where seed has been distributed are added then the use in these states has to at least compensate for any possible overestimate in the realistic scenario for Jharkhand. Hence, the minimum use of the Ashoka varieties is expected to be by at least 194,000 households who grow the variety on a total of over 33,000 ha.

Table 25. Extrapolation to blocks outside of the study areas and Jharkhand where GVT and CRS have distributed seed by 2007. GVT seed distribution commenced in 2002 in all the listed blocks and CRS commenced in 2005 in all of the listed blocks

	GVT				CRS				
State	Blocks (GVT)	Villages (no)	Farmers (no)	Seed (t)	Blocks	Villages (no)	Farmers (no)	Seed (t)	Blocks (total)
AP					1	1	28	1	1
Chhattisgarh					14	43	1678	36	14
Gujarat	7	106	370	4					7
MP	8	69	709	7	9	99	6477	110	17
Orissa	15	109	3397	20	4	20	192	2	19
Rajasthan	5	53	527	5					5
UP					15	62	1239	18	15
West Bengal	14	126	9942	73	2	4	23	0.3	16
Total	67	752	19146	144	52	252	10473	179	94

Estimated potential use in the four study states by area and households

Using the data from the group discussion and the household surveys the potential adoption of the Ashoka varieties can be estimated in the study districts and the five states in which they were located (Table 26). Even though the impact at each individual household may be small when taken in any single year the potential for the overall combined impact of the varieties is very large and, assuming a sustainable seed supply, could be measured in hundreds of thousands of hectares each year and by millions of households (Table 26).

Table 26. Estimated potential area of adoption at district and state level extrapolated from the adoption found in the group discussions and the household survey in 5 districts and 4 states conducted in 2008.

Identified area	Total rice land in state or district from govt. data ('000 ha)	Proportion of total rice land area under Ashoka varieties assumed from survey results† (%)	Total area under Ashoka vars. extrapolated from estimates of adoption (ha)
Banswara district:	32	17.0	5,500
**All Rajasthan**	180	17.0	30,600
Mayurbhanj district:	210	5.2	10,900
**All Orissa**	4500	5.2	234,000
W. Midnapur district:	110	1.8	2,000
**All W Bengal**	6070	1.8	109,000
Ranchi district:	167	3.2	5,300
Hazaribagh district:	110	2.6	4,200
**All Jharkhand**	1480	3.2	47,400
**Total all five districts**			26,600
**Total all four states**			420,000
**Total households††**			2,800,000

† Value of either primary or secondary villages from estimated area (%) in last column of Table 20. Lowest value of the two used to provide conservative estimate.
††Assumes average of 0.15 ha per user household.

The financial value of the additional grain yield from over 400,000 ha of Ashoka varieties is high. Even if we assume a low average yield on this entire area of 700 kg ha^{-1}, growing the Ashoka varieties instead of those currently grown produces an additional 15% of grain with a value of around £17 million. This assumes the rice grain price is £400 t^{-1}, wich is a typical local price based on the authors' knowledge This benefit accrues every year and does not include other benefits such as better eating quality, early harvest and a more assured yield in drought years.

The maximum costs are easy to estimate for the PSP Research and the RF funded project. The total cost of PSP the research projects that relate at all to producing the Ashoka varieties (R7122, R7434 and R8099 – see Introduction) was £466,000 from 1998 to 2006. This is an overestimate as the Ashoka varieties were already released by 2003. The RF funded project from 2005 to 2009 had a total of budget of £382,000 but at most one third of this was spent on scaling out Ashoka varieties. It is more difficult to estimate expenditure in the GVT EIRFP but the total expenditure on supporting all the plant breeding activities in BAU by GVT from 1997 to 2000 did not exceed £10,000.

These estimates of benefits and costs illustrate that a more detailed and comprehensive economic or financial analysis, using constant rather than mixed pounds, is likely to show a high return on the investment in breeding these varieties.

What rice varieties do the Ashoka varieties replace?

In the uplands

The Ashoka varieties were targeted specifically for the uplands so we estimated from the survey how successful they have been compared to other breeding efforts by the public and private sector. There were three districts where the Ashoka varieties were adopted in the uplands (Table 27) and the varietal adoption among the 120 users and 60 non-users was analysed[4]. Many of these farmers did not grow rice in the uplands so the sample sizes are lower (Table 27). Farmers were asked to give the varieties and areas for three years (2008, 2007 and 2006) but there were no significant differences between the years – users had already adopted the varieties on significant areas of land by 2006 – so only data for 2008 are shown.

Table 27. Overall proportion of upland rice area allocated by Ashoka users and non-users to modern varieties (private and public sector) and landraces in 2008 in the three districts where Ashoka varieties were grown in the uplands. From a survey of 120 user households and 60 non-user households in 12 primary villages in 3 districts and 2 states in 2008.

District (State)	Farmer class†	Number growing rice in uplands (N)	Mean area of Ashoka (%)	Mean area of public-sector upland varieties (%)	Mean area of other MVs†† (%)	Mean area of landraces (%)	Released public sector upland varieties present in sample
Ranchi (Jha)	U	32	76±7	0	12±5	11±5	None
	N-U	4	0	0	100+0	0	None
Hazaribag (Jha)	U	34	40±8	0	0	60±8	None
	N-U	7	0	5±5	0	95±5	Vandana (5%)
W. Midnapur (WB)	U	39	44±8	21±6	31±7	4±3	Kalinga III (16%)* Annapurna (5%)
	N-U	16	0	56±11	44±11	0	

†U = user and N-U = non-user.
†† MV = Modern varieties from public and private sector.
* Known by farmers in this district as Culture 1

In Ranchi district, Jharkhand, there were only four non users and they grew modern varieties (Suruchi, Shankar, JK and Advanta mostly recommended for medium lands) on all of their land. Shankar and Surichi are medium-land public-sector varieties from Orissa but the others appear to be hybrids from the private sector although not all of the variety names could be traced. Farmers that had access to the Ashoka varieties opted to predominantly grow Ashoka varieties rather than hybrids and one of the reasons could be the high cost of purchasing hybrid seed. From personal communication with private-sector scientists, Jharkhand is the state in India with the highest adoption of hybrid rice and somewhat surprisingly some (perhaps most) of the hybrids are targeted at the upland and medium land. The 12% of the land of the Ashoka users that was devoted to MVs was of Suruchi, Rasmi

[4] The survey data are more reliable than the group discussions where farmers can easily misclassify Ashoka varieties as Kalinga III. Only farmers interviewed about what they are growing themselves can say what variety they are actually growing with a high degree of accuracy. Hence, only the survey data are used.

(released 1986) and Lalat (released 1988), the latter two being very old public-sector medium land varieties.

In Hazaribag, Jharkhand, the situation was very different. Perhaps hybrid seed marketing is less effective further from the state capital of Ranchi and no hybrids were grown by the sampled farmers and medium land public-sector varieties were also not grown so nearly all of the land was devoted to landraces. The public sector non-hybrid variety, Vandana (released in 1992) had a total of about 5% of the upland area many years after its release compared with over 40% of the area under the Ashoka varieties only seven years after the first distribution of seed and only a few years after their release. Hence, in this district the comparative success of the Ashoka varieties compared with past alternatives was clear.

In West Bengal adoption levels of the Ashoka varieties were higher (44%) than other MVs (31%) or all upland varieties (21%) even though the Ashoka varieties had been released much more recently. The extent of Ashoka varieties may be underestimated as there is a possible confusion between it and Kalinga III (released in 1983). This was the most commonly grown of the two public-sector upland varieties as only a few farmers grew Annapurna (released in 1968).It was earlier distributed by GVT in the EIRFP from 1995 to 2001 on a large scale and due to phenotypic similarities farmers can easily confuse it with the Ashoka varieties. Many of the reported cases of Kalinga III are very likely to be cases of an Ashoka variety because no seed of the former has been distributed for many years and it is known that farmers replace Kalinga III with Ashoka varieties (Virk and Witcombe, 2007). Among modern varieties (MVs) the most frequent were Lalat and Bullet. Lalat (released in 1988) is an old variety from Orissa recommended for medium lands and has poor grain quality but its seed is widely available. Bullet is an unknown variety perhaps from a private company. Very infrequently some farmers also grew hybrids of private seed companies and the old Orissa varieties Supriya (released in 1973) and Parijat (released in 1976). That the public-sector varieties grown in West Bengal were all from Orissa perhaps reflects the more successful breeding programme in that state.

The high adoption of modern varieties in the uplands in West Bengal is unsurprising as it is possible to grow longer-duration, medium-land varieties in this situation as the duration of the monsoon is longer (Table 28). There are five months of significant rainfall in West Bengal compared with 3 to 4 months in Jharkhand and other more westerly districts (data not shown).

Table 28. Comparative rainfall data for Hazaribag (Jharkhand) and W. Midnapur (W. Bengal) during the rice growing season (June to October)

District	June	July	Aug	Sept	October	Total
Hazaribag†	114	3255	311	299	34	1113
W. Midnapur††	256	277	307	282	211	1333

†Mean over 5 years (2004-08); Hydromet Division, Indian Meterological Department of Government of India

††Mean over 10 years (1996-2005); National Information Centre of Department of Information and Technology, Government of India.

Box 2. Changes in replacement over years.

Varietal replacement of Kalinga III and Vandana over time by the Ashoka varieties cannot be seen in the present analysis as the trends for adoption from 2006 to 2008 were static across the three years. Virk and Witcombe (2007) and Witcombe et al., (2007) reported on an earlier stage of adoption than in this study where the areas under the Ashoka varieties were increasing and the areas under Kalinga III and Vandana (where they were adopted) decreasing.

1. Virk, D.S. & Witcombe J.R. 2007. Trade-offs between on-farm upland rice varietal diversity and highly client-oriented breeding. *Genetic Resources and Crop Evolution* 54:823–835
2. Witcombe, J.R., Devkota, K.P., Virk, D.S., Rawal, K.B. Prasad, S.C., Kumar, V. and Joshi, K.D. (2007) Client-Oriented Breeding and Seed Supply Paper presented in 'Farmer First Revisited: Farmer Participatory Research and Development Twenty Years On' at IDS, Brighton, UK, 12-14 Dec. 2007. http://www.future-agricultures.org/farmerfirst/papers.html#theme3

In the medium lands

In Rajasthan, the Ashoka varieties were grown in medium lands as there was no rice grown in the uplands. Existing varietal diversity was high but restricted to longer-duration landraces adapted to medium lands that, inevitably, had a higher yield potential than the Ashoka varieties. However, after the introduction of seed in 2002 or 2003 farmers had replaced these with the Ashoka varieties for their better grain quality and earlier maturity. They were the only modern varieties that have been adopted (Fig. 9).

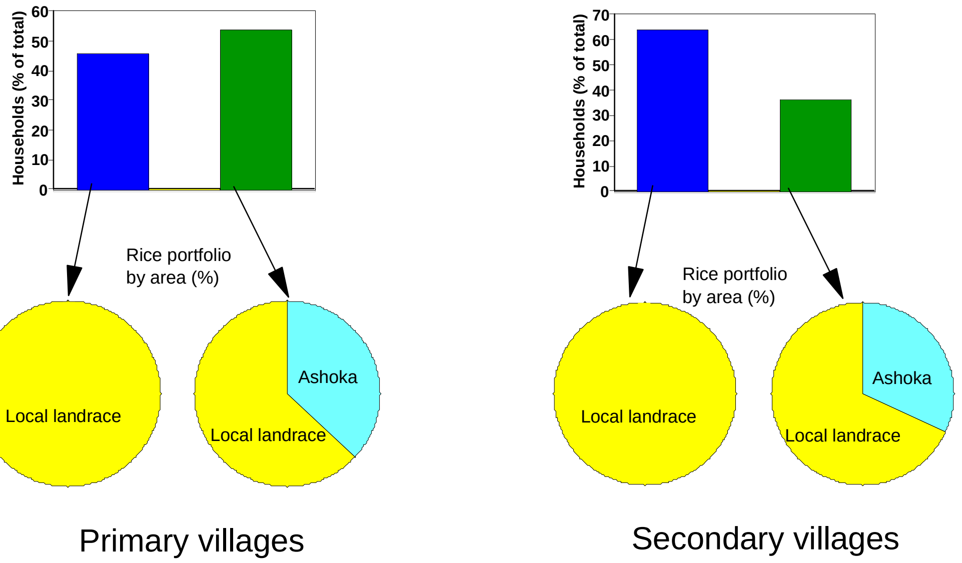

Fig. 9. Proportion of non users (blue) and users (green) in primary and secondary villages in Rajasthan with the proportion of rice landraces that they grow, from a survey of households in 2008.

Orissa was the other state in which the Ashoka varieties were only grown in medium lands. In this state non users of Ashoka grow both MVs (about 58%) and landraces (36%). The Ashoka growers mainly replace MVs as the frequency of these is halved to 29% while landraces are reduced from 36% to 30%. The Ashoka varieties again were much more widely used than other upland MVs. There adoption was 33% compared with a combined total of 8% for released upland varieties Kalinga III and Khandagiri (released in Orissa in 1993).

Environmental impact

The use of the Ashoka varieties does reduce agricultural biodiversity when measured as the number of landraces that are grown (Virk and Witcombe, 2007). However, this analysis is not at a genetic level – only at the level of the names farmers have given to landraces. When genetic dissimilarities are considered, Steele at al. (2009) have shown that the introduction of modern varieties into areas where only landraces are grown can increase genetic diversity even when up to 60% of the land is devoted to the new varieties. Hence, the environmental impact on agro-biodiversity is complex and unresolved.

Other environmental impacts will be largely favourable as the new varieties produce more grain without requiring additional inputs. There are clear benefits from higher yield per cultivated area as the total land and inputs needed per unit of production are reduced. There may be dis-benefits if it encourages farmers to continue to cultivate uplands that could be converted to perennial plantations crops. However, much of the area under the Ashoka varieties was in the medium lands where annual crops are likely to persist.

Benefits to Users

That the majority of the farmers reported increases in food availability was in agreement with the benefits farmers reported from growing the Ashoka varieties where about half of the farmers said they grew the varieties to get more rice (Table 29). Other benefits were also important with better quality grain being ranked highest by most farmers followed by earlier harvest and then better quality straw (Table 29). Farmers preferred the Ashoka varieties for their good grain quality. This was almost certainly because, with the sole exception of Kalinga III, all previously released rice varieties for the direct-seeded uplands were coarse grained as, indeed, are also all the landraces grown in the uplands. It was commonly believed by plant breeders that upland farmers preferred coarse grained varieties. Even if the Ashoka varieties gave no yield increases, which may be the case in the medium lands where longer-duration rice varieties can be grown, farmers traded off the quality of the Ashoka grain and other desirable traits such as earliness and better quality straw against yield.

The relevant question (37) was designed as an open-ended one, rather than one with a fixed list of possible answers, in order to minimise the possibility of unanticipated answers not being given, or not being recorded. There was a list of possible benefits in the questionnaire, to facilitate recording of answers, but enumerators were instructed not to read out this list to respondents.

There was a consistent high ranking of grain quality in all districts (Table 29). The ranking of other benefits differed across districts and the authors have not been able to identify any social or physical factors that could satisfactorily explain these differences other than differences in the local varieties to which the Ashoka varieties were compared. For example, in Banswara district the local varieties may have superior fodder quality resulting in a lower appreciation of this trait in the Ashoka varieties.

.

Respondents could have been influenced by prompting from the enumerators (if they did not follow the spirit of the instruction), who also differed from district to district.

Table 29. Benefits users identified from growing Ashoka varieties across the five districts, shown as an index that has a maximum of 100 that indicates the importance of the reason (= percentage of users giving reason/average rank of the reason), from the household survey .

	Index for					
**District**	**Quality**	**More**	**Fodder in**	**Better**	**Earlier**	**Better**

	rice	rice†	lean period	fodder	harvest	price
Banswara	83	3	0	7	47	3
Mayurbhanj	53	15	14	22	31	13
Ranchi	83	6	10	33	9	1
Hazaribag	87	23	9	32	35	6
W. Midnapur	69	15	3	27	33	10
Overall mean rank	1.3	4.4	4.0	3.1	2.6	4.3
Overall mean response (%)	96	51	27	73	80	28
Overall mean index	74	12	7	24	31	6
Number of users (N)	200	200	200	200	200	200

† the precise question asked was is their increased rice self-sufficiency.

When farmers were asked about changes following the use of the Ashoka varieties in rice grain self sufficiency and rice grain sales none reported a decrease hence all of the results are reported as increases. The majority (83%) of the farmers reported increases in food availability but fewer farmers (18%) reported an increase in grain sales (Table 30). This proportion did not differ greatly across the study districts or between target and non-target farmers.

Farmers who reported an increase in rice self sufficiency said that on average the increase was nearly one month: this represented an average increase of 17% which was highly significant (P<0.001) (Table 31). The differences in increase in rice self sufficiency were not significant among districts, gender of household head and farmer type (target user or non-target user).

The increase in rice grain sales averaged about 150 kg per year (a 46% increase) for those farmers who reported an increase (Table 32). To minimise inter-annual variability farmers were asked to give this information for a ‘normal’ year. There were large differences in the average percentage increase between districts - 37 to 93% - and also in the absolute size of the increase.

Table 30. Proportions of farmers among the Ashoka users who report increases in food grain availability, rice sales, or no increase in either after the adoption of an Ashoka variety, from the household survey.

	Food grain availability increased		Rice grain sales increased		Neither	
District	Yes	%†	Yes	%	Yes	%
Banswara	33	83	6	15	7	18
Hazaribag	38	95	7	18	2	5
Mayurbhanj	32	80	5	13	7	18
Ranchi	32	80	9	23	7	18
W. Midnapur	31	78	8	20	8	20
All target users	89	89	19	19	9	9
All non target users	77	77	16	16	22	22
Overall ‘yes’	166	83	35	18	31	16
Overall ‘no’	34	17	165	83	169	85

†Based on N = 40 households in a district.

Table 31. Period rice lasted for the 83% of the 200 households who reported an increase in rice self sufficiency after using Ashoka varieties.

Period	Mean months rice lasted
Before	5.8

After	6.8
SE difference	0.45
Significance (*P*<0.001)	***
Increase after adoption of Ashoka variety (%)	17
Sample size (N)	166

Table 32. Quantity of rice sold by the 17% of the 200 households who reported an increase in rice grain sales after using Ashoka varieties.

District	No. of responses	Quantity (kg) Before	After	Increase (%)	Paired *t*-value	One tailed *P*
Banswara	6	133	187	41	5.39	0.001
Hazaribag	7	250	371	48	2.44	0.025
Mayurbhanj	5	120	232	93	3.18	0.017
Ranchi	9	361	494	37	5.66	0.000
W. Midnapur	8	694	1010	46	2.60	0.018
Overall	35	341	497	46	4.74	0.000

Farmers were asked if they had ever grown the variety in a *drought* year and all replied that they had not although the qualitative survey did reveal localised instances. Farmers were thus only able to make this comparison for a *typical* growing year and reported a higher grain yield from the Ashoka varieties compared with the popular local[4] ones (a 15% increase, *P*<0.001) (Table 33) and this was reflected in the higher food grain availability reported by the majority of the farmers (Table 30).

The yield advantage of the Ashoka variety over the local variety varied significantly among districts (there was a significant difference for the interaction between varieties and district (*P*<0.001) and the reported advantage varied from 10 to 21%. This probably reflects the differing degrees of abiotic stresses across districts. The yield advantage would probably be greater in a drought year, due to the shorter growing period of the Ashoka varieties. The differences between gender of household head and farmer type (i.e., target recipient or not) were not significant.

Table 33. Opinion of users about grain yield (t ha^{-1}) of Ashoka varieties compared with local varieties in a good growing year by district from the household survey.

District	Grain yield (t ha^{-1}) Ashoka	Local	Increase	Increase over typical local variety (%)
Banswara	1.53	1.26	0.27	21
Hazaribag	1.35	1.19	0.16	13
Mayurbhanj	1.69	1.46	0.23	16
Ranchi	1.50	1.29	0.21	16
W. Midnapur	2.49	2.26	0.23	10
Mean	1.71	1.49	0.22	15
Significance			***	
Sample size (N)	181	181		

*** *P*<0.001.

[4] Local variety was defined as a popular local variety Therefore, there was no single local variety across farmers within and across states.

Characteristics of Users and non-Users

Poverty status

The household characteristics of the users and non-users were compared (Tables 34 and 35). There was no significant difference between users and non-users of Ashoka varieties for any of the six individual traits that were used to compute the poverty index (indicated by *** in Tables34, 35). Among the other individual socio-economic indicators only a few minor differences were found. There were no significant differences for any socio-economic variable relating to natural and physical capital; and there were three variables for social characteristics where there were small but significant differences between users and non-users. Users had:

- a higher average number of household members who regularly work on farm
- a higher proportion of households having a card for being below the poverty line (and this was verified by seeing the card) and
- a lower representation of scheduled tribes (and hence a higher representation of other backward classes).

However, these differences were too small to suggest that social factors are a major factor in the upland rice innovation system. They contrast with the importance of, for example, rainfall in the type of modern variety that is adopted in the uplands (Table 28).

Table 34. Socio-economic indicators relating to gender, household size, work and migration and ethnicity, of users and non users of the Ashoka varieties from a survey in 2008.

	**Category**			
**Socio-economic indicator (people related)**	**User**	**Non user**	**Overall**	**SED††**
Full time job holders in the family (mean number)***	0.14	0.18	0.15	0.05
Unskilled seasonal migration (mean months of husband and wife) †***	3.7	2.4	3.4	0.7
Female headed households (%)	8	7	7	3.2
Household size (capita)	4.9	4.6	4.8	0.3
Full time job holders (mean number)	0.14	0.18	0.15	0.05
Any migration in the family (%)	31	31	31	6
Persons migrating (mean number)	1.5	1.6	1.5	0.3
Farm workers in household (number)	**2.5**	**2.0**	**2.3**	**0.2**
Scheduled tribes (%)	**63**	**74**	**66**	**6**
Scheduled caste (%)	8	9	8	4
Other backward class (%)	**30**	**16**	**25**	**5**
Other caste (%)	0	1	1	1
Below poverty line (BPL) (%)	**68**	**54**	**63**	**6**
NREGA§ card holder (%)	82	76	80	5
Worked last year on NREGA Scheme (%)	67	59	64	6
Sample size	200	96	296	

*** Element of poverty index.
†Mean months of migrating husband and wife head of the household
††Standard error of the difference between the means. Significant ones are indicated in bold font.
§National Rural Employment Guarantee Act, NREGA

Table 35. Socio-economic indicators relating to natural and physical resources, of users and non users of the Ashoka varieties from a survey in 2008.

Socio-economic indicator (Natural and physical capital related resources)	User	Non user	Overall	SED†
Roof indicator***	0.76	0.81	0.78	0.06
Thatched roof (%)	25	26	25	5
Tiled roof (%)	75	72	74	6
Concrete roof (%)	1	2	1	2
Livestock equivalents (mean of index)***	2.3	2.5	2.4	0.3
Tractor ownership***	0	0	0	0
Food grain production per capita (kg/person/year)***	347	313	336	44
Months of grain self sufficiency (no)	3.0	3.2	3.1	0.4
Total grain production per household (kg/ year)	1540	1280	1455	199
Total land (ha)	1.41	1.62	1.48	0.37
Total upland (ha)	0.60	0.78	0.66	0.20
Total medium land (ha)	0.47	0.52	0.48	0.12
Total lowland (ha)	0.35	0.32	0.34	0.08
Proportion having own animal for traction (%)	83	75	80	5
Sample size	200	96	296	

*** Element of poverty index.
†Standard error of the difference between the means. Significant ones are indicated in bold font.

The poverty indexes for the users and non users were not significantly different (Fig. 10). Although the maximum possible poverty index was 25the highest was 14 and 99% of the 296 respondents were in the poor class when defined as having a poverty score of less than 12.5. The only two cases where the poverty score was above 12.5 were for users. One was in Jainagar primary village in W. Midnapur where the household had a concrete roof, high per capita food availability, one full time job holder and no migration. The second case was in Hazaribag village Rajhar where the household had 4 full time job holders and so had a score of 6 for this one component.

As was expected from the non-significant differences in the six components (Tables 34 and 35), the index that resulted from the sum of the scores of these components also showed a non-significant difference between users and non users.

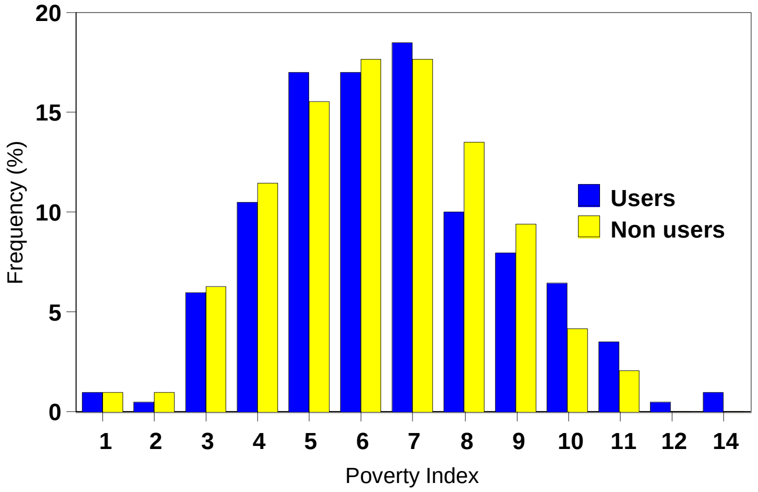

Fig. 10. Distribution of poverty scores (index) for the users and non-users.

The high levels of poverty was in accordance with the targeting of villages by GVT in the DFID-supported EIRFP and WIRFP bilateral projects. The villages were chosen to have a

high proportion of indigenous tribal people and comparatively poor access to markets and other institutional support. Furthermore, the sampled states and districts are among the poorest in India.

There were significant differences among the districts for all the individual component indexes and the total index (Table 36). The surveyed households in Mayurbhanj district, Orissa, were significantly the poorest followed by the households in Banswara district, Rajasthan.

Table 36. Mean values for poverty indexes for districts which were significant (*P*<0.001) for all components by analysis of variance

**Index**	**Banswara**	**Hazaribag**	**Mayurbhanj**	**Ranchi**	**W. Midnapur**	**Lsd (5%)**
Overall	6.1	7.2	5.3	6.6	7.0	0.81
Roof type	1.0	0.9	0.3	1.0	0.7	0.17
Live stock units	1.6	2.1	1.3	1.4	1.5	0.36
Food production	0.9	0.9	0.6	1.3	1.6	0.45
Job holders	0.1	0.5	0.2	0.5	0.3	0.30
Tractor ownership	0	0	0	0	0	0
Migration score†	2.5	2.8	2.9	2.5	2.8	0.27

† Migration score of unskilled labour when husband and wife migrate (0= wife migrates, 1= husband migrates, 3= none migrates)

REFERENCES

Steele, K.A., Gyawali, S., Joshi, K.D., Shrestha, P., Sthapit, B.R. & Witcombe, J.R. 2009. Has the introduction of modern rice varieties changed rice genetic diversity in a high-altitude region of Nepal? *Field Crops Research* 113: 24-30.

Virk, D.S. & Witcombe J.R. (2007). Trade-offs between on-farm upland rice varietal diversity and highly client-oriented breeding. *Genetic Resources and Crop Evolution* 54:823–835.

Virk, D.S., Singh, D.N., Prasad, S.C., Gangwar, J.S. and Witcombe, J.R. (2003). Collaborative and consultative participatory plant breeding of rice for the rainfed uplands of eastern India. *Euphytica* 132: 95-108.

Witcombe, J.R, Petre, R., Jones, S. and Joshi, A. (1999). Farmer participatory crop improvement. IV. The spread and impact of a rice variety identified by participatory varietal selection. *Experimental Agriculture* 35:471-487.

Witcombe, J.R., Joshi, K.D., Gyawali, S., Musa, A.A., Johansen, C., Virk, D.S. and Sthapit, B.R. 2005. Participatory Plant Breeding is better described as highly client-oriented plant breeding. I. Four indicators of client orientation in plant breeding. *Experimental Agriculture* 41:1-21.

Witcombe, J.R., Virk, D.S., 2001. Number of crosses and population size for participatory and classical plant breeding. *Euphytica* 122, 451-462.

ANNEXES

Annex B1 Group Discussion in primary villages

Group discussions (GDs) were held in all 20 primary villages that included:

- Procuring of district wall map
- Procuring of block map of selected block/s where P villages were located
- Informing the villagers a day before that a team would come for discussions
- Explaining the purpose of visit in the beginning without raising expectations.
- GDs included adopters from all the wealth categories, ethnic groups and gender.

(i) Primary information about the village: In the GD primary statistics of the village in terms of total land, cultivated land and classification of land area by ecology and crop profiling was done. This way the total rice area by ecology was found in the village. This process was facilitated by using a Revenue Map of the village procured before the GD.

(ii) *Identification of rice adopters:* The survey team compiled a list of all households (and their ethnicity/caste) involved in rice production in the uplands, medium and low lands. The list also gave the identity of Ashoka rice adopters and non-adopters and those who were target Ashoka seed recipients from GVT in the earlier years or were non-target Ashoka adopters following receipt of seed from secondary sources.

This list was either prepared in the GDs or procured from the village revenue official (Patwari).

(iii) *Varietal composition of rice:* Varietal spectrum of rice in the uplands and medium land was prepared and apportioned in terms of areas of rice allocated to them and the number of households growing them. This led to the preparation of two pie charts or Tables constructed on the basis of areas and number of households growing different varieties of rice. This information was helpful in finding out the relative area of Ashoka varieties and proportion of households growing them in the village.

(iv) *Reasons for growing or not growing Ashoka:* Reasons as to why farmers prefer to grow Ashoka or why the non-adopters or droppers did not adopt them were asked in the group discussions and a summary prepared.

(v) Dissemination of Ashoka from primary villages: The survey team procured a block-level map beforehand and located on it, during the discussion, the villages mentioned by the group members.

- Mark the S villages on block or district map where people indicate that rice seed has travelled from the P village.
- Indicate on district/block map to show major physical barriers between P and S villages with Ashoka (mountain, river, dense forest).

Groups in the primary villages were asked

- To which village, if any, they had distributed Ashoka seed;
- If they know of any villages nearby in which Ashoka is grown. If so prepared a list of villages where Ashoka was grown and indicated on the block map;
- To which villages they are well connected in relation to various factors, including:

1. Kinship
2. Ethnic groups
3. Historical factors
4. Ease and speed of access (road access)

5. Any other community activities in the connected, e.A., annual festival ground, religious activity, Panchayat activity, place for sports, school, health centre, veterinary centre, weekly market etc.

- Why Ashoka was grown in some S villages and not in others? Explored:
    1. If there was no upland/medium land in some villages that did not grow Ashoka
    2. If there was no rice cultivation at all
    3. If farmers preferred other cash crops in *kharif* over Ashoka rice

- Who are the farmers in S villages with Ashoka and without Ashoka? (Absentee farmers, tribal, CS, ST etc.)

Annex B2 RFA Household Survey Questionnaire: Ashoka Rice Growers

**Interview Information**		
1	Enumerator	Name …………………………………………………………
2	Date of survey	……..Day………..Month ……….Year
3	Tola Name	…………………………………………………………
4	Village	
5	Block	…………………………………………………………
6	District	…………………………………………………………
7	State	…………………………………………………………

**Household Profile**		
8	Female (F) or male (M)–headed household?	(F/M)
9	Name of respondent	………………………………… tick M / F
10	Family status of respondent?<br><br>**(PLEASE CIRCLE CORRECT ANSWER)**	a. male HH head<br>b. wife of HH head<br>c. son of HH head<br>d. daughter of HH head<br>e. other – please specify
11	BPL status	YES___NO___…………………………………
12	Type of roof<br><br>**(PLEASE CIRCLE CORRECT ANSWER)**	a. Thatched<br>b. Tiles or Tin<br>c. Pacca
13	Livestock Ownership<br><br>**(GIVE THE NUMBER OF ADULT ANIMALS)**	a. Cow ……………….<br>b. Buffalo……………<br>c. Goat……………….<br>d. Pig…………………<br>e. Poultry ……………<br>f. Others, if any………
14	What type of traction do you use?<br><br>**(PLEASE CIRCLE CORRECT ANSWER)**	a. Own animal<br>b. Rented animal<br>c. Own tractor<br>d. Rented tractor<br>e. Other-please name ………………………..
15	For how many months in a normal year do your home-grown cereals (total of rice, maize, wheat etc.) feed the household?	………………………months

**Household composition**		
16	Circle correct answer	ST/SC/OBC/Other casts/ other tribe………
16 a	Specify name of caste or tribe	……………………………….
17	No. of adults (18 years old and above)	male...... female…….
18	No of children aged 10 to 17	Male……female….....
19	No of children aged under 10	Male……female….....
20	No. of HH members who are full time job holder and contribute income to HH	…………………………………………………
21	No. of HH members who regularly work	…………………………………………………

	on farm	
22	Do you have a NREGA Job Card?	YES___ NO____
23	Did you benefit from NREGA during the last year?	YES___ NO____
24	If YES, in which months	……………………………………………….

Farm Profile:
NOTE: PLEASE GIVE THE LOCAL UNIT-------------------------------e.g., BIGHA/ KATTHA/DECIMAL) AND ITS CONVERSION FACTOR INTO ACRE-------------------------------------.

RECORD ANSWERS TO QUESTIONS ABOUT LAND AREA IN ACRES IN THE WHOLE QUESTIONNAIRE.

Land holding

**Land owned**	**Land type (Acres)**		
	**Upland**	**Mid land**	**Lowland**
Total land owned by respondent's household			
Own land cultivated by respondent's household			
Own irrigated land cultivated by respondent's household			
Own land rented out by respondent's household			
Own land shared out by respondent's household			

26.	If you have irrigation what is the source of water?<br><br>**(PLEASE CIRCLE CORRECT ANSWER)**	canal<br>tube well<br>open well<br>pond<br>river<br>other ……………………………..

Does your household cultivate any land other than your own land last year?
YES____NO____

If YES, please describe:

**Land cultivated**	**Land type (acres)**		
	**Upland**	**Mid land**	**Lowland**
Land rented in by respondent's household			
Land shared in by respondent's household			

27. In the last year's *Kharif* (2007) what food grain crops did you grow and how much did you harvest?

Crop	Please tick against each crop grown	Kg harvested
1. Rice		
2. Maize		
3. Groundnut		
4. Other (please name)		
5.		
6.		
7.		
8.		
9.		

28. In the last *Rabi*/summer season (2007-2008) what food grain crops did you grow and how much did you harvest.

Crop	Please tick against each crop grown	Kg harvested
Rabi		
Wheat		
Chickpea		
Maize		
Lentil		
Linseed		
Other (please name)		
Total		
**Summer**		
Rice		
Any other		

29. Rice area under each land type by season

	Type of land (Acres only unit allowed)		
	Upland	Mid land	Lowland
Total rice area in *Kharif*			
Total rice area in summer			

30. Year Ashoka (BVD 109, BVD 110) variety was first grown

Year of first introduction	Source†	Source: inside / out side of village (CIRCLE ONE)
		Inside / Outside

†GVT=GVT; Market =M; Neighbour/Friend =N/F; Relative=R; DoA=DoA; Other NGO=NGO

31. Rice varieties grown in the *Kharif* season

**Varieties**	**Upland (acres)**			**Mid land (acres)**		
	**2008**	**2007**	**2006**	**2008**	**2007**	**2006**
**Ashoka (BVD 109,110)**						
**MV 1………………**						
**MV 2………………**						
**MV 3………………**						
**MV 4………………**						
**MV 5………………**						
**Land races**						

Note: MV – Modern Varieties (PLEASE WRITE THE NAME OF THE VARIETY)

32. Rice varieties grown in the summer season

**Varieties**	**Upland (acres)**			**Mid land (acres)**		
	**2008**	**2007**	**2006**	**2008**	**2007**	**2006**
**Ashoka (BVD 109,110)**						
**MV 1………………**						
**MV 2………………**						
**MV 3………………**						
**MV 4………………**						
**MV 5………………**						
**Land races**						

Note: MV – Modern Varieties (PLEASE WRITE THE NAME OF THE VARIETY)

33. From the last year harvest of (2007) did your household supply Ashoka seed? YES __ NO __

a. If YES, PLEASE COMPLETE TABLE BELOW, ONE ROW AT A TIME.

To whom did you supply seed?£	How far away? (km)	How much did you supply? (kg)	Type of transaction†

£ Farmer; Traders; Relatives; Friends

†1. Exchange; 2. Gift; 3. Sold; 4. Seed Bank; 5. Other.

36 Did anyone in your household migrate in the last year (2007-2008)? YES __ NO __

a. If YES, how many people? ………………………………….
b. Who are they? **(PLEASE CIRCLE CORRECT ANSWER)**

1. Male HH head
2. Female HH head
3. Wife of HH head
4. Husband of HH head
5. Son of HH head
6. Daughter of HH head
7. Other (please name) ………

c. Record the migration details in the following table (only for HH head and where appropriate their spouse)

Hints for enumerator to get information for question (PLEASE ASK THE FOLLOWING QUESTIONS IN SEQUENCE):

- Who migrated;
- How many times in the year;
- When was the first trip;
- First trip - for how long (weeks);
- To do what kind of work?
- When was the second trip;
- Second trip - for how long (weeks);

(PLEASE RECORD THE MONTHS OR ITS FRACTION ON MIGRATION)

Who migrates?	Jul	A	S	O	N	D	Jan	F	M	A	May	Jun	Total Months

Male: (HH head)													
Type of work *<br>(US, SS or S)													
Female (HH head's wife)													
Type of work*<br>(US, SS or S)													

* Work should be classified into 1 of 3 categories: unskilled (US), semi-skilled (SS) and skilled (S).
Enter the relevant abbreviation in the appropriate cells.
Unskilled work includes any kind of manual labour (e.g. construction work, farm labour). Examples of semi-skilled work are: masonry, making/repairing shoes or driving a vehicle.
Skilled work requires a certain level of literacy and numeracy (e.g. managing a tea shop, doing a desk job in an office).

IMPACT ON LIVELIHOODS

37. What are the benefits your household gains from growing Ashoka rice?

PLEASE DO NOT READ OUT LIST , ONLY TICK IF RESPONDENT MENTIONS.

Benefits	Tick if mentioned	Rank - only if ticked. (1 = most important)
Source of cash		
Better quality of rice		
Increased rice self sufficiency		
Better health		
Reduced or avoided migration		
Fodder availability in lean period		
Better quality fodder		
Earlier harvest		
Better price		
Food in hunger gap		
More rice in drought years		
Less weeding required		
Other (please name)		

Drought

Note: CIRCLE THE ANSWER

38. Have you grown Ashoka varieties in any year when there was a drought? (Yes/No)

39. If yes, what year was this?……………..

40. If yes, what land? (upland/ medium)

RECORD ANSWERS TO THE FOLLOWING FOUR QUESTIONS IN TABLE BELOW

41. Give an estimate of the yield of the Ashoka variety in that drought year.
42. Give an estimate of the yield of a popular local variety in that drought year?
43. Give an estimate of the yield of the same popular local variety in a good year?
44. Give an estimate of the yields of the Ashoka variety in a good year.

	Ashoka<br>kg per acre	Local (name…………….)<br>kg per acre
Good year		
Drought year		

Rice self-sufficiency

45. Has the adoption of Ashoka varieties changed your food grain availability? (Yes/No)

IF YES, ASK

46. For how many months did the rice from your harvest last in a normal year before Ashoka? ………………..
47. For how many months does the rice from your harvest last in a normal year after Ashoka? ………………..

Rice grain sales (income)

48. Has the adoption of Ashoka varieties changed your grain sales? (Yes/No)

IF YES, ASK

49. How many kg of rice grain you sold in a normal year after adoption of Ashoka varieties? ---------------(kg)
50. How many kg of rice grain you sold in a normal year before adoption of Ashoka varieties? ---------------(kg)
51. To whom and for what price did you sell rice grain from the 2007 harvest:

THIS INFORMATION IS TO BE OBTAINED FOR THE YEAR 2007 ONLY.

To whom	Ashoka (Rs/kg)	Local type (e.g. Gora) (Rs/ kg)	Modern varieties grown (Rs./Kg.)
		Variety/price	Variety/price
Visiting trader			
Local trader			
Local hat			
Market (Mandi)			
Others			

52. **If answer to question 11 is YES please ask respondent to produce BPL card and record here whether (s)he did produce it. YES __ NO __**

Annex B3 RFA Household Survey Questionnaire: Non-Ashoka Rice Growers

<u>Interview Information</u>

1	Enumerator Name	………………..……………………………………………
2	Date of survey	………Day………..Month ……….Year
3	Village	………………..……………………………………………
4	Block	………………..……………………………………………
5	District	………………..……………………………………………
6	State	………………..……………………………………………

<u>Household Profile</u>

7 Female or male –headed household? (F/M)

8 Name of respondent ………………………………… tick M / F

9 Family status of respondent?

(PLEASE CIRCLE CORRECT ANSWER)

f. male HH head
g. wife of HH head
h. son of HH head
i. daughter of HH head
j. other – please specify

10 BPL status YES___NO___…………………………………

11 *Tola* name (hamlet name) ……………….……………………….………………

12 Type of roof

(PLEASE CIRCLE CORRECT ANSWER)

a. Thatched
b. Tiles or Tin
c. *Pacca*

13 Livestock Ownership

(GIVE THE NUMBER OF ADULT ANIMALS)

g. Cow ………………
h. Buffalo……………
i. Goat………………
j. Pig…………………
k. Poultry ……………
l. Others if any…………..

14 What type of traction do you use?

(PLEASE CIRCLE CORRECT ANSWER)

f. Own animal
g. Rented animal
h. Own tractor
i. Rented tractor
j. Other-please name ………………………..

15 For how many months in a normal year do your home-grown cereals (total of rice, maize, wheat etc.) feed the household? ……………………months

**<u>Household composition</u>**		
16	Caste/ethnicity	ST / SC / OBC / Other castes/ other tribe………
16a	Specify name of caste or tribe	…………………………..
17	No. of adults (18 years old and above)	male...... female…….
18	No of children aged 10 to 17	male……female….....
19	No of children aged under 10	male……female….....
20	No. of HH members who are full time job holder and contribute income to HH	………………………………………………
21	No. of HH members who regularly work on	………………………………………………

	farm	
22	Do you have a NREGA Job Card?	YES___ NO____
23	Did you benefit from NREGA during the last year?	YES___ NO____
24	If YES, in which months	…………………………………………………

Farm Profile:

25. Land holding (PLEASE GIVE THE LOCAL UNIT-------------------------------e.g., BIGHA/ KATTHA/DECIMAL) AND ITS CONVERSION FACTOR INTO ACRE---------------------.

THIS APPLIES TO THE WHOLE QUESTIONNAIRE.

**Land owned**	**Land type**		
	**Upland**	**Mid land**	**Lowland**
Total land owned by respondent's household			
Own Land cultivated by respondent's household			
Own Irrigated land cultivated by respondent's household			
Own land rented out by respondent's household			
Own land shared out by respondent's household			

26 If you have irrigation what is the source of water?<br><br>**(PLEASE CIRCLE CORRECT ANSWER)**	a. Canal<br>b. tube well<br>c. open well<br>d. pond<br>e. river<br>f. other ……………………………..

27. Does your household cultivate any land other than your own land last year? YES____NO____

28. If YES, please describe:

**Land cultivated**	**Land type**		
	**Upland**	**Mid land**	**Lowland**
Land rented in by respondent's household			
Land shared in by respondent's household			

29. In the last year's *Kharif* (2007) what food grain crops did you grow and how much did you harvest?

Crop	Please tick against each crop grown	Kg harvested
1. Rice		
2. Maize		
3. Groundnut		

4. Other (please name)		
5.		
6.		
7.		
8.		
9.		

30. In the last *Rabi* and summer season (2007-2008) what food grain crops did you grow and how much did you harvest?

Crop	Please tick against each crop grown	Kg harvested
Rabi		
Wheat		
Chickpea		
Maize		
Lentil		
Linseed		
Other (please name)		
Total		
**Summer**		
Rice		
Any other (please name)		

31. Rice area under each land type by season

	Type of land (Acres only unit allowed)		
	Upland	Mid land	Lowland
Total rice area in *Kharif*			
Total rice area in summer			

32. For *Kharif* season

**Varieties**	**Upland**			**Mid land**		
	**2008**	**2007**	**2006**	**2008**	**2007**	**2006**
**Land races**						
**MV 1………………**						
**MV 2………………**						
**MV 3………………**						
**MV 4………………**						
**MV 5………………**						

Note: MV – Modern Varieties (PLEASE WRITE THE NAME OF THE VARIETY)

33. For summer season

**Varieties**	**Upland**			**Mid land**		
	**2008**	**2007**	**2006**	**2008**	**2007**	**2006**
**Land races**						
**MV 1………………**						

**MV 2………………**						
**MV 3………………**						
**MV 4………………**						
**MV 5………………**						

Note: MV – Modern Varieties (PLEASE WRITE THE NAME OF THE VARIETY)

34. Do you know about Ashoka rice varieties? YES __ NO __
    IF THE ANSWER IS NO THEN GO TO QUESTION No. 38 (MIGRATION)
    IFTHE ANSWER IS YES THEN ASK QUESTION 35 (NEXT QUESTION)
35. Have you ever grown Ashoka rice varieties? YES __ NO __
    IF YES ASK QUESTION 36 IF NO ASK QUESTION 37
36. Why are you not growing now? **FILL IN TABLE BELOW**
37. You know about Ashoka then what are the reasons of not growing Ashoka? **FILL IN TABLE BELOW**

PLEASE DO NOT READ OUT LIST ONLY TICK IF RESPONDENT MENTIONS.

Reason	Tick if respondent mentions any reasons	Rank – only reasons if ticked. (1 = most important)
Not the best upland variety for my land		
Can not obtain seed		
Not cultivating upland		
Lodging		
Others (specify)………………..		

40. Did anyone in your household migrate in the last year (2007-2008)? YES __ NO __

    a. If YES, how many people? …………………………………..
    b. Who are they? **(PLEASE CIRCLE CORRECT ANSWER)**

1. Male HH head
2. Female HH head
3. Wife of HH head
4. Husband of HH head
5. Son of HH head
6. Daughter of HH head
7. Other (please name) ………

c. Record the migration details in the following table (only for HH head and where appropriate their spouse)

Hints for enumerator to get information for question (PLEASE ASK THE FOLLOWING QUESTIONS IN SEQUENCE):

- Who migrated;
- How many times in the year;
- When was the first trip;
- First trip - for how long (weeks);
- To do what kind of work?
- When was the second trip;
- Second trip - for how long (weeks);

(PLEASE RECORD THE MONTHS OR ITS FRACTION ON MIGRATION)

Who migrates?	Jul	A	S	O	N	D	Jan	F	M	A	May	Jun	Total Months
Male: (HH head)													
Type of work * (US, SS or S)													
Female (HH head's wife)													
Type of work* (US, SS or S)													

* Work should be classified into 1 of 3 categories: unskilled (US), semi-skilled (SS) and skilled (S).
Enter the relevant abbreviation in the appropriate cells.
Unskilled work includes any kind of manual labour (e.g. construction work, farm labour). Examples of semi-skilled work are: masonry, making/repairing shoes or driving a vehicle.
Skilled work requires a certain level of literacy and numeracy (e.g. managing a tea shop, doing a desk job in an office).

39. If answer to question 10 is **YES** please ask respondent to produce BPL card and record here whether (s)he did produce it. YES __ NO __

Annex B4 Poverty index

(Methodology for computing poverty index for India Case Study as proposed in the 25-29 January 2009 meeting at Dhulikhel, Nepal)

The purpose of the index

The poverty index was constructed to enable the study to distinguish wealthier households from the rest of the households among those surveyed. Its use will allow the study to assess the extent to what projects have worked with the poor in the areas of intervention.
The poverty index does not attempt to place households in relation to the poverty line established by the government of the State or country where the intervention took place. The data demands for such exercise are too big in relation to the resources and information needs of the projects.

Development of the index

These indicators fall in line with the categories of indicators used by the Consultative Group to Assist the Poorest (CGAP) Poverty Assessment Tool (cited in Falkingham, J. and Ceema N., 2002), developed by the CGAP of the World Bank in collaboration with the International Food Policy Research Institute.

The indicators

The indicators selected for the poverty index are:

- Livestock units
- Total quantity of all food grains produced in the season 07-08 per capita
- Roof type
- Number of jobholders in household who provide income
- Ownership of a tractor
- Extent of unskilled labour migration

Livestock units This was calculated by computing a weighted sum of all livestock owned by the household. The weights of the different types of animals were as follows:

Type of animal	Weight
Cows, Buffaloes, horses, donkeys	1
Goats, Sheep	0.1
Poultry	0.01
Pigeons	0.005

The number of animals owned of each type was multiplied by the corresponding weight and the products added up to obtain the “Livestock Units”. Thresholds for this indicator index were derived from consultation of secondary sources (NSS Report No. 493(59/18.1/1), Maltsoglou, I and Taniguchi, K., 2004) and consultations with key informants from the partner organisations participating in the study.

Food production per capita This was the total quantity of all food grains produced in the season 2007-2008 per capita and included the weight (kg) of grains (cereals and legumes) produced in the 2007-2008 season. The indicator included grain that is produced for consumption as well as that that is produced for sale.

For the calculation of the indicator, the total production was divided by the number of adult equivalents per household. Adult equivalents per household were calculated as a weighted sum suing the following weights: Adults = 1 ; 10 – 17 years = 1; children under 10 = 0.1. The

contribution of this indicator to the overall index was based on a scale that increases in accordance to the increase of food production. At the lower end of the scale are those households with production less than 180 kg grain per capita, roughly equivalent to 0.5 kg per day. Households with more than 730 kg per capita (roughly 2 kg per day per capita) are at the higher end of the scale.

Other indicators
The other indicators were derived directly from the corresponding questions included in the questionnaire and did not require specific calculations.

Scores for the poverty indexes of India

	Score							
**Indicator**	**-1**	**0**	**1**	**2**	**3**	**4**	**5**	**6**
Livestock units		<1	1-<3	3-<5	5-<10	>=10		
Food production per capita		<180	180-<365	365-<730		>=730		
Roof material		Thatch	Tile	Tin	Pucca/ concrete			
Jobholders in the family		No job		1 job		2 jobs		3 or more
Tractor ownership		No tractor					Own tractor	
Seasonal unskilled labour migration		Wife migrates	Husband migrates		Neither migrates			

Falkingham, J. and Ceema N., 2002. “Measuring health and poverty: a review of approaches to identifying the poor. Issue Paper. DFID Health Systems Resource Centre.

Maltsoglou, I and Taniguchi, K., 2004 “Poverty, livestock and household typologies in Nepal”. Pro-poor livestock Policy Initiative. Working Paper No 13. FAO.

NSS Report No. 493 (59/18.1/1), 2006. “Livestock Ownership Across Operational Land Holding Classes in India, 2002-03”. National Sample Survey Organisation, Ministry of Statistics & Programme Implementation, Government of India, January 2006.

MIL RA Cluster Study, 2008. Quarterly Report April-June 2008. Internal report.

PART C: REPORT ON THE QUALITATIVE STUDY

Prepared by
Malika Basu, Marlene Buchy & Aniruddha Dey

INTRODUCTION

Since the mid 1990s, the DFID Plant Science Research Programme has supported participatory plant breading research. Collaborative work between an NGO the Gramin Vikas Trust (GVT), Birsa Agriculture University in Ranchi and then Centre of Arid Zones Studies-Natural Resources (now CAZS-NR) in the UK, produced in 2001 two varieties of rice, Ashoka 200F and Ashoka 228. These very released in 2003 and notified in 2005 (See Introduction to Part B). These two varieties are drought resistant, tall and early maturing, produce good grain and fodder yield and are suited for Upland agriculture (Bourai *et al* 2002). Seeds were distributed to villagers in Orissa, Jharkhand and west Bengal, and later on in Rajasthan, by GVT field teams in 2002 (19 tonnes) and 2003 (34 tonnes) (Bourai *et al* 2002). In 2003 GVT also distributed seeds in Jharkhand to the Government and the Birsa Agricultural University, to 6 NGOs and 1 private seed grower. In Orissa the seeds were distributed to the department of Agriculture in 4 locations and to at least 4 NGOs (Virk *et al.* 2003). The farmers received seeds for testing in PVS trials along with other varieties. This was a research project which explains the relative small quantity of seeds distributed.

This report presents the results of the qualitative survey conducted in 5 districts in the 4 states of Rajasthan, West Bengal, Orissa and Jharkhand between September 2008 and March 2009. Originally the qualitative survey, labelled as ‘Method 4’ in the overall case study methodology of the Monitoring and Learning component of the Research Into Use programme, was conceived as a tool to complement the analysis of 3 structured methods applied by the case study team. The overall aim of the study is to document and explain impacts of the introduction of Ashoka rice varieties in the districts on livelihoods and agriculture practices; and to the extent possible get a measure of the impacts for the poorest sections of the community. It does not seek to measure the extent of use of the varieties in the districts.

METHODOLOGY

It was originally envisaged that this study would be conducted by one independent academic researcher, as part of a wider contract. The first person (M Basu) withdrew her services after conducting field work in 3 districts (Banswara in Rajasthan, Mayurbhanj (Baripada block in Orissa) and W. Midnapur (Jhargram block in West Bengal) and the study was then completed by another person (A Dey) in 2 other districts (Hazaribagh (Katkamsandi block in Jharkand) and Ranchi (Bero block). The original plan was that the qualitative field work would follow the structured survey so that the latter would focus on issues identified in the former. It was envisaged that during the first week of qualitative work, enumerators who had been involved in the structured survey work would be able to share with the qualitative researchers their insights and knowledge of possible emerging issues as a result of their survey work. However in practice this did not happen as the timing was tight and by the time the qualitative survey started no analysis of the quantitative data had started. In September 2008 M. Buchy joined M. Basu for initial field work in the district of Banswara, Rajasthan in

order to develop and test the best approach for the qualitative survey. A discussion in Banswara with GVT staff revealed that little information to guide the qualitative work was available and that only one GVT staff member was allocated to accompany the team to the field to facilitate the logistics. This meant that the qualitative work had to start with 2 days of field work dedicated to generic discussions with villagers in order for the researchers to familiarise themselves with the context and the issues and hopefully to try to see emerging patterns in order to develop the methodology to follow in all the subsequent districts.

Based on the work in Banswara we identified a list of core areas to investigate in each of the villages (see box 1).

Box 1: Core topics for the group discussions and interviews

a. The proportion of Ashoka acreage in relation to suitable land and other crops and possible yields and how the production was used;
b. Farmers' choices and strategies as well as other aspects like the use of irrigation, quality of land, market value of crops, impact of the job card scheme (National Rural Employment Guarantee Scheme) and changes in labour from a gender perspective;
c. A discussion on changes 'after Ashoka'. Are farmer able to clearly identify the impact Ashoka has had on their lives and livelihoods? is there extra cash being generated and what do households do with this money? Is there a variation between categories of farmers? (ie do small farmers and medium farmers have the same needs or priorities in spending their extra cash?) for eg. if more money is spent on education who in the family is the money spent on? Does it mean an extension of schooling? Is the money making a difference between sending a child to school or not?
d. Differential impact: ie is there a social differentiation influencing the beneficial impact, and also do the improved seeds encourage social differentiation? Are some categories of farmers benefitting more?
e. A discussion on the spread of the seeds: accessibility of seeds, dissemination, but also information available;
f. A discussion on possible organisational changes in the village traceable to Ashoka, including community groups, increased access to services

These questions were asked in all the visited villages, to farmers who had received seeds initially through the project as well as to farmers who had decided to try the crop of their own initiative. We were also interested to meet farmers (if any) who had stopped growing Ashoka. In all the villages we wanted to interview women to get their views but also to try to assess whether women had seen specific impacts on their work load or the household income following the introduction of Ashoka.

Three methods were used:

- The group discussion, including farmers growing Ashoka varieties, those who might have grown it and stopped and those who might want to grow it in the future.
- In depth interviews of 'interesting' individual households if any. These typically could be households having experienced dramatic changes in their livelihoods or farming systems or households that have tested the seeds for a few years and decided to stop;
- Cross checking some data with other actors such as: primary health care nurse or health visitor, trader, extension worker, nearby research station.

In each district all the 'primary' villages surveyed during the structured work, as well as a selection of 'secondary' villages have been visited. A primary village is a village where GVT distributed the seeds originally and secondary villages are villages where farmers who grow Ashoka acquired the seeds through personal means (though seed swaps, barter, family and

friends) from someone in a primary village. Tables 1 and 2 give details of the number of group and individual interviews conducted. Organising women meetings was not always possible or easy, but we made sure that at least in one of the primary villages in each district (except Jhargram) only women growers would be called to the meeting.

Table 1: Summary of Focus groups discussions and interviews conducted by A. Dey

Villages	Primary village	Secondary village	Tertiary village	Mixed Groups	FGD Men	FGD women	In-depth interview
**In Hazaribagh District**							
**Kud**		X			1 (10)	1 (13)	1 (Woman)
**Bajha**	X						1 (Man)
**Uridiri**		X					1 (Woman)
**Lakhnu**			X				1 (Man)
**Hutkona**	X				1 (8)	1 (8)	1 (Woman)
**Kherika**	X			1 (7+7)		1 (7)	4 (Men)
**In Ranchi District**							
**Jaria***				1(12)			1 (Woman)
**Muramu**	X				1(22)	1(12)	2 (Men)
**Nehalu**	X			1(11+5)			
**Hulsi**	X			1 (10+2)	1 (8)		4 (Men)
**Benjara**		X		1 (14+3)			2 (Men)

*Jaria was neither a primary, secondary or tertiary village

Note: Numbers in bracket indicate number of persons attending the meeting and where two numbers are indicated the first one indicates the number of men followed by the number of women.

Table 2: Summary of interviews and FGD conducted by M. Basu

Villages	Primary village	Secondary village	Tertiary village	Mixed Groups	FGD Men	FGD women	In-depth interview
**In Banswara District**							
**Bhuripara**	X				1		
**Sundripada**	X				1		
**Rohniya Mann**	X				1		
**Bhojapara**	X						
**Haldupara**		X			1	1	
**Macchharasath**		X				1	
**Jeharpara**		X					
**In Jhargram District**							
**Jainagar**	*X*				1		
**Choto Purulia**	X						
**Amakuli**	X	X					
**Bhagabandh**	X						
**Ialbona**		X					
**Khairbani**		X					
**In Baripada District**							
**Patharchakuli**	X						
**Bhurdurbani**	X						
**Badbila**	X						
**Badputika**	X						
**Mundripal**		X				1 (8)	
**Patharchakuli**		X				1 (7)	

FINDINGS

Extent of innovation within survey villages

There is no doubt from the discussions that the qualities of Ashoka rice are appreciated by the farmers who have grown the seeds (see Box 2): the short duration and the resistance to drought are the two main positive factors mentioned by all groups in the 5 districts. Improved taste is also an appreciated characteristic. Thanks to the shorter duration food grain becomes available at a time in the year when grain stocks are low and it will also facilitate the growing of a follow on crop. Women also commented that the early maturing of Ashoka eases the bottle neck period for threshing rice so it reduces the intensity of their work during this period. Ashoka also requires less cooking time which reduces the amount of firewood needed (as mentioned by women in Kherika and Udiri for example)

Box 2: Qualities of Ashoka as identified by farmers

- Shorter duration, which allows a better spread of harvest, earlier food production and possibility of second crop;
- Need of less fertiliser and water
- Less prone to disease, also in some places the only variety that will grow
- Higher yields than *desi* varieties and in case of drought still more yield than local varieties Branching more - six to eight branches from one seed , the bunch of stems

are developed and thick. The pinnacle is taller and there is more grains in the pinnacle;

- Palatable straw thus increased livestock food security
- Easier to cook and reduced amount of fuel needed
- Better taste, long white grains and used for important social events like weddings
- Higher market price

Yet, though the number of farmers who grow Ashoka has increased, the proportion of the potentially suitable land allocated by households to growing Ashoka remains small. In Kud (Hazaribagh district) for example only 25% of the suitable land is under Ashoka and in Benjara (Ranchi district) it is 20%. In all the groups across the 5 districts, farmers maintained that they will continue to grow their local *Desi* rice varieties. Farmers want to spread the risks by maintaining a variety diversity but also *Desi* rice still has some qualities valued by local people such as for example, those presented in Table C (in Annex C11) in the case of Benjara village.

Who are the innovators?

Based on the qualitative survey there is no straight answer to this question. First of all the way the seeds were distributed initially would have played a major role in determining who got seeds or not. Unfortunately there is no information from the initial growers as to what motivated them to test the seeds. GVT approached a number of villages mostly through self help groups or community groups with a given quantity of seeds to be shared equally between volunteer farmers to do PVS trials on Ashoka varieties. Typically in the villages we visited farmers got 2 kilograms per household (but in Jaria as only 2 volunteers showed interest they each received 15 kg). Then, some farmers who saw the crop growing in someone's field would have shown interest in the seeds and some of them would have been able to access seeds through relatives or friends from the original set of growers. In this process it is difficult to see whether some socio-economic characteristics would make a farmer more likely to want to try the seeds her/himself.

The structured survey (Part B) found that there is no significant difference between the people who grow or don't grow the seeds in terms of poverty levels – and that the vast majority of growers are poor. The study also concluded that "3 social traits with small but significant differences were the number of farm workers in the household, the proportion of households having a card for being below the poverty line and a lower representation of scheduled tribes". Yet in Patharchakuli and Bhudurbani (Baripada block) for example, the farmers identified as selling surplus rice, were families who had been grain secure before the introduction of Ashoka. In Bajha (Hazaribagh district) one of the growers interviewed (see Annex C4) is educated and owns a business. In the tertiary village of Lakhnu (Hazaribagh district) the seed was introduced by a farmer who owns 25 acres of land which is a large land holding for the region (Annex C9). He approached a grower when he saw his crop in field and they agreed to swap seeds of Ashoka for seeds of another crop after the harvest. Though these examples may not be representative of the farmers in the area this would suggest that examples of 'success' stories are found in households with substantial assets. As another example of a non-representative grower, in Hulsi (Ranchi district) the seeds were initially given to one farmer Litu Oraon to distribute amongst the interested self help group members (Annex C13). Litu himself had grown the seeds since 2002 and he is known for his dynamism and his personal connection with various government agencies. So he probably is an innovating farmer. These examples encountered during the qualitative work do not necessarily contradict the findings of the quantitative survey but perhaps they remind us that

even amongst poor communities there are social, economic and possibly cultural differentiations which may be subtle but which no doubt play a role in increasing the capacity to innovate.

The role of women in innovation is not very clear and seems to vary between places. In a number of the villages visited the seeds were initially distributed through the women's self help group. This was the case for example in Kud and Hutkona (Hazaribagh district) and Jaria (Ranchi district). In Jaria men claimed that women contributed to decision making on the farm but in Muramu (Ranchi district) women reported that they have little say in decision making and though they contribute quite heavily to the farm work they do what they are asked to do. In Haldupara and Macchharasath, both secondary villages in Banswara district, the seeds were initially introduced through a woman. The two women both initially got seeds from relatives and after their first harvest distributed some seeds through their SHG.

Impact of innovations

The direct positive result of the introduction of Ashoka is an increase in grain production which extends the grain self-sufficiency of the household by 2 weeks to 1 month in Banswara district, for example ; and for up to 2 months even for marginalized farmers in Hazaribagh district, depending on the size of the land available. This may sound trivial but for a household below the poverty line, this increase is not negligible. Yet this amount will not change the level of dependency on off farm income through seasonal migration and if some groups have noted a decrease in migration it is linked to the NREGA card scheme which guarantees 100 days of work per annum in the village or close vicinity. The yield increase has resulted in a decrease in grain related expenditure rather than an increase in income. There are other contextual changes such as electrification, development and roads and schools which have been reported in the discussions: these would also have contributed to the overall positive changes in local livelihoods which cannot be linked to Ashoka. Impact can also not be meaningfully visible in most secondary villages, where Ashoka has been grown only since 2006.

One technical impact that was mentioned by villagers is related to increased knowledge of better agriculture practices, in particular the benefit of transplanting rather than broadcasting of rice. (However, Ashoka varieties are not meant to be transplanted on uplands, so this is something that they presumably learned from other sources.)

One positive impact women have mentioned is that as Ashoka matures earlier than other varieties the period of harvesting and the threshing of rice is less intensive as it is more spread out. Women have said that they felt less under pressure. At the same time, women in Bhuyan Tola (Katkamsandi block) also reported that there had been an increase by 15-20 days of agriculture paid work due to the spread of rice harvest.

One woman in Kud (Hazaribagh district) reported that she had been able to convince her in-laws of the benefits of Ashoka and they had been growing it since 2003 without a loss; she said this success had improved her status within the family but also the extra production had increased the food self-sufficiency by 2 months and had allowed her to continue sending the children to school. She has 24 katthas of suitable land.

What are factors influencing the growing of Ashoka?

There are a number of factors which can help explain the level of use, but it is difficult to be categorical about their importance except for the access to bigger areas of suitable land. It is also difficult to know why in some places more people have been willing to try than in other places. These factors can be put into 2 categories: those linked to the dissemination of the technology and those linked to broader contextual issues.

- The dissemination: the picture we got from discussions with farmers across the districts is that the NGO, GVT, approached villagers with some seeds which were to be distributed in equal weight between volunteer farmers. Some farmers recall being told what to do with the seeds but not all farmers recall the same information. This could be because they don't remember exactly or because they were indeed told different things by different field workers. It is also possible that some of the GVT staff employed for this work were non agriculturists as at the time GVT was managing a livelihood project and so they may have been less able to give proper advice. In Muramu for example farmers claimed that they received the seeds from GVT without any advice. Farmers were explained that the variety was suitable for upland but some farmers said that they were told the seeds had to be broadcasted whilst others said it had to be transplanted. After the initial seed distribution there was it seems no monitoring, no follow up and GVT staff did not come to visit the fields (though according to project reports there were some subsequent field visits by the research team). Individual farmers in Bahja or Uridiri (Hazaribagh district) specifically commented on the need to have more information available. Farmers in secondary villages who got the seeds through friends or relatives, often planted the seeds in the wrong type of land or following inappropriate practices leading to failure of the crop. It seems in many cases when farmers passed the seeds on they did not pass on the technical information they had initially received. However we met one farmer in Banswara who had been advised what to do, but he said that as he 'did not believe' it was an upland variety he planted the seeds on the wrong type of land and the crop failed. In the villages in Ranchi district it seems many farmers reported initial failure because of delayed transplanting. (However, this does not make sense, given that Ashoka varieties are not meant to be transplanted.) So clearly a lack of post-distribution support by the project has played an important part in limiting the success of the technology transfer. The project was a research project and therefore no funds were available for further technical support and extension.

  The availability of, or rather the lack of seeds also limits the scale of uptake. In Bahja and Huktona (Hazaribagh district), farmers reported losing all their seeds after the drought in 2006. Not all farmers kept seeds for the following year, for example; and when they ran out there was no alternative seed supply. Many farmers told that they ate all their seeds after the first harvest and many did not have an answer to the question as to why they did not keep seeds for the following year. They simply ate the seeds. In one household in Kherika (Hazaribagh district), all the Ashoka seeds were boiled by mistake. Farmers also complained that after a while the seeds get mixed with other varieties and as they lose their purity the yield also decreases. Aspects such as planning for the future or maintaining high quality seeds could also have been part of a supportive extension package if funds had been available for this purpose.

- The context: The broader context brings a number of entangled issues. Here we are in a context of extreme poverty where most households are dependent on off-farm income for the greatest part of the year. Farmers are interested in a rice variety that increases food self sufficiency but farmers have very limited land and therefore the scale of the increase of production remains too small to lift anyone out of poverty. This can explain partly why the interest in the variety remains relatively low. There is no doubt that farmers like Ashoka. However, as its introduction has not revolutionised their livelihood system, farmers will grow it if they get the opportunity and it is the best option available at the time, but most of them will not go out of their way to grow it if they encounter difficulties.

  Farmers will also grasp any opportunity for development that comes their way. In Jaria (Ranchi district) for example, high yielding rice varieties have been introduced

by the government services and compete with Ashoka; in this village there has also been another NGO supporting different livelihoods activities such as flower growing which provide further opportunities; vegetable farming too has been developed on upland plots in the kharif season as well. In Hutkona (Hazaribagh district) people reported stopping growing Ashoka because growing vegetables upland was more profitable and also because, as vegetables mature gradually, the production extends over a longer period and so does the period of selling at the market, ensuring a longer stream of income. In Baripada, many farmers use their upland to grow sabay grass for rope making, which secures a good market price, and Ashoka is not competitive. Banswara is a maize growing district so rice remains comparatively marginal any way. This would tend to show that farmers will take advantage of any opportunity to improve their livelihoods rather than any one specific type of intervention.

The level of literacy and education is low which possibly also influences people's individual capacity to take risks or to be willing to experiment. One also cannot underestimate the influence of emulation on decisions to grow a particular variety. For example in Nehalu (Ranchi district) farmers initially grew the seed because one of the villagers experimented with it. However they stopped in their second year because they reported this man had stopped after his fourth year. There was no other reason given for stopping growing the seeds than the fact that one of the users had stopped. In Kherika (Hazaribagh district) it was reported that some farmers did not volunteer initially because they felt that if the seeds were distributed for free they would probably not be valuable.

INSIGHTS

This survey was designed to contribute to understand the field reality from a qualitative perspective, to try to unpack some subtleties which may not always be captured by quantitative surveys. Whilst there is no doubt that interviewed farmers have been very positive about Ashoka and that Ashoka has spread geographically, farmers have still been keen to reserve some of their limited land available to their local varieties. Farmers have always preserved diversity on their land to spread risks but also farmers value other qualities beyond higher yields. Though rice remains the main staple crop in most of our area of study (except for Banswara where maize is more important), farmers are also keen to seek other opportunities to increase their income. As we have seen across the sample there are other crops that farmers will grow instead of Ashoka, because of their market value, where the ecology makes it possible. (In most upland the main alternative to rice is maize and that has a lower market value.)

Discussions with farmers also highlighted that perhaps the delivery mechanism of the seeds was not as supportive of farmers as it could have been and it feels as if farmers were left to get on with the job. Naturally, technical support was non-existent in the secondary villages, as GVT was not working in these villages and would not have known if Ashoka use had spread to them. The project intended to provide support to PVS trials farmers only. More support might have helped in conserving seeds or avoided crop failures which then discouraged some farmers and decreased the level of use.

Though the study did have a gender dimension and tried to assess the level and nature of impact on women, the data does not show a strong differentiation between genders. Women did not highlight problems related to the introduction of Ashoka in terms of increased workload and they seemed to share men's views about the positive attributes of Ashoka. As overall the impact of Ashoka has been relatively small, increased benefits have not altered dramatically livelihoods and gender relations.

Perhaps another limiting factor for Ashoka expansion is its target group. By definition poor small famers have only limited land available and any increase in production per unit of land can only have a relatively minimal impact overall if the household has access to only a small number of units of land. And though for one household an increase of 2 months grain self sufficiency is important globally the impact will be limited. An increase of 20 days of labour available for women is important for these women but it remains too little to change dependency on migration. This highlights the difficulty to measure impact in an absolute way: for example women mention that Ashoka has reduced the intensity of the harvesting period, which may go unnoticed by scientists or economists but which is an important change for these women. However at the same time this change does not revolutionise women's life and hence it remains difficult to measure the impact of these changes. The example of the introduction of Ashoka in rainfed upland areas also reminds us that improved technology can only have as much impact as allowed by the context: the green revolution in Punjab did not create the dynamic wealthy landowners. Improved technology targeted at poor small farmers on marginal dry land is important for these communities but cannot produce miracles.

REFERENCES

Bourai E.A, Choudhary A., & Misra M. (2002) Participatory crop improvement in Eastern India: a preliminary impact assessment, PSP Annual report 2002, pp 25-33

ANNEXES - FIELD REPORTS by Dr. M. BASU and Dr A. DEY

The fieldwork for the qualitative study was undertaken by social scientists with limited knowledge of technical aspects of agriculture, and it appears that in some parts of the village reports in these annexes there may have been technical misunderstandings. For example, reference is made in some to farmers recounting their experiences of growing the Ashoka varieties under transplanted conditions - which is not the primary situation for which these varieties were targeted. Also, reference is made in some to high value crops (e.g. vegetables) replacing upland rice. This would only be possible if irrigation water was available and this would not generally be the case.

IA Qualitative Study - Field Visit Report: Banswara District

Malika Basu/LFC-India

Duration: 8 days (7th to 14th September 2008).

Location: Banswara-Udaipur/Rajasthan, with GVT (West) team

Planned Tasks: Meet the GVT (West) team to get an overview of the quantitative survey they had undertaken earlier and identify any emerging issues or trends; to decide upon a methodology (to follow in Banswara and subsequently in other districts) to conduct the qualitative study on the impact of 'Ashoka variety' on farmers' livelihoods.

Actual: Accomplished the planned tasks above with guidance and inputs from Dr. Marlene Buchy, Social Advisor to the IA study. (Detailed description and findings from the field stated below).

From the Field: General Information

1. The trip to Banswara was undertaken jointly with Dr. Marlene Buchy. We arrived in Udaipur on 7th September morning and drove to Banswara, approximately 160 Kms. At Banswara, we met with Dr. Yadavendra, agricultural specialist and Consultant to GVT and Mr. Tomar, a crop specialist and presently managing one of GVT's projects at Banswara. Mr. Tomar was also involved in the quantitative survey and assisted us in organising village meetings with the help of GVT fieldworkers during our stay.
2. During the meeting with Dr. Yadavendra, we discussed GVT's experience and findings from the quantitative survey. GVT team made two pertinent points. First, Banswara is a 'maize-growing' region hence a farmer's interest here to produce more of Ashoka has remained static. In other words, farmers currently may show interest in growing Ashoka but it is not their priority; nor necessarily their preferred crop. If another variety of rice gets introduced now, farmers would feel equally inclined (to forego Ashoka) and try out the new introduced variety. Second, as per GVT, following distribution of Ashoka seeds in the primary villages, the fact that seeds have further been disseminated to a number of secondary villages nonetheless highlights the upscaling of Ashoka (in terms of increase in the number of farmers growing Ashoka from the initial few who had received the seeds directly from GVT).

This, according to GVT, should be treated as an impact of introducing the Ashoka variety.

3. While we had generic discussions with the GVT team regarding Ashoka and the pattern of rice cultivation in the Banswara region; based on their quantitative survey work, we were hoping that GVT would point us towards issues or specific trends (such as migration or changing livelihood patterns or improved income and health of farmers). We had decided, as part of our methodology, to draw from some specific trends that may have come up during the quantitative survey and undertake an in-depth analysis of the same. However the team did not really have such information.
4. Given the limited information we received from the quantitative survey, we decided that we would cover all the four primary villages: Bhuripara, Sundripada, Rohniya Manna and Bhojapara. We would also visit selected secondary villages, preferably the ones, which show maximum number of growers (adopters). Eventually we visited three secondary villages: Haldupara (Primary source: Sundripada); Macchharasath (Primary source: Rohniya Manna) and Jeharpara (Primary source: Bhojapara).
5. In both the primary and secondary villages, we raised and discussed some key questions/issues which depending upon the farmers' response had other supplementary questions. The issues or questions we raised involved A) On how much acreage is Ashoka grown; whether following the first time distribution of seeds, the farmers have increased the acreage to grow Ashoka. If yes, what has been the motivating factor; if not, then what has been dissuading them from growing Ashoka on a larger scale. (Farmers who had discontinued after growing Ashoka were specifically asked the reason for their discontinuance). B) As farmers were keenly drawing comparisons between the *desi* variety of rice and the Ashoka they were asked whether this has influenced them to produce more Ashoka than the *desi* variety. C) Farmers were asked as to what they do with the Ashoka produce and whether producing Ashoka added to their food self-sufficiency D) The focus was also on existing livelihood patterns of the farmers wherein they were asked to draw a yearly calendar highlighting their agricultural and other activities in a year. The question was whether growing Ashoka has in any ways affected this livelihood pattern thereby increasing/improving their income or restricting

migration E) Other than asking farmers to state any differences they see in the *desi* variety and Ashoka, the discussion was also on the 'before and after' situation (asking whether farmers can mark any differences in the family/household situations from the time they started growing Ashoka). F) Women were specifically asked whether Ashoka has made any difference to them with regards to their agricultural activities and also owing to Ashoka whether they would like to attribute any change/s in gender roles within the family.

6. The non-growers and also those who discontinued growing Ashoka were asked what impedes them to grow Ashoka or is there any demotivating factor? Further, whether they see any marked change in their fellow farmers' lives and livelihoods who were growing Ashoka. Similarly, the non-targeted growers (Adopters) were asked as to what made them adopt Ashoka in the first place and what difference they see it has made.
7. Initially, we had hoped that atleast for the primary villages, we would conduct separate meetings for men and women. As per GVT, it would not have been possible to organise such separate meetings. Hence, in the primary villages, meetings or group discussions were conducted jointly with men and women. However, on insistence, in two secondary villages, GVT organised the women only. Interestingly, in both these villages, the starting source for Ashoka was a woman – in one case it was an *Aganwadi* worker and in another, a *panchayat* leader. These women later distributed seeds to other farmers in the village from their produce of the first year.
8. As part of our methodology, we met a health worker in Sundripada area. She had not heard anything specific about Ashoka in the village/s. According to her, in general the health status of the villages she was visiting in the area has improved. The reasons behind this were general awareness through TV and also NGO activities plus sustained government efforts through organising various camps.
9. During our visit, we also met one plant breeder, Mr. Rajesh, from GVT. According to him, Banswara being a Maize prone area, the concerned government officers have not shown interest to certify a rice variety. If a seed is certified then awareness is created regarding the seed and it is also available in the market. Mr. Rajesh was also of the opinion that the farmers to

whom the Ashoka was targeted did not own enough land hence growing Ashoka was not an incentive for them.

10. It took some time for the GVT team to realise that as part of our qualitative study, we were concerned not merely with ‘outcomes’ but also with “impact” of any outcome/s. For instance, for GVT staff, the dissemination of seeds from primary to secondary was an impact; they did not easily differentiate between outcome and impact. By the end of the trip nonetheless, they appeared quite convinced and also interested in our methods of carrying out in-depth discussions with the farmers.
11. Our trip ended with a debriefing session with Mr. Rana, the Rajasthan State Coordinator of GVT. Mr. Rana was of the opinion that Ashoka though not produced on a large scale in Banswara has nevertheless, if dissemination of seeds were any indication, gone down well with the farmers. He reiterated the point that this was a Maize growing region. Also, as per Mr. Rana GVT had a locus- standi in the state and on many matters related to agriculture, GVT was often consulted by the state government.

Highlights from Primary Villages

1. In each of the primary villages: Bhuripara, Sundripada, Rohniya Manna and Bhojapara, five targeted growers, five non-targeted growers (adopters) and five non-growers were administered the questionnaires by GVT under quantitative survey. In the village meetings conducted under qualitative survey therefore, these 15 were primarily our targets, though other villagers also turned up to take part. Amongst these, some were those who were growing Ashoka but their names did not come up under random selection for questionnaire administration.
2. In all the villages, farmers were quick to draw the comparison between Ashoka rice and the desi variety. Other than the fact that Ashoka, grown in the Kharif season, is ready for harvest within 3 months, two other points were mentioned. First, unlike the desi variety, Ashoka depends on less rainfall and second, the quality of produce is much finer for Ashoka, with its taste being the ‘talk of the village’. *Desi,* on the other hand, would take 3 months to grow; it also feels heavy after consumption and is not as tasty as Ashoka

3. In neither of the villages people showed tremendous interest in growing Ashoka. Rather farmers felt that it would not affect their livelihood patterns if they were to stop growing Ashoka now or after couple of years. Also, seed being not available in the market, it makes it difficult for them to access it. Further, farmers were themselves not careful in saving the seeds. For instance, a farmer in Bhojapara said that whatever he grew, the family consumed it all and they neither saved seeds for themselves nor did they give to anyone else in the village. So in the current year he could not grow any Ashoka. In another village, a farmer said he just did not grow it though if he wanted he could have got the seeds from a fellow farmer growing it. By not growing Ashoka he did not feel he missed something.
4. Most farmers said that they started growing Ashoka in 2003-04 when GVT gave them some seeds saying that this is a new variety, it would take less time to mature and that it was to be grown on medium type of land (called *Baigar* in the local language). The cost of producing Ashoka was minimal and even if there is less rainfall, it would yield atleast something. Farmers said they had started by sowing 4-5 kilos of Ashoka seeds. Apparently, 4-5 kilos is sufficient for 0.5 Acres. 4-5 kilos generally produced 40-50 kilos. The maximum a farmer was seen to sow was 20-25 kilos in approximately 1.5 acres of land.
5. In the primary villages, one exercise aroused tremendous interest amongst farmers. This involved drawing the yearly calendar, highlighting their agricultural activities round the year. While farmers gave a detailed description of the variety of crops they grow and produce both in Kharif and Rabi season; what we also wanted to get an idea was - which months saw an increase in migration in the village; the months which farmers feel they suffer more from illness; how many months they have food shortages; what were the other multiple livelihoods they may be engaged in other than agriculture.

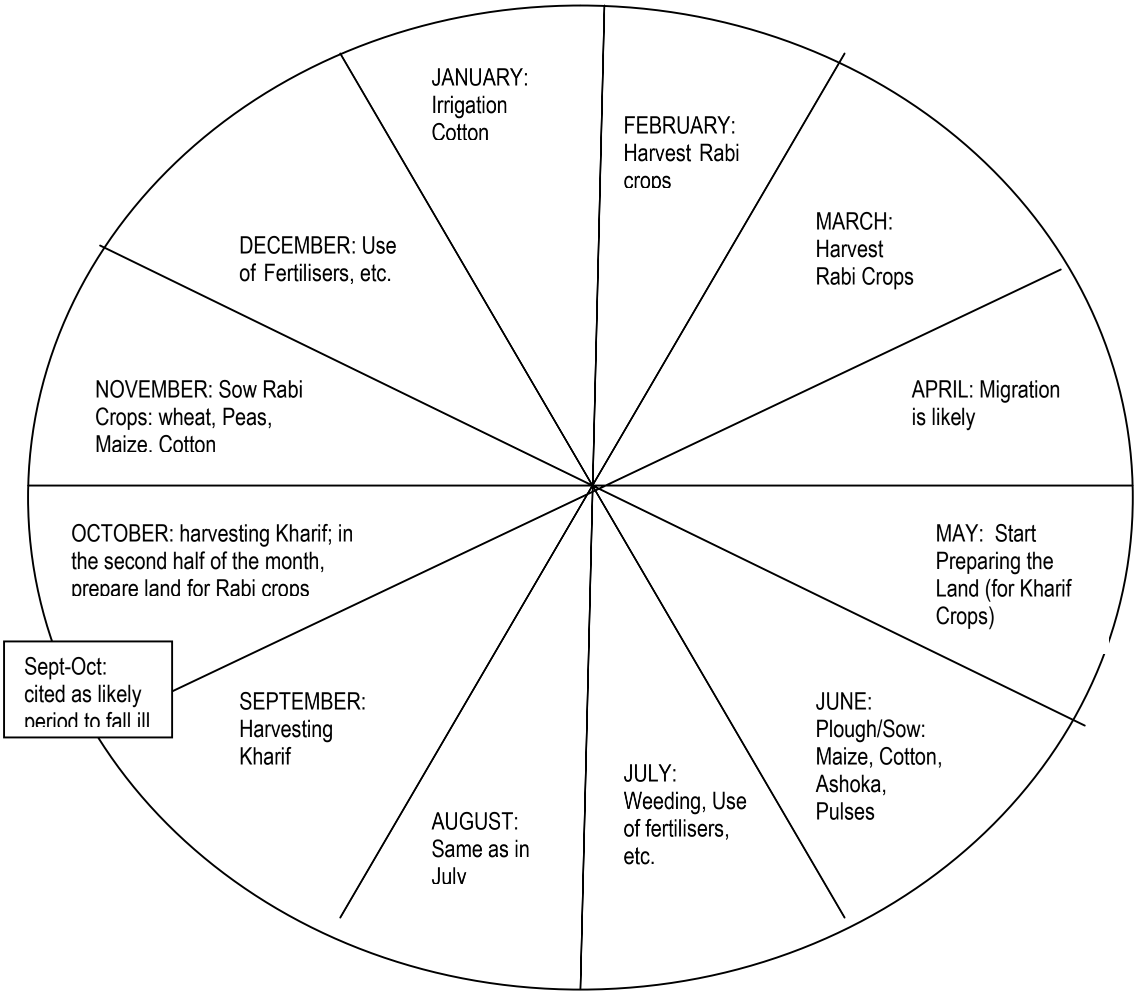

Note: The above information depicted is from village Bhojapara. At Bhojapara, amongst all primary villages, diversification of crops was most evident. Here, as compared to other villages, migration was also reported less.

6. Regarding Migration, villagers were of the opinion that with the introduction of the job card scheme (ensuring minimum 100 days work for the rural poor), the rate of migration has somewhat been arrested though not completely stopped.
7. Farmers stated the prices they receive for growing Cotton, Soya Beans and Ashoka. While one quintal of cotton fetches Rs.2000; a quintal of Soya Beans is sold for Rs.1500. One quintal of Ashoka the farmers, if sold in the market, would fetch them anywhere between Rs 1000-1200.
8. In Rohniya Manna, during our meeting, one of the farmers mentioned a name of a village where he had given some seeds. He apparently had not mentioned the name during the focussed group discussion conducted earlier

as part of the quantitative study; hence, the village name could not be listed under secondary villages (for a random sampling)

Highlights from Secondary Villages

1. In the secondary villages we visited: Haldupara (Primary source: Sundripada); Macchharasath (Primary source: Rohniya Manna) and Jeharpara (Primary source: Bhojapara), in two of these – Haldupara and Macchharasath, it was possible to hold meeting with women. Interestingly, in both these villages the primary source i.e. the person who got/introduced the Ashoka seed first was a woman. In both the villages, the women had visited their relatives (in primary villages) where they also had eaten the rice. They quite liked it and hence got around 10 kg to sow it on their own land. After their first produce, they distributed the seeds to others in their respective villages. The seeds were distributed to members of SHG group to which these women belonged.
2. It was difficult to showcase the impact of Ashoka in the secondary villages. Moreso, as here the primary source had been growing Ashoka only for two years on a limited scale. The current – 3rd year – it had not been harvested yet. In secondary villages where seeds had been distributed, at the time of the field work, it was only their 2nd year (yet to harvest). So, other than saying that they liked the taste of Ashoka, Ashoka growers felt it was difficult to talk about impact.
3. In one of the secondary villages, Jeharpara, two people had brought the seeds. Unfortunately though they lost their crop as they grew it on low land. Also, they had not spoken about the new seed in their village. Hence, the meeting with these two farmers did not yield any answers on Ashoka. Such cases appeared in the secondary villages i.e. lack of information leading to loss of crop.
4. In the secondary villages, the amount of Ashoka seeds distributed from the primary source (the person who got it into the village first) to others was minimal – not more than 2 kilos and to a maximum of 10 members. The 10 however had not distributed further the seeds.
5. Like in primary villages, in secondary villages too farmers cited the same differences between the *desi* variety and Ashoka.

6. Some of the non-growers sitting in the village meetings stated that they would like to grow Ashoka but how to get the seeds?

Other Observations

During the field visits, a lot of issues came up from the farmers' side that made the discussions move beyond just outcomes and impacts. These issues relate to the process of innovation itself and how it engages community participation; it relates to issues of dissemination, upscaling and sustainability.

1. Issues of Upscaling: Talking to GVT members, it was clear that in their understanding 'upscaling of the Ashoka variety' meant increasing the number of growers (especially the non-targeted growers). What was interesting to note in the field was that while upscaling in terms of number of farmers may have taken place somewhat, there has been no significant upscaling in terms of increase in (individual farmers' interest to upscale) the acreage in which s/he was growing Ashoka. The point is, even if a farmer has 1-2 acres of medium land conducive to Ashoka growing, s/he would not prefer to grow it on more than 40-60 decimals. There were very few cases of the farmers who said that they have increased the acreage in terms of growing Ashoka One prime reason cited was that Banswara region was a maize growing area.
2. Issues of Dissemination: This became more apparent in the secondary villages. For GVT, 'dissemination' is taken more as 'passing of seeds' from one farmer to another; from one village to another. However, what was caught lacking in the secondary villages was the dissemination of information that goes with the growing of Asoka seeds. The result of such lack of dissemination was farmers lost their crop by growing it on lowlands or spreading unwarranted pesticides.
3. The perception that Ashoka would yield good results on a medium land seem to vary. This is to say that even in medium land, there exists difference in its texture and soil type, which may lead to different types of medium land yielding different amounts of Ashoka.
4. Of all the farmers we met, only one farmer in Rohniya Manna has so far sold Ashoka twice in the market. Also, it appears that if farmers sell it they would sell the paddy and not the rice after threshing. The *desi* paddy fetches Rs.6 per kilo while the paddy of Ashoka is sold for Rs.11-12 per kilo.

5. Be it in the primary or the secondary villages, farmers cited the same differences between the Ashoka and the *desi* variety. *Desi* in particular has one quality, which is lacking in Ashoka and i.e. it gives more fodder to the farmers to feed its cattle.
6. Issues of Livelihood: meeting the farmers one could not draw a clear and direct impact of Ashoka on farmers' livelihoods. If some changes were mentioned with regards to Ashoka it related to food consumption of farmers. Following Ashoka, the farmers feel that owing to the fact that Ashoka tastes better and also is easy to digest, it is possible that when they eat rice, they consume more of it. Also, since Ashoka gives more produce per kilo as compared to other variety of rice and matures early, the farmers have more rice to eat for longer months but it has not had a tremendous effect on the food self-sufficiency. The margin of food shortage may have got reduced from 15 days to 1 month.
7. A point excitedly made by farmers in all villages was that Ashoka being good quality rice, it was served with much pride during weddings. It added to getting complemented by fellow villagers for feeding the guests with good rice. There were occasions when even if a farmer was not growing Ashoka he requested on such special occasions to borrow from fellow farmers in exchange of something else.
8. A point that was pointed out by GVT was that Asoka is not a recognised variety in the state. Sustained efforts have also not been made to certify it. It being a Maize-growing region, where farmers grow maize both in Kharif and Rabi season, the farmers too in the meetings spoke of introducing variety in Maize crop rather than pointing out any improvements in Ashoka.
9. Issues of Sustainability: this could be a cause of concern, moreso, as farmers presently do not show that they are making efforts to save the seed for future planting. If at all seeds are being saved and re-planted, it is because farmers at an individual level are saving it. There is as yet no concept of seed bank or any other method so that access could be made easier for other farmers and also that seeds are available for the future. One woman in a meeting laughingly said, she had saved some seeds but then she went out of village for work and by the time she returned she saw the family had consumed it all – since all like the taste of Ashoka. That was the end of Ashoka growing for

her; she did not ask seeds again from anyone else nor was it available anywhere.

1. IA Qualitative Study - Field Visit Report: Baripada District

Malika Basu/LFC-India

Duration: 6 days (17th to 22nd September 2008).

Location: Baripada /Orissa, with GVT (East) team

Planned Tasks: Meet the GVT (East) team to get an overview of the quantitative survey they had undertaken earlier and identify any emerging issues/trends. Conduct the qualitative study on the impact of 'Ashoka variety' on farmers' livelihoods.

Actual: Accomplished the planned tasks above. (Detailed description and findings from the field stated below).

General Information

1. In Baripada, GVT (east) team assisted me in conducting meeting and interviews. The same team of Mr. V.K.Tomar (programme officer) and Mr. Das (field level officer) had undertaken the quantitative survey. Upon arrival in Baripada, from Bhubaneshwar (approx. 230 Kms), I had a meeting with the team regarding their experiences during the survey. Dr. S.P. Prasad, senior consultant in GVT and agricultural specialist was also present. Like in Banswara, though general discussions on Ashoka took place, the team could not cite any specific trend/s in the villages they had covered. Dr. Prasad in particular, though appreciating the qualitative study we had decided to undertake, nevertheless added that discussing Ashoka is also a 'matter of perception'. He believed that Ashoka, a medium-land crop has received wide acceptance in the area; though its upscaling may appear restricted. The reason for this would be that people might not be having adequate amount of medium land to grow Ashoka. From a scientific (and agricultural) point of view, Ashoka was an innovative variety with peculiar characteristics that enabled farmers to harvest an early crop.
2. Following the discussion with GVT team, I decided to follow the same methodology as followed in Baripada i.e. cover all the primary villages and cover one secondary village of each primary village. Alongside, if any interesting individual case comes up during the village meetings I would follow it up with an in-depth interview (with the concerned person). Further, I would

try to meet someone from the agricultural department and if possible, also the health or the *Aganwadi* worker who generally have close links with the village/villagers.

3. It may be mentioned that due to time management issues on the part of GVT staff and also difficulties faced by the staff in organising village meetings, the first two days were not as productive as one had hoped. One had to wait long in the villages before the meeting could be arranged. In one place, despite the long waiting, we could not gather and meet the farmers. Given the time lost, it was not possible to cover, in the remaining days, all the secondary villages. GVT suggested that the lack of village cooperation was due to the fact that a few years previously GVT had suddenly withdrawn activities in the villages (owing to withdrawal of a DFID project). When the quantitative study for Ashoka was conceived, GVT team tried to re-establish contacts in these villages, which apparently proved difficult.
4. Though the team cited no specific trends, they did mention that in Baripada, Ashoka was grown on the medium land. In the upland people were growing *Sibai* grass. This was a major source of livelihood for the villagers. *Sibai* grass is primarily used for making ropes - found very useful for doing multiple things (e.g. cot making, to fasten and secure things, etc.)
5. As per GVT, given their efforts, Ashoka will soon be a certified seed in Orissa. It has already gone through all the formalities of registration. Certifying a crop, as GVT said, ensures its quality control.
6. In the Baripada region, GVT has also opened a seed production unit. Under this, it has identified a number of farmers, who are promoted to produce Ashoka on a large scale. All these are big farmers from whom at the end of the harvest period, GVT buys the seed back.
7. Under the RIU project, GVT has recently distributed Ashoka seeds (2 kilos each) to. 900 farmers. As per GVT, all their secondary villages got covered under this new distribution scheme. It appears that the seeds are given randomly to farmers (who show interest to grow Ashoka) and also no documentation is being carried out at this stage regarding the farmer who is receiving the seed. The field officer told me that they go to a village and for record, only write down the name of the person to whom the seed is being given.

From the Primary Villages

1. All the four primary villages: Patharchakuli, Bhudurbani, Badbila and Badputika - were covered. In Bhudurbani, it was possible to conduct a meeting with the women. Apparently, in all these villages, GVT had targeted the existing men's groups in 2003-04. The secretary of the group was given the seeds (2 Bags= 60 kilos); then in a group meeting the members decided it amongst themselves as to who should receive the seeds or who was interested to experiment with the new seeds. As per the farmers, during the disbursement, GVT gave them the basic information regarding Ashoka – its characteristics, its benefits and how/when to transplant. Roughly 6-7 farmers received the seed in the first instance. Following their first harvest, the farmers distributed the seeds further.
2. In Badbila, the group was most vocal regarding their experiment with Ashoka and its benefits. But they also added that it would benefit more if it could be grown on a larger area. They cited examples from amongst themselves - of couple of farmers, who were currently selling Ashoka in the market. After the group meeting, I could speak to one of these farmers who pointed out that beginning with 80-90 decimals, today he is growing Ashoka on nearly 2 acres of land which gives him roughly 12 quintals. Last year, he sold the paddy between Rs.600-700/per quintal whereas the *desi* variety fetched him around Rs.550. He also noted that he received training and information from GVT regarding line transplanting of Ashoka, how and when to use compost, the time of sowing and harvesting. What he missed in not getting proper information was knowledge about how to package the seed so that it can be saved for subsequent years. Cases like this (targeted) farmer who was selling Ashoka were rare in the villages I visited.
3. In Patharchakuli and Bhudurbani however, two non-targeted farmers were found selling Ashoka. But neither of them had been administered the quantitative survey questionnaire since their names did not appear in the randomly chosen sampling list. Again, both these farmers had 1 acre or more on which they were growing Ashoka. Their families had not faced food shortages even earlier. One of them also had a government service.

4. Like in Banswara, in all the primary villages, the farmers drew the yearly calendar depicting their year-round agricultural and other livelihood activities. It was felt that with the growing of Ashoka even if the entire food deficit was not covered, the period of shortage (depending on the family size, land yield) had reduced by at least 15 days to a month. The farmers said that they managed their food deficit earlier (and even now) by buying rice from the local market.
5. In the primary villages, talking to farmers regarding what advantages they think the Ashoka variety has over others; five points were made. First, the *desi*/other varieties take more time to mature as compared to Ashoka. Second, other varieties require more fertiliser, whereas Ashoka can do without too much fertiliser. Third, even if the rainfall is less, Ashoka would yield atleast something. Fourth, Ashoka yields more produce than other varieties. Finally, it tastes better; also, its fodder is softer and hence easy for the cattle to chew.
6. In the meetings when the farmers were involved in drawing a yearly calendar to cite their agricultural and other activities, it appeared that they do not grow any crop during the Rabi season. Apart from the kharif season, the farmers grew paddy in what they prefer to call the 'summer season' (Jan-Feb to Apr-May). But this summer season crop is possible if the farmers have irrigation facilities, which again is rare.
7. Despite the benefits people tend to draw when asked about their experiences with Ashoka as a crop, not all in the village having medium land were found growing it. Further, people did not grow only Ashoka on their medium land; they preferred to combine it with some other variety. The other variety, which people were growing, was *Lalat*. Farmers cited one prime reason for not growing Ashoka 'only'. With Ashoka if you do not transplant within a definite period then the crop gets spoilt and there is no yield. Farmers would not like to take risks with growing just one crop on their medium land. Just incase Ashoka does not yield anything, then they would atleast have the other one.
8. Few farmers in the primary villages noted that they find the straw of Ashoka very useful in making the roof of their houses.
9. As mentioned, rope making from *Sibai* grass is a very popular activity in Baripada region. There were hardly any households in the village, which did not engage itself with this activity. Depending on whether the rope is made

thick or thin, price is fixed but as farmers said that this rope making activity - it could easily fetch them Rs.150 per week. It seems, 80% of the upland is used to grow *Sibai* grass. People even buy this grass in the market, as this business is found lucrative.

From the Secondary Villages

1. Two secondary villages: Mundripal (primary source: Bhudurbani) and Badputika (primary source: Badbila) were covered. In Badputika, the meeting could be held with the women.
2. From the FGD reports of GVT, it was interesting to know that quite a number of farmers were engaged in growing Ashoka. During the meetings however it came out that only one-two persons got the seeds from the primary village and following his first harvest gave seeds to couple of others. But beyond that the seeds were not further disseminated. In the records, the reason for the increased number of farmers growing Ashoka was that last year, GVT had again given seeds to farmers under the RIU project. GVT team said that in 31 villages (which also included all the secondary villages) around 900 farmers were given 2 kilos each. So this year when the team came to do the FGDs before the quantitative survey all these farmers (who received the 2 kilos of seed) put their names.
3. In Badputika in particular, people said it was too soon to talk about impact, as here it was only a year that the people had experimented with Ashoka. This was their second year in running.
4. Women both in Badputika and Bhudurbani made a pertinent point. As per them since Ashoka matures early, it gives them more time to undertake the threshing work thus distributing their workload over a period of time. Earlier all the crops were maturing at the same time; it meant too much work at the same time – and threshing work is also quite tiresome.
5. A common point that was made by farmers, both in the primary and secondary villages, was that with Ashoka they are atleast assured of some yield. Also, whether they sell paddy or not, the farmers felt that Ashoka is likely to fetch them more money as compared to other varieties. Farmers, if they want to sell do not generally sell paddy at on go; they sell as and when faced with any emergency/difficult situation. Having Ashoka at home could

help them get a higher price when in need. But they also added that problem with Ashoka is that, if not stored well it is likely to get spoilt.

6. In the secondary villages, farmers said that they did not receive any information on Ashoka from GVT. If they got to know anything about the nature of the crop then it was from amongst themselves. The farmers who had grown it and seen the harvest shared their experiences with those who were interested to grow. It was however added that because farmers did not have adequate information, they did not want to take the risk with this new variety, especially those who had very less land. But when GVT came again giving 2 kilos to each farmer, more farmers showed interest (hence the number has increased).

Other Observations

1. Apart from rope making, be it in the primary or secondary villages, people were engaged in multiple activities, which included collecting and selling of *Mahua* and *Tendu* leaves. Selling of Bamboo was also common.
2. Almost all those who came for the village meetings said that they had job cards. Villagers noted that issuing of job cards was a positive step towards ensuring work during lean periods but this must not suggest that the migration flow has been completely arrested. Members from different households, especially the landless or ones with very less land, still go out of their villages for work.
3. What was mentioned in all the villages was that though people liked Ashoka, they could not still grow it. The reason being, Ashoka was said to be conducive to medium land and such land was limited in the village. All the villages had far more upland than they had medium land. Medium land roughly amounted to 20% of total land type. In the upland almost all in the villages were growing the *Sibai* grass.
4. On recommendation of GVT, I met one farmer who has achieved tremendous success by growing Ashoka. Meeting him rather reconfirmed the fact that Ashoka is likely to benefit large farmers than small and marginal ones. In this case in particular, the farmer was initially given some seeds on an experimental basis. He was to try it out on his 10 decimal land. From 10 decimal he is now growing Ashoka in 3 acres and more and receives 30

quintals of which 28 is sold back to GVT at the rate of Rs8/per kilo. This farmer did not belong to any primary or secondary villages. (Such cases were also not visible in the villages visited by us, though there were couple of cases wherein the farmer particularly mentioned doing well by selling Ashoka like the targeted farmer in Badbila). As mentioned earlier, GVT is running a seed production unit in Baripada for which it is promoting selected farmers to grow Ashoka on a large scale. The farmer in question is a promoted farmer.

5. Following the qualitative study, the programme officer who was accompanying me was of the opinion that their quantitative survey could have added some additional questions, which would have facilitated our qualitative study. The findings from the field made him feel that the following two questions should have been included in the questionnaire. First, apart from knowing the three distinct land types the farmer owns, there should have been a question regarding the distinctions within each land type (he has). The reason being, farmers said that they were not growing Ashoka not only because medium land was limited but also because within the medium land, the lower side of it was more conducive to grow Ashoka and not many may have that. Second, a question should have been dedicated to the multiple activities a farmer was engaged in which allowed him/his household a source of income other than agriculture. The reason for this question to be included was that in all the villages, farmers opined that if they did not have these other multiple activities, it would be extremely difficult for them to survive and sustain themselves. These activities brought money in hand by which they could meet their daily and other needs.
6. GVT also organised meetings with two agricultural officers in Baripada. It turned out that the two had extreme views on Ashoka. One of them felt that information regarding Ashoka was not correctly disseminated. As per him, Ashoka was a not a genetically pure variety; also, it was not meant for kharif season but more conducive to medium land during Rabi season. The maintenance of Ashoka seeds was also difficult. Also, Ashoka does not take 90 days to mature as propagated but actually takes 100-110 days. He said his source of information were the farmers themselves. He felt that for any new variety seed, the following is most essential – field demonstration; training with proper guidelines to ensure timely application; certification for quality

control. The other officer however was of the opinion that as far as his knowledge goes Ashoka has been a good variety that is helping farmers to a large extent to meet/overcome their food deficit.

7. Finally, one health worker met in the area who visits couple of primary villages, said that in her opinion, people are much more informed these days in terms of health issues. She however has not heard of Ashoka hence could state if it had had any impact on farmers lives.

2. IA Qualitative Study - Field Visit Report: Jhargram District

Malika Basu/LFC-India

Duration: 5 days (23rd to 27th September 2008).

Location: Jhargram-Kolkata/West Bengal, with GVT (East) team

Planned Tasks: Meet the GVT (East/Jhargram) team to get an overview of the quantitative survey they had undertaken earlier and identify any emerging issues and trends. Conduct the qualitative study on the impact of 'Ashoka variety' on farmers' livelihoods.

Actual: Accomplished the planned tasks above. (Detailed description and findings from the field stated below).

General Information

1. At Jhargram, to assist me, GVT had assigned one of its programme officers (POs), newly appointed (four months), and another local Bengali boy – not a GVT employee but who had helped the programme officer (and another GVT staff) during the quantitative survey. Conducting village meetings proved difficult as communication issues amongst the GVT team and between the team and the villagers. It was also difficult to gather targeted villagers rather than whole village meetings. The idea was not to bring anyone who can be found in the village to the meeting but arrange the meeting particularly with those who came under GVT's list of targeted and non-targeted growers and preferably the 5 non-growers who were administered the questionnaire. While tasks were accomplished, lot of time was equally wasted.
2. Before making the field visits, like in Banswara & Baripada, I had a session with the two GVT representatives to discuss any trends or impacts they witnessed during their FGDs and/or the quantitative survey. Once again, it did not yield any result. GVT team could not highlight a particular issue/s.
3. Though the GVT team did not mention it but looking at their FGD reports on secondary villages, it was interesting to note that in comparison to primary villages, the number of farmers growing Ashoka in at least in some of the secondary villages there appeared to be more growers.
4. For the qualitative assessment, the same methodology was followed as in Banswara i.e. cover all the four primary villages and at least one secondary

village of each primary village. Unfortunately, only three secondary villages could be covered. One of the secondary villages, I had marked ‘to visit’ (looking at the reports of FGDs conducted earlier) – GVT said that it was too far and the roads were not good at all. Also, even if we reach the village, if it rains then we are stuck as roads were of mud and sand. A second village amongst the secondary villages I had decided to cover too could not be visited – for the same reasons; instead, the team itself proposed another village.

5. The questions asked in the village meetings were also on the same lines as Banswara (Refer to Banswara report).

6. In one primary and one secondary village, meetings could be conducted with women only. In one secondary village, Kherikabad, women appeared very well informed on Ashoka (and this was not via any training or information they received formally or through GVT).

7. In Jhargram, people do not grow Ashoka on medium land. Here, upland is found conducive to grow Ashoka. Also, while no paddy is grown during Rabi season; other than the Kharif season, some people (like in Baripada) also talked of a “summer season” (Jan-Feb to April-May) when they grow paddy. (It seems during this season, those who grow Ashoka – though the number is very few - they benefit from it in terms of better yield as compared to Kharif season).

8. Two farmers – one each in two primary villages were also interviewed separately as they were the rare cases who, since they first started growing Ashoka in 2003-04, had been growing it in consecutive years, without a break. And both strongly felt that their situation has improved from growing Ashoka.

9. In Jhargram, except in one village, farmers preferred calling Ashoka as Khandagiri. While most could not cite the reason as to why it is called so; one farmer said that there is a variety called Khandagiri – its seed is sold in the market and the paddy also resembles Ashoka. Some farmers who had received seeds directly from GVT however knew that the name was Ashoka.

From the Primary Villages

1. In all the four primary villages: Jainagar, Choto Purulia, Bhagabandh and Amakuli, meetings could be conducted. In Bhagabandh, an *adivasi* (tribal) village, it was possible to conduct the meeting with the women (female

members primarily from those households constituting targeted and non-targeted farmers). These women were also part of SHG.

2. In all the primary villages, two points were common. First, in all the villages, farmers said that after growing Ashoka for 2-3 consecutive years; it was quite natural for them to change the variety crop. Hence, following Ashoka, farmers were growing other varieties such as ‘Bullet’, ‘Culture’. Apparently, these varieties – though their yield was comparatively less - were also sowed and harvested more or less during the same time as Ashoka. Second, in all the villages, farmers were engaged in activities other than agriculture, which also allowed them to earn some money either on weekly/monthly basis.
3. As for Ashoka, an interesting fact was mentioned. Almost all farmers were growing a variety called ‘Swarna’ on their medium land. It was stated that if people had to sell paddy they preferred to eat Ashoka and sell Swarna. Other than the fact that Ashoka is tastier and easy to digest, there were two reasons cited: one, Ashoka is harvested early so households do not have to wait for Swarna to mature to feed themselves, they start consuming Ashoka (and it is also liked); second, since Ashoka yields more as compared to other varieties, the food shortage is reduced. Earlier with the local varieties grown on upland - the yield being comparatively less than Ashoka - households had to eat into their stock of medium land (variety) produce. That stock is now better saved so people use it (or at least have the option) to sell in the market.
4. As in Banswara and Baripada, farmers were quick to cite the benefits of Ashoka. These included – Ashoka requires less input and incurs less cost; it is infected less with diseases; less spray is required over its crop; its paddy fetches more price than other varieties.
5. In no village did farmers say that they did not like Ashoka. However despite this, not all - having some amount of upland - were found growing Ashoka. The farmers stated that if they were not growing Ashoka it was because they had limited upland; also, it was possible that some farmers who had grown Ashoka earlier were now growing something else as farmers preferred to diversify their crops rather than sticking to one variety.
6. There were many farmers who indeed had stopped growing Ashoka. While some of them cited less water as a problem that could affect any rice cultivation; some categorically stated that since Ashoka is grown on the

upland and it matures faster; during its harvest, farmers have to walk through their standing crops on the medium land. This adds to the difficulty of transporting Ashoka and also since farmers carry it on their shoulders, that too is quite tiresome.

7. No one as such reported any decline in the produce of Ashoka i.e. if farmers used 5-6 kilos of Ashoka seeds and received nearly 1.5-2 quintals, according to them, this ratio has remained more or less the same in the last 3 years. Though, few farmers pointed out that they feel the seed has reduced its size.
8. In all the primary villages, GVT gave roughly 30/60 kilos of Ashoka seeds to the farmers' (male) group (rather GVT gave it to the secretary of the group). The seeds were then divided amongst interested farmers. The number of farmers using it in the first instance varied between 6-7. Following their first harvest, others showed interest and requested for seeds. That is how the use of seeds spread in the primary villages. The farmers (as mentioned earlier) stated that not all those who first used the seeds may still be continuing. Per village, roughly 2-3 people may have grown Ashoka without a break.
9. Here again in the primary villages, we asked the farmers to draw their yearly calendar of agricultural/other livelihood activities. The calendar again showed people involved in multiple activities throughout the year. In Jhargram area, though people have job cards but apparently it has not yielded as much work as they thought it would. Migration therefore remains a common factor.

From the Secondary Villages

1. Three secondary villages were covered: Lalbona (primary source: Jainagar); Khairbani (primary source: Choto Purlia) and Kherikabad (primary source: Amakuli).
2. As mentioned earlier, FGD reports of GVT had showed that as compared to the primary villages, more people seem to have experimented with Ashoka in the secondary villages. In the village meetings, the number appeared to be far less than what was stated in the FGD reports. The farmers said that the number of Ashoka growers has been constantly fluctuating so they would not negate the number mentioned in the FGD report; what is equally true is that many may have experimented once and then stopped. (This does not mean that they would not grow it in the future).

3. In Kehrikabad in particular, where the meeting was conducted with women (also part of SHGs), they appeared well informed regarding Ashoka. For instance, women were clear that they need to transplant Ashoka within 21 days; that it is an upland crop needing less inputs and it yields more than other varieties. Women said that the person who had got the seed first in the village had told them these facts; also, they witnessed the harvest. Kherikabad was also one village where women stated that they preferred to eat other varieties of rice than Ashoka. They said Ashoka is 'thin and slender'; whereas they were used to the 'thick and heavy' grains. Thus, in Kherikabad, if people have to sell paddy after covering their food deficit, then they would prefer to sell Ashoka. However cases of selling paddy in the village were rare.
4. In the secondary villages too, some farmers noted that seed after 2-3 years appears marginally smaller than the original seed.
5. Here too, farmers cited benefits of Ashoka amongst which they noted that due to Ashoka they could now grow better (quality) vegetables (on the same land).
6. Other than agriculture, farmers' households were engaged in other activities, which yielded some income. These activities (also in primary villages) included mattress making, vegetable selling and leaf selling. Migration was common in secondary villages as well.

Other Observations

1. One of the health workers met in the area made an important observation. She was initially surprised that I had come to speak to her regarding the health situation in the villages she covered (one of them being a primary village of GVT). While she noted a general improvement in health in the villages, she had not heard of Ashoka in particular. Rather upon hearing the purpose of our visit to conduct a qualitative study, she had a suggestion. She felt that at the time Ashoka seed was introduced, if the organisers had also informed the village health worker, *Aganwadi* worker, schoolteacher, then they too would have kept themselves informed regarding the farmers who received the Ashoka seed. As per this health worker, the villagers know people like her or the *Aganwadi* worker; so it would be easier for them to communicate with the villagers and be aware of their situation.

2. In Jhargram, people said they were growing Ashoka on their upland area. (The medium land was used for growing a popular variety called *Swarna*). In the FGDs conducted by GVT in the villages, it showed that villages had more upland than medium land. But when it came to growing Ashoka not even 20 percent of the upland was being used to grow Ashoka. On asking why more of upland was not being used, farmers noted that even in upland there are differences. That is, people were growing Ashoka on the ‘low-side’ of upland – and not all may have land like that.
3. In Jhargram area, people were growing paddy under Kharif season and a “ summer season” (Jan-May). In the Rabi season primarily wheat and mustard were grown. Alongside paddy in the Kharif and summer season, farmers were growing maize, vegetables, groundnut and peas. Some were even growing cashew.
4. In all the villages we visited, the number of Ashoka growers has been fluctuating. When farmers were asked that when they stop growing Ashoka for a year or two, from where do they expect to get the seeds when they wish to grow again – farmers said that ‘someone or the other in the village is growing Ashoka, we can easily ask the seeds from him’. However, it was not denied that saving seeds might gradually become a problem if people do not start thinking about saving them. One farmer in fact said that he thinks the risk of not having access to Ashoka seeds is more for two main reasons: first, it is not accessible in the market so if a farmer loses his crop then he may not have any seed to save. Second, the storing of Ashoka He cited his own example. It seems he lost the seeds he had saved for the next season, as he did not store it properly. He said that his way of storing is still very ‘traditional’ (putting in a big earthen pot) and Ashoka requires proper packing for a better storage.
5. Even in Jhargram it was the big farmers with 10-12 Bigha who spoke highly of Ashoka (and tend to benefit more, even monetarily).
6. Farmers noted that those who gave Ashoka seeds to others to grow they did that in exchange of other variety seeds (*Swarna*).

3. **Bajha, GP Bajha, Block Katkamsandi, District Hazaribagh, Jharkhand**

Dr Dey

**Date**	24 March 2009
**Methods**	Observation, IDI (1)
**Participants**	Ashoka growers
**Qualitative Study team**	Dr Aniruddha Dey, LFC India and Mr Albert Xalxo, India – Field Support Officer, supported by Mr Vinay Tomar and Mr Sanjeev Ku Singh, representatives from GVT East.

Bajha was not initially selected for qualitative study but on 24 March when the GVT representatives were busy in contacting people in different villages for the study The LFC India decided to conduct an interview with a few Ashoka growers, mainly to develop an understanding. Accordingly interviewed Mr. Ram Kumar Rana (40 years), Matriculate, he is farmer, also work as LIC agent, he has own Photo Studio at Katkamsandi market. He is grower of Ashoka rice. His family backgrounds are as follows:-

Family member	Age	Qualification	Occupation
Father	70	Literate	Agriculture
Mother	62	Illiterate	House wife + agriculture
Wife	32	VII	House wife + agriculture
Daughter	14	IX	Student
Son	10	V	Student
Son	8	III	Student
Son	6	I	Student
Elder brother	46	X	Medicine shop, shifted to Dhanbad with his family, no
Elder brother	43	V	Agriculture
Elder brother's	40	Literate	House wife + agriculture
Daughter	15	X	Student
Son	12	VII	Student
Son	9	IV	Student
Younger brother	37	Non metric	Carpenter staying at Delhi
Younger brother	30	Metric /IA	Photo studio
Younger brother's	28	Non metric	House wife + agriculture
Son	4	LKG	Student
Daughter	3	-	-

Total land area: - 7 acres [undivided land among family members]

1. Up land = 1 ½ acres.
2. Mid land = 2 ½ acres and
3. Low land = 3 acres

Crops grown in up land along with sowing and harvesting time:

Sl. No.	Item name	Sown	Harvest time
1.	*Sarguja*	June -	Sept -
2.	*Kulthi*	June -	Sept -
3.	*Urad*	June -	Sept -
4.	*Mater [pea]*	October	Dec - January
5.	*Chana*	October	Dec - January
6.	*Mustard*	October	Dec -January
7.	vegetables	October	Dec - January
8.	*Goda* rice	July	September
9.	*Ashoka* rice	July	September

Remaining four months from February to May land remains fallow.

Crops grown in mid land along with sowing and harvesting time:

Sl.	Item name	Sown	Harvest
1.	*Goda* rice	July	October
2.	*Ashoka* rice	July	October
3.	*Karhani* rice [early 6o days variety]	August	October
4.	*Menathori* rice	July	November
5.	*Tesi* (oil seed)	November	January
6.	*Khesari* (Pulses)	November	January
7.	*Batra* (Peas)	November	January

Remaining Five months from February to June land remains fallow.

Crops grown in low land along with sowing and harvesting time:

Sl. No.	Item name – Only Rice	Sowing time	Harvest time
1.	*Khoraphul*	June	
2.	*Lal Gandhari*	June	
3.	Pioneer (HYV)	June	
4.	JK rice (HYV)	June	

Remaining Six months from December to May land remains fallow.

He started growing Ashoka rice in the year 2003.

Year	Seeds were	Quantity of seeds	Type of land	Area covered	Production
2003	GVT	10 kg	Mid land	0.20	2 quintal (200kg)
2004	Own	40 kg	Mid land	1 .00	4 quintal (400 kg) during this year
2005	Own	-	Up land	0.50	12 quintal (1200kg)
			Mid land	1 .00	
2006	Drought declared. They could grow some potatoes because of irrigation facilities				
2007	own	-	Mid land	0.50	6 quintal (600 kg)
2008	own	-	Up land	0.50	8 quintal (800 kg)
			Mid land	0.50	

Knowledge

He said they were informed that it could be grown only in medium land, no fertilizer required and after harvest to share seeds with relatives and other willing villagers to increase the number of growers. Seed bed should be prepared by the July end and then transplantation within 20 to 25 days required.

Seed priming

Seeds to be soaked in water for whole night and taken out in the morning and then to be covered during the day and to be broadcasted in the evening. However sometimes they allowed the seeds to germinate and in the next morning broadcasted.

Gender issues

Event	Process	Responsibilities
Preparation of seed bed	3 to 4 time ploughing and levelling and this was done accordingly. Only cow dung was	Male
Transplantation	After completing above process transplantation was done when there was	Female
Weeding	After 15 to 20 days weeding was done and no [illegible]	Female
Harvesting	Within 90 days	Male + female
Threshing	On the field	Male
Winnowing	On the field	Male
Carrying	From the field to thrashing field [*Khalian*]	Male + female
Boiling	Paddy with water	Female
Rice husking	Separating of rice from husk	Female
Milling	Taking paddy to machine for making rice. There exists traditional method of grinding at [illegible]	Male

Table A: Differences between Ashoka rice and local variety

**Ashoka**	**Local variety**
Rice grains are thin, long and tasty	*Menathori* is comparable with it in terms of taste only and not in other quality.
Less water required and best adapted	Requirement of water more
Early variety hence after harvest other crops can be grown	Late varieties and other crops impossible
Pest infestation low – no pesticide used	Pesticides required
Not laborious	Laborious

Farmers visit less in the field for watching because they don't have to bother much about the water	Local variety need frequent visits
Own seeds can be grown repeatedly	Own seeds can be grown repeatedly
If everything goes right then in 1 acre of land production of Ashoka will be 7 quintals (700kg).	Local variety *Goda dhan* production will be 5 to 5.5 quintiles (500 kg to 550kg) and *Karhani dhan* production will be 4 quintals (400kg).

He wants to continue but he will not grow in more than 1.5 acres.

Seeds spread from him - 2 people in the same village 10 kg each in 2004 against *Lal Gandhari* seed. In Bajha 8-10 farmers have been cultivating Ashoka. Its not spreading because the growers cannot give seeds to others as for them its even less.

Concerns

- He feels that "hum logo ka hi laparwahi tha, jaisa humko mila tha pahale yisahi dusro ko bhi hum dete apne se to thik raihata. Uske badle me hum to khud hi pura kha gaye hai" [Means it did not spread because of their negligence and as they got seed from GVT they should have shared with others after harvest so that number of Ashoka growers would have been more, instead most of the original growers consumed it entirely].
- As it was distributed in 2003 to a few farmers it should be further distributed to other farmers.
- Because of Drought in 2006 some other growers could not save even seeds and stopped growing.
- The area where Ashoka is grown is slightly isolated and other large number of farmers do not go there hence storing could not be possible with large section of farmers.
- He had suggested that there were large number of tribal farmers in the area who always prefer traditional varieties, hence seeds should have been distributed among the tribal farmers then it might have spread.
- Otherwise the local farmers needed more counseling and advocacy and then only they would have shifted from hybrid varieties to Ashoka. There should be more campaign on benefits of Ashoka.
- There are local agents for hybrid varieties and even the dealers are campaign for hybrid varieties but no such campaign for Ashoka, or seeds available.

- There was no monitoring and follow up from GVT. They only distributed seeds and there were no queries for next year related to production, problems and benefits. If there had been some follow up from GVT's side then farmers would have taken it seriously.

4. *Kherika,* GP Bajha, Block Katkamsandi, District Hazaribagh, Jharkhand

Dr Dey

**Date**	25 and 27 March 2009
**Methods**	Observation, FGD (2), IDI (4)
**Participants**	Ashoka growers, Non growers and Drop outs
**Qualitative Study team**	Dr Aniruddha Dey, LFC India and Mr Albert Xalxo, India – Field Support Officer, supported by Mr Vinay Tomar and Mr Sanjeev Ku Singh, representatives from GVT East.

Participants: Anil Tuti (Grower), Lajrush Tuti (Drop Out), Mahesh Paswan (Grower), Sunil Munda (Grower), Vinod Paswan (Grower), Sarju Paswan (Grower), Rajinder Paswan (Grower),

Women – Liladevi w/o Mahesh Paswan (Grower), Dhanua Devi w/o Chander Yadav (Grower), Saloni Konk w/o Anil Tuti (Grower), Sushana Nag w/o Lajrush Tuti (Drop Out), Chitu Devi w/o Sunil Munda (Grower), Munia Devi w/o Bijay Yadav (Adopted), Rina Devi w/o Ajay Yadav (Adopted)

Ashoka rice was introduced in 2004. They call it *"Charkha Goda"* because its grown in Up land (*Tanr jamin*) like other *Goda* rice (traditional upland varieties of paddy) and *'Charkha'* because of white grains. No irrigation / watering required. There was no other exotic rice variety to grow in the *Tanr Jamin.* Earlier they used to grow *"Lalka Goda"* (indigenous species), grains of which were red in colour. But when Ashoka was introduced it was also of the same duration and similar in terms of process of growing but different in terms of colour and taste. So they started growing Ashoka.

Upland is known as *Tanr jamin* – 60 percent of the entire agricultural land

Medium land is known as *Tarkha jamin* - 40 percent of the entire agricultural land

Low land is known as *'Khet'/ 'Gahera Jamin'* - Nil

In **upland** they grow the following crops:

**In Rabi season**			
CROP	% of land	Time of sowing	Time of harvest

Sarso (Mustard)	10	*Aswin* (Sep. end.-mid Oct.)	*Magh* (Jan. end.- Mid Feb.)
Matar (Green Peas)	3	*Kartik* (Oct.-Nov.)	*Fagun* (Feb-March)
Desi Chana (Chick Peas)	2	*Aswin* (Sep. end – mid Oct)	*Chait* (Feb – March)
Tomator (Tomato)	15	*Kartik* (mid Oct –Nov)	*Chait* (Feb. end – end March)
Aloo (Potato)	5	*Aswin* (Sep. end – Oct.)	*Magh* (Jan. end – mid Feb)
Kulthi (Horse Gram)	15	Do	Do
Sarguja (Oil Seed)	25	Do	*Pus* (Dec. end – mid Jan)
**In Kharif Season**			
Arhar (Pigeon Pea)	5	*Asadh* (early May – mid June)	*Aghan* (mid Nov.– end Nov)
Urad (Black Gram)	10	do	*Bhado* (Aug. end – early Sept)
Marua (Millets)	4	*Saon* (early July -mid July)	*Aghan* (mid Nov.– end Nov)
Makai (Maiz)	6	*Asadh* (earlyJune – mid June)	*Aswin* (Sep. end.-mid Oct.)
Tomator (Tomato)	35	Saon (early-Mid July)	Kartik (mid – end Oct)
Paddy – *Ashoka*	15	*Asadh* (mid Jun – end June)	*Aswin* (end Sep. – Mid Oct)

Note: Ashoka maximum grown in lower part of the up land and medium land. In March, April and May land remain fallow.

In **Medium** land they grow only different varieties of paddy

Only six families of the village have low land.

2004 was the trial period for Ashoka. It found suitable, production was more in

Mainathori (Indigenous)	15	*Asadh* (mid Jun – end July)	*Kartik* (early Oct - mid Oct)
Charka Goda / (Ashoka -Exotic)	25	*Asadh* (June end-mid July)	*Aswin* (mid Sep. – early Oct)
Karhani (Indigenous)	5	*Saon* (early July-mid July)	*Kartik* (end Oct-mid Nov)
IR 36 (HYV)	10	*Asadh* (end June -mid July)	*Kartik* (early Oct-mid Oct)
IR 64 (HYV)	10	*Asadh* (end June-mid July)	*Kartik* (end Oct-mid Nov)
Anjali (HYV)	10	*Asadh* (end June-mid July)	*Kartik* (early Oct-mid Oct)
Bhera Kabar (Indigenous)	10	*Asadh* (end June-mid July)	*Kartik* (end Oct-mid Nov)
Ratgoli (Indigenous)	5	Do	Do
Pioneer (HYV)	10	Do	Do

comparison with *Lalka Goda*. In 2005 climate was suitable/favourable (same as 2004). After seeing the result two villagers started experimenting Ashoka for the second time in low land, which was sown in end of February and harvested in May. It was an extra crop which was not possible with other varieties. Production was more than usual, grains were matured and number of false grains (*Khakhri)* were less. (Though two persons reported to have experimented but one person informed that later on he realized that by mistake he put some other variety).

In 2006 less rainfall and all crops affected, drought (*akal)* was declared in the area. Only those who grew Ashoka got some amount of paddy, which at least could be used as seed for the next season. But in other varieties there was no production.

In 2006 Ashoka was not grown in February because of the problem of grazing – since all land remain fallow people allow free grazing during that time and the farmers who had experimented it earlier could not repeat as they were unable to sort out the problem of animals to eat the crop.

In 2007 there was adequate rain fall. They thought in 2007 also there would be drought, hence the farmers grew only Ashoka rice seeing the result in 2006. Production was good and more than satisfactory. In February again they did not grow because other land was fallow and free grazing of animals was the problem hence they dropped.

In 2008 less rain fall but Ashoka's production was not affected. *Mainathori*, *Anjali*, *Bera Kabar*, *Karhani* varieties production was less.
Since Ashoka gets matured early so climate did not affect the production much.

Table B: Benefits of Ashoka

- Early variety / short duration paddy
- Production is more in comparison to other varieties
- Less water required
- Branching more - six to eight branches from one seed
- Bunch of stems are developed and thick.
- Grain section / pinnacle is taller
- More grains in pinnacle
- Do not use any fertiliser and only cow dung (FYM – Farm Yard manure) used
- Not laborious, same as *Lalka Goda*
- They have stopped growing *Lalka Goda*
- Better quality fodder, fodder soft and smooth, animals prefer to eat much.

Number of Ashoka growers

In 2004 they received from GVT 30 kg seed in a packet. 15 members of male SHG distributed the seed amongst themselves and all of them got 2 kgs each. Almost 50 percent of the recipients gave their seeds to other members, who were willing to experiment and the willing farmers got more seed to grow. Lajrush Tuti said he got 4 kgs extra from Vijay Yadav and another member (could not remember the name). According to Lajrush Tuti since the seeds was supplied free of cost some of the members were not convinced about the potentials and they were not willing to grow and at the same time the farmers who were willing to grow the new variety took this as an opportunity as they could grow more seed in their land. Later on in 2007 some of those same SHG members took seed from the original growers as they saw the

result that even in 2006 when the area was declared as drought hit area only Ashoka growers got some yield (eg. Same Vijay Yadav took seed from Lajrush Tuti), similarly some other non-growers also adopted Ashoka and took seed from the fellow villagers. However, some of the regular growers could not save sufficient seed in 2006 and they took seed from the fellow villagers (eg. Sunil Munda got 5 kg seed from Anil Tuti and production was 1 qtl. However, it was confirmed that none of the villagers of Kherika got seed from outside.

Distribution

Name of the Farmer	Distributed to	Place	Distance	Year
Anil Tuti	Sister 20 kgs	Khorhar	65 kms	2005 (Adopter)
	Sister 20 kgs	Chatra	40 kms	2005 (Adopter)
	Unty10 kgs	Within Village	0 km	2005 (Adopter)
	Sunil Munda 5kgs	Within Village	0 km	2006
Lajrush Tuti	Sister 20 kgs	Jori	8 km	2007 (Adopter)
	Diyali Yadav, Neighbour 20 kg	Uridiri	1 km	2007
	Kaila Yadav, Neighbour 6 kgs	Within village	0 km	2007
	Vijay Yadav, Neighbour 15 kgs	Within village	0 km	2007 (Adopter)
Mahesh Yadav	Neighbour 10 kgs	Within village	0 km	2007 (Adopter)
Sarju Paswan	Neighbour 6 kgs	Kathkumsandi	1 km	

Concerns

GVT stopped project. Now they are not getting any suggestions beneficial for other livelihood support. They are not empowered and not very confident to liaise with

bank and other agencies. For example pigs were purchased through bank loan last time pigs died and bank is pressing them to repay loan. When GVT was there they went to other places for exposure visits. GVT as a facilitating agency they feel is still required.

Production

In this village without fertiliser *Ashoka* grown in 1 Katha = 65 kgs

Lalka Goda in 1 Katha = 30 kgs

Lalka Goda remains red even after milling.

Rice soup of Ashoka is also tasty and people like it most after hard work in the field. Almost all of them said that even if they don't have other vegetables irrespective of their age people like to take cooked Ashoka rice with soup and it is less expensive as pulses are not required.

They have never tried transplanting Ashoka but some of them said they have seen in other villages farmers have done this and they will try this once, because then weeding will not be required.

DROP OUT – Lajrush Tuti (56), non matric, working as a mason and also doing farming.

Kins	Age	Sex	Education	Occupation	Staying at
Wife	42	Fe	BA	Service - Primary Teacher	Village
Son	35	M	BA	Service – Police	Kodarma
So's Wi	28	Fe	Non mat	HW	Do
Da	20	Fe	Nursing Training	Apprentice	Patna
So	22	M	BA	Student	Patna
So	18	M	IA	Student	Patna

Own land 4 acres: *Tanr* – 1 acre, Medium – 2 acres and Low – 1 acre

Started growing Ashoka in 2004 in 0.6 acre medium land and production was 1.8 qtls. In 2005 in 1.5 acre medium land and production was approximately 12 qtl.

He wanted to grow in February and accordingly low land was prepared but by mistake grew another variety since seed he could not identify and it was *Taichun,* which was brought by him from Jori village in exchange of other local varieties.
In 2006 again in 1.5 acre, but there was a drought and only got double the seed sown. He tried in February again but there was no germination as it was very cold.
In 2007 dropped.
He brought *Anjali*, because he felt that growing the same variety in the consecutive years would led to less yield and hence started growing *Anjali.*

Distribution

Jori – to sister –8 km – 20 kgs in 2007

Uridiri - Diyali yadav – 1 km – 20 kg in 2007*

Within village - Kaila yadav - 6 kgs in 2007*

Within village – Vijay Yadav -15 kgs in 2007*

**Because of drought they could not save seed.*

Anjali's production is also good but not as good as *Ashoka* in comparison to taste, but *Anjali* is laborious. But he will again shift to *Ashoka* after 2 years.

Paddy	Market (Rs)
Ashoka	Rice 1100/- qtl and paddy 800 /- qtl
Anjali	Rice 1000/- qtl and paddy 700 /-qtl

Grower and Trendsetter: Anil Tuti (32), Non- Matric, carpenter - farmer

Total seven members' family

Land 1.5 acres – *tanr* 0.50 acre, *Tarkha* 0.70 acre, *Gahari* land 0.30 acre

Distributed Ashoka Seed to

1. Chachi (Unty) – within village – in 2005 – 10 kgs
2. Sister – Chatra – 40 km – in 2005 – 20 kgs
3. Sister – Kharsoa – 65 km – in 2005 – 20 kgs

(No information about the production in both the sister's land was available. They wanted seed after seeing the result then they were offered with knowledge and technology).
He wanted to experiment with transplanting.
Anil Tuti informed that in 2003 a very small amount of seeds was distributed as a trial only by GVT. Anil Tuti (32) had experimented with that and he was satisfied with the production. By using only 500 g seed he got approximately 65 kgs paddy. He has

used the same seed till 2007 and there has been no change in the production. In 2007 by mistake all grains were boiled and no seed could be saved. In 2008 12 kg *Ashoka* seed in exchange of 12kg *Anjali* seed from Deyali Yadav of Uridiri (neighbouring village) was brought.

After seeing the result Anil Tuti started experimenting Ashoka for the second time in low land, which was sown at the end of February and harvested in May. It was an extra crop which was not possible with other varieties. Production was more than usual, grains were matured and no of false grains (*Khakhri)* were less. His uncle Lajruish Tuti also did the experiment but by mistake he put another seed.

In 2006 he did not grow Ashoka in February because of the problem of grazing – since all land remained fallow, people allowed free grazing during that time and he could not repeat as he was unable to sort out the problem of animals to eat the crop. He said time was required for other varieties, otherwise there would be no germination but Ashoka was harvested in October and again sown in February end and germination was proper – very good observation.

Apni atma se main kahe sakta hun ki koi bhi kisan ise pasand karega aur akal mein bhi kuch na kuch fasal milega (He said from the core of heart I believe that any farmer would like this variety as even in droughts also people would get some yield)..

Adoption: Vijay Yadav (35)

Started growing in 2008 and got seed from Lajurish Tuti

He was one of the recipients of 2 kgs Ashoka seed in 2004 as a member of the SHG but he was not convinced and did not grow. He had given his seeds to Lajurish Tuti in exchange of HYV for his low land. After seeing the results in the consecutive years and also the remarkable result of Ashoka in the drought year in 2006 when no other crops grew he and his family members started developing interest and finally brought 15 kgs seed. His wife Munia Devi (31) said its ‘A’ grade paddy.

Moreover, Vijay said that in the village Anil Tuti and Lajurush Tuti have very good influence and they can be approached any time, hence when they saw the result they have shown interest in Ashoka and approached.

Before this they used to grow *Dehati – Lalka Goda* variety and production of which was less than half of Ashoka (they have not measured). Ashoka is tastier, fine grains and they have liked it.

Grown in upper part of medium land, applied broadcasting method.

Non Grower: Chander Yadav (50)

Own land 10 katha 50% *Tanr* land and 50% *Tarkha* land (upper portion of medium land)

He grew only *Lalka Goda* in *Tanr* land and production was 18-20 kgs per *katha*

He has seen Ashoka in Kaila Yadav's land and was impressed after seeing the growth and health of pinnacle but he did not know the variety and never asked Kaila because of enmity. He also did not know how to get the variety. In the meeting he could share with other growers and expressed interest.

FGD with Women:

Women – Liladevi w/o Mahesh Paswan (Grower), Dhanua Devi w/o Chander Yadav (Grower), Saloni Konk w/o Anil Tuti (Grower), Sushana Nag w/o Lajrush Tuti (Drop out), Chitu Devi w/o Sunil Munda (Grower), Munia Devi w/o Bijay Yadav (Adopter), Rina Devi w/o Ajay Yadav (Adopter)

Division of Labour:

Seed priming – women [Process – soaked in water for the whole night/ remove water in the morning/ allow for germination – (*Bora Jhapi* method – keep the soaked seeds in basket which then covered with sack), germination starts after two days.

Ploughing and levelling - men

Broadcasting - men

Weeding – women (3-4 days)

Manuring – men (before broadcasting DAP and after weeding urea)

Harvesting – men and women (mostly women)

Carrying to Khalihar – men

Thrashing – men

Winnowing – men

Boiling – women

Husking – women

Milling – men

In comparison to *Lal Goda* though process remains the same but husking Ashoka in the traditional method is easier. Boiling time for *Lal Goda* is also more, hence more time and fuel required.

In upland Ashoka is the best. In medium land mostly they grow *Mainathori,* because only that variety seed was available at home. They have related Ashoka as "*Bin Pani ka Dhan"* – Rice variety with less water required.

Ashoka is tastier, all other varieties they grew are course. When guests come they also prefer Ashoka rice. They don't want to take other variety and say "*ghar mein bhi to mota chawl khate hai, mehman banke aaya tab bhi ohi mota chawl khilayenge?* (guests expect Ashoka rice as they eat other coarse varieties when they are at home).

Those families grow different varieties they consume other varieties everyday and keep Ashoka for lien periods. In this village rice production is less and they don't sell and only consume.

Since last year transplanting started, production is more and requirement of seeds less.

5. ***Uridiri,* GP Uridiri, Block Katkamsandi, District Hazaribagh, Jharkhand**

Dr Dey

**Date**	25 March 2009
**Methods**	IDI (1)
**Participants**	Ashoka grower
**Qualitative Study team**	Dr Aniruddha Dey, LFC India and Mr Albert Xalxo, India – Field Support Officer, supported by Mr Vinay Tomar and Mr Sanjeev Ku Singh, representatives from GVT East.

Uridiri was also not selected as a village for conducting the qualitative study but since we found one GVT woman staff, who was deeply involved in the process, the team thought of conducting an In-depth Interview. Interview with Smt. Rina Devi (35 years), non matric worked as "Dissemination *Jankar*" [animator] in GVT from 2002 to 2004. Before that she was a leader of one women SHG [Sudha group – Bero] active SHG members were selected as Dissemination Jankar by GVT.

Her family background

**Kins**	**Age**	**Education**	**Occupation**
Her husband	4o	VII	Mason and agriculture
Her son	2o	BA	Student
Her daughter	14	IX	Student
Her son	1o	V	Student

Her Job description: Formation of new SHGs, knowledge sharing with the members, facilitating and monitoring the groups in 2 villages Uridiri and Gardoba.

Approach: There was a system. In the area all animators used to meet with the supervisor once in a month. There were 2 way interactions in the meetings.

- Animators – informed the supervisors about all the activities in the villages, like what crops people were growing, what were the different plans to execute in terms of agriculture and all other activities.
- Supervisors – informed about the GVT activities and plans to the animators, trained them so that the animators could implement those activities and shared knowledge and processes with the villagers.

Responsibilities of the Dissemination *Jankar* for motivating people to grow *Ashoka* rice: Meetings were conducted in the primary villages by the GVT staff and animators were given the responsibility to disseminate knowledge in the secondary villages.
In 2004 she got only 30 kg seeds from GVT. She had distributed 15 kg seeds in each group of in two villages. At Uridiri village she gave 10 kg seeds to men groups and 5 kg to women groups. At Gardoha village she had distributed 10 kg *Ashoka* seeds to the leader of the men group and 5 kg to the leader of the women group.

Knowledge: In July 2004, the farmers first grew in the respective villages. It was not in the up land but in the land where some amount of water was available, mainly moist areas. They prepared the land after ploughing and levelling and then broadcasted the seeds. Used one kg DAP, 1.5 kg Urea per katha [1 katha = 4 decimal]. Seed priming was done in both the villages. Seeds distributed considering those who were willing and at the same time have got suitable land.
Characteristics of Ashoka according to her:-

- Less water required
- Early variety
- Tasty
- More price if sold
- Less laborious mainly during processing of paddy (boiling) after harvest
- Less fuel required.
- Less time for cooking
- People like to drink rice water as soup.

Best result from Uridiri because of more amounts of suitable land then Gardoha.
It was asked whether there was any relation with education level of the farmers in accepting the variety. In response she said uneducated farmers know more about farming then educated people.
She also grew Ashoka rice by broadcasting in the following years:

Years	Area covered	Type of land	Seeds in kg	Seeds were own/ GVT	Production
2004	4 decimal	Medium land	3 kg	GVT	1 quintiles

2005	16 decimal	Medium land	12 kg	Own	4 quintiles
2006	20 decimal	Medium land	15 kg	Own	4.2 quintiles
2007	20 decimal	Medium land	15 kg	Own	4 quintiles
2008	60 decimal	Medium land	45 kg	Own	11 quintiles

Seeds were not shared with anybody.

In 2008 for the first time she sold 4 quintiles *Ashoka* @ Rs 800/ quintile. No rice varities were grown in mid land. Previous year in rest of the mid land they used to grow tomatoes and other vegetables which were laborious and demand constant monitoring but since 2007 she started growing *Ashoka* only in all land.

In the low land hybrids rice varieties are grown also sold at same price. Like, *Lalat* they grow but other varieties in mid land yield less with less value in the market (@ Rs 5 to 6 per kg). Rests are same in taste and other things.

Was it beneficial to poor? And if so how?

Earlier people used to grow *Lalka goda* but production was very less (50 kg in 4 decimal) and in the same amount and type of land (4 decimel) *Ashoka's* yield was almost 4 quintiles. It implies that food sufficiency increased to those who have suitable land.

Species grown in low land were more expensive as it required more fertilizer and pesticide. In comparison to *Lalka goda* in mid land *Ashoka* grown well if same amount of DAP and urea used.

Suggestions:-

- Awareness campaign and knowledge sharing programme needs to be launched to involved others
- Seeds are not a big problem
- Monitoring and follow up should be there. This would help understanding the feelings of the farmers.

6. *Kud,* GP Danto, Block Katkamsandi, District Hazaribagh, Jharkhand

Dr Dey

**Date**	26 March 2009
**Methods**	Observation, FGD (2), IDI (1)
**Participants**	Ashoka growers, Non growers and Drop outs
**Qualitative Study team**	Dr Aniruddha Dey, LFC India and Mr Albert Xalxo, India – Field Support Officer, supported by Mr Vinay Tomar and Mr Sanjeev Ku Singh, representatives from GVT East.

Kud was selected as a sample in Hazaribagh District, Jharkhand for qualitative study on Ashoka rice under the category of secondary villages. In the quantitative study report this was shown as a secondary village of Bajha (P3), where Ashoka rice was grown in 6.6 acre of the 25.80 acre total rice area in medium land, meaning 25.6 percent medium land was under Ashoka rice and the report also mentioned that 54 percent households were growing Ashoka.

After entering into the village the team first contacted Mr Kishore Kumar Rana and the purpose of visit was discussed. With the help of Mr Rana the team could meet some other male members available in the village and the first FGD started with 10 male members. Two FGDs were conducted in the village. In the first FGD only men (10) were present. Information collected on demographic profile, socio economic status of the people, livelihood issues and programmes, etc. by using different PRA tools and techniques like social mapping, Venn diagram, matrix analysis, seasonal mapping, cohort analysis, etc. From the FGD two growers but drop outs were identified for in-depth interviews. Second FGD was with the tribal women (13) of both grower and non-grower families, where primarily gender issues, awareness on Ashoka rice, decision making, migration, education of the children etc. were emphasised.

Village Kud is surrounded by Bajha in the East, Danto Kala in the South, Edla and Kadotia are in the North and Danto Khud in the South. This village has 8 hamlets, which are as follows:

- Katgharwa Tola (2 HH),
- Surjhahi Tola(18 HH),
- Pisraba Tola (6HH),

- Oraon Tola (5HH),
- Bhuiya Tola (10HH),
- Badai Tola (17HH),
- Bhokta Tola (35HH) and
- Kodiya Tola (8HH).

There are 4 types of land which are as follows:

A. *Tanr jamin,* which is also referred as 4 *number jamin* (type 4 is the upper part of the up land, which is situated in the foot hills and non-cultivable).
B. *Tarkha jamin,* which is lower part of the up land and referred as 3 *number jamin* (type 3 and rainfed).
C. Medium land, which is also referred as *2 number jamin* (type 2 land). It is though rainfed but irrigation can be possible and from this place *kshet* (cultivable land) starts.
D. Low land, which is also referred *as 1 number jamin* (type 1 land) because of its water retention capacity, where they can grow rice, which is their staple food and at the same time by growing HYVs in such land they could generate surplus for sale in the market.

Although as per record it was a secondary village of Bajha but it was confirmed by the participants in the FGD that in 2003 seeds of Ashoka rice were distributed to 17 women SHG members of Bhuian Tola. It was informed that poorest of the poor families were given importance and first seed as sample was given to Bhuiyans (tribes). All of them received 2 kg each. However, since the sample given was inadequate, Prakash Rana and Kishore Kumar Rana borrowed the Ashoka seeds 10 kgs each from their relatives at Bajha in the same year after hearing the benefits of growing Ashoka and 2 person Krishna and Prakash bought 10 kg each from Bajha village from their relatives.

Special features of *Ashoka* rice as described by the people in the FGD:

- Pinnacles are long
- Grains are long and thin
- It is tasty to eat
- Mature early than other local varieties [within 80 days]
- Sowing time is June and Harvest in August
- Double in production of *Ashoka* in comparison to *Dehati Goda Rice* (Traditional upland rice variety)
- If fertilizer applied and weeding done properly on time yield can be 3 times more
- Easily digestible.

Other benefits of Ashoka:

- ✓ Early variety hence other Rabi crops like potato, cabbage, tomato, wheat, gram, mastered and green peas are also grown after harvesting *Ashoka.*
- ✓ In the area maximum food security from their own land was estimated 8 months and for the marginal farmers it's very less, not even for three months. However, most important part in Ashoka, as people said that many people have got the opportunity to grow one extra crop and during the usual lean season Ashoka could be harvested. During *Dusherra (the main festival of the Hindus),* now at least they have some food to eat.
- ✓ This *Ashoka* rice grown on fallow land where no other crop generally could be possible.
- ✓ *Ashoka* rice required less time to cook and hence economic, as it saves fuel.
- ✓ In type 3 land *Ashoka* can be grown without any fertilizer but in type 4 land fertilizer [DAP+urea] required.
- ✓ Straw of *Ashoka* is soft and light then *Lal Goda dhan* and animals prefer it.
- ✓ The seeds of *Ashoka* are reusable. People said that they have been using their own seeds since 2004 and it has not change the production.
- ✓ It's also a drought resistant variety.

Cultivation of Ashoka rice and Traditional upland variety (in percentage) since 2003

**Years**	**Ashoka Rice**		***Lalka Goda*** **- Traditional upland rice variety**
	**Area covered in percentage**	**Own/ borrowed seeds**	**Area covered in percentage**
2008	80%	Own	2%
2007	40%	Own	5%
2006	30%	Own	10%
2005	10%	Own	30%
2004	5%	Own and some	40%

		from Bajha	
2003	2%	Seeds distributed to 17 Women SHG members and two persons borrowed from Bajha.	60%

Estimated expenses of cultivating Ashoka rice:

**Process**	**Expense per acre**
Ploughing 1 acre land [one day 4 plough x 4 times @ 150 x 4 x 4 = 2400]	2400.00
Seeds required for 1 acre = 50 kg	[no need to buy]
Weeding [need at least 25 persons]	1250.00
Harvesting	1250.00
Thrashing	400.00
Milling / Husking	500.00
**Total**	**6400.00**

Production in 1 acre = 10 quintiles. Those who sell the finished rice after milling get INR 6000.00 for 10 quintals.

Depending upon the availability of water in type 3 land some farmers experimented with transplanting and this was beneficial to farmers because

- ✓ Less laborious for weeding means less expensive. Sometimes weeding not at all required,
- ✓ Less amount of seeds required,
- ✓ More production (almost double than broadcasting).

It was informed that Prakash Rana has given 6 kg seeds to Mohan sing of Bhokta Tola, Kud in 2005 in exchange of other variety of paddy. Bijay Rana has given 5 kg seeds to his uncle Kariyasan – 12 km away from Kud, in 2005.

However, the participants have suggested a few points for poverty eradication, which are as follows:

- More emphasis on vegetables as cash crop should be taken.
- Selected farmers should be given intensive training and they should be treated as the task force in agricultural development activities in the village. Other would take lessons from them.
- Ashoka seed distribution was one activity but other livelihood resources and activities should also be identified and introduced simultaneously for poverty reduction.
- Facilities on agricultural counseling and Integrated Paste Management (IPM) should be considered seriously.
- Watershed development activities should also be taken.
- Farmers should be facilitated to access formal credit, which would help them expand their agriculture activities.

The members informed that Ashoka has spread in the village through relatives, friends and neighbors but it was difficult for them to say specifically who has given to whom and how much, but it was sure that Ashoka has spread over in all the nearby villages. Prakash Rana informed that he has seen the tribals were selling Ashoka seeds in *Dato* (10 km from the village) market. He asked the vendors and confirmed that it was Ashoka.

It was also reported that some people have stopped growing Ashoka and when asked one such farmer, Ganesh Rana (35 yrs) informed that they have 5 acres family land (1.5 acre type 4 land and 3.5 acre type 1 land) and for two reasons they have stopped growing Ashoka. Firstly, all the adult males are carpenters (traditional activity) and work outside. They work in their agricultural land only in monsoon and put all efforts in growing

HYV in type 1 land. Since carpentry is more profitable they definitely never considered growing Ashoka in the Rabi season and Secondly, from the type 1 land they get enough yield to meet their consumption needs.

FGD with Women growers (12) of Bhuyan Tola

They have 17 members in the Mahila Mandal. In 2003 members of the group shared 30 kg seeds among themselves and broadcasted in type 4 land. Their land was in cluster and actually it was the ancestral property and divided later among the successors. All members have sown in one plot. Finally they produced 360 kg [15 *mon* and 1 *mon* = 24 kg]. Grains were accumulated in one place after harvesting and then taken their individual share. All of them first taken out the seeds for the next year and consumed the remaining part. Only Kaliya Devi gave seed to three other women (Sarita - 6 kg, Kusuma - 3 kg and Kapurua – 9 kg) of the village in exchange of assurance of labour and sold only 24 kg in market @ 150/-*mon* = 150.00 and purchased other essential ingredients for cooking. Some women also took land on lease for cultivation in exchange of one-third of their production. Besides this, these women go to work in others field as agricultural labor and in turn they get only 6 kg of paddy per day and no cash.

In type 3 land farmers grew through transplantation and production was 5% more even though seeds were sown less - only 2-3 seedlings were transplanted together.

Differences felt after the introduction of *Ashoka* rice

- ✓ Tasty
- ✓ Early variety
- ✓ Little effort gives more production
- ✓ Only Rice which can be grown in *Tanr* land
- ✓ Even if they don’t have any vegetables or pulses only rice can be consumed with a little salt. It is so tasty that even rice with soup can be taken without any side dish.
- ✓ Cooking time is less hence they don’t have to wait for long before going for the day’s work, moreover, fuel consumption is also less.
- ✓ It grows at a time when most of the families do not have anything to eat and mostly they buy everything. Since they get their harvest during the usual lean period they feel very happy.
- ✓ This helps in increasing their number of working days as agricultural labors – at least 15-20 days.

- ✓ Even the marginalized farmers also get food for at least 2 more months. Three members said earlier they used to buy rice for 6-7 months and after the introduction of Ashoka it has reduced and now they are buying rice for maximum 3-4 months.
- ✓ Farmers can grow another crop after harvesting Ashoka. It was informed that After Ashoka they can grow two crops – intercropping wheat plus *chana* (gram) to meet their consumption needs.
- ✓ There is a change in their status in the family. Seeds were given to the members of the women SHGs and they got a very good result is the most important thing.

However, they said that all the male members of the Bhuyan Tola migrate as industrial labor to different cosmopolitan towns and its common in the entire village. People generally starts leaving in the middle of January and again come back in June – before monsoon and participate in the agricultural activities with other family members.

***IDI* - Kaliya Devi,** age 45yrs, literate from SHG night school, work in agriculture field and wage labor, her family profile is as follows:

SN	Relationship	Age	Education	Occupation
01	Husband	49	illiterate	Agriculture and migrate labour
02	Son [married]	27	III	Migrate labor
03	Son's wife	24	Literate	House wife and work in the family agricultural field
04	Son	20	IA	Student at Hazaribagh College and stay in a rented house
05	Son	18	IA	Do
06	Grand son	2		Going to ICDS centre
07	Grand daughter	1		

She has only 25 *Katha* Type 4 land for Ashoka rice and never experienced loss. Traditional upland paddy varieties are not beneficial hence using the entire type 4 land for Ashoka. Never experienced loss. She took up the agricultural activities since 2002 and in 2003 they got Ashoka seeds from GVT. She was very keen to follow the instructions and also led other women members. She said Ashoka helped to increase her confidence and she is now in a position to convince her family members and can decide what to grow when. Before she took the initiatives her in-laws never cultivated the type 4 land, rather they used to go for wage earning, collection of forest produce, etc.

Year	Seeds in kg	Production in *mon* [1 *mon* = 24 kg]
2005	9 kg	9 *mon*
2006	12 kg	12 *mon*
2007	12 kg	12 *mon*
2008	12 kg	12 *mon*

Difference felt

1. At first it was not given importance
2. But finally it added food security for 2 more months
3. Due to early variety they get time to grow other crops.
4. Difference was in decision making.
5. Said for eight members in the family they require at least 6 kg rice per day, which costs minimum Rs 60.00 per day and in 30 days its approximately INR 1800.00 and if they save for 2 months at least then it's a saving of INR 3600.00 to INR 4000.00, which three adult members in her family earn in 33-34 days, in a period of at least three months.
6. They get extra fodder also for animals for three months.
7. *Lal Goda* was not very beneficial so now they have almost stopped growing.
8. Ashoka rice is insect resistant and they have never used any insecticides.

- ✓ Question was asked that whether *Ashoka* has any impact in their family economy and she said it would not have been possible to send her children for higher studies and they would have gone for wage earning in Banglore, Mumbai, Delhi, Kolkata etc. places. Moreover, alcoholism decreased with children's education as the children are conscious and not allowing their parents to consume liquor.

- ✓ Migration could not be stopped as *Ashoka* was not very labor intensive and land for growing Ashoka was also less. She can do this alone. When they men-folk starts coming during monsoon that time is considered as crisis period in terms of availability of labor and the men work as agricultural labour.

7. *Hutkona,* GP Hutkona, Block Katkamsandi, District Hazaribagh, Jharkhand

Dr Dey

**Date**	27 March 2009
**Methods**	IDI (1), FGD (2)
**Participants**	Drop outs, Non growers, Dissemination *Jankar*
**Qualitative Study team**	Dr Aniruddha Dey, LFC India and Mr Albert Xalxo, India – Field Support Officer, supported by Mr Vinay Tomar and Mr Sanjeev Ku Singh, representatives from GVT East.

Hutkona was selected as a sample in Hazaribagh District, Jharkhand for qualitative study on Ashoka rice under the category of primary villages. In the quantitative study report this was shown as a primary village (P2), where Ashoka rice was grown in 2 acre of the 5 acre total rice area in upland and 33.3 percent households were involved, and out 65 acres of rice area in medium land 3 acre land was under Ashoka rice, where 27 percent households were involved.

***IDI* - Mundrika Devi (35 years),** illiterate, house wife and work in agriculture field.

Her family members are shown below:

Family member	Age	Education	Occupation
Husband [Umesh Pr. Mehata]	40	Illiterate	Agriculture
Son	18	Matric	Migrated labour
Son	15	VIII	Working as a cleaner in a Trekker
Son	13	VII	Student

In 2002 seeds supplied through women SHG [Saraswati Mahila Mandel and Bhogta Bikash Mahila Mandal]. She was not a member of any of the Mahila Mandals.

They saw the result in others fields and both the husband and wife decided to grow Ashoka rice in their upland in 2003. They were aware of the seeds availability.

Husband went for land preparation and by the time Mundrika went to collect seeds.

She offered money to Damyanti in exchange of Ashoka seeds but Damayanti accepted only *Dudh Kandar* seeds in exchange of Ashoka seeds. Mundrika took 2

kg seeds from Damyanti (her neighbour) and grew in more than 1 katha (4 decimal) and production was 70 kg.

Division of work between genders:

**Type of work**	**Responsible person**
Ploughing	Male
Levelling	Male
Weeding	Female
Harvest	Male and female
Thrashing [Dauri] by bullocks	Male and female
Boiling of paddy to prepare rice	Female
Husking	Female
Milling	Male

- In 2004 grown in 5 katha (20 decimal) land, which produced approximate 2 quintiles (200kg). This was unexpected and they thought that as they have grown their own seeds so production reduced since this generally happen in high yielding varieties. Since then they stopped growing Ashoka and started growing cash crops [vegetables – brinjal, tomatoes], which gives them return of Rs 5000 in one season from only 8 decimal land. It was found difficult to get any information on Ashoka rice from them as they were unable to remember *Ashoka* rice, but after repeating the type of paddy, GVTs role in distribution, name of the animator and GVT staff worked in those days and distributed one type of upland paddy etc. they could finally remember and relate their activities almost ended some 5 years back.

Own land: - 15 katha (60 decimal)

Upland – 10 katha (40 decimal)

Low – 5 katha (20 decimal)

Reasons to stop growing Ashoka rice and opinion about Ashoka

- There was no other reason but growing vegetables was more beneficial which could be grown in Upland (*Tanr jamin)* hence they have switched over to vegetable cultivation. During rainy season there upland becomes suitable for vegetables.
- Vegetable cultivation is beneficial because of its harvest period is prolonged and they could sell in weekly markets and got regular returns.

- According to them rice was white but not as good as the varieties produced in low land. Although they said that among the upland varieties *Ashoka* was the best and it was far better than *Lal Goda*.
- More time consuming in husking using traditional device.
- Tasty
- Less time consumption in boiling of paddy to make rice.
- No seed was distributed, all consumed.

FGD with Bhokta Bikash Mahila Mandel

Below is the list of members of Mahila Mandel.

1. Lalita Devi – drop out since 3rd year.
2. Masumar Jitni Devi – drop out since 3rd year.
3. Jitni Devi – non grower – not a member
4. Phulu Devi – drop out since 3rd year.
5. Khirodri Devi – drop out since 3rd year.
6. Kiran Devi – non grower – not a member
7. Parwati Devi - non grower – not a member
8. Mundrika Devi – non grower, grew only in first 2 years – not a member

In 2002, Ashoka seeds were distributed to 19 members at one kg each. Earlier they used to grow *Lal Goda*. *Ashoka dhan* is better then *Dehati Dhan* (traditional rice varieties) in terms of:

- Better production
- Better taste
- Long pinnacles
- White grain
- Less water requirment
- Better yield than *Lal Goda Dhan.*

They all started growing vegetables as they could see the immediate result. It was decided by the male members. Women did not play any role in decision making. They practice share cropping also.

In this village Babulal Sing Bhokta started cultivating vegetables and seeing the production and huge return others also felt attracted and switched over to vegetable cultivation. Basu Mahato was another villager who introduced the cultivation of cucumber in the village first

and that knowledge has been transferred, as a result now all farmers have started growing cucumber during rainy seasons.

However, they said that they have started realizing the fact that to earn more they have started using more fertilizers and pesticides with which land quality has started deteriorating.

The team could also interact with a few men and below is the list of male members present during the interaction:

1. Naresh Sing Nath Bhogta – first year he had sown Ashoka seeds in 3 katha (12 decimal) and got 70 kg yield.
2. Sreenath Sing Nath Bhogta – Drop out, grew Ashoka only in the 1st year
3. Umesh Sing Nath Bhogta – non grower
4. Indra Nath Mahato – non grower
5. Mohan Sing Bhogta – Dissemination *Jankar*
6. Baleswar Sing Bhogta - non grower
7. Umesh Prakash Mehta – non grower
8. Shisnath Prakash Mehata – received Ashoka seeds from Mohan Singh Bhogta and grew only in 2nd and 3rd year

Perception:

Depends on rainfall, if there is good rain production is also good. However, since its tasty its prone to flies (*Gandhi bug*), one type of grain borer which destroy the crops.

However, it was confirmed that nobody was growing *Ashoka* Rice in the village. In fact they all have stopped growing upland paddy varieties after the introduction of vegetables ciultivation in a large scale.

They are growing only vegetables now.
In the uplands they grow the following crops:

**Name of the crop**	**Sowing time**	**Harvest time**
Maize, Cucumber, ladies finger, brinjal, tomato, urad	Asadh (June- July)	Ashwin (Sep – Oct)
Green pea, mustard, pea, tomato,	Ashwin – Kartick	Fhagun – Chait (Mar –

potato	(Sep – Oct)	Apr)
Kulthi (pulses), Sarguja (oil seed)	Bhado (Aug – Sep)	Magh (Jan – Feb)

Crops grown in different types of land:

**Type of land**	**Name of the crops**
Type 4 land	Urd, Kulthi, Sarguja and Adhar
Type 1 land	HYV rice.

- Ashoka growers incurred loss in 2004 as it was a drought year and the entire crop dried and burnt and they could not even save seeds, which also compelled them to stop growing Ashoka.
- Almost 40 farmers in the village started growing Ashoka and in the 2nd year almost 35 farmers grew. But all of them were influenced after seeing the results in vegetable cultivation and stopped at a time.
- They have very good production of vegetables and they even take their produce to sell at Hazaribagh [only when there was bulk production] market, which is 22 km and nearest market [Wednesdays and Sundays] at Katkamsandi.
- They can grow vegetables in the up land (*Tanr jamin)* at least for 5 months and earn almost Rs. 5000/- per year per katha (4 decimal) while by growing *Ashoka* in the same land they could hardly earn Rs. 500/- per year.
- Since the introduction of High Yielding Variety (HYV) in the village, almost 20 households are getting rice for the whole year for their consumption by using the low land.
- Those who don't have low land buy rice using the money earned through selling vegetables.

Perception of Mohan

1. Pinnacle of *Ashoka Dhan* were attacked by flies which suck the milk of rice during its growth.
2. There was drought in 2004 and farmers suffered most.
3. Farmers did not show interest in growing *Ashoka Dhan,* instead they are more interested in cultivating cash crops [vegetables] which gave them more profit.

9. *Lakhnu,* GP Bajha, Block Katkamsandi, District Hazaribagh, Jharkhand

Dr Dey

**Date**	26 March 2009
**Methods**	IDI (1)
**Participants**	Ashoka grower
**Qualitative Study team**	Dr Aniruddha Dey, LFC India and Mr Albert Xalxo, India – Field Support Officer, supported by Mr Vinay Tomar and Mr Sanjeev Ku Singh, representatives from GVT East.

Lakhnu is the tertiary village of Kherika and secondary village of Uridiri. This was not initially selected for the study but when the study team was informed about one grower, who had also shown interest in seed supply, the team agreed to interact with that person. Accordingly, time was fixed with Mr Tushif Khan (35 yrs) and the team interviewed him.

He was a matriculate and including his children and wife they were 6 in numbers, owned 25 acres of land. In 2007 while going to Parbatori via Uridri he saw very good production of *Ashoka dhan* in one field, he stopped and contacted the owner of the land and enquired about the seed, availability, techniques, etc. and also requested the owner to give him some seeds. The farmer agreed to give him *Ashoka* seeds in exchange of *Sadma Dhan* (another traditional variety). Accordingly after harvest he was given 6 kgs *Ashoka* seeds, which was sown in the end of the July in 3.5 katha [14 decimal] land in lower part of *Tanr jamin* through broadcasting.

Process followed:

- ✓ 1st plough – after 1st plough land remained fallow for 2 months in order to destroy weeds. No fertilizer was used. During summer they put fire on the dry cow dung, which was spread over the plot before ploughing.
- ✓ 2nd plough – ploughing the plot in both horizontally and vertically and leveling done immediately.
- ✓ 3rd plough – after 3 to 4 days 3rd plough required. As soon as ploughing got over seeds were broadcasted and leveling was done.
- ✓ After 8 to 10 days germination took place.
- ✓ After 30 days weeding was done.
- ✓ After 60 to 65 days they do harvest [Oct end]
- ✓ Production was 4 *mon* (approximate one quintal).

No seed priming was done because there was already moisture in the soil. He was the first person in the village, who had grown *Ashoka* rice in the village. Farmers of the neighbouring villages saw his yield in the field and requested him not to consume it rather to use as seed. Almost 15 to 20 people made this request to him. Demands were from – Katkamsandi, Ratrua, Hutkona and Lukhnu.

In 2008 he had grown in 2 acres. It was slightly upper portion of the land used in the previous year. Sown 48 kg Ashoka seed and from this he got 16 to 17 quintiles rice. He had distributed seeds to his neighbors:

1. Pora Oroan 10 kg in exchange of local variety *Sadma dhan*
2. Dhanyyu Rana – 8 kg in exchange of local variety *Bhera kabab dhan*
3. Samali Ram – 10 kg in exchange of local variety
4. Mustak Khan – 12 kg in exchange of local variety

He said still there were 8 to 9 quintiles of *Ashoka dhan* with him even after consumption and he was thinking of selling them as seeds through which he would earn @ 12 /- per kg, which was higher than selling rice.

Perception of Ashoka qualities

- ✓ Even the land was dry but it grew and gave good yield
- ✓ Deep rooted, enlarged pinnacle, more grain holding
- ✓ It grows at a time which actually considered as lean season – for animal there was lack of fodder, people have to buy rice, only Ashoka growers are saved
- ✓ Animals like straw and according to him he saw some difference in milk production.
- ✓ Rice very tasty
- ✓ Rice soup is also very tasty.
- ✓ He wants to grow in 7 to 8 acres of land in the next year. And this year he will sell seeds in the market. He is confident that all the seeds will be sold.
- ✓ He felt he was more beneficial because of having maximum upland.

10. *Jaria,* GP Jaria, Block Bero, District Ranchi, Jharkhand

Dr Dey

**Date**	28 March 2009
**Methods**	Observation, GD & IDI
**Participants**	12 villagers (3 women) and three members from one Ashoka grower's family (2 men & 1 woman)
**Evaluation team**	Aniruddha Dey and Albert Xalxo supported two representatives from GVT East.

The team first decided to visit a place not selected as a sample village for the qualitative study, based on the result of the quantitative study in September 2008, to develop an understanding about the agricultural practices prevailed in the locality. Accordingly the GVT staff accompanying the study team selected Jaria, which was not in their list but the SHG members were distributed Ashoka seeds in 2003 by the GVT field staff from Katarpali village.

First we met and interacted with Shrimati Devi (29), who was married and staying with her parents with her 11 years son. She has studied up to Class VIII and actively involved in the agricultural practices with her father and elder brother. She was the leader of PUJA Mahila Mandal, an SHG promoted by GVT in Jaria and one of the recipients of Ashoka seeds in 2003, which she had grown in consultation with his father and elder brother for an experiment. His father Mohan Mahato (56) and her elder brother Suresh (24), a non-Matric were present during the interaction and active participants in the discussion.

Once the discussion was started a few other villagers (12 nos.) joined the meeting. From the discussion it could be revealed that since last two years there was no GVT staff and when they got the message there was a ray of hope that they might get associated with new livelihood development schemes. When the purpose of visit was made clear to them they started looking at each other helplessly as they all were non-growers. However it was known from the group discussion that lots of changes have taken place in the last couple of years. They have got electricity, village roads were good and well connected with Ranchi road (3 kms), all children were in school, after completing primary education (upto class IV) from the village school almost all children have been continuing upto class VIII in the Junior High School at Meso (2

kms), though very few then pursue for high school education and above. Girls' mainly suffer because the high schools and other educational institutions for higher studies were situated at faraway places and lack of security was the main reason for the girls' to discontinue. They have access to government health facilities and gradually people have started shifting towards modern medicine leaving their traditional healing methods because of unavailability of practitioners and natural resources. Livelihood status has changed for the land owners with the introduction of High Yielding Varities (HYVs) and access to seeds, technologies and markets. At the same time opportunities to work as agricultural labours have increased for the landless people. Village youths have got opportunities to work as mason, masonry labour, carpenter, daily wage labour for various other activities in the nearby villages, small towns and Ranchi, which has a direct impact in significant reduction of inter-state migration. National Rural Employment Guarantee Scheme also provided opportunities to the people 'Below the Poverty Line' (BPL) threshold to get assured income through wage earning mainly during the lean seasons. They perceived that connectivity played an important role in bringing change. After being benefitted through several livelihood development initiatives of GVT, people have adopted floriculture in the medium land, which was introduced three years back by Rama Krishna Mission – another NGO and those who have suitable land have earned reasonably good amount by selling 'Jarbera' sticks (flower used in decoration). People have started cultivating vegetables as cash crop during kharif season on the upland, which was remained fallow a few years back. Large families have started taking upland on lease from the small families for vegetable cultivation and in the process both were benefitted as the lessee used to give one third crop produced in a year to the land owner.

The villagers said they have adopted new agricultural practices and no farmer was using broadcasting method in paddy cultivation. In fact it was informed that they were facing labour problems during transplantation and harvest since broadcasting method has been replaced with transplanting method everywhere in the region and with the introduction of HYVs. They have started using chemical fertilisers and pesticides but they said they were totally dependent on the dealers or distributors of fertilisers and pesticides as they did not have adequate knowledge on requirements for different crops and in different situations.

Almost 80 per cent participants could not remember Ashoka rice and the rest of the participants could remember with a little clue and they in the first year only two families did the experiment and result was not satisfactory. Moreover, Ashoka rice was not a replacement of vegetables, hence they did not show any interest.

Different activities of paddy cultivation and division of labour of men and women:

Steps	Major Activities	Male / Female
1st	Preparing seed bed (*Bira*)	Male
2nd	Removal of seedlings (*Gachhi nikalna)*	Female
3rd	Ploughing and Levelling	Male
4th	Transplantation (*Ropa ropna)*	Female
5th	Weeding (*Nikai*) after 21 days	Female
6th	Using Fertiliser (*Khadh dalna*)	Male
7th	Spray	Male
8th	Harvest (*Katai*)	Female
9th	Carrying from field to *Khaliyan* (specially prepared field for thrashing)	Male and Female
1oth	Thrashing (*Misai*)	Male and Female
11th	Winnowing (*Osai*)	Male
12th	Storing of grains	Male and Female
13th	Boiling and drying	Female
14th	Husking	Female
15th	Milling	Male and Female

It was informed by the participants that men and women play equally important roles in agricultural activities. However, regarding decision making it was informed that generally the male folk consult their wives but they take the decision on the selection of crops and other agriculture related issues, like they take decisions on other family matters. Women agriculture labours were low paid in comparison to their male counterparts. If the wage for a woman agriculture labour was INR 35, then a man

would get almost INR 60 for the similar kind of work and time. This, they said was another important reason for crisis of agriculture labour during the peak seasons as the land owners would like to engage only women labours.

Thereafter, discussion continued with the family of the Ashoka grower. Family profile is as follows:

Family member	Age	Education	Occupation
Respondent (Primary)	29	Upto VIII	Cultivation
Father	56	Literate	Agriculture
Mother	48	Illiterate	House wife
Brother	24	Non-Matrick	Cultivation
Brother	19	IA	Student
Son	11	VI	Student

The family has 3 acres of land of their own, of which 0.5 acres low land (locally known as *Don/Gadda jamin*), 1.0 acre medium land (locally known as *Bari jamin*) and 1.5 acres up land (locally known as *Tanr jamin*). For share cropping they take upto 4 acres of upland on lease per year and give 1/3 of the product to the land owner. The respondent also does share cropping separately to generate her own sustenance.

In upland generally they grow the following crops:

Crops	Time of propagation	Time of Harvest
	August	Nov-Dec
Potato (*Aloo*)	August	Nov-Dec
Green Peas (*Matar*)	August	Nov-Dec
Bins	August	Nov-Dec
Tomato	August	Nov-Dec
Mustard (*Sorso*) - Kharif	August	Nov
Mustard (*Sorso*) - Rabi	Dec-Jan	Feb-March
Zinger (*Adrak*)	May-June	Feb-March

**Household with one year and more food security*

How did they get Ashoka seeds?

Jaria was one of the neighbouring villages of Katarmali, identified as a primary village (P4). There were 14 SHGs promoted by GVT in the nearby villages and

together they have formed a Federation and the centre of the SHG Federation was situated at Katarmali. All the SHG related training programmes and facilitations were conducted centrally from Katarmali where the SHGs of the Federation used to participate from their respective villages. It was reported that in 2003 all the groups of the Federation received Ashoka seeds in packets (30 kgs) for the first time. Thereafter the members of each group distributed the seed amongst themselves in equal quantity. One SHG of Jaria village received one packet (30 kg) of which only two members took that seeds @15 kg each as the other members, who had land were not interested to do an experiment and some of them were landless [5-6 members in the group].

Decision of growing Ashoka

When the information about seed distribution was disseminated first by GVT she had discussed this with her father and brother. Her brother was also aware and was also a member of another SHG, opted for some other livelihood development scheme. After the consensus at home to make a trial she brought the seed home.

Knowledge about growing Ashoka and process followed

It took time for the respondent to recapitulate the discussion held in 2003. It was more difficult for her as there was no discussion and follow up once the seed distribution was over. However, what she could remember was the GVT staff said it was for trial and after successful implementation they should grow it on their own and distribute seeds to their neighbours on demand. Her brother, who was also present during the seed distribution, said they were advised for broadcasting and told that no fertilisers and pesticides would be required. He could also remember that they were suggested to grow this variety in the upland.

However, they took a decision to grow this in the lower part of the upland and accordingly selected 0.20 acres land for 15 kg seeds. In response to the basis of selection of land they said that it was also suitable for other HYVs like ‘Pioneer’, ‘JK’ etc. and since they were informed that Ashoka was an upland variety, they thought it proper to grow in the same area where they grew HYVs before. It was confirmed that three members were involved in the trial and her father was not convinced in the broadcasting method as for all rice varieties they had adopted transplanting method.

Accordingly, they took a decision to prepare a seed bed for which approximately 5 kg DAP and cow dung were used. By the end of first week of July seed bed was prepared. In reply to the question why did they use DAP, while they were advised not

to use any fertiliser, they said it was a common practice for all the varieties and since there was no specific training on how to grow Ashoka they had applied their knowledge from experience. Seeds were soaked in the evening and in the next morning, after approximately 12 hours sown in the seed bed. Removal of seedlings took place approximately after 25-30 days and by then flowering started. 2-3 seedlings were planted together but did not follow any line. However, they could remove only 50 percent of the seedlings as they took a decision to grow only in 0.20 acres land. In response to the excess seedlings issue and whether there was any second thought to increase the area of land for Ashoka, they said they were experimenting and did not want to take risk in the first year. They required very little weeding after 3 weeks of transplantation. Harvesting was done in mid October and they got approximately 2 quintal (200 kg) paddy. Actually they generally keep the grains in jute bags (*bora*) in which approximately 70 kg paddy can be stored and from their memory they could say that Ashoka grains were stored in three such bags.

Observations, if any

- Plants were not as tall as the plants of other traditional upland rice varieties (*Goda dhan*).
- 2-3 seedlings planted together of which when one plant was matured others were not
- Harvesting got delayed and when harvested grains from the early matured varieties fell on the ground by then.
- Pinnacles were smaller than other traditional upland rice varieties, but number of grains in one pinnacle was more than traditional upland rice varieties.
- Animals preferred Ashoka fodders might be because of softness and taste.

They have compared Ashoka rice with traditional upland rice varieties and said the followings:

**Ashoka rice**	**Traditional upland rice varieties**
Tall and fine grains	Comparatively small and coarse
White and polished	Red
Tastier	Not so tasty
Can be consumed more	Difficult to consume more
Less time in cooking	Time consuming

They have compared the production of Ashoka rice, which was less than other HYVs like ‘Pioneer’ & ‘JK’ and informed that the area was very developed in terms of agricultural activities. Since they could grow HYVs in the lower part of upland Ashoka was not preferred there. Moreover they have started growing vegetables as cash crops in such land suitable for Ashoka, and it was more beneficial as they could generate regular cash income.

They did not grow Ashoka for the second time and said they have consumed the entire production. There was no demand for seeds in the village and since it was soft and tasty they did not sell Ashoka rice. Moreover, since it was not available in the market they were not sure about the price and marketability.

11. *Benjara*, GP Bero, Block Bero, District Ranchi, Jharkhand

Dr Dey

**Date**	29 March 2009
**Methods**	Observation, GD & IDI
**Participants**	Observation, FGD (1) and IDI (2)
**Evaluation team**	Dr Aniruddha Dey, LFC India and Mr Albert Xalxo, India – Field Support Officer, supported by Mr Vinay Tomar and Mr Sanjeev Ku Singh, representatives from GVT East.

Benjara was selected as a sample for secondary villages in Ranchi District, Jharkhand for qualitative study on Ashoka rice. In the quantitative study report this was shown as a secondary village of Nehalu (P2), where Ashoka rice was grown in 10.50 acre of the 15 acre total rice area in upland, meaning 70 percent upland was under Ashoka rice and the report also mentioned that all the households were growing Ashoka. However, in medium land only 2.4 acre land out of a total rice area of 12 acre was covered under Ashoka (20%).

One FGDs was conducted in the village and the villagers were very cordial. Both Ashoka growers and non-growers were present. It was a mix group of men (14) and women (3) Information collected on demographic profile, socio economic status of the people, livelihood issues and programmes, etc. by using different PRA tools and techniques like social mapping, Venn diagram, matrix analysis, seasonal mapping, cohort analysis, etc. Discussion continued and issues of Ashoka rice were discussed as most of the villagers received Ashoka seed in 2008, which was originally sent to Boda village under RIUP. PRA tools and techniques were used to understand the factors influenced them to grow Ashoka, process adopted, training and knowledge transfer, experience, problems faced, perception analysis, benefits of Ashoka in comparison to other varieties, impact on socio economic status, gender issues, dissemination and adoption, etc. From the FGD the team had identified two young farmers who had adopted Ashoka rice, for in-depth interviews.

It was a tribal village and they were Munda tribe. There were 20 families (two women headed households) with 140 total population. The village was surrounded by forests and there were three types of land – up (50%), medium (30%) and low land (20%). Most of the land situated in the eastern part of the village. They call the upland as

Tanr jamin, which is also referred as 4 *& 3 number jamin* (type 4 is the upper part of the up land, which is situated in the foot hills and non-cultivable, type 3 is lower part of the up land); medium land as *Chaonra jamin,* which is also referred as *2 number jamin* (type 2 as rainfed agriculture is possible there and they can grow more crops in such land in comparison to up land) and low land as *Gadda jamin,* which is also referred *as 1 number jamin* because of its water retention capacity, where they can grow rice, which is their staple food and at the same time by growing HYVs in such land they could generate surplus for sale in the market. In 3 *number jamin* they grow pulses, oil seeds and some traditional varieties of rice, namely *Charka Goda, Karia Goda and Dani Goda.* In the medium land they used to grow vegetables (mostly for their own consumption) and traditional rice varieties, namely *Rangi, Tilasair, Dudharice,Geruanthod, Karheni,* etc. during kharif seasons and since last 9-10 years they have started growing High Yielding Rice Varieties in the low land. They generally consume rice grown in their medium land and sale the low land varieties.

It was reported that only three villagers (Bhagirath Dhan, Rajendar Dhan and Relu Munda) got Ashoka seeds, which was known as *Birsa Dhan,* in 2003 from one GVT field worker and animator responsible for the neighbouring Boda village. Originally Ashoka seed was supplied to Boda village. Three farmers of Benjara were advised to try this new variety and then distribute seeds to other farmers. But they said there was no demand in the next year hence they did not give seeds to other villagers,

They were also told that it was a better crop for 3 *number jamin* as it was an early variety and the production would be more in comparison to other traditional rice varieties. Since transplantation was adopted they were suggested to go for seed bed preparation and removal of seedlings within 15 days. The farmers could remember that they were cautioned for not to allow the seedlings in the seedbed beyond 20 days, and they were told that "*bija budha ho jane se upaj kam hota hai*" (once the seedlings were older production would be affected). They were also suggested not to transplant more than three seedlings together and advocated to manure with cow dung.

However, in the first year all three of them grew in the medium land (type 3 land) as they did not want to take any risk because from their experience they knew that requirement of water was high in paddy cultivation and since their agriculture was totally rain fed they were vulnerable.

Only in 2006 Karma Dhan took 30 kg Ashoka rice from his neighbour Relu Munda in exchange of equal amount of seed of other rice variety. Karma Dhan and his son Dipu were present and Dipu said because of shortage of man power in the family they followed broadcasting method and finally production of paddy was approximately 2 qtl (200 kg). Ashoka rice was grown in medium land. They followed the same process and used same amount of seed in 2007 but production was approximately 3 qtl (300 kg). They said the difference was mainly due to good rain fall in 2007 and another perceived reason for low yield in 2006 was less efforts in weeding.

In 2007, another farmer, Lachhu Munda took 30 kg Ashoka seed from Karma Munda as he was fascinated after seeing the result. He also grew Ashoka rice in medium land and used transplanting method and yield was approximately 4 qtl (400 kg). According to Karma Munda's son Marwari Munda production was more because they followed transplanting method. However, in 2008 Lachhu Munda wanted to experiment in type 2 land and his experience was very bad. According to Marwari, who actually cultivated the land informed that seed bed was prepared but for transplanting they had to wait because of lack of sufficient water in the field required for transplanting. As time passed away seedlings became older and flowering started in the seed bed, which could not be transplanted and they had to grow *Karia Goda,* a traditional variety. As seedlings matured in the seed bed itself they could save only seeds.

Both Dipu and Marwari informed that they had applied 3kg DAP and 2 kg Urea in 0.15 acre land each.

Although only three villagers got the Ashoka seed in 2003 from GVT staff, grew them and no other farmer wanted to experiment with this variety till Karma Dhan volunteered to grow in 2006 and Lachhu Munda in 2007, but in 2008 when the same GVT staff offered them seeds of "*Birsa Dhan 109*" (empty packets were shown by the villagers)[5] 10 other farmers accepted it. This means in 2008 there were 15 Ashoka growers. According to them, seeds were supposed to be distributed in Boda village but one GVT staff (namely *Banshi)* brought the seed packets containing 2 kg each and distributed to 10 farmers. They could remember the process of cultivation

[5] On the packet "under RIUP" was printed. However, Benjara was not selected under RIUP and it could be revealed that since Boda was selected under RIUP, the GVT staff brought from that stock and distributed them in 2008 to Benjara.

as suggested by the GVT staff, who had also said that it was the same variety which was distributed to three farmers in 2003. They were suggested to follow the transplanting method and seedlings should be removed within 15-20 days.

The participants said Ashoka rice was not comparable with other traditional upland varieties as all the traditional rice varieties were grown in Type 2 and even some in Type 1 land, while Ashoka was suitable for Type 3 land. Example was cited from the experience learning of Lachhu Munda. However, they made a comparison with the traditional medium land varieties like, *Rangi, Tilasair, Dudhrice, Geruanthod and Kerhani,* which were as follows:

Table C: Comparaison between local land races and Ashoka rice

Traditional Medium Land Rice Varieties	***Ashoka Rice***
Yield more, eg. if 3. 5 qtls from 0.15 acre land	Ashoka's production would be 4 qtls from the same amount of land.
Time consuming and grows late	Early variety – and ensure food security during lean season, possible to grow a second crop
Coarse grains	Fine grains, long and white
Small pinnacles with lesser amount of grains	Long pinnacles with more number of grains
No taste	Very tasty and children liked most
Cannot be consumed in sufficient quantity at one go	Required amount can be consumed
Once eaten it can stay for long and farmers can work for longer period	Easily digestible and farmers cannot work for longer period
Once cooked can be taken for 2-3 days, which the villagers said they prefer	Cannot be kept overnight. Only hot rice preferred
Like rice straws are also coarse	When straws of Ashoka offered Animals preferred, which could be assessed by observation
No use of fertilizer only use cow dung.	Some amount of DAP and Urea required.
Not prone to diseases and infestation	Often attacked by stem borers locally known as *Gandibagh*

Broadcasting method can be applied and hence less expensive	Transplantation required, which is labour intensive, hence expensive and at the same time its difficult keep the timeline

Moreover according to the villagers Ashoka was more preferable because of its short span. They said in Sadri *“jitna jaldi paki utna hi achha hoyela, kale ki uprekar pani mein nirvar hai, jitna deri hui to utna sukha hoi jai aur dana ni hui – pila hoi jai”* (early varieties were preferred because their agriculture was totally dependent on Rain fall, and hence if delayed plants would get dry and there would be no grains – plants and grains would look pale.

All the new and old growers said they were happy with the yield and the potential. They said they would continue with Ashoka rice, but they would not switch over completely from the traditional varieties as they still give value to the food security issues of the traditional crops. It was informed that no seed was distributed outside the village.

Villagers said the GVT staffs never visited their place after 2003 and there was no monitoring and follow up. Farmers’ felt helpless when there were problems. Only in 2008 same people came and distributed new seeds and then they asked the original farmers about the production and related problems. According to them had there been minimum follow up the farmers’ would have been benefitted more.

12. ***Nehalu,* GP Nehalu Kapadia, Block Bero, District Ranchi, Jharkhand**

Dr Dey

**Date**	29 & 30 March 2009
**Methods**	Observation, FGD
**Participants**	17 villagers (5 women) – 6 Drop outs (5 in the second year and 1 in the fourth year), 1 Adopter & 10 non growers
**Qualitative Study team**	Dr Aniruddha Dey, LFC India and Mr Albert Xalxo, India – Field Support Officer, supported by Mr Vinay Tomar and Mr Sanjeev Ku Singh, representatives from GVT East.

Nehalu was selected as a sample in Ranchi District, Jharkhand for qualitative study on Ashoka rice under the category of primary villages where impact was high. In the quantitative study report this was shown as a primary village (P2), where Ashoka rice was grown in 9.28 acre of the 20 acre total rice area in upland, meaning 47 percent upland was under Ashoka rice and the report also mentioned that 46.15 percent households were growing Ashoka.

Nehalu village was visited by the team twice. As the project was over and there was no field staff from GVT the representatives accompanied the team felt it difficult to organise meetings there. After reaching over there in the afternoon on 29 March it was found that information was not disseminated and hardly there were any male member in the village. Then after some time a few villagers could be mobilised and a group discussion took place. Both men and women participated in the discussion of which there were six growers but drop outs and among the non growers three persons were aware about the Ashoka rice. Discussion continued and at the end another meeting was fixed on 30 March 2009 with their consent. List of Ashoka growers was consulted and a list of farmers was given to the willing participants, who took the responsibility to inform others and it was decided to conduct at least one more FGD and some IDIs. Accordingly the team arrived in the morning on 30 March but absolutely there was no improvement and the few who were present in the previous meeting said others were not willing and left for their routine works. Hence the team decided to move to another village, Hulsi.

Nehalu was a large village having 12 hamlets namely Ambatoli, Kaparia, Ghartoli, Barihatoli, Bartuli, Pokatikara, Dipatoli, JJtoli, Lathia, Nirmali, Tikratoli and Nehalu.

Even Ambatoli was too spread and it was heterogeneous. It seemed the residents of Ambatoli were slightly better off and there were many job holders. Ambatoli was there in the approach and well connected with Bero, the Block town.

Seeds of Ashoka rice were distributed in Ambatoli only in 2002 and not in the entire village (checked from the original distribution list also). The name Ashoka was not very familiar to them but they could easily relate with *Birsa Dhan.* In fact, one person who grew Ashoka rice in the first year and then dropped from the second year gave a small hint – that "*Ashoka Tanr jamin me lagane wala dhan"* (the rice variety to be grown in upland) after that all the participants took some time to remember then another participant started describing and people could remember. However, four women and one man could not remember, as they were not aware about the variety.

Out of the 16 participants only six persons grew Ashoka rice in the first year and then except Jagannath Sahu others stopped growing the variety since the second year. Jagannath Sahu also stopped growing from fourth year. He however, distributed seeds to two other villagers – one in Nehalu (3.5 kg) and the other one in Lathia (5.5 kg). There was a club in the village having 20-25 members and GVT staffs hold a meeting in the first year and distributed Ashoka seeds to the willing members 2 kg each. The participants could remember that only 15 members were given equal amount of seeds from a 30 kg packet and rest of the members did not show any interest. However, a few of them further exchanged their share with other farmers, hence the number of original recipients of Ashoka seeds became less and a few people grew more Ashoka seed in the first year. At the time of seed distribution the process of growing Ashoka and its benefits were also discussed, they could remember some points which were as follows:

- Both broadcasting and transplanting methods could be adopted.
- Broadcasting was suitable for upland
- For broadcasting they were advised to first plough then sow seeds in line and then for levelling.
- Transplanting should be done in medium land
- Very little water required
- Seedlings should be removed within 15-20 days
- After harvest the farmers should deposit at least 10 times of the seed received at the club.

Except one participant all others had deposited seeds from their yield to the club and in the second year all the deposited seeds were distributed from the club and there were new Ashoka growers in the second year, while some of the farmers grew Ashoka in the first year had left.

Some of the participants informed that false grains (*khakhri*) were more in the pinnacles which to their understanding was due to pest infestation and they were not in a position to afford to spray pesticides. According to them they did not have any complain against the seed but there might be other reasons like the absence of adequate attention and labour during cultivation, inadequate weeding and absence of any pest control measure as perceived by them for low yield.

Those who left had gone back to traditional upland rice varieties like *Dani goda, Naoa Khani Goda, Karenga Goda,* etc.

**Traditional Upland Rice Varieties**	**Ashoka Rice**
Production of *Dani Goda and Karenga Goda* almost equal – approximately 3 qtl in 0.20 acre.<br><br>While production of *Naoa Khani Goda* (very few people cultivate only as a ritual and to cook years first crop) – approximately 2 qtl in 0.20 acre.	Production was high – approximately 4 qtl in 0.20 acre.
Dani Goda - Both paddy and rice grains are red in colour and coarse.<br><br>*Karenga Goda* – Paddy black but rice grains red and coarse.<br><br>*Naoa Khani Goda* - Coarse but white.	Long, white and fine polished grains, soft and tasty, less time required for cooking
Except *Naoa Khani Goda,* which is also an early variety (it matures at	An early variety. Actually people only consume during the monsoon

least 15 days before any other traditional upland rice varieties) all other varieties are time consuming	seasons and they generally wait for their crops to grow. An early variety ensures more food security.

According to Mr Jagannath Sahu

- Production was a little less in the second year
- Production was further less in third year because of inadequate rain fall, grains could not mature and he could not even save the seeds.
- Though he wanted to continue but there was no seed available, which compelled him to switch over to traditional upland rice varieties again.

13. *Hulsi,* GP Hulsi, Block Bero, District Ranchi, Jharkhand

Dr Dey

**Date**	30 March 2009
**Methods**	Observation, FGD (2), IDI (4)
**Participants**	Ashoka growers, non growers and drop outs
**Qualitative Study team**	Dr Aniruddha Dey, LFC India and Mr Albert Xalxo, India – Field Support Officer, supported by Mr Vinay Tomar and Mr Sanjeev Ku Singh, representatives from GVT East.

Originally two primary villages and one secondary village were selected for the qualitative study and Hulsi was not in the initial selection. Since response was not satisfactory in Nehalu, which was selected as the high impact village under Primary village category based on the quantitative study report and whatever information collected did not match with the quantitative survey report, the team then selected Hulsi (the next high impact village) as another primary village in consultation with the GVT representatives. In the quantitative study report this was shown as a primary village (P3), where Ashoka rice was grown in 5.4 acre of the 12 acre total rice area in upland, meaning 42 percent upland was under Ashoka rice and the report also mentioned that 42.8 percent households were growing Ashoka.

Two FGDs were conducted in the village. In the first FGD both Ashoka growers and non-growers were present. It was a mixed group of men (10) and women (2) Information collected on demographic profile, socio economic status of the people, livelihood issues and programmes, etc. by using different PRA tools and techniques like social mapping, Venn diagram, matrix analysis, seasonal mapping, cohort analysis, etc. Another FGD was conducted with the Ashoka growers (8) present in the village, of which there were three participants who were continuing and five were drop outs. PRA tools and techniques were used to understand the factors influenced them to grow Ashoka, process adopted, training and knowledge transfer, experience, problems faced, perception analysis, benefits of Ashoka in comparison to other varieties, impact on socio economic status, gender issues, dissemination and adoption, etc. From the FGD the team had identified two growers, who were continuing and two drop outs for in-depth interviews.

It was a tribal village situated in a very interior place and isolated. The nearest village was Saheda Taber in the West, which was situated 3 km away. In the South the first village, Latratu was situated in 10 km away. There were 75 households (only 6 women headed households) and the inhabitants were the Oraon, a scheduled tribe and only one *Lohar* (black smith) family was there. From the document available with one villager it was known that the total land in the village was 1846.69 acre of which 54.25 acre was forest land. The village was surrounded by deep *Sal (Shorea robusta)* forests.

They have originally three types of agricultural land – up (60%), medium (30%) and low land (10%). They call the upland as *Tanr jamin,* which they have further divided into two and referred as 4 *number jamin* (50%) *& 3 number jamin (10%),* (type 4 is the upper part of the up land, which is situated in the foot hills and non-cultivable, type 3 is lower part of the upland, which is also known as *Tanr muha*); medium land as *Chaonra jamin,* which is also referred as *2 number jamin* (type 2 as rain fed agriculture is possible there and they can grow more crops in such land in comparison to up land) and low land as *Gadda Don,* which is also referred *as 1 number jamin* because of its water retention capacity, where they can grow rice, which is their staple food and at the same time by growing HYVs in such land they could generate surplus for sale in the market. In 4 *number jamin* they grow Millet, pulses, oil seeds, vegetables (for their own consumption) and wheat during monsoon. Only 15 families have 3 *number jamin,* where they grow traditional upland rice varieties, like *Bala Goda, Karheini, Ashoka,* etc. In the medium land (type 2 land) they used to grow traditional rice varieties, namely *Rangi, Bara Goda,* etc. during kharif seasons and since last 9-10 years they have started growing High Yielding Rice Varieties in both medium and low land. They generally consume rice grown in their medium land and sale the low land varieties.

Rice grown in different types of land

Type of land	Traditional	HYV
Low land/ *Gadda Don/ 1 number jamin*	Ghunghudata, Pani Sahil, Lal Khessa, Sorpia, Panthfor,	JK, Adwanta, 6444, IR64
Medium land/ *Chaonra*	Rangi and Bara Goda	IR36, IR64 and Adwanta

Jamin/ 2 number jamin		
Upland/ Tanr Muha / 3 *number jamin*	Ashoka, Bara Goda, Karhaini	None
Upland/ Tanr / 4 *number jamin*	No rice	No rice

It was derived that only 8-10 families were food secure for the entire year, 15-20 families were food secure for 8-10 months, 45-50 families were food secure for 6 months and only 5-6 families did not have food security even for two months. They have very little land and did not have agricultural implements and animals and unable to cultivate their land as it was difficult for them to invest. These families migrate to other states for 6-8 months. Ten years back almost all adult male and female members of the families used to migrate to the nearby towns and other big cities in search of wage employment in the fields of masonry work, factory labour, carpentry, brick klein, rickshaw pulling, etc.

However, lots of changes have taken place in the area and lots of job opportunities were also created in the locality. It was informed that before 1990 agriculture also was not improved and people did not have access to knowledge and information about the new technologies introduced for improved agricultural practices. The scenario has changed and with improved agricultural practices people have started investing in pump-sets and irrigation wells. They have shifted to HYVs from the traditional low yielding varieties. From the block level also farmers get information about new varieties and technologies and they get seeds and fertilisers at subsidised rates. Different organisations also started entering into the villages and working with the villagers for improving the quality of life. It was informed that even the villagers were facing problems of shortage of labour during peak agricultural seasons. During peak seasons they used to hire agriculture labourers for transplantation from the nearby villages like Saheda Taber, Muramu and Dola. Similarly villagers of Hulsi also used to get employment as agricultural labour in the nearby villages during peak seasons. However, it seemed the traditional customs of exchange labour system was an important reason for labour crisis. It was informed that if one villager required the services from his neighbour for ploughing his land then he would have to fed all the family members of his neighbour including rice beer. But priorities have changed

and people were looking for hard cash in exchange of labour, therefore most of the villagers would like to work outside the village.

There was discrimination in the wage rate in the entire region. While a man was paid INR 60 for his services as transplantation labour, for the same work a women was paid INR 30.

With the improvement in the livelihood areas people have started giving importance in children's education. There was a primary school in the village and all children were enrolled. After class V children go to Masu (5km) for studying upto class VIII. Five boys and one girl appeared in first School Board examination (class X) this year from Bero High School (10 km), next 3 boys and 2 girls would appear in School Board examination (class X) from Bero High School.

Although there was a Forest Protection Committee formed with the participatory forest management concept and people have access to non-timber forest process (NTFP) like *kend, piar, ranoo, imli, karanj, kathal, jamun* etc. for their own consumption as well as for sale, almost every family was engaged in selling fuel wood in the market. Ten families were engaged in making agricultural implements from forest wood.

Now almost 30 households have their own pump sets and there were 15 irrigation wells. There were 3 hand pumps and 5 wells in the village used as the main source of drinking water for the villagers. There was no electricity.

Ashoka rice was first distributed in the year 2002. Litu Oraon, one of the participants in the FGD said one GVT staff first gave a 30 kg packet of Ashoka seeds to Litu for distribution through the Mahila Mandal (women Self Help Group). He then distributed to 10 willing SHG members @ 3 kg per farmer. He had prepared the list of farmers to whom the seed was distributed and handed over the list to the concerned GVT staff. There was no instruction on the process, selection of land, etc. and never there was any follow up and counselling for the growers organised. It was interesting to note that some of the growers did not know the name of the variety but they could relate when they were referred that some years back seeds were distributed by Litu.

Farmers accepted it as it looked like one of their traditional varieties, *Bala Goda* and grew in type 2 land (*Tanr Muha*) from their experience. In the first year most of the farmers incurred loss as flowering started before removal of seedlings from the seed bed because of delay in transplanting. However, from the next year yield was good and till 2004 there was a demand. Somru Oraon, Mangra, Bande, Mohan and Rijervan Toppo of the same village adopted Ashoka in 2003 after seeing the benefits of this variety. They could remember that the seed spread to only three farmers, who were relatives of the Ashoka growers, outside the village to a maximum distance of 25 km. It was informed that only four farmers continued with Ashoka till 2008. Others left mainly because since last few years elephants started destroying their crops. The participants said they have observed that Ashoka fields were the first point of attack by the elephants, might be for several reasons like:

d) the plants were tall and it was easier to remove with their trunks
e) softness and taste of the fodders and grains
f) sweet smell from the matured plants

It was told that those who were continuing had land in the safer zone, otherwise all other farmers had to incur loss due to elephants.

Participants informed the following benefits of Ashoka:

- Early variety, it means securing their requirement for food during the crisis period. In fact, during monsoon the farmers mainly consume from their stock, and it depends on the size of land, investment capacity, knowledge, etc. of the farmer. In Hulsi village as the number of such elite farmers was less and most of the farmers were not food secure beyond six months period, the Ashoka growers could gain for at least two months depending on their family size, area of land under Ashoka and yield.
- Long pinnacles and more number of grains.
- Similarly it ensured food security of animals. They have observed that animals also have a preference for its fodder, might be because of its softness and taste.
- Tastier than other local varieties.

They have noticed one demerit of this variety that because of the height plants cannot stand in winds and the pinnacles touch the soil for which the grains get damaged. One of the participants said in Sadri that "*iss dhankar mahek haik aur*

mithas bhi ahhe, sekrele hathi badhia khaila, aur kisankar nuksan karela" (it has sweet smell, which attracts elephant and farmers incur loss).

Goyo Oraon (40), read upto class VIII in Masu School and took agriculture as his primary occupation and the primary earning member of his 10 members' family. Only 3.5 acre cultivable land (low land 2.5 acre and 1 acre medium land) was the main livelihood resource of the family. He has 5 acre more land, which he referred as *parti,* meaning fallow as that land was situated in the foot hills and no cultivation could be possible there. To cater to the basic minimum requirements of the family members he also does share cropping, his wife works as an agricultural labour and the eldest daughter has migrated to Kolkata to work in a brickyard. He was willing and a recipient of 3 kg Ashoka seed from Litu in 2002 and grew them in medium land adopting transplanting method. Finally they got approximately 1.2 qtl paddy (he said so far he remembered in the first year they stored in three plastic sacks and each sack had a capacity to store 40 kg paddy), which they consumed in 35 to 40 days period. He had compared this with previous years and said earlier they used to buy because during that time they did not have any stock of rice. Their expectation was more as production of *Bala Goda* was more. However they realized that low yield was not related to grain quality rather it was more because of late removal and delayed rain fall. Next year also they grew Ashoka and used 15 kg seed, removal of seedlings were in time and applied cow dung, DAP and urea and they did not have to buy rice for more than 45 days (after that harvest of other varieties start). However, since 2005 he had left growing Ashoka because in 2004 the entire crop was spoilt by elephant and even seeds could not be saved.

Samru Oraon (30) can only sign and agriculture as the primary occupation. They have 8 acre family land of which 5.5 acre was cultivable (1.17 acre low land, 1 acre medium land, 3.3 acre *Tanr Muha*). He had adopted this new variety in the second year. He said he was interested to grow Ashoka in the first year itself but as there was no seed left he had to give up. Next year he took 30 kg seed from Litu Oraon and grown Ashoka rice in 0.5 acre *Tanr muha* land. He had followed transplanting method but because of labour problem removal of seedlings was delayed as there was no agricultural labour available and he with her wife could remove only 1/3 seedlings and transplanted. Finally they got approximately 2 qtl (200 kg) from the cultivated land and approximately 30 kg from the left over seedlings. He said Litu also did not inform him anything about the process of growing Ashoka.

Samru continued with Ashoka rice for two years and in the second year (2004) he also faced the similar problem like Goyo Oraon. His crops were badly damaged by the elephants and there was no seed left. In response to the issue of the fate of other crops he said *Deshi Goda* (traditional upland rice) varieties were dwarf and there might be some problems for the elephants to grip those smaller plants. Moreover, the pinnacles were also smaller and they have experienced that those varieties were less attractive to the elephants and damage was comparatively much less. However, the traditional varieties also got damaged because of the movements of the elephants on the plot. He said the situation has improved since last two years as the number of elephants decreased.

About its benefit he said in Sadri "*Je bera kharcha pani sirai jaila sehi bera Ashoka dhan paik jaila*" (Ashoka matures at a time when the farmers' stock of rice get exhausted) and hence it was really beneficial as they could save their hard earned cash.

Since 2005 he had shifted to another traditional rice variety – *Geruanthod* and compared Ashoka with it, which were as follows:

Geruanthod	***Ashoka***
Coarse grains	Fine grains
Small grains	Long grains
Smoke gray colour	white
Less consumed but do not feel hungry for long	Can be consumed more but do not give equal strength
Less tasty	Very tasty
Hard fodder	Soft fodder
More time required for cooking	Less time required for cooking

Gahnu Oraon (56) also grew Ashoka in 2002 but he was the only person from his experience realised after 15 days that seedlings were well grown and without wasting time he had transplanted the seedlings and from his 0.4 acre *Tanr muha* land he got 4 qtl (400kg) yield. During transplantation he had applied 5 kg DAP and also did weeding well. He did not spray any pesticides nor used urea for Ashoka rice.

Next year he had given 3 kg Ashoka seed to one of his neighbours and rest of the Ashoka rice was consumed in the family. In 2003 he again changed the variety as he felt changing variety would produce more yield. He grew another traditional upland variety – *Bachha Kalma* in the same plots and by using 40 kg seeds he got 4 qtl yield. When asked which one was better, he replied Ashoka was better than any other traditional upland rice variety as he had experienced 3 months food sufficiency because it was an early variety and saved his hard earned cash. However, he did not want to continue growing Ashoka as HYVs were available by that time and he had shifted to IR 64, which could be sold in the market.

Litu Oraon (30) was very dynamic and social. He had contacts with outside agencies and had a tendency to generate resources external resources. For his social activities he was highly respected in the village. In 2002 Ashoka seed was first introduced in the village through him. He had distributed to 9 other villagers and experimented in his own land. He was continuing with Ashoka rice but he felt the villagers should have been given proper orientation on the process of growing Ashoka and in absence of it many farmers incurred loss in the first year. He also felt that some follow up from GVT would have helped the farmers to gain confidence and its spread in the village. Regarding the potential they were convinced but even after growing it for so many years he felt there was scope for improvement with guidance. He said even after so many years he was not very comfortable in saying how much seed required for 1 acre of land. According to him had there been some hand outs or pamphlets mentioning the selection of land, amount of seed required for a specific unit, methods to be followed, requirements of fertilisers and pesticides, sowing and harvest time, etc. the situation would have been much better and more number of people would have been benefitted from the variety. After the distribution of seeds in 2002 only in 2008 for the first time some people had visited the village and enquired about the Ashoka variety and Litu said after that visit he had prepared a list of Ashoka growers as a preparatory for further investigations.

14. ***Muramu,* GP Jaria, Block Bero, District Ranchi, Jharkhand**

Dr Dey

**Date**	31 March 2009
**Methods**	Observation, FGD (2), IDI (2)
**Participants**	Ashoka growers, non growers and drop outs
**Qualitative Study team**	Dr Aniruddha Dey, LFC India and Mr Albert Xalxo, India – Field Support Officer, supported by Mr Vinay Tomar and Mr Sanjeev Ku Singh, representatives from GVT East.

Muramu was selected as a sample in Ranchi District, Jharkhand for qualitative study on Ashoka rice under the category of primary villages where impact was low. In the quantitative study report this was shown as a primary village (P1), where Ashoka rice was grown in 3.37 acre of the 34.64 acre total rice area in upland, meaning only 10.02 percent upland was under Ashoka rice and the report also mentioned that 11.25 percent households were growing Ashoka.

Two FGDs were conducted in the village. In the first FGD only men (22) were present of which there were 18 tribals and 4 non-tribals were present. Information collected on demographic profile, socio economic status of the people, livelihood issues and programmes, etc. by using different PRA tools and techniques like social mapping, Venn diagram, matrix analysis, seasonal mapping, cohort analysis, etc. from the FGD two growers but drop outs were identified for in-depth interviews. Second FGD was with the tribal women (12) of both grower and non-grower families, where primarily gender issues, awareness on Ashoka rice, decision making, migration, education of the children etc. were emphasised.

It was a heterogeneous village having 130 households of Oraon (Scheduled tribes-60%), Mahato (30%), Rajput (5 HHs), Weaver (6HHs) and Lohar (1 HH). Though it was a heterogeneous village but they were united and there were a few very active members and under their leadership village development works were in progress. During the study we saw the young adults remained busy in digging soil. The villagers said it was to erect the electric poles. They also said electric poles were there since last 20 years without cables. A sum of INR 1500 was collected from villagers' contribution and deposited to the State Electricity Board and they the villagers were assured of getting electricity very soon. To expedite the matter they

took up the digging work for erecting the poles in their hands for which at least INR 12000 would be back to the same village as wages of the village youth worked for 60 poles, for which rate was fixed @ INR 200. This small piece of information helped the team members to understand the quality of leadership, spirit and involvement of the villagers to join hands for common causes, togetherness, etc.

It was informed that only two men from the Oraon community were in service. From almost all Oraon HHs at least 1-2 members and almost from 10-12 Mahato families work in brickyards in and around Kolkata. They take their kids also with them those are not in school. In the families where elderly members present they become the custodians of the school going children. Generally they migrate in November-December and come back in June-July and again work as agricultural labour in the region. It was reported that since last 2-3 years such migration has been reduced in 20 percent cases. Rest of the members of the Mahato families work in various trades like wage labour, porter, rickshaw puller etc. in Ranchi. Rajputs do not migrate but the Weavers neither have sufficient land nor there is any demand for their skills, hence they also migrate to other states. The Lohar family (black smith) makes agricultural implements for others.

Food sufficiency in %

- Whole year food sufficiency = 5%
- Eight months food sufficiency = 20%
- 6 months food sufficiency = 50%
- 3 months food sufficiency = 25%
- Only one family was completely landless.

Land

- Agriculture land = 60%
- Forest area = 35%
- Village cover = 5%

There are 4 types of agricultural land exists in the village

1 no. land – Low land / Don / Gaddhsa = 20%

2 no. land – Medium land / Chawra = 10%

3 no. land – Up land / Tanr Muha = 10%

4 no. land – Up land / Tanr = 20%

Crops grown in 1 no. land are as follows:-

HYVs - Pioneer, JK, IR36, 6444 and IR64. 10 years back they used to grow *Dehati dhan* (Traditional local varities for low land) like, *Karia, Dusri, Dudhkalam, Kalamdani, Tilasari, Bachhakalam, Aginsal, etc.* and depending upon the volume of land maximum food sufficiency was for 7 to 8 months, but now with HYVs they can even sell their produce. In fact after the introduction of HYVs the local small land holders also got the opportunity to do business.

Crops grown in 2 no. land are as follows:-

HYVs - *Pioneer 2, IR64, Adwanta, Tulsi Goda, Karheni,* etc. 10 years back they used to grow traditional varieties like *Dudha rice, Tila Sair, Karhaini etc.* and depending upon the volume of land maximum food sufficiency was for 5 to 6 months and now left all these and with HYVs they can even sell their produce.

Crops grown in 3 no. land are as follows:-

Now they grow *Tulsi Goda, Birsa Dhan / Ashoka rice, Karhani.* It remained fallow to 10 year's beck.

Crops grown in 4 no. land (*Tanr jamin)* are as follows:-

Potato, Pea, Mustard, Wheat, Onion, Bins etc. with the help of irrigation well and almost 70 percent people have their own irrigation well. Now they have started growing two crops in *Tanr Jamin*. Some 10 years back even during monsoon seasons these land remained fallow as they were not aware about vegetable cultivation.

Oroans have maximum land in the village.

Education

There was a primary school in the village where children can study upto class V, which was established in 2006 and till then the nearest primary schools were at Masu (1 km) and Jaria (2km). After class V still the children are going to Masu School for their studies upto class VIII and after that to pursue further they have to go to Bero (4 km). This year only six girls' were in class X and 5 boys' were studying in Bero College.

10 to 15 years beck girls' used to get marry at the age of 10 to 12 years and boys' at 15 years of age. Now girls' are getting married at the age of 18 to 22 years and boys' at the age of 21 to 25 years.

Prevailing diseases in the village

Malaria, fever, cold and cough are the common ailments. Almost all of them go to the PHC at Bero for treatment with modern medicine and 20 years beck the scenario was completely different when they used call ethno medicine men or faith healers.

Source of water in the village

Since 1990 the villagers started using hand pumps for potable water and there were three hand pumps at different locations. However, because of insufficient number of hand pumps many people still use water from the wells for drinking and for other household activities.

Social groups

Three Mahila Mandals and one Forest Protection Committee (FPC) exist in the village and FPC consists of both men and women. The first Mahila Mandal was formed 8-9 years back with only Oraon women. Then the number of members increased and the second group was formed after 5-6 years and the third group was formed very recently. There are two other clubs for men, namely Junior Club, Muramu and Football Club, Muramu.

Agriculture and Division of labour

**Type of work**	**Men / Women**
Ploughing	Men
Seedbed preparation	Men
Sowing seeds	Men
Removal of seedlings	Women
Transplanting	Women
Weeding	Women
Fertile and pesticide spray	Men
Monitoring	Men
Harvest	Women
Carrying (*dhoai/bahai*)	Men & Women
Daori [Thrashing with bullocks]	Men
Winnowing	Women
Accumulation	Men & Women
Boiling	Women
Milling	Men & Women

Husking	Women

Most of the village women remain busy in gainful employment during July-August as its the time for transplantation and women are preferred for this job. For this work in other villages as agricultural labour they earn @ INR 40/- per day and for the same job in the village they earn @INR 30/- per day. Very few men do this but they get just double the amont in both within the village and outside. Similar scope for gainful employment comes during harvest season in November-December. With the introduction of HYVs scope of work for the women in both the seasons has increased and at least 4 months works are ensured.

Birsa Dhan / Ashoka rice was first introduced in 2002. Seeds were distributed first to ten farmers through the Junior Club, Muramu. One packet (30 kg) was handed over to the group by the GVT field staff and the members were requested to distribute them amongst 10 willing members equally. Accordingly 10 farmers received seeds 3 kg each and they grew it in 3 no. land. However, GVT's role was only as a carrier as apart from bringing the seed packet they did not play any further role like, there was no interaction with the farmers on why they were introducing the new seed, benefits of the new variety, selection of land, process of cultivation, etc. In the first year all growers incurred loss because of delay in the removal of seedlings and flowering started in the seedbed and there was a negative impact among others. Six farmers dropped in the second year. Of the four growers, who wanted to grow, only Charoan Oraon continued till 2007, two farmers grew Ashoka for 3 years and one for 2 years. It was informed that only because of lack of knowledge they lost their crop in the first year but the variety was good because those who grew it they did not incur any further loss due to lack of knowledge but they became victims of elephants. Others could have experimented but they said since it was a rain fed crop and seedlings matured very early they could not take further risk as transplanting might get further delayed because of inadequate rain fall.

Jena Oraon, though continued for 3 consecutive years said that had there been minimum information on time of removal of seedlings they would have got very good yield as in the first year there was adequate rain fall. From his experience he said if transplanting got delayed beyond 25 days then there would be no production. From his experience he has seen that because the plants are tall and pinnacles long with

more grains they are susceptible to wind and fall on the ground, which affects the yield and grains become black.

Filman Tigga was another grower who was interested to continue but left after second year, because first year transplanting got delayed and flowering started in the seed bed and in the second year again got delayed not because of knowledge but there was insufficient water in the field and seedlings damaged for the second time. From third year Ashoka was replaced with IR64, for which they have adequate knowledge and production was good.

Charoan Oraon said he was continuing till 2007 but since 2005 production was not upto the expectation though from experience they could solve and address all the problems, but they have experienced that pest infestations were more. In 2007 he had sprayed pesticides of about INR 700 but could not save much. According to him sweet smell of Ashoka rice might be one of the reasons of pest infestation.

Sadho Oraon referred Ashoka rice as *"Hathi Khane wala dhan"* – the rice variety most preferred and consumed by elephants. He also said *"hathi iska mithas ke karan isiko pasand karta hai, isika poal bhi naram hota hai"* – it has a sweet taste hence elephants like it more, moreover, its fodders are also soft. These are all perceptions of the victims who have lost very heavily as elephants destroyed their crops. They have observed that elephants do not like HYVs to eat but HYV crops are also destroyed mainly because of elephant's movements in those plots. People perceive that use of fertilisers and pesticides might be the reason for aversion.

It was interesting to note that the women were not well conversant on the agricultural issues, though they are the main work force. They said they simply follow the instructions and they don't have any role in decision making either in the agricultural practices or in other family related issues. For family related issues they are simply consulted and it depends on whether the adult males like their suggestions or not. Regarding payment as agricultural labour the women get only INR 30 per day while for the same work one man gets INR 60 per day.
