# Supplementary material for: Plant breeding can be made more efficient by having fewer, better crosses
Source: BMC Plant Biol. 2013 Feb 7;13:22. doi: 10.1186/1471-2229-13-22 (PMC3621587; doi:10.1186/1471-2229-13-22)
Supplement: Additional file 3 — Impact of New Upland Rice Varieties in Eastern India from Client-Oriented Breeding: Evidence from Whole Village Surveys. Bangor, UK: Centre for Arid Zone Studies (CAZS), University of Wales; 2004. [file 1471-2229-13-22-S3.pdf]

New Rice Varieties for Nepal
Rainfed Agriculture Impact Assessment Study No. 2

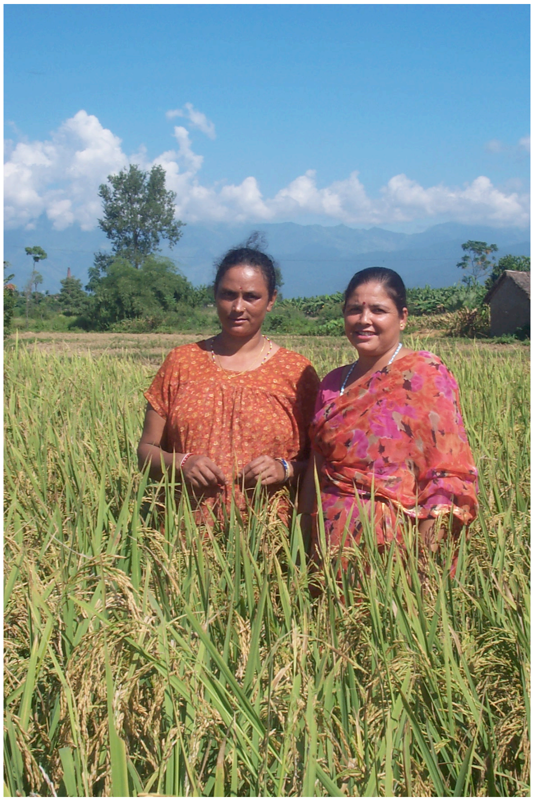

Monitoring Impact Assessment and Learning Component (MIL) of the Research into Use Programme (RiU)

New Rice Varieties for Nepal: Outcomes of Client-Oriented Breeding or Identified by Participatory Varietal Selection

Prepared by:

Part A: Czech Conroy

Part B: John R. Witcombe, Bikash Paudel, Rachana Devkota and Dhruba Neupane

Acknowledgements

In addition to the authors of the different parts of this case study, several staff members of LI-BIRD were involved in conducting the fieldwork for both the structured survey and the qualitative survey: we are grateful to them all. We would also like to thank LI-BIRD management for their support throughout the study. We are also grateful to Dr Sheelagh O'Reilly of IOD for her continual support, advice and encouragement throughout the study; and to Dr Krisha Joshi for his liaison role and logistical support in Nepal. Finally, we acknowledge the contribution of the many farmers who made this study possible and gave so freely of their time.

TABLE OF CONTENTS

Page no

Acknowledgements ... i
PART A SYNTHESIS OF STUDY FINDINGS ... 1
INTRODUCTION ... 1
GENERAL OVERVIEW OF THE METHODOLOGY ... 2
CASE STUDY FINDINGS ... 4
LEARNINGS and INSIGHTS ... 8
PART B REPORT ON THE STRUCTURED SURVEY ... 1
EXECUTIVE SUMMARY ... 2
INTRODUCTION ... 4
METHODS ... 12
Overview of methods used ... 12
Identification of four high-to-medium intervention districts for sampling ... 13
Identification of two low-intervention districts for sampling ... 13
Selection of villages within the districts ... 14
The survey methods within each village: Group discussions ... 14
The survey methods within each village: Household survey ... 15
Household survey using transects defined by the Global Positioning System ... 17
Poverty index ... 21
RESULTS AND DISCUSSION ... 22
Extent of Use of the COB and PVS Varieties in the Household survey Area ... 22
Seed spread ... 30
Estimated extent of Use of the COB and PVS Varieties Outside of the Household surveyed Villages ... 35
Environmental impact ... 40
Benefits to users ... 41
Characteristics of users and non users ... 45
REFERENCES ... 49
Appendix 1. Districts in the Nepal terai with year of project entry and approximate seed quantities distributed by 2007 ... 51

PART A SYNTHESIS OF STUDY FINDINGS

Prepared by:

Czech Conroy
Natural Resources Institute
University of Greenwich, ME4 4TB
UK

INTRODUCTION

This case study is an impact assessment of new rice varieties, primarily ones that were developed through client-oriented breeding (COB) in DFID Renewable Natural Resource Strategy (RNRRS) supported projects of the Plant Sciences Research Programme (PSP). The participatory research in rice in the RNRRS projects in Nepal was termed participatory crop improvement (PCI) and had two major components i.e., participatory varietal selection (PVS) and client-oriented breeding. (These terms are defined in Part B.)

The first PSP project, on PCI in high potential production systems, ran from 1997-2000 and was followed by a second one (2000-2003). Although the RNRRS projects were initially designed to test PCI in high-potential production systems, much of the project area was less favourable for agriculture. Rice was grown under rainfed conditions or with only limited quantities of irrigation water. The projects focused on Nepal's terai, the country's 20 to 30 km wide low-lying belt of largely flat and fertile land stretching from the east to the west. Rice was targeted as it is the most important crop in the terai. The National Rice Research Program has released relatively few varieties for the Terai, particularly considering its importance in area; and only a minority of the released varieties have been widely used by farmers.

PVS and COB were *methodological innovations*, both of which have been applied to other crops as well as rice, and these were the subject of a separate impact assessment study in this series. This study focuses on *technological innovations* - mainly on eight COB varieties, namely: Barkhe 1027, Barkhe 2014, Barkhe 3004, Judi 572, Barkhe 2001, Barkhe 2024, Sugandha 1 and Sunaulo Sugandha. It also considers one of four varieties identified by participatory varietal selection (PVS), namely BG 1442.

PVS and COB activities started in 1997 in the central districts of Chitwan and Nawalparasi and by 2002 some seed of PVS and/or COB varieties had been supplied to villages in 10 districts of the terai. Seed was supplied both directly by NGO partners and also indirectly via District Agricultural Development Offices (DADOs) – who did not always keep records of the villages to which the seed had been distributed.

BG 1442 was identified by farmers as a useful variety in PVS trials in the RNRRS projects and was subsequently widely promoted within these projects: large scale seed supply of BG 1442 by the PSP project began in 2001. BG 1442 was released by NARC as *Hardinath 1* in 2004. The first COB varieties were tested in PVS trials in 2001 and 2002; and scaling up of the first promising varieties began on a small scale in 2002. In 2006 the first COB variety (Barkhe 3004) from a RNRRS project was released and the second (Sunaulo Sugandha) in 2008.

The PSP projects were managed by Bangor University's CAZS-Natural Resources (CAZS-NR). The principal local partners were NGOs, namely Local Initiatives for Biodiversity in Research and Development (LI-BIRD), Nepal, and; and the Forum for Rural Welfare and Agricultural Reform for Development (FORWARD). The former was involved in both rice COB and PVS and the latter only PVS.

This study used a variety of structured and semi-structured methods. Part B of this report describes the methodology and findings of fieldwork undertaken by CAZS-NR, in collaboration with the Nepali NGO, LI-BIRD. Part C describes a subsequent semi-structured and more qualitative piece of fieldwork undertaken by Dr Marlene Buchy and LI-BIRD staff. This part of the document:

- describes the overall methodology used in this case study;
- synthesizes the key findings from the two studies, identifying both similarities and differences; and also
- draws lessons from the findings relating to technology development and innovation processes.

This is one of seven case studies on rainfed agriculture innovations in south Asia, which are part of a broader 'cluster study'. Each case study, and the broader cluster study, aims to obtain information regarding:

1. *extent* of use of the innovation
2. factors *explaining extent* of innovation (factors influencing use)
3. *Sustainability* of use of innovations by farmers.
4. *who the innovators are* and are not
5. *impact* (including benefits and disbenefits) of the innovation
6. factors *explaining differential impact* among potential users/innovators.

GENERAL OVERVIEW OF THE METHODOLOGY

Six survey districts were purposively selected to represent the range of agro-ecological conditions across the terai – 2 in the west, 2 in the east and 2 central ones; and to cover different levels of project intervention. Six villages were surveyed in each district. (See Part B for details.)

The *structured survey* involved two methods (see Part B for further details). First, village-level group discussions to determine the varietal composition of rice among rice growers, and the extent to which the COB/PVS varieties were being grown. Second, a questionnaire-based household survey of rice growers in the survey villages, comprising 10 current COB/PVS users and 4 non-users per village.

The *qualitative (semi-structured) study* was conducted in three of the six districts covered by the structured survey – one in the east, one in the west and one central. Three villages were selected in each district, of which one had been covered by the structured survey (see Part C for details). The methods used in this study included fairly open-ended group discussions that had a number of core topics, such as: changes in livelihoods and sustainability of livelihoods.

What kind of comparison?

Assessing the impact of any particular development intervention – be it a technology, policy or institutional change – is seldom straightforward. The context in which the intervention takes place is real life – dynamic, complex, uncontrolled and often spatially heterogeneous – and this makes it difficult to separate the effects (if any) of the intervention from any other changes in people's lives that may have been taking place. There may be other contextual changes in rural study areas - such as electrification, development and roads and schools -that could have contributed to any general positive changes in local livelihoods that have taken place since the intervention was implemented or initiated.

In controlled experiments scientific researchers often make *'with and without'* comparisons involving two or more very similar groups of people, livestock, crops or whatever is being studied and is expected to be affected by the 'intervention' or 'treatment'. Any differences that develop between the groups during the period of the experiment can then be plausibly attributed to the 'intervention'. However, use of a *'with and without'* approach was not possible in this study for two reasons. First, it would have been difficult to ensure that any two villages involved in a comparison were sufficiently similar, and hence be confident that any differences subsequently identified between rice-growing farmers in the two villages were due to the COB/PVS varieties. Second, records of the villages to which rice seed had been distributed were incomplete, particularly in the case of distribution by DADOs, and hence there was no comprehensive list of 'project' villages with seed.

The 'before and after' approach was used. This approach has its challenges – for example, if we had been measuring impact in terms of changes in household income since COB/PVS rice varieties had been 'adopted' by farmers then any increase in income could have been due to many other factors. Thus, the study team used a kind of 'results chain' approach to minimise this kind of problem. The before and after comparisons were based on farmer recall and focused on changes in variables like rice yield and self-sufficiency in rice (in months per year). The team examined the linkages between area of land planted to COB/PVS rice varieties, the yield obtained, the ways in which the rice was used and any benefits that farmers said they had experienced as a result of growing these varieties. However, this was complicated by the fact that the study covered nine rice varieties, each with its own traits and benefits.

Minimising bias

The senior staff of RIUP's MIL component decided that the structured survey would be implemented by staff of the organisations that had been involved in developing and distributing the COB/PVS varieties varieties, as they were familiar with the technology and with the locations where the seed had been distributed and the villagers living there. An independent Cluster Study Team Manager (Czech Conroy) was appointed to oversee the design and implementation of this case study and several others. He had the final say on survey methods, wording of questionnaires etc. In the structured survey household selection was random, but for practical reasons the selection was done by the field teams implementing the survey.

In the questionnaire used in the structured household survey almost all of the questions were closed (e.g. Yes/No) rather than open in order to avoid bias (sub-conscious as well as conscious) in the way that answers were recorded. The qualitative study, on the other hand, would be inherently semi-structured or unstructured, which would have increased

the potential for bias (conscious or sub-conscious) among survey team members creeping in. For this reason, and also because of the special skills required to undertake qualitative work of this nature, the original plan was that this study would be done by a suitable qualified independent researcher. However, the person concerned withdrew his services as the work was about to commence. It was then decided that the study would be done by suitably qualified LI-BIRD staff, under the supervision of the Study Team's social development advisor, Dr Marlene Buchy.

The use of two different sets of study methods was also seen as advantageous in terms of triangulation – cross-checking – of survey findings.

Assessing the wealth status of respondents

The Cluster Study Team developed a poverty index (PI) to be used in four technology impact assessment studies, including this one. The PI enables the study to distinguish wealthier households from the rest of the households among those surveyed, and to compare the wealth status of technology users with that of non-users. There were six indicators selected for the poverty index, each of which was given a set of possible scores (see Part B, Table 6 for details), namely:

- Livestock units
- Total quantity of all food grains produced in the season 07-08 per capita
- Roof type
- Number of jobholders in household who provide income
- Ownership of a tractor
- Extent of unskilled labour migration.

CASE STUDY FINDINGS

This section synthesizes the findings from the structured survey (found in Part B) and the semi-structured, qualitative survey (Part C). By and large the two sets of findings are consistent and complementary: where discrepancies occur they have been noted.

1. Extent of Use

Within a household's rice-growing area The area on which the varieties were grown was small and averaged about 0.17 ha per household per variety. This could not be explained by land availability as there was no correlation at all between the area a household devoted to COB varieties and the area of rice that a household cultivated.

Apart from two exceptions, individual COB varieties were grown on an average of at least 0.1 ha – or 12.5% of the land used for rice production. Barkhe 3004 had the highest average area of any variety (more than one third of a hectare) and accounted for 27% of the total rice area of the households that grew this variety. The overall proportion of land devoted to COB varieties among the users was somewhat higher, at 15%, as 17% of COB user households grew two or more COB varieties.

Over all six districts, eight COB varieties were found to be grown by at least 1% of all 2,222 households identified in the group discussions. Three other COB varieties were used by less than 1% of households and were excluded from the analysis to reduce its complexity. Sunaulo Sugandha, one of the two released COB varieties was the most widely grown (by 7.5% of all 2,222 households) among all of the varieties. Barkhe 3004 (2.4%), the other released variety, did not have the higher use that might be expected

from its official release and greater promotion (more seed of it had been supplied than of other varieties). It was about as widely grown as three unreleased varieties i.e., Barkhe 1027 (2.8%), Judi 572 (2.4%) and Barkhe 2014 (2.0%).

Within and between survey villages By 2008, a range of COB varieties had been adopted for upland and lowland rice ecosystems by an average of 17% of all the households in the 36 study villages.

PVS varieties were more widely used than COB varieties i.e., 36% of rice growing households grew a PVS variety compared with 10% for COB varieties. This is probably related to the longer time in which PVS varieties were available as the research project started with PVS and first seed distribution took place in 1998 whereas the first COB varieties were not distributed until 2001 and then in only small quantities.

Within and beyond survey districts High rates of spread of seed and information were found, and current use of COB varieties in the six study districts was 15 times higher than the amount of seed that had been supplied since 2002. If current use (estimated from the group discussions and household survey and from the opinions of DADO staff) were to be extrapolated to all the terai districts, then a range of 1.5 to 2.4% of the rice area (15,000 – 25,000 ha) would have been devoted to COB varieties and grown by more than 150,000 households by 2008. By 2010 to 2011, assuming that rates of spread found in the study continue, then up to 100,000 ha could be occupied by COB varieties.

BG 1442 (Hardinath 1) was found to be used more extensively than any of the individual COB varieties. Hence, the total area under COB and PVS varieties combined would be expected to exceed 100,000 ha and 150,000 households by 2010 or 2011.

The **scoping study** identified more COB varieties than the household survey as it was done for all of the terai districts and not just six. It identified Barkhe 3004 as the most widely grown COB variety. The scoping study identified a similar number of COB varieties to the household survey in the six survey districts, although there was very poor agreement across the two methods. Since the use of individual COB varieties varied greatly across villages, and only six villages were sampled per district, good agreement is unlikely between the two methods; in the scoping study, expert opinion was based on the entire district. However, the DADO officers seemed not to have kept up with the rapidly changing situation: they were not always aware that the use of new, non-released COB varieties, such as Barkhe 1027, had increased quickly.

2. Factors explaining extent of innovation

Within a household's rice-growing area There are different possible explanations for the small proportion of rice land on which COB varieties were grown. *One* is that the low areas per farmer may partly be because this is an early stage in the innovation process so the use of the COB varieties could be limited by seed availability as well as a desire by some farmers to try the variety for more years before taking the risk of growing it on a larger area. However, no meaningful test of this explanation was possible as many farmers were not able to say when they first got access to seed; and because the sample size for earlier years was very low as at that time only small quantities of seed were distributed.

A *second* explanation is that all of these varieties (with the possible exception of Barkhe 3004) are niche varieties that will be grown by many farmers but on relatively small

proportions of total rice land. However, the breeding programme was not targeted at producing niche varieties and the wide use of some of the varieties across districts would make this seem unlikely. A *third* possible explanation is that some COB varieties have negative traits as well as positive ones, and therefore farmers may prefer to grow a mixture of varieties. In the qualitative survey farmers were asked to compare some of the COB and PVS varieties with other commonly used ones, and this comparison revealed a number of perceived limitations in some of these varieties (see Table 5, Part C).

Within and between survey villages In 12 of the 36 villages COB varieties were not grown at all, while in some others over 70% of the households grew them. The most likely reason why none were grown in some villages is lack of access to seed, given the very small quantities of seed that were distributed at a district level compared with the rice area. If this access were to improve then the use of COB varieties could increase in villages where use is currently low.

Users of particular varieties The agro-ecological niche of a particular variety could be one of the factors influencing the nature and number of farmers using it. Some varieties may require relatively good growing conditions, while others may be well adapted to the less favourable conditions more likely to characterise the landholdings of poorer farmers. An analysis was made of the difference between users and non-users of the Sunaulo Sugandha variety as this was the only variety with more than 30 users among the 344 users of any PVS or COB variety covered by the household survey. There were 53 users of this variety in the three districts where it had the highest use. On average the farmers that adopted Sunaulo Sugandha had more medium land and significantly more grain production. The authors of Part B concluded that differences between the users and non-users are a reflection of *the adaptation of Sunaulo Sugandha for fertile medium and lowland conditions*. The qualitative study found that poorer farmers in one village were disinclined to grow Sunaulo Sugandha because it has a lower yield, and they prioritised quantity rather than quality and market price (Table 5, Part B).

It is possible that some of the other COB varieties may also be used by households that differ from the non-user households for wealth indicators - for example, Barkhe 1027 might be expected to be used by poorer households with more upland. However, the sample size of 19 users was too small for a meaningful analysis, as in any one district the sample size of users was very small.

3. Sustainability - continuity of use

The study findings do not provide a clear answer to this question. This is partly because many of the farmers surveyed only started using COB varieties relatively recently, and partly because of the complexity involved in obtaining this kind of information for nine different rice varieties.

4. Who are the innovators?

Poverty The maximum value for the poverty index in Nepal was 23, and any user household with a score of 12 or less was considered to be poor. No households had a score above 15. There was a small but significant ($p < 0.05$) difference in the mean total poverty scores of users and non-users – see Table A1.

Table A1 Poverty Status of Users and Non-Users (mean PI)

Study	Users	Non-Users
Improved rice	6.96	6.37

This overall difference stemmed from significant differences for two of the PI indicators, namely food grain production per capita and livestock units, with users having higher average quantities of each.

Inter-district differences An analysis of variance showed that there were highly significant differences between the districts for the poverty index (P<0.001). As can be seen in Table A2, the households in Rautahat and Banke tended to be poorer. Higher mean scores for users were found in all districts except Nawalparasi, but these differences were only significant in two districts.

Table A2 Mean Poverty scores of users and non-users of PVS and/or COB varieties, by district in rice study.

	Poverty scores			Significance	District
District	User	Non-user	Overall	of difference	rank for HDI
Banke	5.2	4.7	5.0	ns	30
Chitwan	8.8	8.4	8.7	ns	2
Kanchanpur	9.5	8.8	9.3	ns	35
Morang	8.0	6.5	7.5	**	11
Nawalparasi	7.0	7.6	7.2	ns	37
Rautahat	3.4	2.6	3.1	*	68
Overall	6.96	6.37	6.8	*	

Source: Poverty index calculated from household survey data
* P< 0.05, ** P<0.01, ns not significant.

5. Differential impact

Farmers who reported only an increase in food self sufficiency sold no grain but had an average increase in rice self sufficiency of over 2 months (amounting to an increase of nearly 25%). This, on average, brought them into approximate rice grain self sufficiency. On average those farmers that reported an increase in grain sales were better off farmers as they have a grain surplus for sale and had twice as much cultivated rice land. Hence, they already had a rice harvest sufficient to last 22 months on average and this increased by 4 months or 18%, on average. Their grain sales increased by about 300 kg – an increase of 12%.

6. Benefits and Impact of COB Rice Varieties

The qualitative survey found that there has been a general improvement in rural livelihoods in the terai during the last few years, due to various factors such as electrification and better access to health and education services. It is impossible to separate the impacts of the new rice varieties from those of other changes that have been taking place.

In the structured household survey farmers reported benefits that varied according to the variety: these included increased grain yield, increased straw yield, better grain quality, and earlier harvest. About three quarters of farmers who were growing any COB or PVS variety reported an increase in rice grain sales (by an average of about 300 kg) or increased rice self sufficiency (by an average of about 2 months' supply).

However, it is not clear to what extent the increase in rice self-sufficiency is due to the use of new varieties. The qualitative survey also found that users of COB/PVS varieties had experienced an increase (of 2-4 months) in their rice self-sufficiency; but they attributed this change to a combination of factors – the new varieties, fertiliser use (better or more) and irrigation (better or more). The qualitative survey found that rice self-sufficiency had also increased in villages where use of the COB/PVS varieties was considered to be low. Nevertheless, throughout the survey villages COB/PVS users identified the new varieties as an important factor.

A major change in agriculture identified in the qualitative survey (Part C) is a shift from growing rice in a single cropping season to growing rice in the spring season as well, due to the use of shorter duration varieties in the main season and the availability of a suitable variety for the spring season. For example, in one village of Rautahat district:

> "Five to ten years ago, villagers only grew one crop of rice during the main season as well as wheat and maize. The land remained fallow during the spring season but now they grow one spring season rice – BG1442 which is the main source of income for the villagers. Some of the villagers repeated that the introduction of BG1442 in the village not only increased yields but also provided jobs for the villagers during what used to be the fallow period".

7. Local innovation processes

Seed spread of COB variety Barkhe 2014 from village to village To obtain a better understanding of the process of the spread of seed from village to villages an additional group discussion was held in Malhanama village in Saptari district in May 2009 by staff from FORWARD. This focused on the distribution of seed of Barkhe 2014 by farmers. A group of farmers was asked to identify all of the transactions relating to Barkhe 2014 from a single harvest. The total of 18 farmers that distributed seed did so to farmers in thirteen new villages, indicating a very high spread from village to village. On average, villages were situated 16 km away from Malhanama.

LEARNINGS and INSIGHTS

The study's finding that BG 1442 (Hardinath 1) is now in widespread use provides an illustration of the value of PVS in identifying varieties that farmers like and will adopt. It is also the latest of many examples of farmer-suitable crop varieties that remain 'in the locker' of national research organisations. This variety was introduced into Nepal about 20 years ago by the National Rice Research Program but was never released.

The early use of eight COB varieties by surveyed farmers provides strong evidence that the breeding methods that were used, although highly simplified and cheaper than conventional ones, can produce successful varieties.

The fact that COB varieties were not grown at all in a third of the 36 villages surveyed appears to be due, at least primarily, to the limited availability of seed. The quantities of COB seed supplied by the PSP projects were small, and the implication is that if larger quantities of seed had been available the rate of spread could have been more rapid. Being research projects they were not able to supply large quantities, and this suggests that an alternative funding source would have been desirable once the value of the research outputs (the COB varieties) had been demonstrated.

The limited use of COB varieties in some villages suggests that it may have been unduly early for an assessment of their impact to be undertaken.

Three different methods were used in this study to obtain information about the extent of use. This was desirable for the purposes of triangulation – cross-checking – of findings; but there was considerable inconsistency between them in the results obtained, and it appears that no one method was entirely satisfactory.

PART B REPORT ON THE STRUCTURED SURVEY

Prepared by:

John R Witcombe
CAZS-Natural Resources,
Bangor University LL57 2UW,
Wales, UK
Bikash Paudel, Rachana Devkota
and Dhruba Neupane
LI-BIRD,
Nepal

Acknowledgements

We are grateful to Parshu Ram B.K. of LI-BIRD for work on data entry, and to the enumerators - Birendra Yadav, K.P. Sapkota, K.H. Koirala, Kiran Karki, and N. Dhami. We would also like to thank LI-BIRD management for their support throughout the study.

EXECUTIVE SUMMARY

- An impact assessment was made in the Nepal terai of rice varieties identified by participatory varietal selection (PVS) or bred using client-oriented breeding (COB) in Department for International Development (DFID) Renewable Natural Resource Strategy (RNRRS) projects. In a total of 36 villages in 6 widely separated terai districts of Nepal group discussions were held that identified 2,222 rice growing households and whether they were users of at least one COB or PVS variety or not. Later, nearly 500 farmers were randomly selected for interview from these 2,222 households comprising about 10 users and 4 non users from each village. Users were asked about which varieties they grew and their impact and all households were asked questions concerning their livelihoods and resources. In addition, key informants from the Department of Agriculture District Development Offices (DADOs) were interviewed on the relative frequency of all of the rice varieties that were grown in their district, including those from PVS and COB.

- By 2008, a range of varieties from the RNRRS-funded COB rice breeding programme, which was targeted at the low altitude (terai) districts of Nepal had been adopted for upland and lowland rice ecosystems by an average of 17% of all the households in the 36 study villages.

- Farmers reported benefits that varied according to the variety and these included increased grain yield, increased straw yield, better grain quality, and earlier harvest. About three quarters of farmers who were growing any COB or PVS variety reported an increase in rice grain sales (by an average of about 300 kg) or increased rice self sufficiency (by an average of about 2 months).

- About 58% of the users came from disadvantaged groups i.e. from communities least favoured in the social system, in one of the poorest countries in the world. There were only small differences in socio-economic status between users and non-users.

- High rates of spread of seed and information were found, and current use of COB varieties in the six study districts was 15 times higher than the amount of seed that had been supplied since 2002. If current use (estimated from the group discussions and household survey and from the opinions of DADO) were to be extrapolated to all the terai districts, then a range of 1.5 to 2.4% of the rice area (15,000 – 25,000 ha) would have been devoted to COB varieties and grown by more than 150,000 households by 2008. By 2010 to 2011, assuming that rates of spread found in the study continue, then up to 100,000 ha could be occupied by COB varieties.

- Variety BG 1442 (Hardinath 1) was introduced two decades ago by the National Rice Research Program (NRRP) into Nepal but never released. It was identified by farmers as a useful variety in PVS trials in the RNRRS projects and was subsequently widely promoted within these projects. It was found to be used more extensively than any of the individual COB varieties. Hence, the total area under COB and PVS varieties combined would be expected to exceed 100,000 ha and 150,000 households by 2010 or 2011.

INTRODUCTION

This report contributes to an examination of the uptake and impact of rice varieties from DFID Plant Sciences Research Programme RNRRS projects in Nepal. These projects evolved and used the techniques of client-oriented breeding (COB) and participatory varietal selection (PVS) that are described below. Both rice COB and PVS were carried out by a non governmental organisation (NGO), the Local Initiatives for Biodiversity in Research and Development (LI-BIRD), Nepal, and CAZS-Natural Resources, UK, (CAZS-NR); and PVS by the Forum for Rural Welfare and Agricultural Reform for Development (FORWARD).

Rice was targeted as it is the most important crop in the terai and covers about 1 million of the total 1.2 million ha of rice grown in Nepal. The vast majority is grown in the main season (June to November) and most is grown under rainfed conditions. There is a much smaller area of February sown (*Chaite*) rice (about 0.1 million ha) that is grown under irrigation.

The participatory research in rice in the RNRRS projects in Nepal was termed participatory crop improvement (PCI) and had two major components i.e., participatory varietal selection and client-oriented breeding.

Participatory varietal selection (PVS)

In conventional plant breeding few varieties ever make it to the stage of on-farm testing and even fewer are formally recommended and released. Therefore, farmers have access to a very limited choice of varieties. Participatory varietal selection (PVS) gives farmers more choice by providing seed of new varieties that are carefully selected to meet identified farmers' needs.

The PVS programme in Nepal had four steps:

1. First, farmers' needs in new cultivars were identified. This was done, in Chitwan and Nawalparasi districts, by participatory rural appraisals (PRA) that identified the varieties that farmers were growing and their important traits. In addition project scientists examined farmers' crops around harvest time to learn the traits that suitable varieties might have.
2. Project scientists then searched for suitable varieties that best matched these needs. The varieties that were included in the trials were:
    - pipeline varieties from the national research system (e.g., BG 1442),
    - varieties that had been introduced into Nepal in the farmers' own innovation system (e.g., Swarna), and
    - released varieties from India (e.g., Pant Dhan 10) that had not been discovered by farmers in their own innovation system.
    - Later, varieties that had come from the COB breeding programme (see below) were tested in PVS trials.
3. The project scientists and farmers collaborated on testing the new varieties on farmers' fields using a participatory trials system.
4. When promising varieties were identified these were scaled up, using the limited resources available in the research projects. Despite such constraints, tonnes of seed of varieties such as BG 1442 were distributed across many terai districts.

There are many ways of carrying out highly participatory trials. The Nepal RNRRS projects employed a mother (Fig. 1) and baby trials system (Snapp, 1999). The mother trials were single-replicates of all of the entries grown under a single management regime (the farmer's own). The baby trials were much simpler and had

only one test entry from the mother trials grown alongside a local control. However, across all of the baby trials all of the varieties in the mother trials were included and there were several replicates of each entry (Fig. 2). In the simplest design for a baby trial, originally called informal research and development (IRD) by Lumle Agricultural Research Centre, Nepal (Joshi and Sthapit, 1990) small packets of seed are informally distributed to many farmers. This is also called extensive PVS (Gridley *et al.*, 2002). IRD was used by the RNRRS projects in Nepal and a comparison in Chitwan and Nawalparasi showed it gave similar results to the more elaborate baby or mother trials but used fewer resources (Joshi and Witcombe, 2002). The latter have been used because they produce the kinds of systematic data required in varietal release proposals.

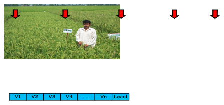

Fig. 1. An example of a mother trial in Nepal with the trial design shown (all entries in a single replicate) and the individual plots indicated by the arrows.

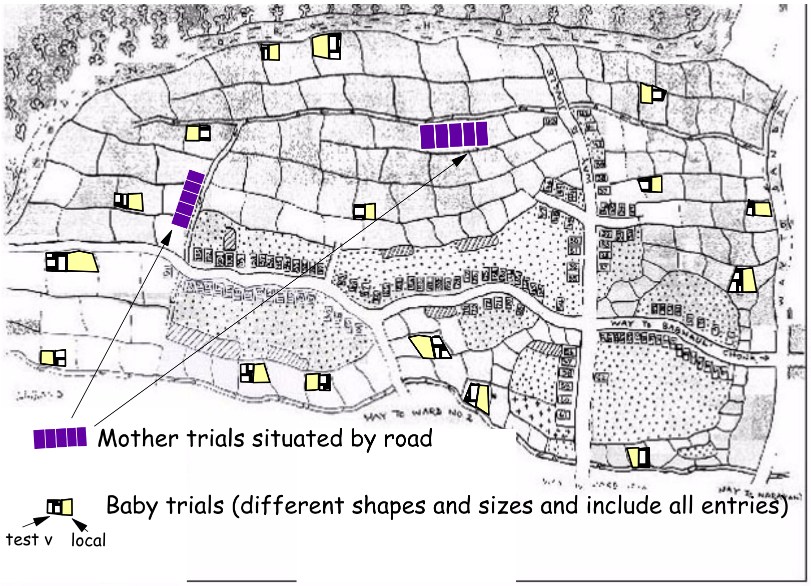

Fig. 2. An outline as to how a mother and baby trials systems might look in a single village. Here there are two replicates of the mother trials and 16 baby trials, with each of the four test entries in the mother trial replicated four times.

PVS has major advantages over on-station varietal testing:

1. It allows farmers to evaluate varieties for all traits and to make trade-offs between traits e.g., grain yield against straw yield, maturity, and grain quality.
2. It tests varieties under realistic management conditions.

3. It tests varieties across more of the physical niches in which the crop is grown because the trials are replicated across more locations and across social niches where food preferences might vary.
4. It promotes use.

Client oriented breeding

The PVS trials in Nepal were restricted to testing varieties that were already available from various sources. Given that this existing varietal diversity was restricted – for example, it only included one aromatic variety and this was rejected by farmers – a client oriented breeding programme was started in the Nepal RNRRS project. Initially client-oriented breeding was called participatory plant breeding (PPB) to describe the *activity* of involving farmers (Witcombe et al., 1996). Later, client-oriented breeding (COB) was adopted as a better term to explain the *purpose* of involving farmers (Witcombe et al.2005). More strictly, it needs to be called highly client-oriented breeding as many plant breeders would argue that since all breeding programmes have some degree of client orientation it is the extent that varies.

The breeding method adopted in the Nepal project (Joshi et al., 2002; Gyawali et al., 2002) used many fewer crosses than is conventionally the case, and large populations from those crosses were grown in the segregating generations (Witcombe and Virk, 2001). This was done because it was not only theoretically sound but it fitted much better with farmer participation – farmers can easily grow one or two populations on large areas but would find it difficult to grow many small populations. Selection in the segregating generations was done in farmers' fields, primarily in Chitwan. Grain quality testing took place with the end users before yield trials. This saved resources as it is cheaper and quicker to check the quality of a variety than to grow it for a whole season in replicated yield trials. Once a new variety from the breeding programme was produced it was immediately tested by farmers in the villages where the project was working, predominantly in Chitwan and Nawalparasi, in participatory varietal selection (PVS) trials. The fields for the trials were selected by project scientists and farmers to represent the various domains (upland, medium and lowland) to match the varieties to their potential domains. As many farmers were involved they were assumed to be reasonably representative. Hence, no particular effort was made to deliberately attempt to select representative farmers although attention was paid to involve women farmers.

The report concentrates mainly on the use of eight varieties produced by COB (Table 1). It only considers one of four varieties identified by PVS, namely BG 1442, as it is the only one with significant uptake where the contribution to its spread could be clearly attributed to the RNRRS projects. In the other cases the variety was: either in the farmers' own rice innovation system, i.e., Swarna; or was also promoted by the national programme e.g., Rampur Masuli; or the uptake was low i.e., Pant Dhan 10.

Table 1. The varieties produced by client-oriented breeding that were included in the study.

Cross Variety	Maturity (days)	Adaptation to land type	Adaptation to season	Major trait
***Cross 1*†**				
Barkhe 1027	Early (125 days))	Upland & medium upland	Main	Combination of earliness, yield and good eating quality.
Barkhe 2014	Medium (140 days)	Medium	Main	Superior replacement for Kanchi Masuli. Phenotypically similar but has higher yield and matures earlier.
Barkhe 3004	Late (155 days)	Medium & lowland	Main	High yield and disease resistance.
***Cross 2*†**				
Judi 572	Early (125 days)	Upland	Main & Chaite	Similar to Barkhe 1027 but has better lodging resistance but poorer eating quality.
***Cross 3*†**				
Barkhe 2001	Medium (130 days)	Medium	Main	Excellent eating quality and high yield.
Barkhe 2024	Medium (140 days)	Medium	Main	Excellent eating quality and high yield.
Sugandha 1	Early (125 days)	Upland	Main	Aromatic with excellent eating quality.
Sunaulo Sugandha	late (150 days)	Medium & lowland	Main	Aromatic with excellent eating quality and very high yield compared with other aromatic alternatives.

†The three crosses are described below.

When this impact assessment was started in 2008 only three crosses had been made with sufficient time to produce new varieties that could have been used by farmers to any detectable extent.

- The first cross, Kalinga III/IR64, was made in IRRI in 1996 at the request of CAZS-NR and seed was brought into Nepal from India at the F_3 generation.
    - This cross produced varieties Barkhe 1027, Barkhe 2014, Barkhe 3004.
- A second cross, Radha 32/Kalinga III, was made in Nepal in 1998. It was chosen as both of the parents were liked by farmers in PVS trials in Chitwan but they had weaknesses that could be eliminated through the complementary traits of the parents by making this cross.
    - This produced variety Judi 572.
- A mutation breeding programme in Pusa Basmati 1 was equivalent in effort to that of a third cross. Variety Pusa Basmati 1 is an aromatic, dwarf, rice variety from India. Seed of it was irradiated in 1998 to produce mutations but, it was later found that the irradiated seed came from Pusa Basmati 1 plants that had naturally crossed to other varieties in the field. This out-crossing, almost certainly, produced more variation than the mutations and there was huge

diversity in the material. Hence, only one of the parents (Pusa Basmati 1) can be known of any variety resulting from out-crossed, irradiated Pusa Basmati 1.

- This cross produced varieties Barkhe 2001, Barkhe 2024, Sugandha 1 and Sunaulo Sugandha.

Major events in the projects involved the identification of early duration variety BG 1442 by PVS and the release of two varieties from COB (Table 2).

Table 2. Timeline of major events in the PVS and COB activities

Process	Year	Event
PVS	1998	• RNRRS project started in Chitwan
		• BG 1442 tested in PVS trials
	1999	• BG 1442 identified as preferred variety
	2001	• Large scale seed supply of BG 1442 by project
	2004	• BG 1442 released by NARC as Hardinath 1
COB	1996	• KIII/IR64 cross made at IRRI (cross 1)
	1998	• RNNRS project started in Chitwan
		• Seed of first cross at F_3 in Nepal
		• Radha32/Kalinga III cross made in Nepal (cross 2)
		• Pusa Basmati 1 irradiated (cross 3)
	2001	• First COB variety from cross 1 tested in PVS trials
	2002	• First COB varieties from cross 2 and cross 3 in PVS trials
		• Scaling up of first promising varieties began on small scale
	2006	• First COB variety from RNRRS project released (Barkhe 3004)
	2008	• Second COB variety from RNRRS project released (Sunaulo Sugandha)

Assessment of poverty focus and potential impact at the start of the RNRRS projects

At the time of the RNRRS projects the poverty focus of the research and the potential impact of producing new rainfed rice cultivars was assessed. The UN has compiled a poverty and deprivation index for all of the districts of Nepal. The average index for Nepal and for the Terai as a whole was 0.47 (on a scale of 0 for least developed to 1 for most developed). This overall average development in the Terai was only because a few districts were highly developed (Fig. 3). Of the 20 Terai districts, 14 were average, or below average, in development. Rautahat, the poorest district in the Terai has a population of over 500,000 and was the fourth poorest district in Nepal. Several population groups in the Terai, including the Tharus and Musahars, have been disadvantaged for generations and remain so. Moreover, the improvement in the human development index from 1996 to 2000 in the Terai as a whole (12.1%) was lower than in the hills (17.5%).

For the two project districts, one was below average (Nawalparasi) and the other (Chitwan) was better off.

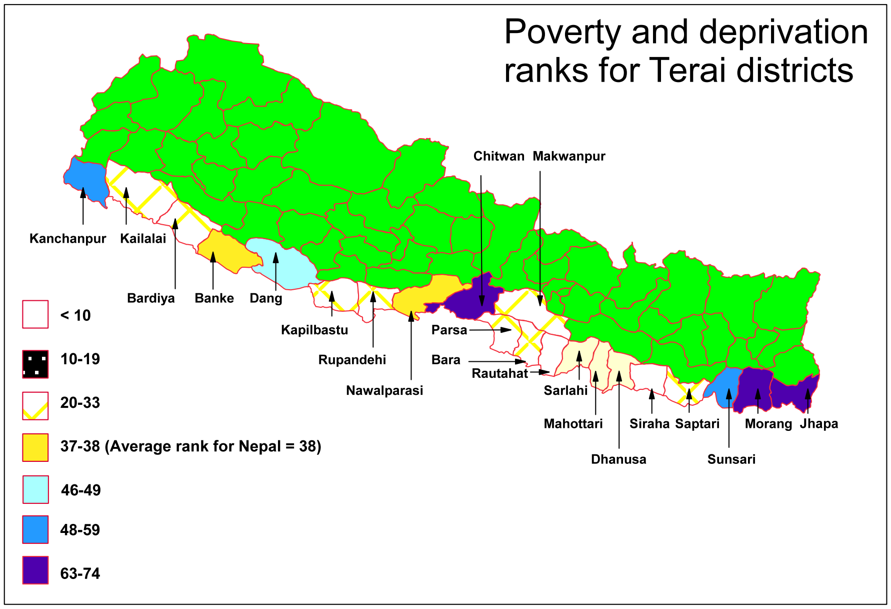

Fig. 3. Poverty and deprivation index ranks (1 = least developed district, 75 = most developed district) for the Terai districts, 2001. Source UNDP 2002.

More recent data show there has been some improvement in the Terai districts relative to the hills but one third of the Terai districts were still among the less developed (Fig. 4).

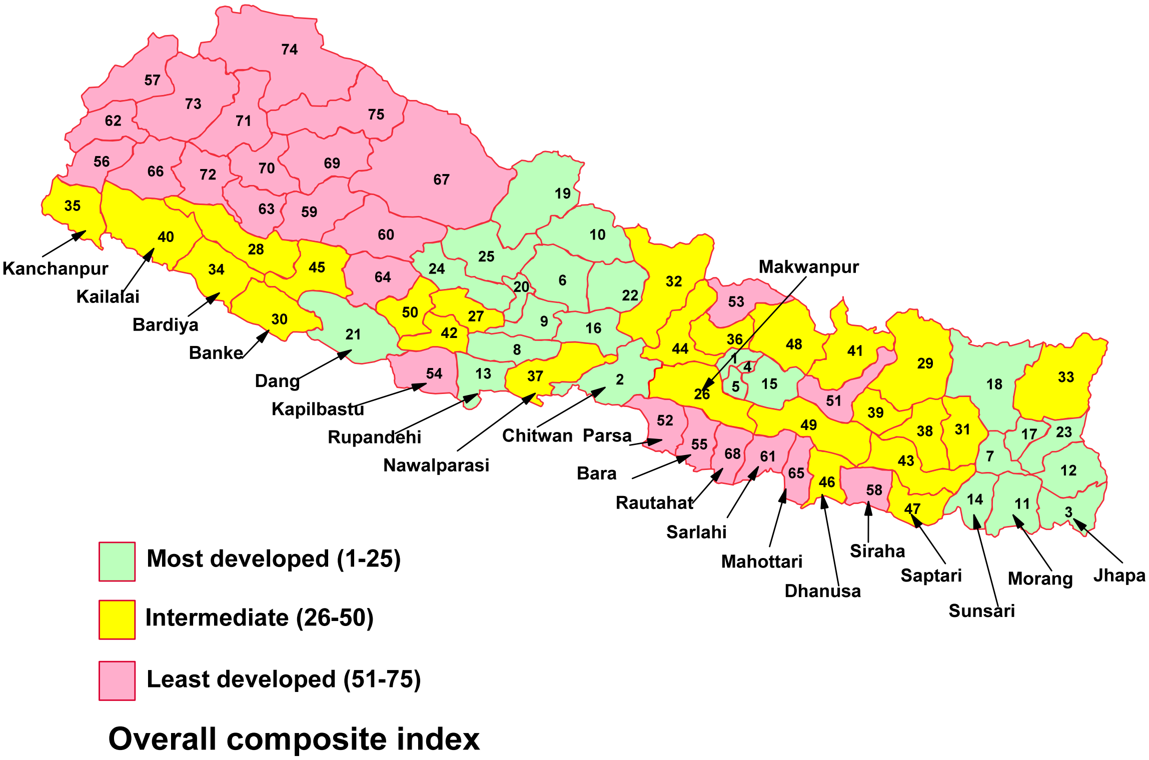

Fig. 4. Poverty in Nepal using 2003 data (BBS & ICIMOD, 2003).

Food self sufficiency

The UN does not provide data on food self sufficiency but RNRRS project baseline data were obtained for households in eight villages in Chitwan and Nawalparasi (Rana et al., 2004). Despite the relatively high degree of development in these two districts, the majority of farmers in the 8 studied villages were food deficit (Fig. 5).

From interviews with key participants, the landholding of food-deficit farmers was very low, and was usually less than 0.5 ha. Food balance farmers had about 1 ha of land, but this varied from village to village depending on the productivity (largely determined by the availability of irrigation water) of the village rice fields.

The key informants commonly mentioned the importance of off-farm income as a determinant of the wealth ranking of households. Nonetheless, rice production was important for people’s livelihoods; and increased production can provide more opportunities for earning income from labour since harvesting and threshing are predominantly manual operations in the Terai.

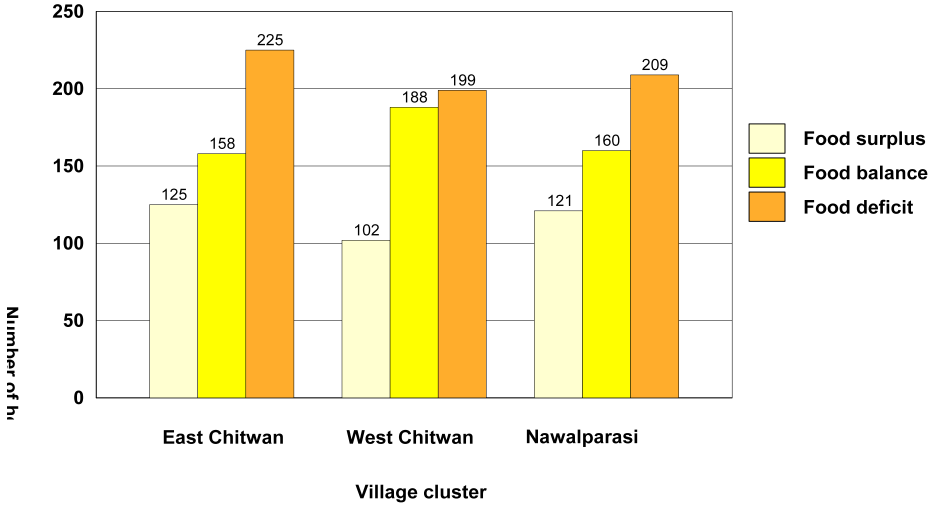

Fig. 5. Distribution of households according to whether they are food-deficit, food balance, or food surplus. Two villages from east Chitwan cluster, 3 villages from west Chitwan cluster, and 3 villages from Nawalparasi cluster (Rana et al., 2004).

Although the RNRRS projects were initially designed to test participatory technology development (PTD) in high-potential production systems much of the project area was less favourable for agriculture. Rice was grown under rainfed conditions or with only limited quantities of irrigation water. It was estimated that about 70% of the main-season rice in the Terai was grown under rainfed and limited irrigation water conditions (Fig. 6).

Participatory household surveys, in the project villages of Chitwan and Nawalparasi, revealed that farmers were growing old varieties in both rice-growing seasons, sometimes as much as 40 years old (Witcombe *et al*., 2001). The varietal diversity was often extremely low with the most popular variety occupying the majority - sometimes over 90% - of the area (Fig. 7).

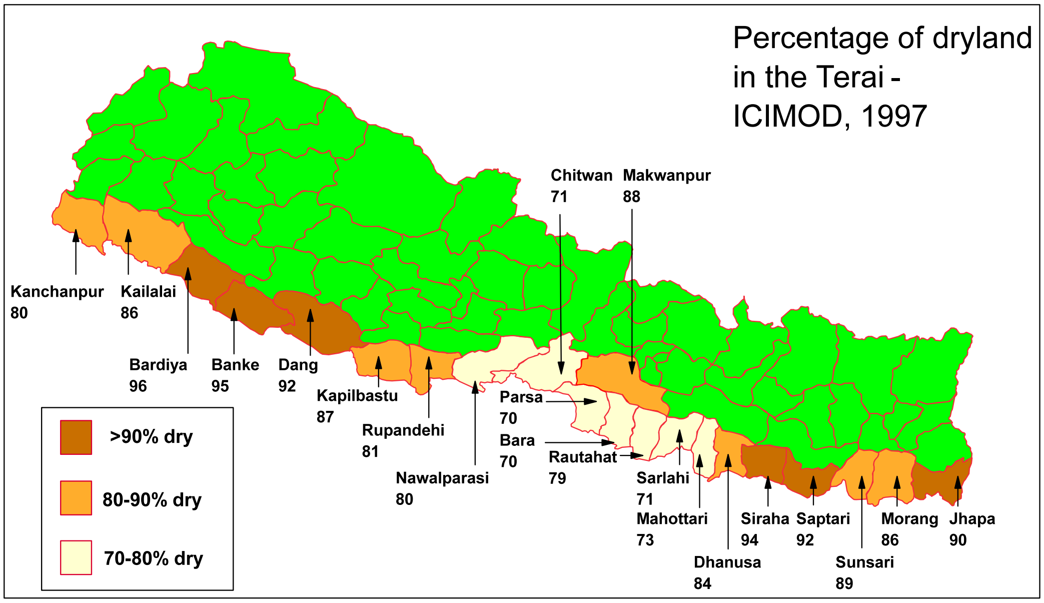

Fig. 6. Percentage of land that is without perennial irrigation according to ICIMOD, 1997.

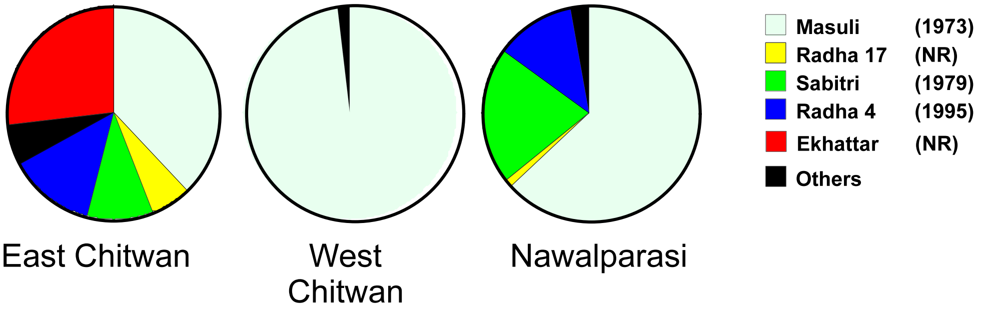

Fig. 7. Area under main-season rice varieties in three village clusters of East Chitwan, West Chitwan and Nawalparasi, 1997. (Year of release in parentheses; NR = not released).

The National Program has released relatively few varieties for the Terai particularly considering its importance in area, and the rate of release for the Terai had declined in the years preceding the RNRRS projects. Moreover, only a minority of the varieties that had been released have been widely used by farmers. Instead, many of the most widely grown varieties, such as Sarju 52 in the west of the country, were farmers' introductions, most of which originated from India. Others were varieties that had not been officially released but had been introduced by the Nepalese research system, such as Kanchhi Masuli†, in the east of the country, and Ekhattar and Radha 17. Sarju 52 and Kanchhi Masuli are two of the most widely grown varieties in the Terai and have spread entirely from farmer-to-farmer, without official support.

---

†Kanchi Masuli was originally from India and also known by Nepalese farmers as Jhapali Masuli, Aus Masuli, Banspate, and Bans dhan. Ekhattar and Radha 17 were tested for several years by NARC in yield trials and in farmers' fields while Kanchhi Masuli was tested in yield trials. None of them were released.

METHODS

Overview of methods used

The study consisted of two main parts:

- A scoping study on overall varietal distribution in the terai (the low altitude area in the south of the country that borders India). This used key informants from District Agriculture Development Offices (DADOs) who gave their estimates of use of rice varieties including those from the PCI project's COB and PVS programme, and
- A study on the use and spread of varieties from PVS and COB in six purposively selected districts (Fig. 8) using survey techniques, namely, group discussions and individual interviews of household heads or their spouses, in villages where project activities (or scaling up by partners) had been done.

Scoping study on varietal diversity

The area for this study was all 20 terai districts plus the inner terai district of Makwanpur. In a series of interviews conducted by telephone and sometimes by personal visit a key informant in each of the 21 District Agricultural Development Offices (DADOs) was interviewed about the varietal composition of rice in the district by either Dr Krishna Joshi of CAZS-NR or by Dr KK Lal, a consultant to CAZS-NR who had retired from a senior position in NARC. The key informants were also asked to give estimates of any use of varieties from the COB programme. The interviews took place from March to April 2008 and in many cases follow up interviews were done to allow time for discussions, mainly with colleagues within the DADOs, to arrive at a consensus. The data were compiled on Excel spreadsheets and most of the data were summarised by the use of pivot tables.

Supporting information was gathered from Nepal Agricultural Research Council (NARC) stations, seed suppliers and producers, and from non-governmental organisation (NGOs).

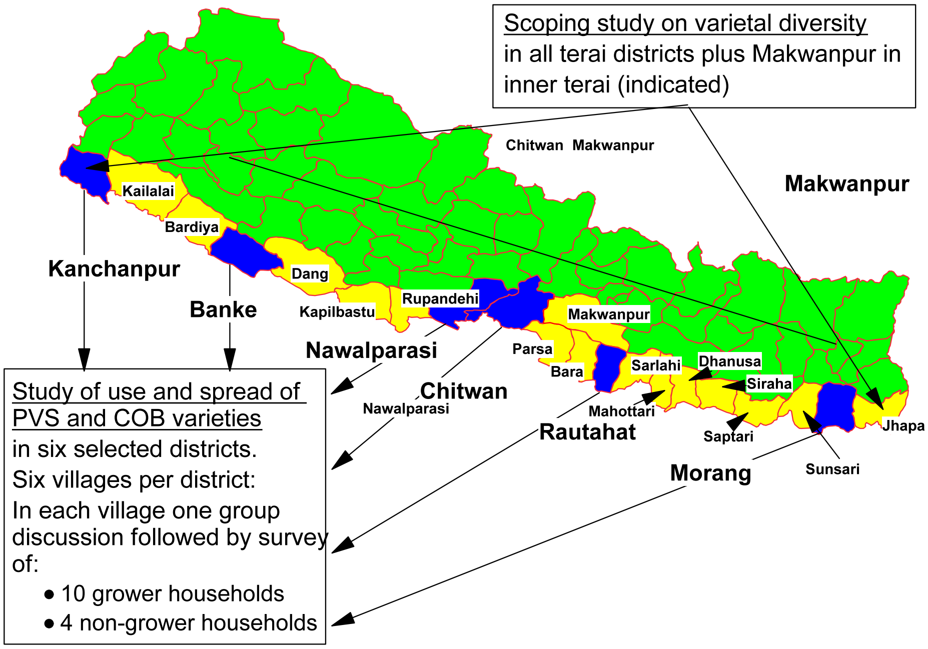

Fig. 8. The locations of two studies.

Study of use and spread of PVS and COB varieties

Identification of four high-to-medium intervention districts for sampling
Villages were sampled in four districts where the RNRRS project had relatively high and early levels of intervention in terms of activities and scaling up. There were a total of 10 districts that met the following criteria:

- A district where the first project intervention into the village had been made by 2002 at the latest, as measurable impact was considered to be more likely from earlier interventions as there was more time for farmer-to-farmer spread (Appendix 1).
- A district where project records showed that in the district as a whole there had been significant distribution of seeds of both PVS and COB varieties (Appendix 1). This seed was supplied for Mother and Baby trials, for Informal Research and Development (IRD) and for community-based seed production.

Ten districts met these criteria and are listed from west to east below (their locations are shown in Fig. 1). Of these ten, Siraha and Saptari were excluded as they were selected in another Rainfed Agriculture Impact Assessment Study on rainfed rabi cropping (RRC). Of the eight remaining, the central districts of Chitwan and Nawalparasi districts were selected because they were also where PVS and COB activities started in 1997. Then the most westerly (Kanchanpur) and easterly (Morang) districts were chosen to give the greatest spread of districts from the east to the west to capture any differences in rice growing conditions across the terai that occur as a result of differences in climate.

District									
**Kanchanpur**	Kailali	**Nawalparasi**	**Chitwan**	Mahottari	Dhanusha	Siraha	Saptari	Sunsari	**Morang**

The four districts that were selected for medium-to-high intervention are shown in bold font.

Identification of two low-intervention districts for sampling
Two districts were selected from the lowest intervention districts for comparative purposes. The six lowest were Rautahat, Makwanpur, Dang, Banke, Sarlahi and Kapilbastu (Appendix 1). Of these, Makwanpur is atypical because it is inner terai and was also adjacent to already-selected Chitwan, and Sarlahi was a district where there was early intervention in 2001 biasing it towards higher intervention. Of the four remaining there were the three adjacent districts of Banke, Dang and Kapilbastu in the west (from which Banke was selected) and Rautahat in the east.

The six selected districts are summarised in Table 3.

Table 3. Some features of the districts selected for the study

District	Geographic region	Year of entry	Extent of intervention	Poverty and deprivation index†
Morang	East	2002	Medium	25
Rautahat	East	2002	Low	68
Chitwan	Centre	1997	High	4
Nawalparasi	Centre	1997	Medium	36
Banke	West	2002	Low	16
Kanchanpur	West	2002	High	19

†The poverty and deprivation index of Nepal ranks districts on a 1-75 scale where 1 = best 75 =worst. (CBS & ICIMOD, 2003)

Selection of villages within the districts

A list of possible villages in each study district was prepared on the basis of one or more of three information sources:

a. Reports and publications of the RNRRS participatory crop improvement (PCI) project.
b. Information from key informants such as project staff, DADO officers, and local farmers.
c. Reports of partner NGOs (such as SUPPORT Foundation in Kanchanpur, and FORWARD in Morang).

The listed villages included those with direct intervention either by LI-BIRD, DADO or another partner. From the list of all the possible villages in a district, the key informants from DADO, other NGO and project staff were asked to rank the villages in order of the greatest likelihood of finding users of COB and PVS varieties and where seeds of COB and/or PVS seeds must have been distributed. These were opinions that only took account of the individual knowledge of the people concerned as there was no time for the concerned individuals to consult with others. In each of the six districts 6 villages were then selected (Figs. 9, 10, 11).

The survey methods within each village: Group discussions

Group discussions were conducted in these prioritised villages by enumerators from LI-BIRD following a structured survey tool (Appendix 2). Participants from the village consisted of key informants and farmers and they varied from a minimum of 9 to a maximum of 42 in each village (Table 4). In the group discussions the extent of use of the varieties, and the approximate area coverage of the varieties in each season by land type was determined.

Then a list of farmers who were users or non-users was prepared. To do this the boundary for the study area for the village was set by the participants by excluding areas for which they had no or little information on the rice varieties that grew there. A rough map was then drawn for this area by the group on paper, which indicated all thoroughfare (lanes) and major features. By each lane on the map, the names of household heads were listed. If there was no-one in the group present from a lane, it was not included in the analysis to reduce the possible errors of key informants providing information on households they were not very familiar with. This resulted in a list of rice growing households in the selected areas of the village. For each household, information was collected from the group on whether or not, in their opinion, it was growing PVS varieties, COB varieties or both or neither (Appendix 2). A user was defined as a farmer who grew at least one of 11 COB

varieties (Table 1)[1] or 4 PVS varieties (BG 1442, Pant Dhan 10, Swarna and Rampur Masuli) for either the spring of 2008 or the main season of 2007 or both. The group discussions identified a total of 2,222 households in a total of 36 villages in six districts and hence percentage use data were all calculated using 2,222 as the total sample size.

These 36 villages had at least 12 users each of rice COB and/or PVS varieties. Any village with less than this number was dropped to allow a sufficient sample for the subsequent household survey. In no district were more than two villages visited where the group identified insufficient users. When villages could not be included they were replaced by other villages

The survey methods within each village: Household survey

The 10 user and 4 non-user households that would be interviewed in each village were randomly selected, using the random number function of a calculator, from the lists of users and non-users of PVS or COB varieties that were prepared in the group discussions. This gave a target total of 84 households in each district (i.e. 60 households who were users and 24 who were non-users) to be interviewed. If a household head or spouse could not be found the household was not replaced (households with available heads or their spouses may have produced bias as the simple fact that they were available may have meant they were not representative of those who were unavailable) so the actual totals were slightly lower (Table 4).

These sampled farmers were interviewed by LI-BIRD staff using a questionnaire (see Annex 1 for a user and Annex 2 for a non-user).

Farmers were defined as users if they grew either a COB or a PVS variety in either the main season or the *Chaite* season. However, the analyses presented in this report are only for the main season varieties – except that for the questions on the benefits of the PVS and COB varieties both main and *Chaite* season varieties were considered.

Of the 344 ‘users’ 57 only grew a *Chaite* season variety (almost invariably BG 1442) so the actual sample size of main-season users was 287 (Table 5). In the text reference is made as to which sample is being considered e.g., all users or just main-season COB variety users.

[1] In addition to the 8 COB varieties in Table 1, Barkhe 2044, Barkhe 3019 and Judi 565 were included. There was a total of only 11 cases of these varieties in the household survey and to simplify the analysis these infrequent varieties were not included.

Table 4. Sampling frame: Survey villages and numbers of farmers that were involved in the household surveys and group discussions, 2008.

District	Village Development Committee (VDC)	Village	Number			
			Users†	Non-users	Total	Group discussion size
Kanchanpur	Daiji-1	Sawadaiji	10	4	14	15
	Jhalari -2 & 7	Nagartol	10	4	14	14
	Krishnapur-7	Singhpur	10	4	14	16
	Suda-1	Lalpur	10	4	14	13
	Suda-5	Sundarpur	10	4	14	15
	Tribhuwanwasti- 5	Ghagaun	10	4	14	18
District total			*60*	*24*	*84*	*91*
Banke	Bhawaniyapur	Balegaun	10	4	14	14
	Puraina	Jhagarpur	10	4	14	18
	Puraina	Puraina	10	4	14	15
	Udaypur	Lihar	10	4	14	20
	Udaypur	Lonianpur	10	4	14	24
	Udaypur	Surajpur	10	4	14	14
District total			*60*	*24*	*84*	*105*
Nawalparasi	Argheuli-1	Argheuli	8	4	12	13
	Argheuli-5	Sherjung	10	4	14	12
	Kalawa-3	Abhiyun	8	3	11	12
	Kawasoti-4	Taruwa	8	4	12	16
	Nayabelani-5	Arunkhola	8	4	12	11
	Tamsariya-5	Tareni	9	3	12	9
District total			*51*	*22*	*73*	*73*
Chitwan	Gitanagar-4	Debnagar	10	4	14	9
	Gitanagar-5	Amarwasti	10	3	13	11
	Parbatipur-8	Parbatipur	10	3	13	11
	Patihani-8	Patihani	9	4	13	12
	Pithuwa-7	Madhavpur	10	3	13	14
	Ratnanagar-16††	Bakular,Nipani	10	4	14	10
District total			*59*	*21*	*80*	*67*
Rautahat	Bagahi	Phatepur Tol	10	4	14	19
	Bhediyahi	Bhediyahi	10	4	14	34
	Dumariya Matiaun	Dumariyatol	10	4	14	31
	Jayanagar	Jayanagar	10	4	14	24
	Mahammadpur	Shivanagar	10	4	14	25
	Rajpur Tulsi	Rajpur Tulsi	10	4	14	42
District total			*60*	*24*	*84*	*175*
Morang	Amagachi	Bahuban	9	4	14	28
	Babiyabirta	Bhaluajhoda	10	4	14	16
	Budhnagar	Damadighi	9	4	14	18
	Rangeli	Madhubari	8	4	14	18
	Shanischare	Aayabari	8	4	14	16
	Sorhabhagh	Karsiya	10	4	14	21
District total			*54*	*24*	*784*	*117*
Grand Total			344	139	483	

†Users are defined across both seasons (main and/or *Chaite*)
††Ratnagar Municipality

Table 5. Sampling frame: Number of users who were interviewed in each district shown by season and whether they grew a PVS and/or a COB variety.

Category	District						
	Kanch-anpur	Banke	Nawal-parasi	Chitwan	Rautahat	Morang	Total
PVS† users, main season 2008	23	45	23	32	54	28	205
COB† users, main season 2008	41	17	33	21	7	17	136
User of COB or PVS or both, main season 2008	51	57	42	48	55	34	287
Only PVS variety in 2007 *Chaite* season	9	3	9	11	5	20	57
Total 'users'	60	60	51	59	60	54	344

†PVS user could also be a COB user and *vice versa*

Household survey using transects defined by the Global Positioning System

In addition to the scoping study and the impact study, the MIL component of RiUP funded a repeat of household surveys in Chitwan that had been made in 2005 and 2006 using a randomly selected set of transects defined by using the global positioning system (GPS) (Joshi et al. 2007). The details of the complex GPS transect method used in this random survey are not repeated here and the reader is referred to the original publication and the separate results of the 2007 household survey (CAZS-NR & LI-BIRD, 2007).

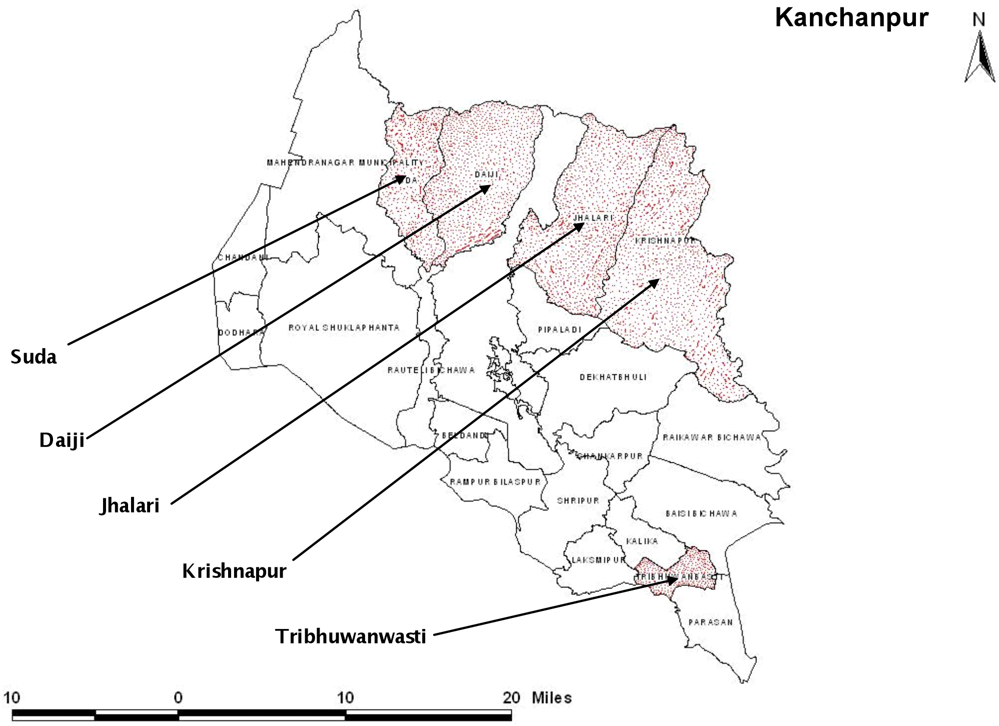

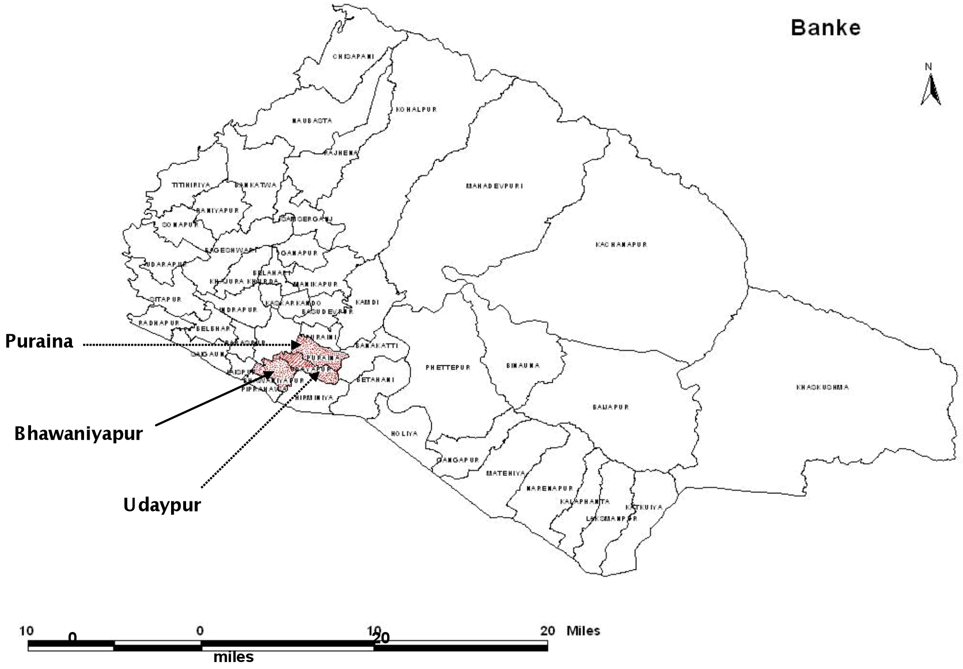

Fig 9. Study villages in Kanchanpur and Banke districts.

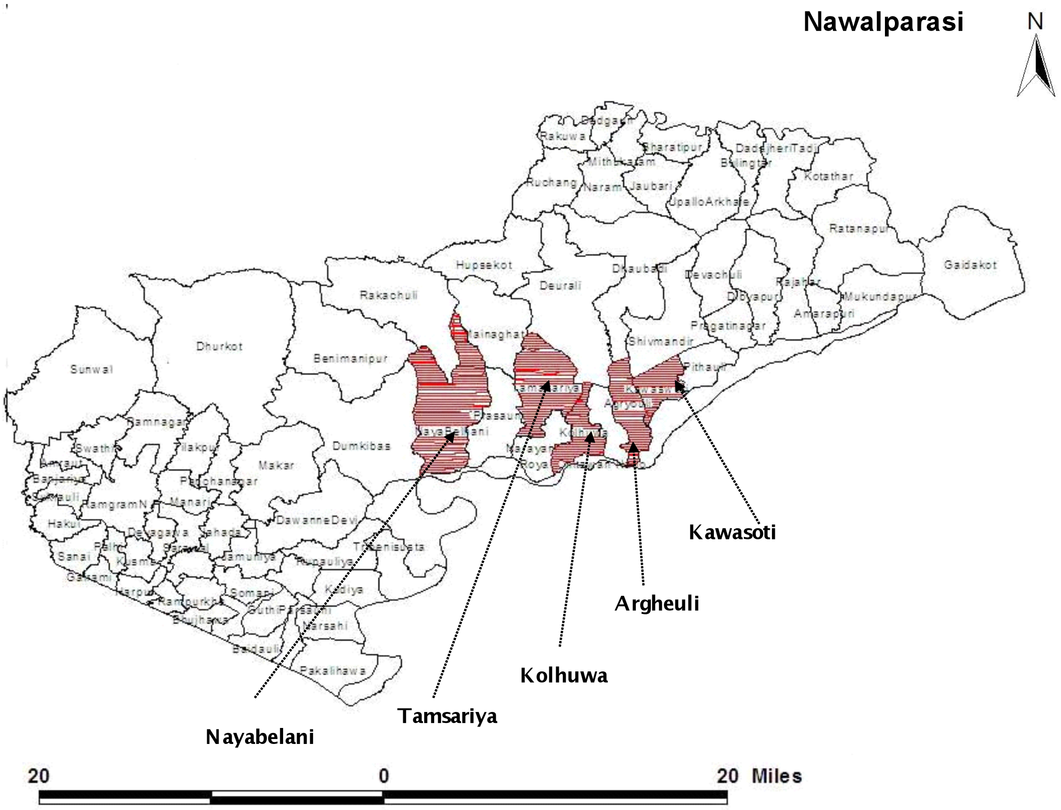

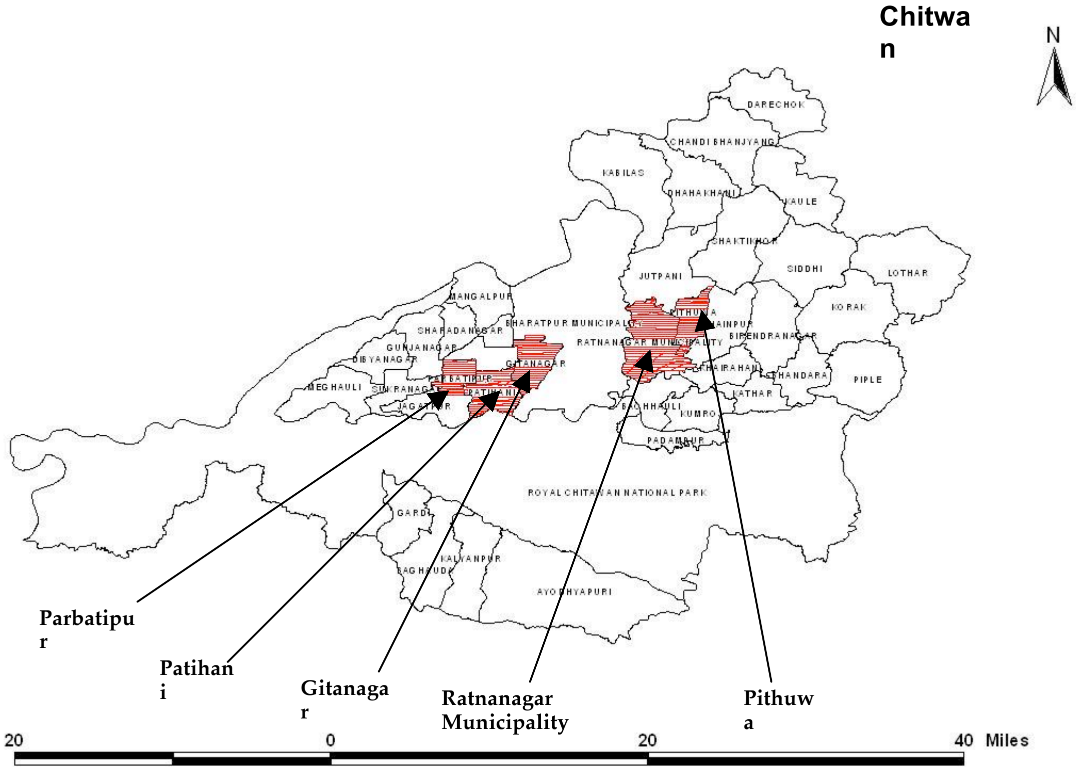

Fig 10. Study villages in Nawalparasi and Chitwan districts.

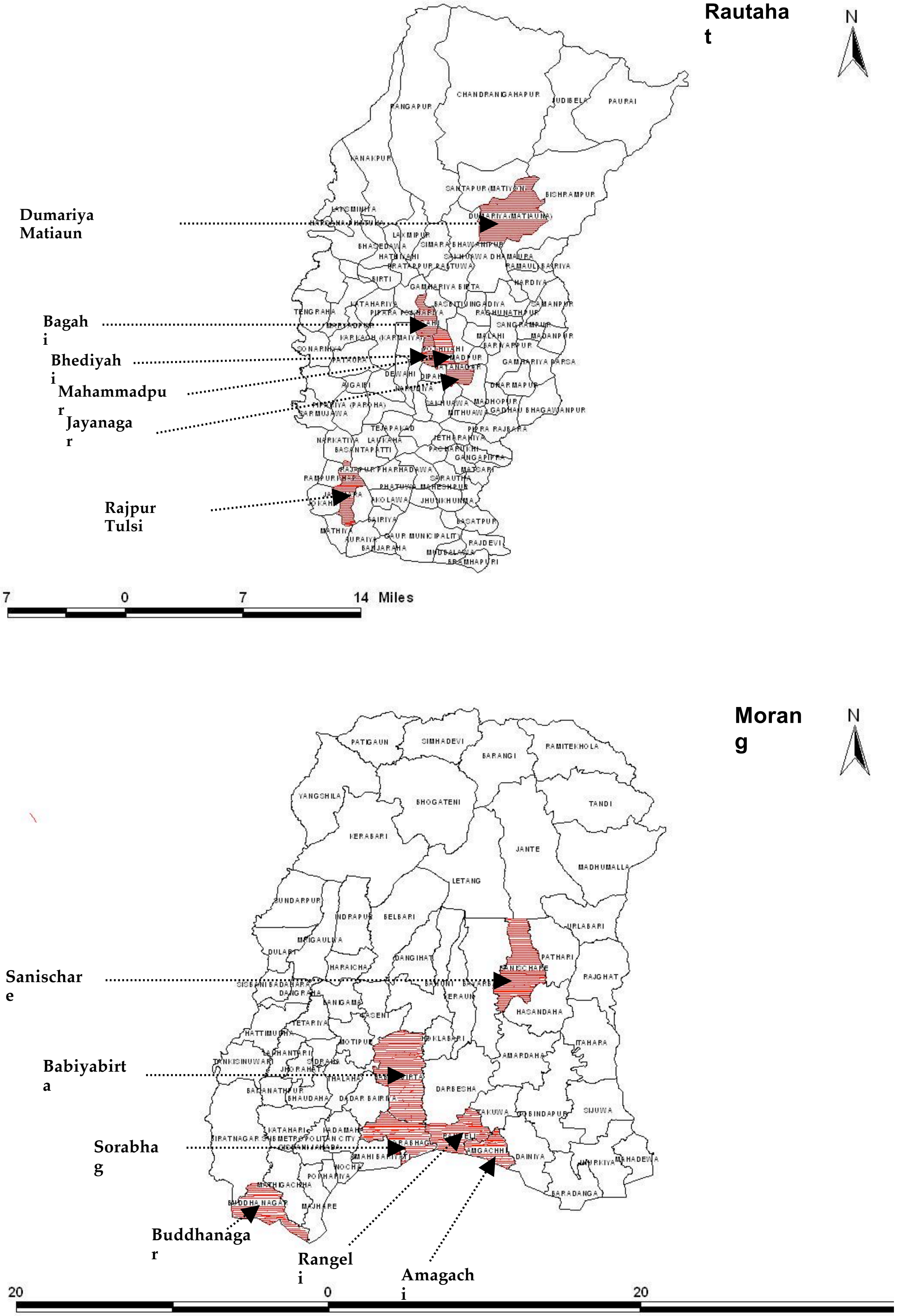

Fig 11. Study villages in Rautahat and Morang districts.

Poverty index

A poverty index was constructed to enable the study to distinguish between poor and wealthier households among those households surveyed. The poverty index did not attempt to place households in relation to the poverty line established by the government of Nepal as the data demands for such an exercise were too big.

There were six indicators selected for the poverty index, each of which was given a set of possible scores (Table 6). A total score (overall poverty index), for which the maximum possible was 23, was calculated for each household derived from the sum of the individual scores.

Table 6. Scores for the poverty indicators.

	Score							
**Indicator**	**-1**	**0**	**1**	**2**	**3**	**4**	**5**	**6**
Livestock units†		<1	1-<3	3-<5	5-<10	>=10		
Food grain production per capita††		<180	180-<365	365-<730		>=730		
Roof material		Thatch	Tile	Tin	Concrete			
Jobholders in the family		No job		1 job		2 jobs		3 or more
Tractor ownership		No tractor					Own tractor	
Seasonal unskilled labour migration	Migration		No migration					

†*Livestock units.* The weighted sum of all livestock owned by the household by four animal types. The relative weightings were: Cows, buffaloes, horses, donkeys = 1; Goats, sheep = 0.1; Poultry = 0.01; Pigeons = 0.005. The number of animals owned of each type were multiplied by the corresponding weight and the products added up. Thresholds for this indicator index were derived from consultation of secondary sources (NSS Report No. 493(59/18.1/1), Maltsoglou, I and Taniguchi, K., 2004) and consultations with key informants from the partner organisations participating in the study.
††*Food production per capita.* The total quantity (kg) of food grains (cereals and legumes) produced in the 2007-2008 season (including grain produced for consumption and sale) was divided by the number of adult equivalents per household. Adult equivalents per household were calculated as a weighted sum using the following weights: Adults = 1; 10 – 17 years = 1; children under 10 = 0.1.

RESULTS AND DISCUSSION

Extent of Use of the COB and PVS Varieties in the Household survey Area

Group discussions

The participants in the group discussions in the 36 villages listed a total of 2,222 rice users (Table 7). Overall, the group discussions revealed a significant use of RNRRS identified and promoted varieties. PVS varieties were more widely used than COB varieties i.e., 36% of rice growing households grew a PVS variety compared with 10% for COB varieties. This was related to the longer time in which PVS varieties were available as the research project started with PVS and first seed distribution took place in 1998 whereas the first COB varieties were not distributed until 2001 and then in only small quantities.

There was no correlation between use levels in the sampled villages and the levels of intervention at the district level (Table 7). Why this was so could be due to a combination of:

- A poor correlation between the district level data for seed distribution and the actual seed distribution in the individual sampled villages. The most likely cause of such a poor correlation is extensive but unrecorded seed distribution by NGOs, DADOs and the private sector in the sampled villages in some of the districts
- Some differences in the suitability of the varieties for districts. Such differences could have been exaggerated by the unequal access to seed of the varieties. Hence, some districts where there was lower use of the COB varieties may have only had seed of varieties that were not particularly suitable, while in other districts the opposite may have occurred.

Unequal access to seed would apply even more when this is considered at a village level. For example, in 12 of the 36 villages COB varieties were not grown at all, while in some others over 70% of the households grew them (Fig. 12). The most likely cause is lack of access to seed given the very small quantities of seed that were distributed at a district level compared with the rice area (Appendix 1). If this access were to improve then the use of COB varieties could increase in villages where use is currently low. The alternative explanation - that there is an agro-ecological reason to explain the low use in particular villages - is less convincing as all villages have some upland, medium and lowland and various combinations of the eight COB varieties can be grown in all of these three rice domains. hence, it could be a contributory factor but is unlikely to be the sole explanation.

Table 7. Total households (HH) identified in the sample villages and the proportions of them that grew PVS and COB varieties in at least one of the two seasons i.e. *Chaite* 2008 or the main season 2007, from the group discussions in 2008.

District	Region	Level intervention	HH in 6 sampled villages in each district (total HH)	Total users in GD (% total HH)	COB user† in GD	PVS user† in GD	User of both COB & PVS† in GD
					(% of user HH)		
Kanchanpur	West	High	265	34	65	50	15
Banke	West	Low	245	33	30	82	12
Nawalparasi	Centre	High	262	75	27	75	3

Chitwan	Centre	High	406	38	39	68	8
Rautahat	East	Low	668	57	4	98	4
Morang	East	High	376	20	20	95	15
Total			2222	44	23	82	7

†The COB users may also be growing a PVS variety and *vice versa*. Hence, ‘COB user’ + ‘PVS user’ - ‘User of both’ = 100%

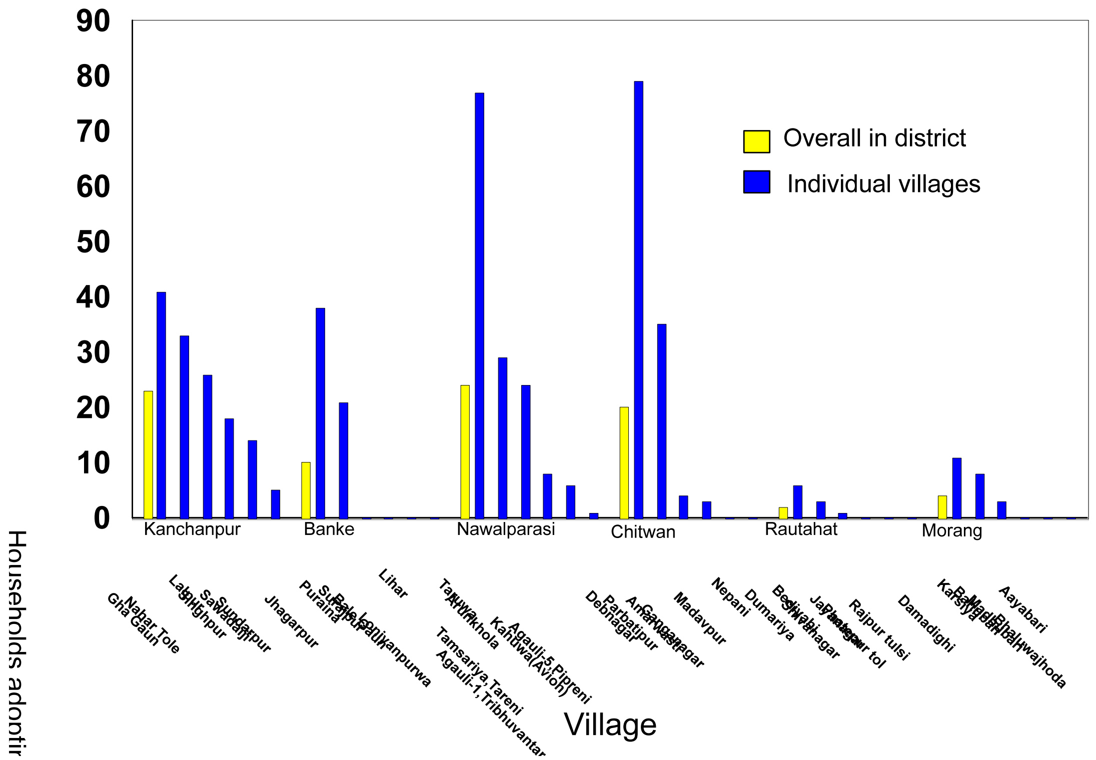

Fig. 12. Use of COB varieties (% of all households identified in group discussions) by village across six districts, from group discussions in 2008.

Household survey results – users of COB and PVS varieties

We interviewed a random sample of households identified as COB/PVS users in the group discussion and 39% of them were growing a COB variety (Table 8). This was much higher than the comparable figure of 23% in the group discussions (Table 8). The estimate from the household survey was considered more reliable as in the GD key informants supplied information for households other than their own, while in the household survey householders reported only on their own experiences. Key informants in the GD in three of the districts (Nawalparasi, Rautahat and Morang) underestimated the number of users of the more-recent and infrequently grown COB varieties; while in the remainder they did give information that was in good agreement with the household survey results. There was no relationship between the number of people taking part in each group discussion (Table 4) and the accuracy of the group discussion. The extent of agreement between a group discussion and the household survey will largely depend on how well-informed the members of the particular groups were.

The proportion of the users growing PVS varieties was high whatever the survey method (75% of users from the GD and 80% of users from the household survey).

Table 8. Estimates of COB users from the group discussion and the household survey.

District	COB users in the GD (% of users of COB and PVS in the GD)	COB users in the household survey (% of users of COB and PVS in the GD)
Kanchanpur	66	68
Banke	30	28
Nawalparasi	27	65
Chitwan	40	36
Rautahat	3	12
Morang	19	31
Total	23	39

The areas on which the COB varieties are grown

The area on which the varieties were grown was small and averaged about 0.17 ha per household per variety (Table 9). This could not be explained by land availability as there was no correlation at all between the area a household devoted to COB varieties and the area of rice that a household cultivated.

For some varieties, grown in particular situations, such as Barkhe 1027 in the medium land and Judi 572 in the upland, areas were very small. In these cases, the varieties were not being grown in their optimal ecosystem and may simply have been under experimentation by new users. Apart from these two exceptions, varieties were grown on an average of at least 0.1 ha. Barkhe 3004 had the highest average area of any variety (more than one third of a hectare) and accounted for 27% of the total rice area of the households that grew this variety (Table 10). The average area of cultivated rice land of the COB users was about 1.2 ha so 12.5% of the land of the users was devoted to individual new varieties. The overall proportion of land devoted to COB varieties among the users was somewhat higher, at 15%, as 17% of households grew two or more COB varieties[2]. There are two possible explanations for this small proportion, the first of which is the most likely:

- The low areas may partly be because this is an early stage in the innovation process so the use of the COB varieties could be limited by seed availability as well as a desire by some farmers to try the variety for more years before taking the risk of growing it on a larger area. However, no meaningful test of this was possible as many farmers were not able to say when they first got access to seed and the sample size for earlier years was very low because at that time only small quantities of seed were distributed.
- That all of these varieties (with the possible exception of Barkhe 3004) are niche varieties that will be grown by many farmers but on relatively small proportions of total rice land. However, the breeding programme was not targeted at producing niche varieties and the wide use of some of the varieties across districts would make this seem unlikely.

[2] Of the 136 COB users in the household survey, 22 grew two COB varieties and one household grew three.

Table 9. The average areas in which eight COB and BG 1442 were grown according to land type in the main season of 2008, from a household survey in 2008.

COB or PVS variety	Cases in upland (no.)	Mean upland area (ha)	Cases in medium land (no.)	Mean medium land area (ha)	Cases in lowland (no.)	Mean lowland area (ha)	Mean area (ha)
COB							
Barkhe 1027	11	0.16	14	0.07			.13
Barkhe 2001			11	0.19			.19
Barkhe 2014			16	0.18			.18
Barkhe 2024			6	0.18			.18
Barkhe 3004			17	0.33	4	0.23	.34
Judi 572	2	0.03	18	0.13			.13
Sugandha 1			8	0.10			.1
Sunaulo Sugandha			53	0.10	6	0.12	.1
Average for COB		0.14		0.15		0.16	.17
PVS							
BG 1442	36	0.18	72	0.15	3	0.21	.17

Table 10. The average areas in which eight COB and BG 1442 were grown by households as a proportion of the total rice land cultivated by them in the main season of 2008, from a household survey in 2008.

COB or PVS variety	Total cases (no.)	Mean COB area† (ha)	Mean total rice area (ha)	COB area (% total rice area)
COB				
Barkhe 1027	22	.13	0.9	7.5
Barkhe 2001	11	.19	1.2	15
Barkhe 2014	16	.18	1.7	11
Barkhe 2024	6	.18	0.9	19
Barkhe 3004	19	.34	1.2	27
Judi 572	19	.13	1.3	10
Sugandha 1	8	.1	0.8	13
Sunaulo Sugandha	59	.1	1.2	9
Average for COB	136	.17	1.2	12.5
PVS				
BG 1442	163††	.15	1.2	12

†From last column of Table 9.
††Includes BG 1442 users in the *Chaite* season for mean total rice area.

Use of individual COB varieties

Over all six districts, eight COB varieties were found to be grown by at least 1% of all 2,222 households in the group discussions (Table 17). Three other COB varieties were used by less than 1% of households and were excluded from the analysis to reduce its complexity. The early adoption of eight varieties provides strong support that the breeding methods that were used, which were highly simplified and cheaper than conventional ones, could still produce successful varieties (see Box 1).

Box 1. Using only a few crosses.
The RNRRS project made three crosses (see Introduction) that have had time to produce varieties that could have been adopted by the time of the group discussions and household surveys in 2008. Varieties Barkhe 2001, Barkhe 2024, Sugandha 1 and Sunaulo Sugandha were all from the irradiated, out-crossed Pusa Basmati 1 population, while Barkhe 1027, Barkhe 2014 and Barkhe 3004 were derived from the cross Kalinga III/IR64. Judi 572 was the only variety from the third cross, Kalinga III/Radha 32, that was grown in the main season but Judi 582, also from this cross, was found in a very low frequency in the *Chaite* season in the six study districts and is also adopted in Bangladesh. Hence, a significant change in breeding method employed in the COB programme, i.e., using few crosses, has been demonstrated to be effective.

Sunaulo Sugandha, one of the two released COB varieties was the most widely grown among all of the varieties (Table 11). Barkhe 3004, the other released variety, did not have the higher use that might be expected from its official release and greater promotion (more seed of it had been supplied than of other varieties). It was about as widely grown as three unreleased varieties i.e., Barkhe 1027, Judi 572 and Barkhe 2014. One contributory factor is that it had been most widely promoted in Chitwan district but did not replace Masuli there.

Table 11. Proportion of farmers who grew a COB variety in the main season among all of the 2,222 households in six terai districts, from the group discussions and the household survey.

Variety	Mean use (% users)†
Barkhe 1027	2.8
Barkhe 2001	1.4
Barkhe 2014	2.0
Barkhe 2024	0.8
Barkhe 3004	2.4
Judi 572	2.4
Sugandha 1	1.0
Sunaulo Sugandha	7.5
COB user	17.4

†% of all 2,222 households derived as: COB users in household survey * % all users in the GD. For full explanation of this calculation see Box 2).

The scoping study on varietal diversity showed that no variety from whatever source or type (Nepal or India, released or unreleased) that was currently grown by farmers was found across all of the terai districts (Fig. 13, 14, 15). However, at this early stage in the innovation process, both Barkhe 3004 and Barkhe 2014 were grown in both the most westerly and easterly of the sampled districts (Fig. 16).

There were big differences in the use of individual COB varieties between districts (Fig. 16) that were reflected in even larger differences between villages. In four districts Sunaulo Sugandha was the predominant variety (Fig. 16); but Judi 572 was most used in Banke, and Barkhe 2014 and Barkhe 2001 were (equally) the most used in Morang. The districts also differed in the diversity of COB varieties that were used, which ranged from 6 in Kanchanpur to 2 in both Banke and Rautahat. The

limited amounts of seed supplied (Appendix 1) is an important factor in determining the use of the COB varieties as, clearly, farmers cannot choose to use a variety if they have not had access to seed.

The COB programme has produced eight varieties of differing adaptations, quality and maturity that are being used. At least three varieties, Barkhe 1027, Judi 572 and Barkhe 2014, have not yet been considered in the release system but have sufficient use to confirm their acceptability to farmers and justify their official release, particularly if it is targeted towards particular terai districts.

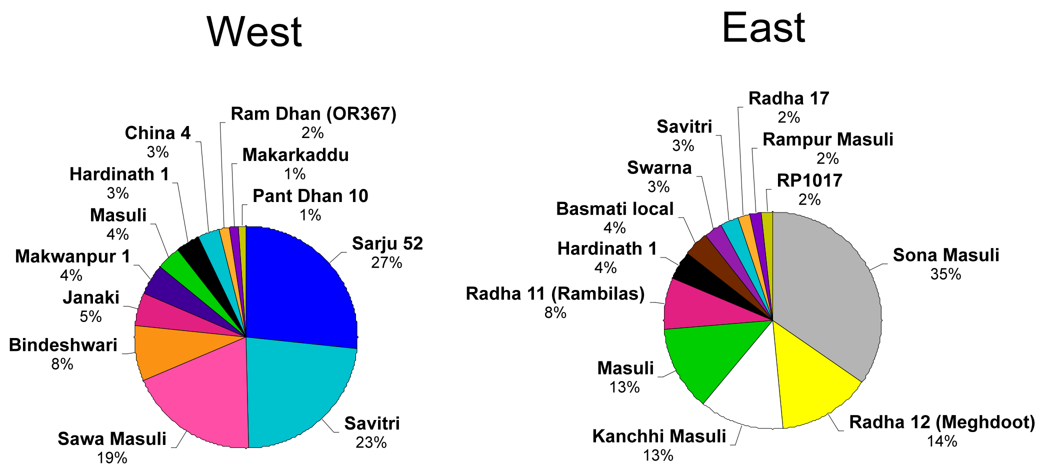

Fig. 13. Differences in varietal composition in western (west of Parsa) and eastern districts (east of Makwanpur) of Nepal from the scoping study. Only three varieties are found in both regions, Masuli, Savitri and Hardinath 1 (BG 1442).

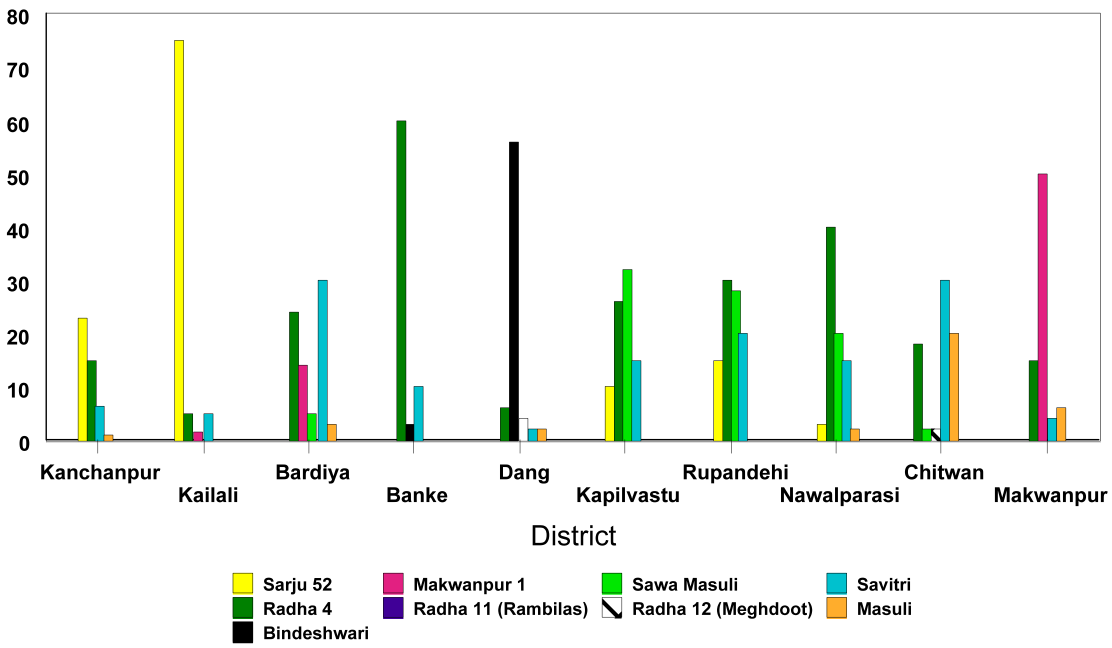

Fig. 14. The most widely grown varieties in the western region of Nepal by district, from the scoping study.

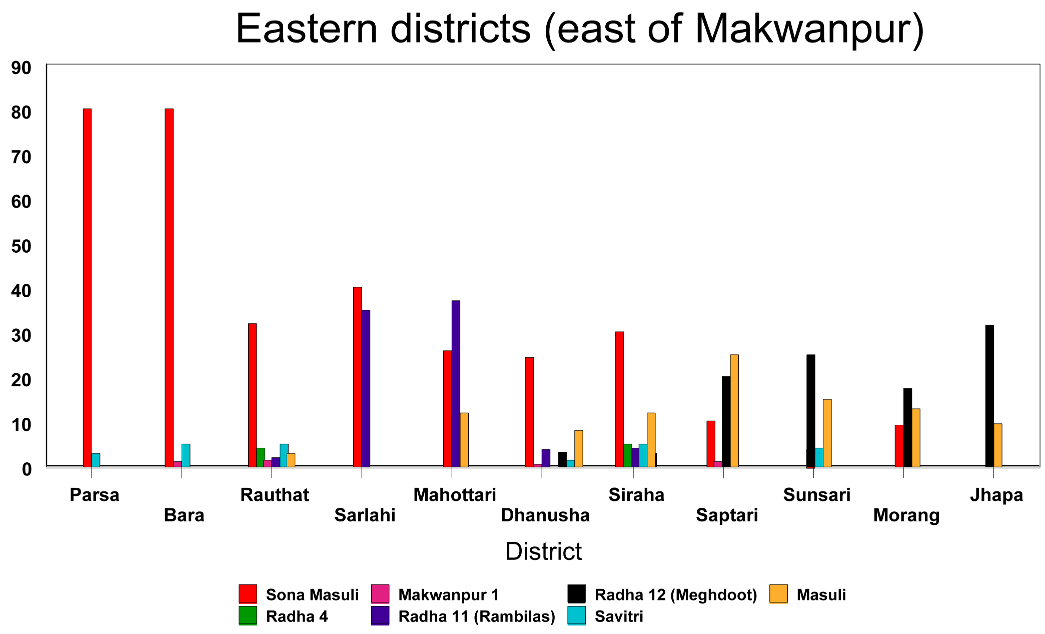

Fig. 15. The most widely grown varieties in the eastern region of Nepal by district, from the scoping study.

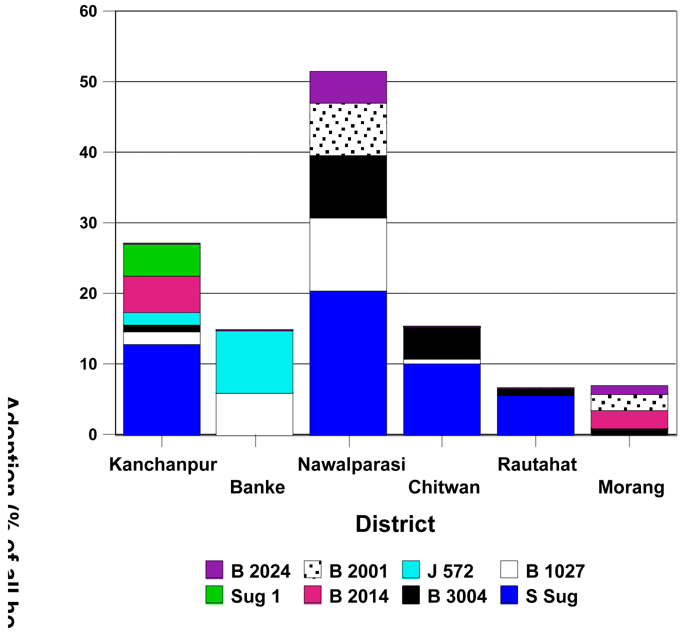

Fig 16. Use of the COB varieties across six terai districts, from the scoping study in 2008.

The scoping study identified more COB varieties as it was done for all of the terai districts and not just six. It identified Barkhe 3004 as the most widely grown COB variety (Fig. 17a). However, the scoping study identified a similar number of COB varieties in the six districts although there was very poor agreement across the two methods (Fig. 17b). Since the use of individual COB varieties varied greatly across villages and only six were sampled per district good agreement is unlikely between the two methods; In the scoping study, expert opinion was based on the entire district. However, the DADO officers seemed not to have kept up with the rapidly changing situation: they were not always aware that the use of new, non-released COB varieties, such as Barkhe 1027, had increased quickly.

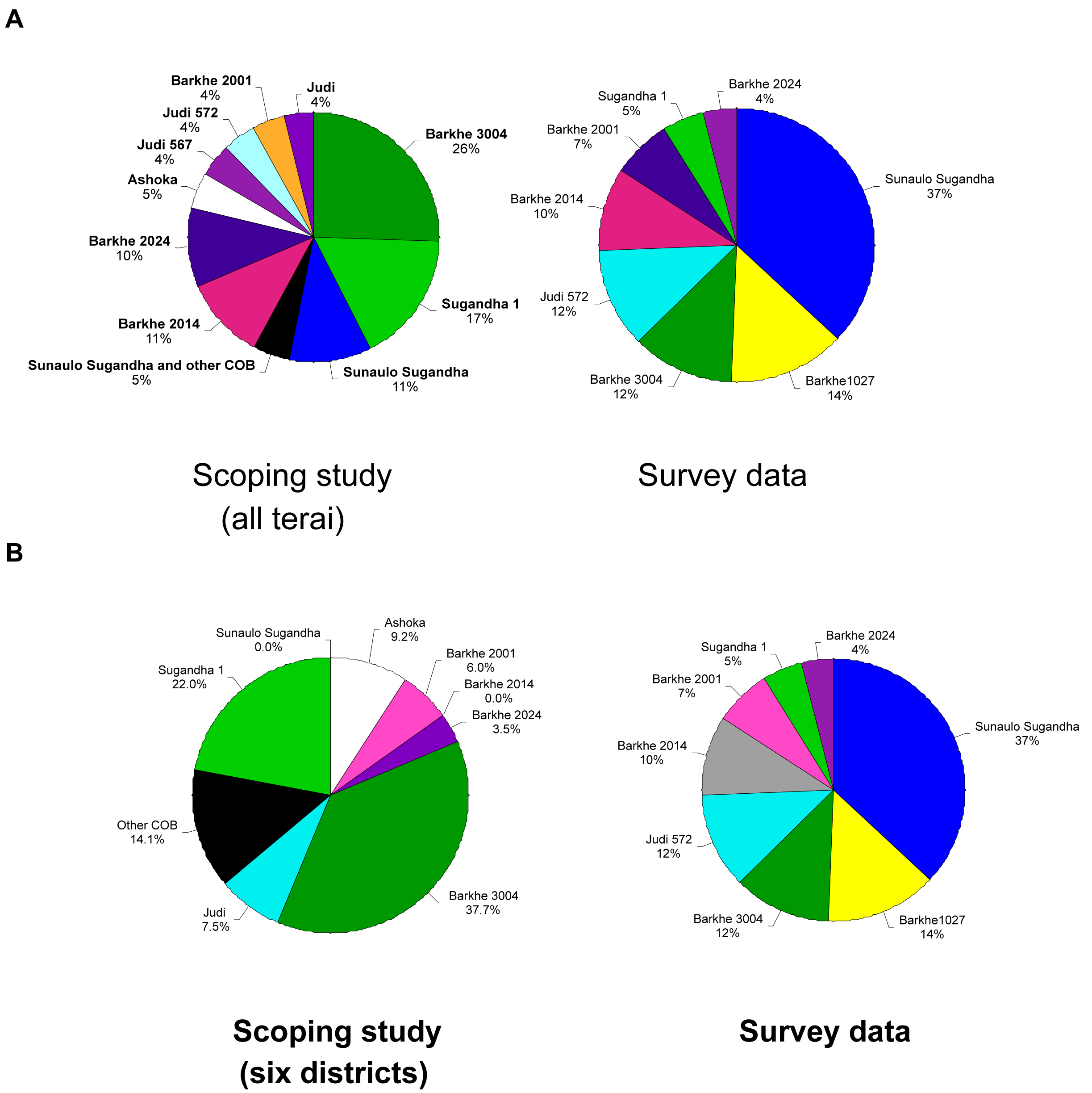

Fig. 17a and b. A comparison of the frequency of the COB varieties identified in the scoping study either in all terai districts (17a) or in the six terai districts in the household survey (17b) with the household survey in six terai districts, 2008.

Level of use of the COB varieties compared with that of the PVS varieties

The amount of use of the COB varieties was compared with that of the PVS varieties. None of the COB varieties was adopted as much as the most widely adopted PVS variety BG 1442 - released as Hardinath-1, which was grown in the main season by 14% of those household surveyed (Table 12). This compared with an overall use of 17% for all of the COB varieties and a 7.5 % use of Sunaulo Sugandha, individually the most widely grown of the COB varieties (Table 13). The higher use of BG 1442 is to be expected, as more seed of it has been distributed and it has been in the system far longer than any of the COB varieties: it was introduced in the 1980s and released five years ago.

Table 12. Main season use by household of three named PVS varieties†, all COB varieties, and combined use of PVS and COB as a proportion (%) of the 2,222 households identified in the group discussions. For method of calculation see Box 2.

Main season use by household	Kanch-Anpur (%)	Banke (%)	Nawal-Parasi (%)	Chitwan (%)	Rautahat (%)	Morang (%)	Mean use (% 2,222 house holds)
Users of any three PVS varieties*	13	0	9	19	51	1	15
BG1442	9	0	9	18	51	1	14
Pant 10	3	0	0	0	0	0	1
PR 101	1	0	0	4	0	0	1
COB users	23	9	49	14	7	6	17
User of PVS and/or PVS	29	9	50	30	52	7	29

†The PVS varieties Swarna and Rampur Masuli were excluded because of problems of attribution as they are grown for reasons other than just interventions in the RNRRS projects.
*This does not equal the sum of the three PVS varieties in Chitwan as some farmers grew more than one.

Seed spread

The source of seed for the COB varieties

In many cases information on the source of seed could not be obtained from farmers. However, for those that responded there was clear evidence of either farmer to farmer spread or market distribution or both of these for four varieties: Barkhe 1027, Barkhe 2001, Judi 572 and Sunaulo Sugandha (Table 13). The numbers of users of these varieties are likely to continue to increase (although at a much lower rate) in the rice innovation system, even if there were no further support from NGOs, as a result of farmer-to-farmer and market spread. Overall, in about one third of the cases the seed was from a source that was independent of an NGO or government intervention i.e. it was from a market or friend/neighbour.

Table 13. The different sources used to grow COB varieties as the number of cases reported and the overall percentage of each source, from the household survey that included 136 main season COB users.

	Number of cases							
Variety	LI-BIRD	SUPPORT	FORWARD	Other NGO	DADO	Market	Friend/ Neighbour	Total
Barkhe 1027	8	3				11		22
Barkhe 2001	3			1			5	9
Barkhe 2014		3	1	1	1		5	11
Barkhe 2024	3							3
Barkhe 3004	12	1		3				16
Judi 572		1	3			10	3	17
Sugandha 1		5					3	8
Sunaulo Sugandha	29	12					8	49
Total number	55	25	4	5	1	21	24	135
Overall (%)	41	19	3	4	1	16	18	100

Seed spread from farmers in the household survey

Farmers were supposed to be asked in the household survey for all of the seed transactions of each variety but in practice a maximum of one transaction per farmer per COB variety was recorded (Table 14). The actual number of transactions per variety would have averaged more than one among the total of 160 users because in the Nepal terai farmers frequently distribute the seed of the same rice variety to several other farmers from a single harvest (Table 15 and Witcombe et al., 2001).

From the 2007 harvest, about a fifth of the household surveyed farmers who were growing a COB variety distributed seed to another farmer (Table 14). There were very large differences between individual varieties but they correlated poorly with seed source the COB growers had used. For example 45% of Barkhe 2014 users got seed from friends and neighbours (Table 13) but only 19% of growers distributed seed (Table 14) whereas no Barkhe 3004 user had got seed from friends and neighbours but 42% had distributed seed. Such discrepancies are perhaps to be expected with the small sample sizes.

It is unclear whether all of the reported seed distribution was in fact for sowing or whether it was sometimes grain because the amount per transaction was high. It averaged 230 kg per transaction when one very large sale of Barkhe 3004 was excluded (and increased to 515 kg when included). Nonetheless, half of the transactions were reported by the household surveyed households to be through exchange or gift so the majority of the transactions can safely be assumed to be of seed rather than grain.

The seed was distributed over reasonably large distances. The average distance between the locations of the recipient and the distributing household was 1.2 km and the maximum distance was 50 km. Of all the transactions, 58% were at a distance of over 1 km and hence very likely to have been distribution to outside of the area covered by the household survey.

Table 14. Seed distribution from the 2007 main season harvest by the users of the COB varieties from the household survey in 2008.

Variety	Users of individual variety (no)	Distributed seed from 2007 harvest (no)	Distribution (%)
Barkhe 1027	22	2	9
Barkhe 2001	11	5	45
Barkhe 2014	16	3	19
Barkhe 2024	6	0	0
Barkhe 3004	19	8	42
Judi 572	19	1	5
Sugandha 1	8	5	62
Sunaulo Sugandha	59	9	15
Overall	160†	34	21

†160 cases of individual varieties grown by 136 users i.e., 113 users of one COB variety, 22 users of 2 COB varieties, and 1 user of 3 COB varieties.

Seed spread of COB variety Barkhe 2014 from village to village

To more precisely target the spread of seed from village to villages an additional group discussion was held in Malhanama village in Saptari district in May 2009 by staff from FORWARD on the distribution of seed of Barkhe 2014 by farmers. Farmers were asked for all of the transactions relating to Barkhe 2014 (not just a maximum of one) and they were also asked which villages the recipients were from. There were 25 key informants present and they reported on a total of 50 farmers who were growing rice. Of these 47 were growing COB variety Barkhe 2014 and 9 of them were present in the group and 38 were absent.

Overall the seed distribution rate from a single harvest was high (Table 15) but those that were speaking for themselves reported a higher level (two thirds of them

distributed seed) than when they reported for others (29% were said to have distributed seed). The number of recipient farmers per distributor and the amount of seed was also higher for those that were present in the group discussion. The data for those present has to be considered more reliable, but, whatever category is considered, the seed distribution of this COB variety from a single harvest was significant.

Table 15. Distribution from the 2008 harvest of seed of Barkhe 2014 from Malhamana village, Saptari according to whether the farmers were present in the group discussion or not.

Parameter	Farmers present in GD reporting for themselves	Farmers not present whose seed distribution was described by those present in GD
Farmers (total no)	9	41
Farmers that distributed seed (no)	6	12
(%)	67	29
Recipients per distributing farmer (no)	2.5	1.8
Total transactions (number)	15	21
Average amount of seed per recipient farmer (kg)	76	21

The total of 18 farmers that distributed seed (whether present or not in the group discussion) did so to farmers in thirteen new villages (Table 16), indicating a very high spread from village to village. One village, Malhaniya, that was 10 km from Malhamana, had the greatest number of recipients and indicates how farmers in a secondary village can quickly and widely use a new variety without any project intervention. On average, villages were situated 16 km away from Malhanama and the furthermost was 45 km distant. One village was located in an adjoining district to Saptari.

Table 16. Distribution of seed by 18 farmers from Malhamana village, Saptari, by village, and the distance of the village from Malhanama.

Village name	Number of times there was a recipient of seed in the village	Distance of recipient's village from Malhamana (km)
Arnaha	1	40
Haripur	1	4
Kalyanpur	1	10
Kamalpur	1	12
Khadkapur	1	18
Kuruwa	1	23
Mahuliya	1	11
Malhaniya	9	10
Pansera	1	2
Pipra West	3	3
Praswani	1	45
Saraswor	2	1
Sukhipur†	1	33
Total transactions to villages outside of Malhanama and average distance of 13 recipient villages from it	24	16
Within-village transactions	12	0.6

†Outside of Saptari District, in Siraha

Farmers' awareness of the COB varieties

In the rice innovation system the extent of spread of information from farmer-to-farmer about new varieties has to be an indicator of their future use – unless farmers find out about a variety they will be unable to grow it and when more farmers that have heard of a variety more are likely to try it. Both users – those that were growing at least one COB or PVS variety – and non-users were asked about their knowledge of the varieties and their intentions concerning those they had heard of.

Farmers that had already grown a PVS or COB variety were a little more aware of the new varieties - 20% had heard of them compared with 16% among non-users (Table 17). Moreover, there was greater willingness to try them among the users - of the 20% of the users who had heard of a new variety nearly half intended to try it, whereas of the 16% of the non-users who were aware of a new variety only one quarter intended to do so. It appears likely that if a farmer had tried a COB variety he or she was more willing to try another – perhaps because, in most cases, they had liked the COB variety they tried.

Banke and Rautahat were low intervention districts and this is reflected in the low level of awareness among the farmers of the new varieties. The greatest awareness was in Chitwan and Nawalparasi where the project had commenced in 1997.

An analysis of the awareness of individual varieties was made only for the 344 users as the total sample size was larger than for the non-users (Table 18). The eight COB varieties differed in the extent to which farmers were aware of them (from 7% for Judi 572 to 44% for Sunaulo Sugandha). The two released varieties, Sunaulo Sugandha and Barkhe 3004 were the most well known of the COB varieties and, perhaps because they are officially recommended, a higher proportion of farmers who had heard of them intended to try them. More farmers were aware of Sunaulo Sugandha and willing to try it than was the case for Barkhe 3004 and overall one third of all the interviewed farmers who did not currently grow it said they intended to try it (Table 18). This reflects the unique characteristics of Sunaulo Sugandha - an unusual combination of high yield and aromatic grains. Sugandha 1, another aromatic variety, was also well known (27%) although the proportion of farmers who intended to try it was much lower than for Sunaulo Sugandha.

There was awareness of the COB varieties among households that would like to try them showing that information travels faster than seed. The now-released PVS variety, BG 1442, that had been first tested many more years before any of the COB varieties, had become better known than any of the newer COB varieties and had a large proportion of farmers who intended to try it (Table 18).

Table 17. Proportion of farmers who had heard about COB varieties that they were not growing and their intentions. Average responses for the eight COB varieties in a household survey.

	District (% of responses)						
	**Kanchanpur**	**Banke**	**Nawalparasi**	**Chitwan**	**Rautahat**	**Morang**	**Overall**
User of COB/PVS variety†							
Heard of COB var. they are not growing	26	2	40	37	5	14	20
Heard, won't grow	12	1	25	19	0	4	10
Heard, intend to try	11	2	13	11	4	6	8
Grew in past, will grow again	3	0	1	6	1	4	2
Grew in past, won't grow again	0	0	1	2	0	0	<1
Non-user of COB/PVS variety							
Heard of COB variety	21	0	33	43	4	9	17
Heard, won't grow	14	0	23	36	2	8	12
Heard, intend to try	7	0	10	7	3	1	4
Grew in past, will grow again	0	0	0	0	0	0	0
Grew in past, won't grow again	0	0	0	0	0	0	0

†In ether main or *Chaite* season (n=344).

Table 18. Proportion of farmers among the 344 users that knew about a named COB variety they were not growing and their intention to grow the variety if they had heard of it. Responses for eight COB varieties and PVS variety BG 1442 from the household survey.

**Variety**	**Heard of the variety (%)**	**Intend to try (% of those that had heard of it)**	**Intend to try (% of all 344 users)**	**Grew in past, will grow again (% of those that had heard of it)**	**Grew in past, won't grow again (% of those that had heard of it)**
Barkhe 1027	9	22	2		
Barkhe 2001	16	25	4	2	1
Barkhe 2014	21	10	2	5	
Barkhe 2024	15	20	3	2	0
Barkhe 3004	28	43	12	2	0
Judi 572	7	29	2	0	1
Sugandha 1	27	26	7	1	0
Sunaulo Sugandha	44	75	33	6	1
Overall for COB variety	20	38	8	11	2
BG 1442 (among users)	89	38	33	37	12
BG 1442 (among non-users)	61	70			

Estimated extent of Use of the COB and PVS Varieties Outside of the Household surveyed Villages

The use in the household surveyed villages was compared with the seed distributed at a district level (Table 19).

Although Nawalparasi had the highest use it was not the district with the highest intervention. The extent of use between districts was determined by many factors some of which were unknown and some of which could not be measured in the household survey. The extent of interventions in the individual villages was not known as the RNRRS research projects did not have resources to measure the levels of development activities at a village level. Factors that could not be unravelled in the structured questionnaire were the large number of factors that would form the basis of each individual decision by a farmer to use or not use a particular variety. This decision not only depends on the overall advantages of each of the eight different COB varieties (and this was determined) but how it varied from household to household depending on the different alternative varieties that were available to each farmer in each district and village and the particular circumstances of each farmer.

In Chitwan the use was low compared with seed supply. In this district the majority of seed supplied was of Barkhe 3004 and farmers were found to be reluctant to grow it in place of the widely grown variety Masuli (see Introduction) that attracts a premium price for its good eating quality and distinctive glume colour. Masuli now occupies about 20% of the area in Chitwan but in some parts of the district with particularly suitable land for the variety it is much more extensively used.

Table 19. Summary of use by district, using extrapolation from the samples in the group discussions and the household survey of 2008 in 36 villages in the six districts.

District	Users in the GD of at least one COB or PVS variety (% households)	COB users in main season in the household survey (% of users in GD)	Estimated COB users in all of the 2,222 households (% of all households)†	Rice area devoted to COB varieties in household surveys (% area)†	Seed of COB rice varieties distributed In district (t)	Seed of COB rice varieties distributed (ha equiv)†
Kanchanpur	34	68	23	3.4	8.7	170
Banke	33	28	9	1.3	1.0	20
Nawalparasi	75	65	49	7.3	4.5	90
Chitwan	38	36	14	2.1	34.0	680
Rautahat	57	12	7	1.0	1.3	30
Morang	20	31	6	0.8	4.7	90
Overall	44	39	17	2.5	54.2	1080

† Extrapolated from the GD and household survey (see example on how this is estimated for the overall values in Box 2).

To obtain a possible upper limit of COB use, the proportions of the area in the household surveyed villages in the six districts were extrapolated to the district level (Box 3). These upper limits were 2.4% of the area in the six districts, amounting to 6,700 ha (Table 20). Overall, 17% of the 2,222 households would grow a COB variety with a high of 49% of households in Nawalparasi and a low of 6% in Morang (Table 19).

Table 20. Summary of use by district, using extrapolation from the samples in the group discussions and the household survey of 2008 in 36 villages in the six districts.

District	Rice area devoted to COB varieties in household surveys (% area)†	Area of rice in district (ha)	Extrapolated area under COB varieties in district (ha) †
Kanchanpur	3.4	44370	1500
Banke	1.3	31840	400
Nawalparasi	7.3	42650	3100
Chitwan	2.1	33690	700
Rautahat	1.0	48950	500
Morang	0.8	95050	800
Overall	2.5	296550	7400

† Extrapolated from the GD and household survey (see example in Box 2)

Box 2. Extrapolating use in the six districts from the group discussions and the household survey data

The best overall estimate of the use of COB varieties in the whole of the 2,222 households identified in the group discussions can be obtained using a combination of the group discussion and the household survey data. The latter is considered more reliable as each sampled household reported on their own experiences.

Group discussion determined that 44% of households (i.e. 1,022) were users of any one of the PVS or COB varieties

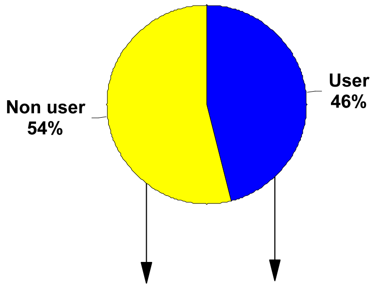

139 non adopter households randomly sampled

344 households from the 44% adopters randomly sampled

39% of them adopted a COB variety

Best estimate of overall use of COB in 2,222 households
= 39% x 44% i.e. 17%
If these 17% of households use them on 15% of their rice land then COB varieties are used on 2.5% of all the rice land

The assumptions relating to the confidence with which this extrapolation can be made to the district level are discussed below. Only the first would lead to an underestimate.

Users were defined in the group discussions for the main season of 2007, whereas, the number of users would have increased by 2008.[3]

The sampling of the villages within districts was purposive in that villages where activities had taken place were selectively sampled. If high intervention always produces high use and vice versa then this would result in a high degree of bias. However, interventions were small and much of the adoption was because of the spread from farmer to farmer.

- There was spread of seed of COB varieties from farmer to farmer within villages and purchase of seed of them from the market (Table 13).
- In district Saptari, there was a thirteen fold village-to-village spread of Barkhe 2014 from a single harvest.
- The awareness and intention to try the new COB varieties was also spreading, particularly for Sunaulo Sugandha and Barkhe 3004.

That the bias of using purposive samples is not very high is supported by the following evidence:

- Previous studies in districts Kailali and Sarlahi had shown that some of the villages known to be non-intervention had higher levels of COB use than those where the interventions had taken place. COB varieties were grown, in the two years for which estimates were made, 2005 and 2007, on 3.3 to 5.5% of the area.
- Studies using random transects in all of Chitwan (the position of which were determined using the global positioning system) showed that adoption in the whole of Chitwan was 1 to 2% of the rice area in 2005 and 2006 (Joshi et al., 2007), compared with 2% in the sampled villages in Chitwan for 2008 (Table 20).
- In the rainfed rabi cropping impact study (part of the same set as this one) the purposive sampling was less biased towards activities relating to COB varieties of main season rice because the major technologies being studied were associated with rabi (winter) crops. In this study in four districts - Jhapa, Kapilbastu, Saptari and Siraha - on average 10% of all households identified in the group discussions used a COB variety (Table 21), which is 40% lower than the 17% of the households that grew a COB variety in the six districts of this study. Use rates varied substantially between districts - from about 1% in Siraha and Jhapa to 4% in Kapilbastu, and a high rate of about one third of households in Saptari who mainly grew Barkhe 2014 .

[3] Any error from non-users being users (and cases of this were found) would be counterbalanced by users who, on interview, were found to be non-users (4% of the cases).

Table 21. Adoption of COB varieties in a study of rainfed rabi cropping technologies in four districts (source Harris et al., 2009).

	Jhapa	Kapilvastu	Saptari	Siraha	Grand Total
COB users in household survey (no)	1	10	50	1	62
Households surveyed (no)	83	73	80	51	287
COB users in household surveys (%)	1.2	13.7	62.5	2.0	21.6
Users of any rainfed technology in group discussion (%)	83.8	29.4	51.0	27.6	46.8
COB users (% households in group discussion)†	1.0	4.0	31.9	0.5	10.1

†This is obtained following the general method in Box 2: Overall COB users = COB users (%) in household survey * all users (%) in group discussion i.e. for Jhapa is 1.2% * 83.8% = 1.0%.

Overall the scoping study was in good agreement with the household survey in that the total area in the six districts estimated by the key informants from the DADOs was also about 6,700 ha but, as might be expected from the big differences in the methods, the deviation district-by-district was very high (Table 22).

Table 22. Comparison of the group discussion and household survey with the scoping study.

	Rice area devoted to COB varieties in district	
District	Group discussion and household survey (% area)†	Scoping study (% area)
Kanchanpur	3.4	1.5
Banke	1.3	0
Nawalparasi	7.3	0
Chitwan	2.1	0.5
Rautahat	1.0	2.0
Morang	0.8	5.8
Overall	2.5	2.3

† Extrapolated from the GD and household survey to the entire district (see example on how this is estimated for the overall values in Box 2).

The overall use is conservatively estimated to be half (after rounding down) of that found in the purposive sample, i.e., 1.2% instead of 2.5% and hence approximately equal to that found in the scoping study (1.4%) (Box 3).

Box 3. Overall use of the COB varieties

About 1.1 M ha of rice land is cultivated in the terai and using the two estimates of use (1.2% and 1.4% of the rice area) about 15,000 ha was estimated to be under COB varieties and grown by about 10% of households (75,000 to 100,000 households) in the main season of 2008.

It is a reasonable assumption that by 2010 or 2011 a much higher proportion than the estimated minimum of 1.2% would be devoted to COB varieties, given: the evidence of high farmer to farmer dissemination, recent efforts in popularisation, levels of farmer awareness, and increased availability of seed in the market. During the period 2004-2008, when seed availability was extremely limited, use had grown from negligible (only a little above zero) to over 1%. Further increase from 2008 to 2010 or 2011 should follow that of a typical S-shaped adoption curve (Rogers, 1962), in which case an additional 2% in this period would be highly conservative given the properties of an S-shaped curve. Hence a reasonable range is 2 to 4% and by this time it is safe to assume that (a) overall areas of COB varieties could increase to 45000 to 75000 ha and (b) the varieties would be grown by a very large number of households (in a range from 150,000 to 500,000 households).

If PVS varieties were included, the area under rice varieties from the RNRRS projects would approximately double as PVS varieties were estimated to be grown by 15% of the sampled households compared with 17% for COB.

The extent of use of the COB varieties has to be considered in the context of the recentness of the RNRRS intervention and the resources spent on the dissemination of the varieties. The breeding programme commenced in 1997 and this impact assessment was made in 2008. After 11 years a conventional breeding programme would not have even resulted in any dissemination of new varieties to farmers. The first significant quantities of seed of the COB varieties were distributed in 2002 but were still extremely small in relation to the total rice area in the terai (Fig. 19).

This can be compared with the age of the varieties from the national programme determined in the scoping study (Fig. 20). Variety Savitri is one of the varieties in the group of varieties released more than 20 years ago. It was released in1979 and 16 years later when the baseline study was done in 1997 it was not widely grown (Fig. 7). Only in the recent past, many years later, has it been widely used by farmers who increasingly found that the extra labour to thresh it gave more returns over competing varieties that were yielding less grain as they became more disease susceptible.

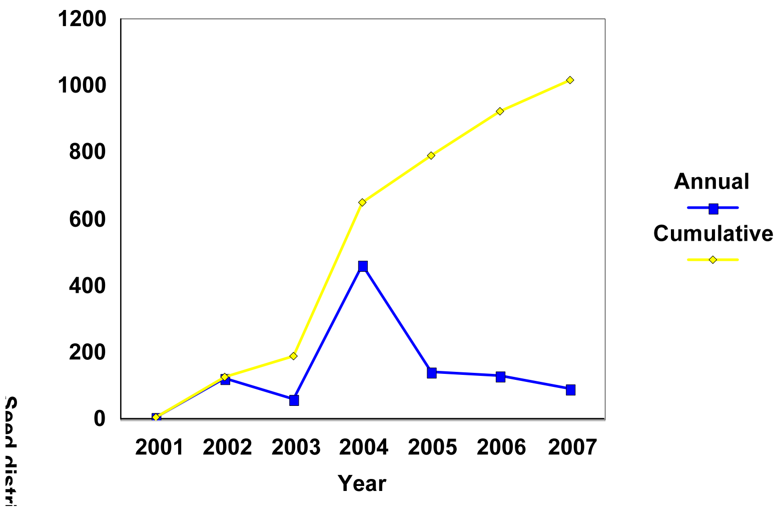

Fig. 19. Amount of seed of COB varieties distributed in the six study districts shown in area the seed could sow assuming a seed rate of 50 kg ha^{-1}.

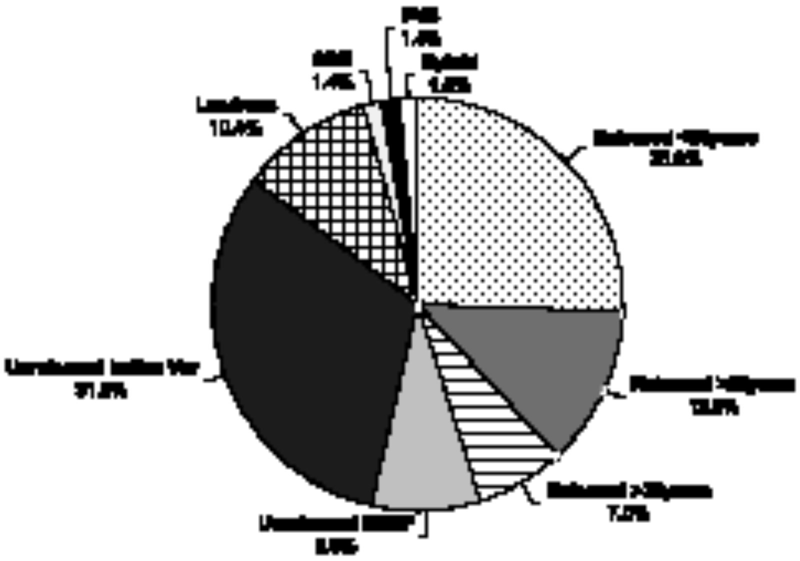

Fig. 20. The composition of varieties in the terai according to age (where known).

Environmental impact

The main anticipated impact on the environment would be expected to relate to agrobiodiversity. Given that many more varieties are used by farmers from the COB programme than would have occurred in the absence of the RNRRS intervention the impact should be largely favourable. However, data have not been collected on the varieties that the COB varieties are replacing so firm conclusions cannot be reached; but prior work (Witcombe et al., 2001) showed that genetic diversity increases from PVS and COB activities.

Other environmental impacts should also be largely favourable as the new varieties produce more grain without requiring additional inputs. There are clear benefits from higher yield per cultivated area as the total land and inputs needed per unit of production are reduced. There may be dis-benefits if some of the varieties, such as Barkhe 1027, that can be grown in the uplands encourage farmers to continue to cultivate uplands that could be converted to perennial plantations crops. However, in

the terai the uplands are still quite favourable agricultural lands that are not subject to high erosion as they are not sloping.

The study did not show that the new varieties were less susceptible to disease even though other studies and the trials data from the COB breeding programme convincingly show this is the case. Increased disease resistance will reduce the use of pesticides.

Benefits to users

Overall benefits

The 344 users were asked about the impact of the new PVS and COB varieties on their livelihoods (Table 23 and 24, Questions 31 to 34 in Annex 1). Farmers who had used any of the COB varieties were asked what the benefits were in the household survey. The question (question 31) was open-ended so whether a benefit was mentioned or not was an indicator of its importance. In addition farmers were asked to give a rank order to each of the benefits they mentioned (with 1 being the most important). Here we use an index of the importance of the benefit derived from these two variables: i.e., the percentage of farmers that mentioned a particular benefit divided by the average rank importance given by farmers to that benefit, which can have a maximum of 100 (all farmers rank the benefit as 1). There were eight benefits that were reasonably important, i.e., had an index of 20 or more, and they varied greatly in their overall importance and among the varieties (Table 23).

Having more income was the most important benefit, followed by the variety having a better taste, and producing more straw. There was a great diversity in the benefits farmers attributed to the varieties. Nevertheless, there were two pairs of varieties with similar benefits:

- Barkhe 2014 and Barkhe 2024 both gave increased income, had better taste and yielded more straw; whereas
- the two aromatic varieties, Sugandha 1 and Sunaulo Sugandha, were perceived to have only two of these benefits (i.e. better taste and higher straw yield).

Although aromatic rice varieties Sugandha 1 and Sunaulo Sugandha fetch a considerably higher market price and hence can give a greater income farmers did not mention or rank more income as an important benefit. Perhaps most farmers were growing these varieties for home consumption and this is supported by the slightly below average areas devoted to them (Table 19).

Only Barkhe 1027 and Barkhe 3004 were not reported to have better taste. Barkhe 1027 was liked for its earliness and Barkhe 3004 for its high yield (more income) and suitability for lowland.

That Barkhe 2001 was the only variety reported as being less prone to disease was a surprising result as it is not known for being particularly disease resistant – on the contrary it was susceptible to neck blast in Chitwan in a single season (but possibly this disease was not found in the areas where the users were interviewed). It may be that the variety it most often replaces is particularly disease susceptible.

Overall the benefits farmers identified from growing these varieties showed how diverse the varieties were and indicated that they occupy different niches. The ceiling for their combined use could be high - already 19% of the COB users in the household survey grew two COB varieties and, in one case, a household grew three of them.

Table 23. Benefits shown as indexes (% response/average rank) for eight COB varieties and one PVS variety from the household survey

Variety	More income	Increased rice grain availability	Better eating quality	Rabi crops because of earlier maturity	More straw	Less disease attack	Early harvest reduces hunger gap	Easy to sell	Suitable for lowland
Barkhe 1027	27						37	25	
Barkhe 2001	28		48			22			
Barkhe 2014	60		33		24				
Barkhe 2024	50		33		21				
Barkhe 3004	57								27
Judi 572	67	36			26				
Sugandha 1			100		48				
Sunaulo Sugandha			96		37				
BG 14442	53		25	24					

Rice self-sufficiency and rice sales

When households were asked about changes in rice self sufficiency and sales through the use of the COB varieties none of the households who reported a change said there was a decrease while about three quarters (77%) reported an increase. Of those reporting increases there was no significant difference in the proportion saying that only rice self sufficiency had increased compared with those that reported an increase in both rice self sufficiency and seed sales (Table 24). No households reported an increase in sales without an increase in rice self sufficiency. It may be that enumerators assumed that those selling were all self-sufficient; and this could be true for cultural reasons – i.e. the importance attached to consuming your own rice.

- Farmers who reported only an increase in food self sufficiency sold no grain but had an average increase in rice self sufficiency of over 2 months (amounting to an increase of nearly 25%). This, on average, brought them into approximate rice grain self sufficiency.
- On average those farmers that reported an increase in grain sales were better off farmers as they have a grain surplus for sale and had twice as much cultivated rice land (Table 24). Hence, they already had a rice harvest sufficient to last 22 months on average and this increased by 4 months or 18%, on average. Their grain sales increased by about 300 kg – an increase of 12%.

These mean increases in rice self-sufficiency and sales reported by users of the COB and PVS varieties were quite large but agreed with the results of the Mother and Baby trials where the COB varieties often yielded an additional 1 t ha-1. The combined area of the PVS and COB varieties of a household would, in many cases, be about one third of a hectare allowing the reported benefit of an extra 300 kg in grain sales.

The increases in rice self-sufficiency and sales reported varied by district (Table 25) and much of this could be attributed to the differences in variety use across districts.

Farmers were not asked to relate the increases to particular varieties. Interpreting the data is difficult because most farmers who grew a COB variety also grew a PVS variety. As the PVS varieties were more widely used than the COB varieties most of the increases would be due to them and this would be particularly true in a district such as Rautahat where COB use was low. In Rautahat only 12% of the farmers in the household survey were COB users (Table 8) and all of the farmers reporting only an increase in food security were growing the PVS variety BG 1442 in the main season. In Banke more (i.e., 28% see Table 8) farmers used COB varieties and some farmers who grew Barkhe 1027 and Judi 572 and no PVS variety reported a benefit in food security. In Chitwan, those reporting an increase in grain sales were growing either BG 1442 or Sunaulo Sugandha.

However, those who reported no increase in either sales or rice self-sufficiency, were better off than those whose rice self-sufficiency alone increased, but much worse off than those with increased grain sales. One possibility for these differences was differences in use of the range of varieties that were available (Table 26). Those that reported no benefits grew Swarna less frequently.

Table 24. COB and PVS varieties (both main and *Chaite* seasons) and increases in rice self sufficiency and rice grain sales from a household survey of 344 users in 2008.

	Response		
**Responses**	**Both rice self sufficiency and sales increased**	**Only rice self sufficiency inreased**	**Neither have increased**
Number of responses	128	139	77
Response (%)	37	40	22
Present rice self sufficiency (months)††	26	11.8	n.d.†
Prior rice self sufficiency (months)††	22	9.5	n.d.†
Present rice seed sales (kg)	2646	-	n.d.†
Prior rice seed sales (kg)	2361	-	n.d.†
Livestock equiv	4.1	2.2	3.2
Total grain (kg)	8916	3483	4107
Grain per capita (kg)	1114	423	460
Own land (ha)	2.0	0.9	0.8
Cultivated rice land (ha)	1.8	0.9	0.8
Mean of poverty index	1.3	0.7	1.0

†Households who answered no increase were not asked the questions relating to prior and present sales and rice self-sufficiency.
††how long rice would last if none were sold.

Table 25. Impacts on livelihoods of the COB and PVS varieties (both main and *Chaite* seasons) in terms of rice self sufficiency and rice grain sales by district from a household survey of 344 users in 2008.

**District**	**Both rice self sufficiency and sales increased**	**Only rice self sufficiency increased**	**Neither have increased**
Kanchanpur	48	13	38
Banke	18	62	20
Nawalparasi	45	20	35
Chitwan	54	8	37
Rautahat	12	88	0
Morang	48	48	4
Grand Total	37	40	22

Table 26. Use of COB and PVS varieties by the three categories of farmers, from a household survey of 344 users in 2008.

	Use (%)		
	**Both rice self sufficiency and sales increasd**	**Only rice self sufficiency invcreased**	**Neither of these have increased**
Barkhe 1027	4	6	10
Barkhe 2001	2	2	6
Barkhe 2014	5	3	6
Barkhe 2024	2	1	1
Barkhe 3004	7	2	9
Judi 572	2	6	12
Sugandha 1	2	0	6
Sunaulo Sugandha	24	7	23
BG 1442 chaite season	41	26	36
BG 1442 main season	27	40	23
Swarna	21	10	0
Pant 10	2	0	3
Rampur Masuli	14	26	8
PR 101	4	0	4
Sample size	128	139	77

Characteristics of users and non users

Individual characteristics. The household characteristics of the 344 users and the 139 non-users were compared (Table 27). Six household characteristics were used to make up the poverty index and neither of the two people-related indicators that were used showed a significant difference between users and non users. There were significant differences among the two groups for two of the four natural and physical capital indicators (livestock ownership and food grain production per capita) used in the index. There were no differences for roof type or for tractor ownership as there was no ownership in the sampled households.

There was a significant difference between the two groups (Table 27), in terms of the proportion of Dalits (where there were 5% more among the non-users). Nevertheless, disadvantaged groups were highly represented among the users as well with 58% of them either Dalits or Tribal.

On average, about 5% of the households were female headed but 17% of the respondents in the household survey were female as women were more often available for interview than men. The only other socio-economic parameter that had any indication that disadvantaged groups were more commonly found among the users was the slightly higher proportion of female-headed households in the users but this difference was not significant and could actually be an indicator of wealth if the male household head is in full time migratory employment.

Table 27. Socio-economic traits of users and non-users of PVS or COB varieties from a household survey in 2008, with traits contributing to poverty index indicated

	Category			
**Socio-economic indicator**	**User**	**Non-user**	**Overall**	**SED††**
People related indicators				
Full time job holders (mean number)***	0.5	0.5	0.5	0.05
Unskilled seasonal migration (number)***	0.04	0.07	0.05	0.04
Persons migrating (mean number)	0.36	0.40	0.3	0.03
Full time migrants (number)	0.03	0.03	0.03	0.01
Persons migrating (mean months)†	9.4	10.4	9.7	1.4
Female headed households (%)	5.5	4.5	5.2	6.5
Household size (capita)	8.9	8.7	8.8	0.26
Farm workers in household (number)	2.6	2.5	2.6	0.1
Group:				
Dalit (%)	**6**	**11**	**7**	**2.4**
Tribal (%)	52	50	52	5.7
Terain (%)	4	4	4	2.0
Brahmin/Chetri/Newar (%)	38	35	37	5.2
Sample size	344	139	483	
Natural and physical capital related indicators				
Roof type:***				
Thatched roof (%)	26	30	27	7
Tiled/galvanised roof (%)	51	56	52	9
Concrete roof (%)	23	14	20	6
Livestock equivalents (mean of index)***	**3.1**	**2.5**	**3.0**	**0.24**
Tractor ownership***	0	0	0	0
Food grain production per capita (kg/person/year)***	**690**	**580**	**660**	**47**
Total food grain production per household (kg/year)	**5640**	**4580**	**5330**	**374**
Months of grain self sufficiency (no)	18.4	16.9	18.0	0.7
Proportion having own animal for traction (%)	51	52	51	39
Total land (ha)	1.3	1.1	1.2	0.08
Total upland (ha)	0.05	0.04	0.05	0.01
Total medium land (ha)	0.9	0.8	0.9	0.08
Total lowland (ha)	0.3	0.2	0.2	0.04
Sample size	344	139	483	

***Element of poverty index (see Table below)

†Mean months of those migrating.

††Standard error of the difference between the means. Significant ones are indicated in bold font.

Poverty index. A poverty index was calculated (see ‘Methods’) based on the six indicators discussed above. The higher the score (maximum total score 23), the less poor households were. An analysis of variance showed that there were highly significant differences between the districts for the poverty index, *P*<0.001 (Table 28). The households in Rautahat and Banke tended to be poorer than average. The mean total poverty score was also significantly higher (*P* < 0.05) for users than non-users although the absolute difference was not large (Table 28). These relatively small differences were reflected in very similar distributions for the poverty index (Fig. 21) and in both the user and non user categories. No households had a score above 15.

Table 28. Mean Poverty scores of users and non-users of PVS and/or COB varieties, by district.

	Poverty scores						
**District**	**User**	**Non-user**	**Overall**	**SE mean**	**SE difference**	**Significance of difference**	**District rank for HDI**
Banke	5.2	4.7	5.0	0.24	0.34	ns	30
Chitwan	8.8	8.4	8.7	0.26	0.36	ns	2
Kanchanpur	9.5	8.8	9.3	0.29	0.42	ns	35
Morang	8.0	6.5	7.5	0.27	0.38	**	11
Nawalparasi	7.0	7.6	7.2	0.28	0.40	ns	37
Rautahat	3.4	2.6	3.1	0.26	0.37	*	68
Overall	6.96	6.37	6.8	0.15	0.21	*	

Source: Poverty index calculated from household survey data using the indicators shown in Table 14.
* P< 0.05, P<0.01, ns not significant.

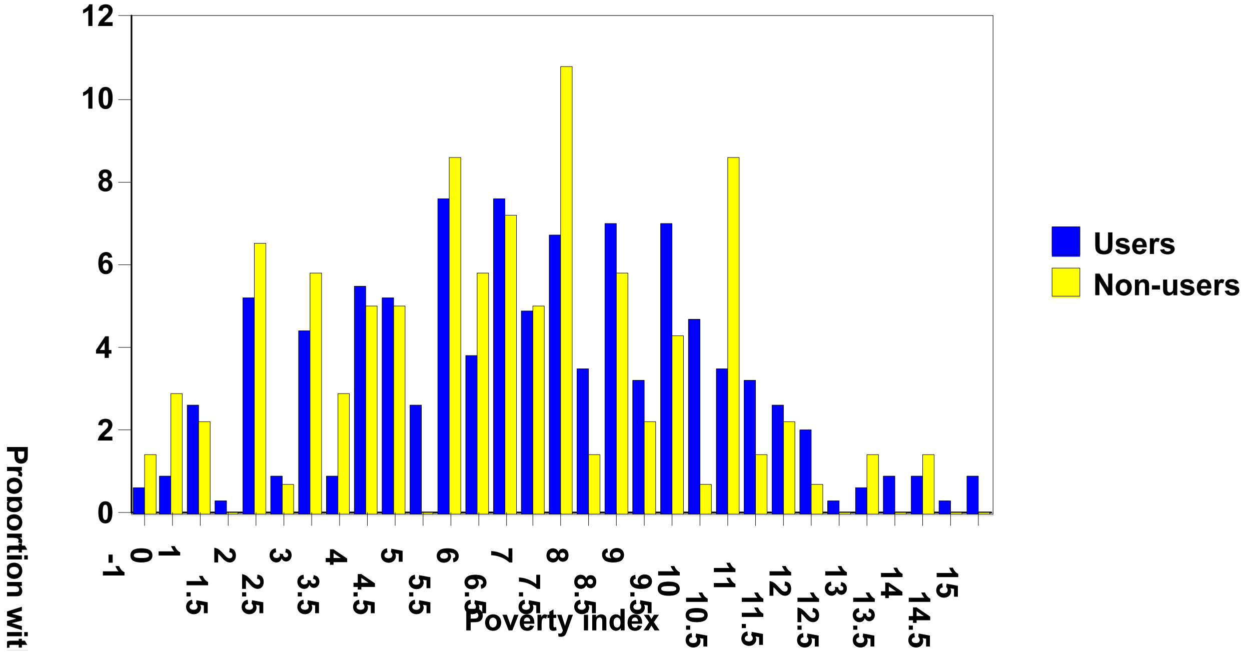

Fig. 21. The distributions of the poverty index for both the user and the non user groups.

In the broader context, Nepal is one of the poorest countries in the world and is 142nd ranked from 177 countries (source for 2007/2008 data: UNDP Human Development Reports http://hdrstats.undp.org/indicators/26.html). The interventions were targeted within Nepal almost exclusively to the terai although there is use in the low hills. At the time the RNRRS projects were formulated, the average poverty and deprivation index of the UN was 0.47 both for all of Nepal (UNDP, 2002) and for the terai as a whole (on a scale of 0 for least developed to 1 for most developed). This overall average development in the Terai was only because a few districts are highly developed; of the 20 Terai districts, 14 were average, or below average, in development. Rautahat, the poorest district in the Terai has a population of over 500,000 and was the fourth poorest district in Nepal. More recent UNDP data now show that the terai is now slightly better off on average than the rest of the country.

Analysis by use of individual COB varieties. An analysis was made of the difference between users and non-users of Sunaulo Sugandha as this was the only variety with more than 30 users (Table 29) among the 344 household surveyed users of any PVS or COB variety. There were 53 users of this variety in the three districts where it had the highest use; Kanchanpur, Nawalparasi and Chitwan. The significant differences in the socio-economic parameters between the users and non-users (Table 29) reflected the adaptation of Sunaulo Sugandha for fertile medium and lowland conditions. On average the farmers that adopted Sunaulo Sugandha had more medium land and significantly more grain production. Nonetheless, these differences were not uniform across all three districts - in Kanchanpur the medium land of the users and non-users was the same (1.2 ha) and the per capita grain production of the users was somewhat lower (730 kg compared with 800 kg).

Only the case of variety Sunaulo Sugandha was analysed because the sample sizes of the other varieties were too small and there were no significant differences. However, it is possible that some of the other COB varieties may also be adopted by households that differ from the non-adopting households for wealth indicators. For example, Barkhe 1027 might be expected to be adopted by poorer households with more upland but the sample size of 19 users was too small for a meaningful analysis as in any one district the sample size of users was very small.

Table 29. Some major socio-economic indicators among users and non-users of Sunaulo Sugandha (among the 344 household surveyed users of any PVS or COB variety) in three districts, Kanchanpur, Nawalparasi and Chitwan, from a household survey in 2008.

	Category		
**Socio-economic trait**	**User of Sunaulo Sugandha**	**Non-user of Sunalo Sugandha**	**SED†**
Poverty index	**9.6**	**8.9**	**0.3**
Persons migrating (mean number)	0.4	0.4	0.05
Total grain (kg per year)	**6500**	**5130**	**670**
Grain production per capita (kg per person per year)	**750**	**610**	**80**
Total land (ha)	**1.1**	**0.8**	**0.1**
Total upland (ha)	0.1	0.1	0.01
Total medium land (ha)	**0.9**	**0.7**	**0.09**
Total lowland (ha)	0.1	0.1	0.01
Sample size	53	117	

†Standard error of the difference between the means. Data for significant traits are indicated in bold font.

REFERENCES

CAZS-NR & LI-BIRD. 2007. Assessment of the Adoption of Rice Varieties in Chitwan Produced from Client-Oriented Breeding or Identified by Participatory Varietal Selection using the Global Positioning System. A Repeat Household Survey in 2007 Funded by the Monitoring Impact Assessment and Learning Component (MIL) of the Research into Use Programme (RiU). CAZS Natural Resources, Bangor University, UK and the Local Initiatives for Biodiversity in Research and Development, Pokhara, Nepal.

CBS & ICIMOD. 2003. Districts of Nepal Indicators of Development update 2003. Kathmandu Nepal: Central Bureau of Statistics, International Centre forIntegrated Mountain Development

Gridley, H.E., Jones, M.P. and Wopereis-Pura, M. 2002. Development of new rice for Africa (NERICA) and participatory varietal selection. In *Breeding rainfed rice for drought-prone environments: integrating conventional and participatory plant breeding in South and Southeast Asia. Proceedings of a DFID Plant Sciences Research Programme/IRRI Conference, 12-15 March 2002, IRRI, Los Baños, Laguna, Philippines,* 23-28 (Eds J.R. Witcombe, L.B. Parr and G.N. Atlin). Bangor and Manila: Department for International Development (DFID) Plant Sciences Research Programme, Centre for Arid Zone Studies (CAZS) and International Rice Research Institute (IRRI).

Gyawali, S, KD Joshi, KD & JR Witcombe. 2002. Participatory plant breeding in rice in low-altitude production systems in Nepal. pp. 8-10 in Breeding rainfed rice for drought-prone environments: intergrating conventional and participatory plant breeding in South and Southeast Asia. Proceedings of a DFID Plant Sciences Research Programme/IRRI Conference,, 12-15 March 2002, IRRI, Los Baňos, Laguna, Philippines. Department for International Development (DFID) Plant Sciences Research Programme, Centre for Arid Zone Studies (CAZS) and International Rice Research Institute (IRRI), Bangor and Manila.

Joshi, GR, Paudel, PK, Rawal KB, & Singh, U. 2007. Assessment of the Adoption of Rice Varieties Produced from Client-Oriented Breeding or Identified by

Participatory Varietal Selection using Global Positioning System. SUPPORT Foundation, PO Box: 24, Mahendranagar, Kanchanpur, Nepal.
Joshi, K.D. & Sthapit, B.R. 1990. Informal research and development (IRD): A new approach to research and extension. LARC Discussion Paper 1990/4. Lumle: Lumle Agricultural Research Centre, Pokhara, Nepal.
Joshi, K.D., Sthapit, B.R., Subedi, M. and Witcombe, J.R. 2002. Participatory plant breeding in rice in Nepal. In: *Farmers, Scientists and Plant Breeding: Integrating Knowledge and Practice*, David A Cleveland & Daniela Soleri (Eds), CABI, Wallingford, UK 10:239-267.
Joshi, K.D. & Witcombe, J.R. 2002. Participatory varietal selection in rice in Nepal in favourable agricultural environments – A comparison of two methods assessed by varietal use. *Euphytica* 127: 445-458.ICIMOD. 1997. Districts of Nepal indicators of development. International Centre for Integrated Mountain Development, Kathmandu, Nepal, 119.
Pandey, S., & Rajatasereekul, S. 1999). Economics of plant breeding: The value of shorter breeding cycles for rice in Northeast Thailand. *Field Crops Research* 64: 187-197.
Rana, R.B., Joshi, K.D., Gyawali, S. & Witcombe, J.R. (2004) Participatory crop improvement project of Chitwan and Nawalparasi district of Nepal. A baseline report. Discussion Paper no. 5, Centre for Arid Zone Studies and Local Initiatives for Biodiversity, Research and Development (LIBIRD).
Rogers, E.M. (1962) Diffusion of Innovations. New York: Macmillan.
Statistical Yearbook of Nepal, 2001. Central Bureau of Statistics, Ramash Path, Tapathali, Kathmandu.
UNDP. 2002. Nepal Human Development Report 2001. Poverty Reduction and Governance. United Nations Development Programme, Pulchowk, Kathmandu, Nepal.
Witcombe, J.R., Joshi, A., Joshi, K.D., & Sthapit, B.R. 1996. Farmer participatory crop improvement. I: Varietal selection and breeding methods and their impact on biodiversity. *Experimental Agriculture* 32:445-460.
Witcombe, J.R., Joshi, KD, Rana, R.B. & Virk, D.S. 2001. Increasing genetic diversity by participatory varietal selection in high potential production systems in Nepal and India. *Euphytica* 122:575-588.
Witcombe, J.R., Joshi, K.D., Gyawali, S., Musa, A., Johansen, C., Virk, D.S. & Sthapit, B.R. 2005. Participatory plant breeding is better described as highly client-oriented plant breeding. I. Four indicators of client-orientation in plant breeding. *Experimental Agriculture* 41: 299-319.
Witcombe, J.R. & Virk, D.S. Number of crosses and population size for participatory and classical plant breeding. *Euphytica* 122:451-462, 2001.

Appendix 1. Districts in the Nepal terai with year of project entry and approximate seed quantities distributed by 2007.

District†	Rice area (ha)	Year of entry (with few trials)	Seed COB supplied (ha equiv.)*	Seed PVS supplied (ha equiv.)	Total COB&PVS (ha equiv.)	Intensity COB†† (%)	Intensity PVS†† (%)
**Chitwan**	33685	1997	680	242	922	2.02	0.72
**Kanchanpur**	44365	2002	174	7	181	0.39	0.02
Kailali	56700	2001	164	21	186	0.29	0.04
Bara	55050	2004	120	9	129	0.22	0.02
Mahottari	50320	2002	109	10	119	0.22	0.02
**Morang**	95050	2002	94	46	140	0.10	0.05
**Nawalparasi**	42652	1997	91	17	108	0.21	0.04
Saptari	72600	2002	85	22	106	0.12	0.03
Dhanusha	65500	2001	73	37	110	0.11	0.06
Jhapa	95000	2002	59	39	98	0.06	0.04
Sunsari	61775	2001	56	19	75	0.09	0.03
Siraha	67700	2002	54	17	71	0.08	0.02
Parsa	46706	2004	36	15	51	0.08	0.03
Rupandehi	71500	2004	36	38	74	0.05	0.05
Bardiya	34875	2001	32	32	64	0.09	0.09
**Rautahat**	48951	2002	26	9	35	0.05	0.02
Makwanpur	12230	2004	22	26	48	0.18	0.21
Dang	38850	2002	21	6	27	0.05	0.02
**Banke**	31840	2002	20	48	68	0.06	0.15
Sarlahi	44215	2001	11	10	21	0.03	0.02
Kapilbastu	70500	2002	11	51	62	0.02	0.07
Mean	54289	2002	94	34	128	0.21	0.08

† Districts ordered by the amount of seed distributed of COB varieties. Surveyed districts indicated in bold font.

* Hectare equivalent = kg seed supplied/50 assuming a seed rate of 50 kg ha^{-1}

†† Combined NGO and DADO efforts to scale out PVS and COB varieties. Calculated as the percentage area covered i.e. ((ha equivalents/rice area in district in ha) * 100.
