# Supplementary material for: Plant breeding can be made more efficient by having fewer, better crosses
Source: BMC Plant Biol. 2013 Feb 7;13:22. doi: 10.1186/1471-2229-13-22 (PMC3621587; doi:10.1186/1471-2229-13-22)
Supplement: Additional file 4 — The impact of new maize and rice varieties on the livelihoods of poor farmers in marginal agricultural areas of western India. In Livelihoods Summit. Udaipur, Rajasthan: Department for International Development (DFID) and Indian Farm Forestry Development Corporation (IFFDC) 27th-30th September; 2005. [file 1471-2229-13-22-S4.pdf]

MINISTRY OF AGRICULTURE AND COOPERATIVE

NATIONAL SEED BOARD

VARIETY RELEASE COMMITTEE

Variety Proposal Format for Commercial Release of Agriculture Crops

Barkhe 3004
(Suggested Name)

Proposal for the Release of Barkhe 3004: A rice (self-pollinated crop) variety developed from the cross Kalinga III/IR64 in Nepal

<u>Proposed by:</u>

Sanjaya Gyawali
John R Witcombe
Krishna D Joshi
Krishna P Devkota
Mahendra Tripathi

<u>A joint accomplishment of:</u>

Local Initiatives for Biodiversity Research and Development (LI-BIRD)
Center for Arid Zone Studies (CAZS) Natural Resources
National Rice Research Program (NRRP), Hardinath
Jaskelo Youth Club (JYC), Chitwan
District Agriculture Development Office (DADO) Chitwan

A rice variety bred by
Participatory Plant Breeding Program of LI-BIRD

List of other individuals involved in Barkhe 3004 development program

INSTITUTIONS	PROFESSIONALS INVOLVED	DESIGNATION
LI-BIRD	Mr. S Gyawali, Mr. M Subedi, Ms S Sunwar, Mr. M Chaudhary, Mr. M Gurung, Mr. Hari Poudel, Mr. K Rawal, Mr. Dev R Karki, Mr. D Poudel, Mr. P Shrestha, Mr. KB Limbu, Ms Durga Pandit, Ms. Parbati Neupane, Mr. Bhim Mr. Hari Ghimire, Mr. S Khanal, Mr. B Gadal, Mahato, Dr. A Subedi and Dr. PK Shrestha,	Plant Breeder, Asst. Plant Breeder, Project Coordinator, Community Organizers, Field Motivators
DFID PSP UK/University of Wales Bangor (CAZS) Natural Resources	Prof. Dr. John R Witcombe, Dr. Krishna D Joshi; Prof. Dr. Daljit Singh Virk, Dr. Katherine Steele	Plant Breeders, Statistician, Molecular Biologist
NRRP/NARC	Mr. B Chaudhary, Mr. D Chaudhary, Mr. T Akhtar, Dr. N Adhikari, Mr. RB Yadav	Plant Pathologists, Plant Breeders, Agronomists Scientists
DADO, Chitwan	Mr. Chandra K Devkota, Mr. Mahesh Regmi	Extensionists
IAAS, Rampur	Mr. Deepak Sharma Poudel	Lecturer and Plant Pathologist
DADOs of terai and inner terai districts	DADO Nawalparasi, DADO Bara, DADO Rautahat, DADO Kailali, DADO Kanchanpur, DADO Saptari, DADO Dhanusha, DADO Mahottari	Agronomists, Extension Officers, JT and JTAs
Chitwan	Mr. Dev R Sapkota, Mr. ….Chapagain, Mr. Prakash Poudel, Mr. Neuapne and	Leader farmers
Chitwan	Mr. Deepak Subedi and Mr. Hari Datta Mishra,	Jaskelo Youth Club

And other scientists and support staffs of different institutions.

Background

Nepal can be broadly divided into three parallel geographic regions based on topography. Proceeding from the east to the west is the terai, or lower elevation fringe of the Gangetic plain in the southern border at 100-500 m elevation with an annual rainfall of more than 1600 mm; the middle hills at altitudes between 500 m and the forest line at 4000 m; and the high mountains of the Himalayas in the north, extending above the forest line as high as 8000 m. With more than 50% of the cultivated land in the country, the terai is the granary of Nepal. Mountain regions cover only 5% of the cultivated area. The population of the country was more than 21.4 million in 1995. The economy is largely rural, with almost 90% of the population engaged in agriculture. The population has been growing at 2.5% per year.

Agriculture contributes about 60% to GDP, provides employment to over 38% of the total population, and produces 80% of the value of exports. Rice, maize, and wheat are the three most important crops, occupying about 55%, 29%, and 23% of the cultivated area, respectively, while millet and oil seeds cover about 10% of the area. Rice (65% of the cultivated plains area) and wheat (25%) are dominant in the terai. Maize (38%), rice (28%), and millet (18%) are the most important crops in the hills.

Rice (*Oryza sativa* L.) is the most important food crops of Nepalese agriculture. The main diet of the Nepalese is also rice. Almost all people in the terai and river basins depend on this crop. In Nepal, rice is cultivated in 1.4 million ha land where more than 70% (1.1 m ha) rice is under rainfed condition resulting drought and flooding stresses each year (CBS, 2003). It is reported that 9% of rice area is cultivated under extreme drought condition in unbunded rice field called *Ghaiya* ecosystem. Similarly 3% rice area is occupied by high altitude rices (grown above 1500 masl) suffered by cold stresses each year.

The rice-growing part of Nepal is characterized as warm subhumid subtropics with summer rainfall. However, within the short span of its width can be found all varieties of climate and topography. The topography varies from the plains of the terai (the narrow band of plains adjacent to the foothills of the Himalayas) to the deep valleys of high mountains of the north.

Rice farming dominates the agricultural sector of Nepal, which itself dominates the economy. It is, therefore, the single most important industry in the country, contributing approximately one-fourth of the GDP and occupying approximately 1.4 million ha. From 1981 to 1994, production has increased at about 2.4% per year. Virtually all of the increase in total rice production came from increases in the area cropped but the productivity remained stagnant. It is estimated that roughly 7% of the total rice area are double-cropped.

Lack of farmer preferred varieties, low or no of agricultural inputs (irrigation, fertilizer and pesticides) and use traditional farming practices are considered reasons behind poor productivity of rices which predispose rice production to the biotic (disease and insect pest) and abiotic stresses (drought, cold and poor soil fertility).

Abiotic stresses such as drought, flooding and cold injuries are common in Nepal. Furthermore, these abiotic stresses predispose the rice varieties to biotic stresses such as insect pest and disease resulting poor productivity. In another hand, farmers have narrow varietal choices to manage these stresses on-farm. The introduction of stress tolerant rice germplasm either from IRRI or National Rice Research Program have realized less successes

in abiotic environments as compared to favorable and high potential productions systems in Nepal. Rana (2004) reported that farmers manage stresses under *Uchha* (rainfed bunded rice field- drought) and *Nichha* (rainfed-water logged and flooding condition with poorly drained rice field) deploying local landraces because these farmers either have little or virtually no varietal choices. In this context, participatory plant breeding has been used as an strategy of maximum use of local landraces in one of the parents in crossing program and integrating farmers local knowledge on target population of traits (TPTs) (traits tolerant to stresses) and target population of environments (TPEs) (selection under stress conditions in on-farm). Therefore, the PPB program of LI-BIRD in collaboration with NARC, IPGRI, CAZS and farming community has focused to bred rice varieties suitable under stress specially drought and poor fertility conditions, poor drainage conditions in on-farms. Highly drought tolerant rice varieties such *as Barkhe 1027, Sugandha 1, Judi 572* and *Judi 582* have been bred by PPB program of LI-BRID and spreading farmers to farmers in Nepal and Bangladesh (Gyawali *et al.,* 2002; Witcombe *et al.,* 2004). In in-situ conservation agrobiodiversity project, drought and poor fertility tolerant rice varieties such as *Mansara* and *Aanga* have been used in PPB program to add value to these valuable stress tolerant varieties on-farms (Gyawali *et al,* 2004).

Among biotic stresses plant diseases and insects pest are major threat to rice cultivation in Nepal. Leaf and neck blast caused by *Pyricularaia grisea* and Bacterial Leaf Blight (BLB) caused by *Xanthomonas campestris* pv. *oryzae*) are major diseases in rainfed rice cultivation whereas rice borer is major insect pest to rice in Nepal. Deployment of resistant or tolerant cultivars is the most effective way of managing disease and insect pest where little or no pesticides is applied as biotic stress management by susstiance rice cultivation in Nepal.

This proposal summarizes the a number of studies carried out in these stress prone and high potential production areas in order to understand the situation for the genotype that is being proposed for release. Barkhe 3004 is a variety that is preferred by the farmers and is characterized by a number of desirable traits such as high yielding, resistant to blast and bacterial leaf blight and other major diseases and insect pest, has excellent post harvest quality traits such as micro milling and organoleptic traits. Barkhe 3004 is 22% and 20% higher yielding than Ram Bilas and Masuli (most popular variety in rainfed lowland in Nepal) on an average. It has produced 4532 kg/ha of grain yield in ideal conditions.

General information

1) Common and Botanical Names: Rice (*Oryza sativa* L.)
2) Original Designation: Barkhe 3004
3) Cross (parents): Kalinga III/IR64
4) Selection History: F3SC98-LDBM99-BM00-S6M01-BM02-BM03-BM04
4) Country of origin: Recombined at IRRI, The Philippines
5) Source of materials (Name of the experiment and year it was first introduced): F_3 generation introduced to breeding nursery of Participatory Plant Breeding program of LI-BIRD in 1998 in Chitwan

6) Years, experiments and locations in Nepal, the variety was tested

APPENDICES	YEAR	EXPERIMENT	LOCATION REFERENCE
	2004	CVT	NRRP, Hardinath
	2004	Mother-baby trials	LI-BIRD, Chitwan and Nawalparasi
	2004	Fertilizer trials	LI-BIRD, Chitwan and Nawalparasi
	2004	National Blast Screening Nursery	NRRP, Hardinath,
	2004	National Bacterial Leaf Blight Screening Nursery	NRRP, Hardinath,
	2003	CVT	NRRP
	2003	Mother-baby trials	LI-BIRD, Chitwan and Nawalparasi
	2003	National Blast Screening Nursery	NRRP, Hardinath,
	2003	National Bacterial Leaf Blight Screening Nursery	NRRP, Hardinath,
	2003	Screening PPB bred varieties for diseases	LI-BIRD, Chitwan and Nawalparasi
	2002	Mother-baby trials	LI-BIRD, Chitwan and Nawalparasi
	2002	National Blast Screening Nursery	NRRP, Hardinath,
	2002	National Bacterial Leaf Blight Screening Nursery	NRRP, Hardinath,
	2003	CBSP	Unnat Seed Producer Group, Patihani,
	2004	CBSP	Unnat Seed Producer Group, Patihani,<br>Sri Ram Seed Producer Groups, Phulbari

3. Summary of Varietal Characteristics

3.1. Agronomic

3.1.1	Plant height (cm)	: 97±5.8
3.1.2	Days to 50% heading from seeding	: 95 days
3.1.3	Days to maturity from seeding	: 153±7.0 days
3.1.4	Yield (Kg ha^{1})	: 4532±753

1.1.5. Other yield components

3.1.4.1	Tiller $hills^{-1}$ (No)	: 8
3.1.4.2	Panicle m^{-2} (No)	: 264±9.9
3.1.4.3	Panicle length (cm)	: 27.26±2.19
3.1.4.4	Grain per panicles (No)	: 113±31.5
3.1.4.5	1000 grain weight (g)	: 26.04
3.1.4.6	1000 milled grain weight (g)	: 21.4

3.2. Response to stresses (please specify):

3.2.1 Biotic stresses

(a) **Insects:** The incidence and severity of insect pest was assessed in mother trials in 2003 and 2004. We did not find any specific problems of insect pest in Barkhe 3004. We noticed this variety was resistant to Borer and Lear folder in mother trials.

(b) **Diseases:** Barkhe 3004 has multiple disease resistance contributed from one of its parents IR 64. This is highly resistant to leaf and neck blast (App.) and bacterial leaf blast. Barkhe 3004 is evaluated in National Blast Nursery and National Bacterial Leaf Blight Nursery by NRRP in 2002, 2003 and 2004. In each year, we found Barkhe 3004 resistant to these major diseases. Besides this, Plant Pathologist from IAAS assessed its response to major diseases in on-farm trials. The results indicated this variety was resistant to Blast, BLB, Brown Spot and tolerant to Sheath Blight (App.).

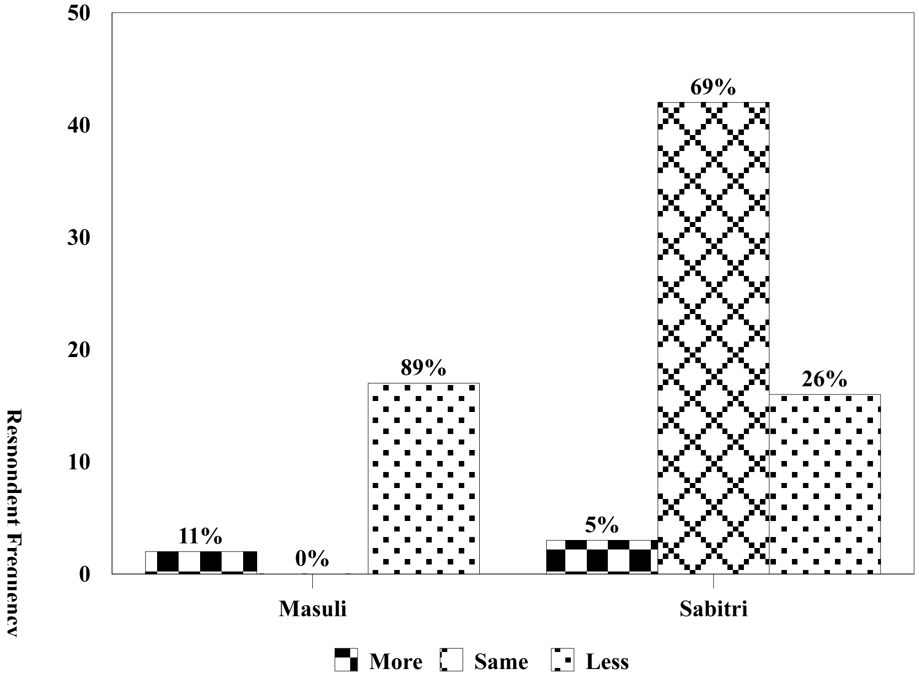

Figure Farmers' response on diseases and insect pest resistance (Blast, Bacterial Leaf Blight and Borer) of Barkhe 3004 compared with popular rice varieties in 87 baby trials in 2004.

(c) Weeds: We did not notice any specific weed problem associated in this variety.

3.2.2 Abiotic stresses

(a) Adverse climatic conditions:
(b) Adverse soil conditions:

(c) Other stresses, if any: In 2004, Nepal experienced extreme drought in many districts and we found Barkhe 3004 has tolerant to drought conditions. In Bara, Simraungarh, farmers noticed and reported significant yield loss in Sona Masuli whereas Barkhe 3004 had the least effect of drought on grain yield. Kalinga III, which is one of the parents of this variety, might have contributed to the drought tolerance to this variety. In Chitwan, farmers experienced drought tolerant characters in Barkhe 3004 and had least effect of lodging on grain yield and quality as compared to Sabitri and Masuli (Figure). We recorded that more than 90% farmers who had compared Barkhe 3004 with Masuli found that it is highly lodging tolerant whereas 18% farmers reported that it is less prone to lodging as compared to Sabitri. However

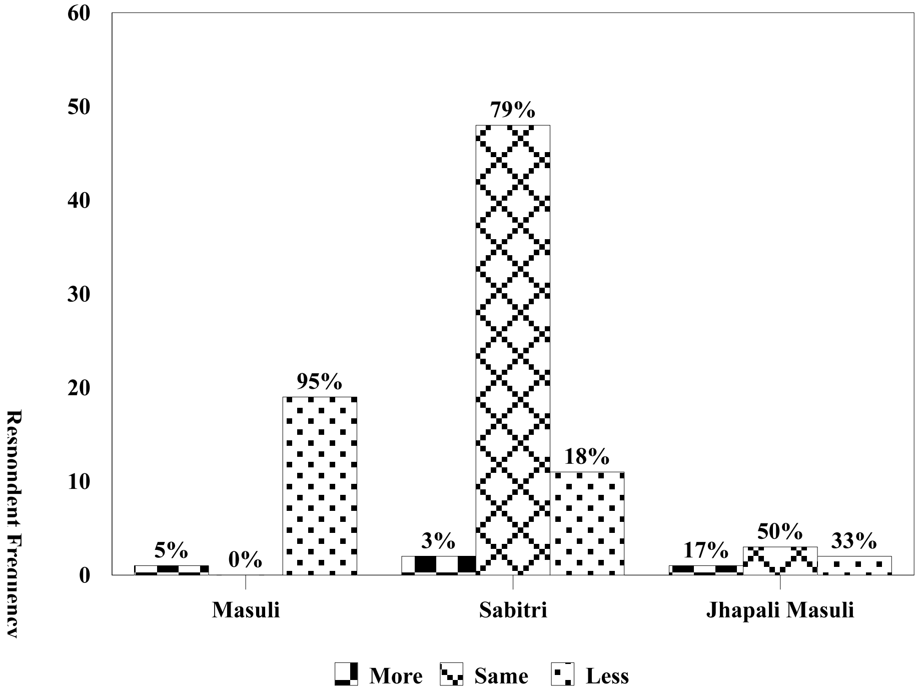

Figure Farmers' response on lodging of Barkhe 3004 compared with popular rice varieties in 87 baby trials in 2004.

3.3. Grain/leaf/root/curd/bulb/seed/fruit/tuber qualities:

3.3.1 Nutritional quality (if available):

3.3.2 Processing quality:

Table Visual observation of grain quality of Barkhe varieties and popular rice varieties of Nepal recorded in participatory evaluation in Chitwan in 2003.

Variety	Rice Color	Grain type		White	Grading	Selection/	Remarks
		Bold/fine	Long/short	Belly %	rice	Rejection	
Sabitri	Ghee	Bold	Long	10	2	S	
Mansuli	White-ghee	Medium	Medium	5	1	S	Less broken
Barkhe 2014	Ghee	Bold	Short	60	2	S	
**Barkhe 3004**	**White-ghee**	**Medium**	**Long**	**10**	**2**	**S**	**Less broken**
Sworna	White-ghee	Medium	Medium	2	1	S	
Mansuli	Ghee	Medium	Medium	2	1	S	
Sabitri	Ghee	Bold	Long	10	2	S	

Overall Grading of rice- 1=Excellent and highly preferred for cooking, 2= Good and preferred for cooking, 3=Rejected for cooking and eating quality

Table Visual observation of grain quality of Barkhe varieties and popular rice varieties of Nepal recorded in participatory evaluation in Chitwan in 2004.

Variety	Length	Width	Color	Grain Breakage	Grading rice	White belly	Decision	Remarks
Barkhe 3017	Medium	Medium fine	Ghee white	1	1	1	Selected	
Sabitri	Medium	Medium fine	Ghee	1	1	1	Selected	
**Barkhe 3004**	**Long**	**Bold**	**Ghee white**	**2**	**2**	**1**	**Selected**	

Pusa 834	Long	Medium fine	Ghee	3	2	1	Selected	
Radha 4	Short	Bold	White	2	3	3	Rejected	
Barkhe 2022	Long	Medium fine	White ghee	1	2	2	Selected	
CNTLR	Long	Medium fine	Ghee	1	1	1	Selected	Special aroma
Masuli	Medium	Medium fine	Ghee	1	1	1	Selected	
Sugandha 2002	Long	Medium fine	Ghee	2	1	1	Selected	Aroma
Barkhe 2001	Long	Medium fine	Ghee white	1	1	1	Selected	
BPI	Medium	Medium fine	Ghee white	1	2	2	Selected	
GAM-WAN	Long	bold	Ghee dull	2	2	1	Selected	Light aroma

Grain breakage- 1=Grain not broken, 2=Grain slightly broken, 3=Lemma and palea completely broken and opened; Overall Grading of rice- 1=Excellent and highly preferred for cooking, 2= Good and preferred for cooking, 3=Rejected for cooking and eating quality; White belly- 1=translucent, 2= <10% area of rice, 3= 11-20% of the area of rice.

Farmers' responses on post harvest grain quality of Barkhe 3004 have been found extremely encouraging. We analyzed data of 87 baby trials conducted in Chitwan and Nawalparasi districts and found that more than 90% Sabitri growing farmers reported that Barkhe 3004 has higher milling recovery. Also 26% Masuli growing farmers reported that it has higher milling recovery. It is noteworthy that farmers and rice merchant take Masuli and Sabitri as reference variety for rice recovery and Barkhe 3004 has been found to have higher rice recovery in on-farm conditions.

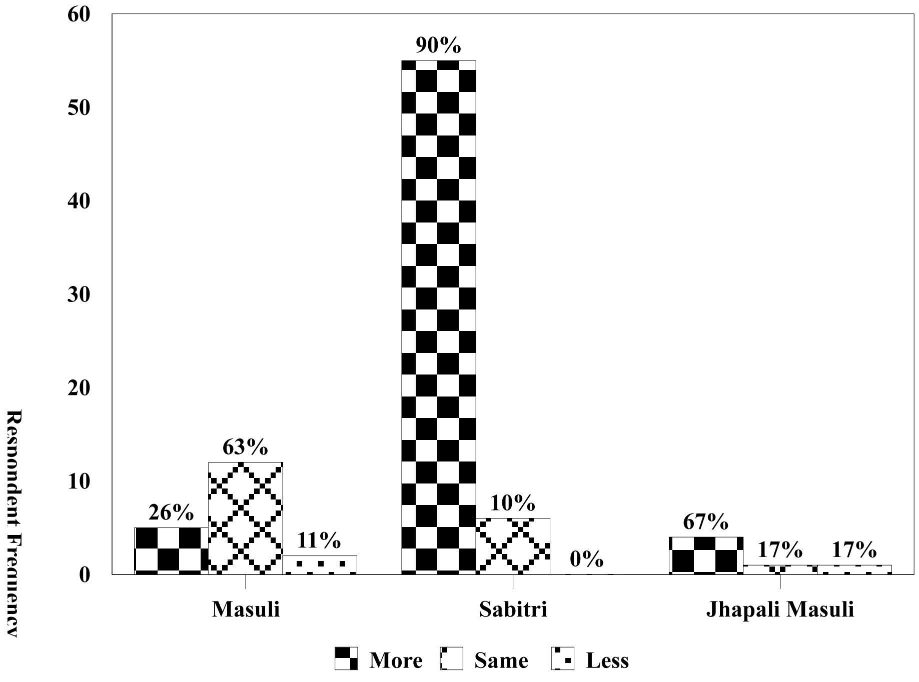

Figure Farmers' response on milling recovery of Barkhe 3004 compared with popular rice varieties in 87 baby trials in 2004.

3.3.3 Organoleptic test (cooking quality, taste, aroma/flavor, etc):

Table Organoleptic assessment of cooked rice of Barkhe and popular rice varieties of Nepal recorded in participatory evaluation in Chitwan in 2003.

Variety	Softness	Flakiness	Taste	Water Absorption	Inner Hardiness	Selection/ Rejection
**Barkhe 3004**	**Medium**	**Good**	**Tasty**	**High**	**Present**	**Selected**
Jhapali masuli	Medium	Medium	Medium	High	Present	Selected
Barkhe 2014	Soft	Good	Medium	Medium	Absent	Selected
Masuli	Soft	Good	Tasty	Medium	Absent	Selected
Sworna	Medium	Medium	Medium	Low	Absent	Selected
Sabitri	Medium	Good	Tasteless	High	Present	Rejected
Masuli	Soft	Good	Medium	Medium	Absent	Selected
Pusa basmati	Soft	Good	Tasty	High	Absent	Selected
Sarwati	Soft	Good	Tasty	Medium	Absent	Selected

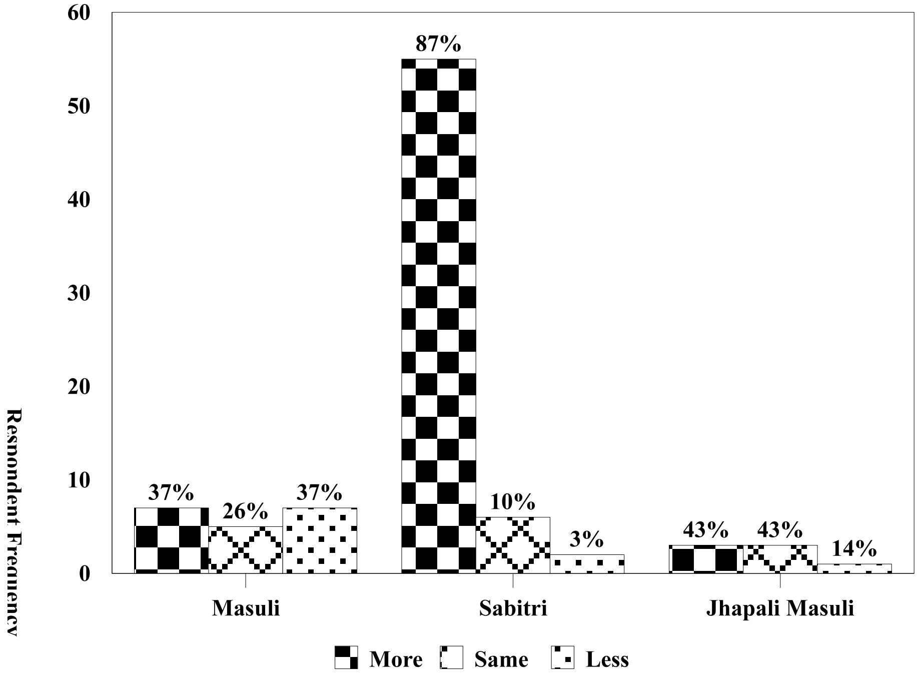

Figure Farmers' response on eating qualities of Barkhe 3004 compared with popular rice varieties in 87 baby trials in 2004.

Table Organoleptic assessment of cooked rice of Barkhe series, advanced NRRP liens and popular rice varieties of Nepal recorded in participatory evaluation in Chitwan in 2004.

Variety	Softness	Flakiness	Taste	Inner hardiness	Water absorption	Quality of rice	Aroma	Decision
Barkhe 3017	2	2	2	3	1	2	Absent	Rejected
**Barkhe 3004**	**1**	**3**	**2**	**2**	**2**	**2**	**Absent**	**Selected †**
Pusa 834	1	3	1	1	3	1	Absent	Selected
CNTLR	1	2	1	1	2	1	Present	Selected
Masuli	1	2	1	1	1	1	Absent	Selected
Barkhe 2022	2	3	2	2	3	1	Absent	Rejected
Sugandha 2002	1	1	1	1	1	1	Present	Selected

Barkhe 2001	1	1	1	1	1	1	Absent	Selected
BPI	1	2	1	1	2	2	Absent	Selected
Sabitri	2	2	1	2	2	1	Absent	Selected
GAN- WAN	1	2	1	2	2	2	Present	Selected

Scoring: 1= Highly preferred, 2= Accepted, 3=Poor and rejected; †= Volume expansion noticed the highest among test entries.

3.3.4 Other specific qualities (if any):

3.4. Other characteristics (threshing, storability, market potential etc.):

Barkhe 3004 is rated as easily threshable variety and it does not shatter in the field. If is considered as easy as Masuli in terms of threshability. Nepalese farmers value threshability as an important post harvest traits because varieties with difficult threshability will cause more cost for manual threshing. Barkhe 3004 can be threshed manually by 3-4 bits which is much easier than Sabitri which require 6-7 bits to thresh.

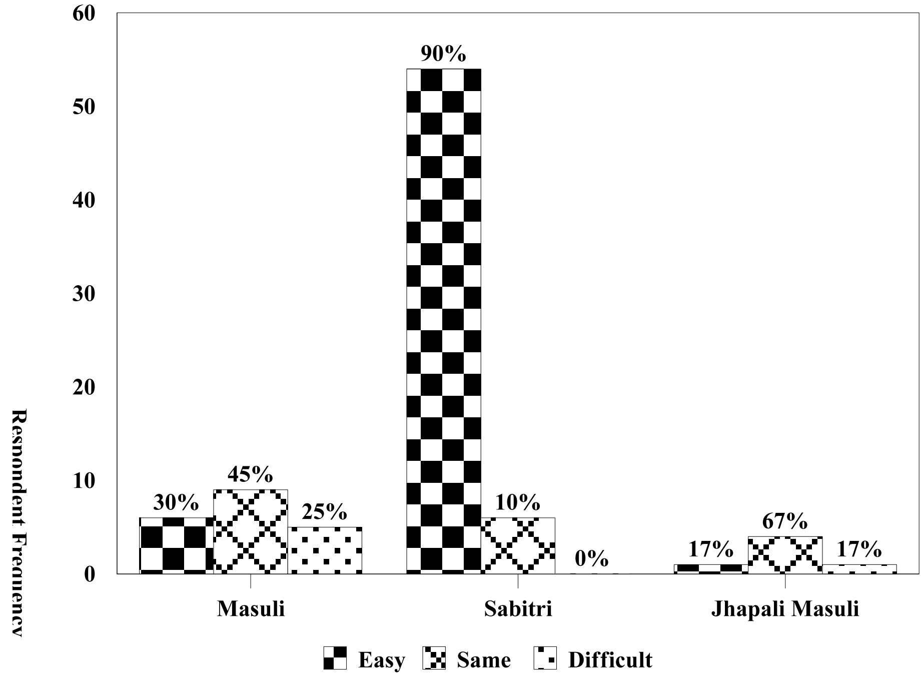

Figure Farmers response on threshability of Barkhe 3004 compared with popular rice varieties in 87 baby trials in 2004.

Barkhe 3004 is an excellent variety in terms of storability since it can tolerate storage grain pest. Farmers involved in 87 baby trials reported that they experienced less pest in stored grain (milled and fresh paddy both).

Barkhe 3004 is perceived a good variety in the market for its high rice recovery in milling, good cooking and eating qualities. Farmers are able to get the similar price to Sabitri for this variety even through this is a new variety to the rice merchants. There is a good market potential for this variety in Nepal.

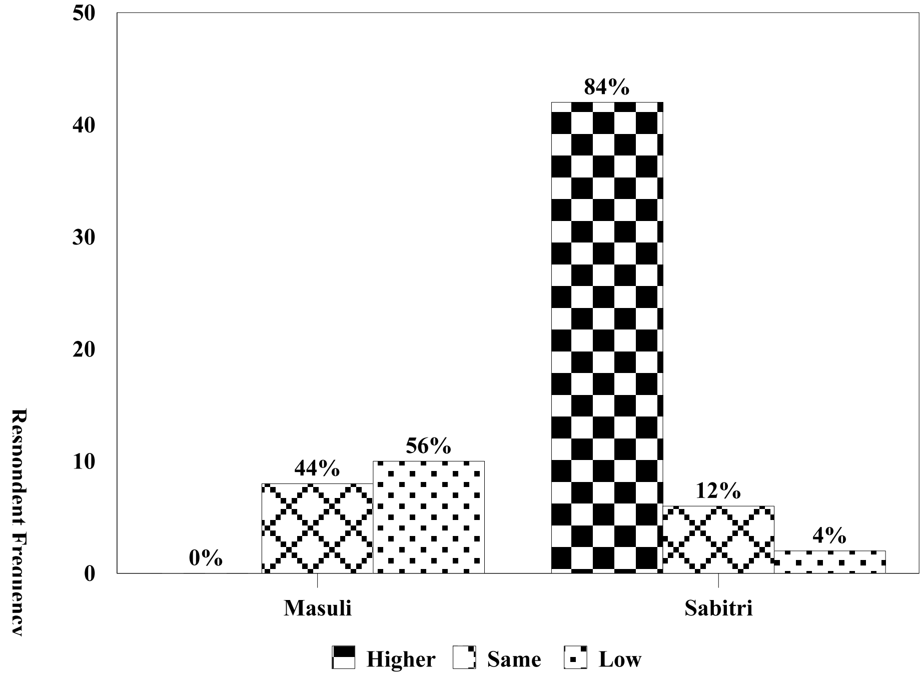

Figure Farmers' response on market price of Barkhe 3004 compared with popular rice varieties in 2004.

4. <u>Morphological characteristics (size, shape, color, etc of various plant parts):</u>

4.1.1 Leaf Characters:

4.1.1.1	Length	: 21.8 cm
4.1.1.2	Width	: 1 cm
4.1.1.3	Blade Pubescence	: Intermediate
4.1.1.4	Blade color:	: Dark Green
4.1.1.5	Basal leaf sheath color:	: Green
4.1.1.6	Angle	: Erect
4.1.1.7	Flag leaf angle	: Erect

4.1.2 Ligule Characters:

4.1.2.1	Length	: 2.2 cm
4.1.2.2	Color	: White
4.1.2.3	Shape	: Cleft
4.1.2.4	Collar Color	: Pale Green
4.1.1.5	Auricle Color	: Pale green
4.1.3	Days to heading (number of days from effective seeding date to 50% heading)	: 95 days

4.1.4 Culm Characters :

4.1.4.1	Length	: 78.5 cm
4.1.4.2	Number	:
4.1.4.3	Angle	: Erect
4.1.4.4	Culm Diameter	: 0.549 cm

4.1.4.5	Internode color	: Green
4.1.4.6	Strength (lodging resistance)	: Strong (no lodging)

4.1.5. Panicle Characters:

4.1.5.1	Length	: 21.57 cm (average of 10 panicles)
4.1.5.2	Type	: Intermediate
4.1.5.3	Secondary Branching	: Heavy
4.1.5.4	Exertion	: Well exerted
4.1.5.5	Axis	: Droopy
4.1.5.6	Shattering	: Very low (<1% at the time of maturity)
4.1.5.7	Threshability	: Easy

4.1.6. Grain (Spikelets) Characters :

4.1.6.1	Awning	: awnless
4.1.6.2	Apiculus color	: Straw (yellow)
4.1.6.3	Stigma Color	: White
4.1.6.4	Lemma and Palea color	: Straw (yellow)
4.1.6.5	Lemma and Palea pubescence	: Hairy on upper portion
4.1.6.6	Sterile lemma color	: Straw (yellow)
4.1.6.7	Sterile lemma length	: Short (not longer than 0.5 mm)
4.1.6.8	Spikelet sterility	: Fertile (75-90%)
4.1.6.9	1000 grain weight	: 26.04 g
4.1.6.10	Length	: 9.23 cm
4.1.6.11	Width	: 2.53
4.1.6.12	Seed coat (bran color)	: Brown
4.1.6.13	Endosperm type	: non glutinous (non waxy)
4.1.6.14	Scent (aroma)	: non scented (non aromatic)
4.1.6.15	Leaf senescence	: Late and slow
4.1.7	Maturity (days from seeding- when 80% of the grains on panicles are matured)	: 157 days

4.8 Major identifying characteristics of a crop variety for cultivar authenticity:

4.9. Molecular characteristics (if available)

5. Recommendation domain:

5.1 Geographical area (altitude, latitude and longitude):

The variety can be grown in *terai* (east to west), inner *terai* and foothills of Nepal. The variety is recommended for sea level to 400 masl in foothills of Nepal.

5.2 Moisture regime: The variety is basically bred for rainfed intermediate production environment classified by IRRI. It is best suited for rainfed intermediate lowland and long standing water conditions (low lying area with poor drainage conditions). Barkhe 3004 is also adapted in irrigated and high potential production systems.

5.3 Climatic conditions: Barkhe 3004 is bred for main season rice Ashadh-Mansir (July –November) in Nepal. Therefore, the cropping period (seed to seed) has been recommended as Ashadh-Mansir (July –November) in Nepal.

5.4 Socioeconomic conditions:

5.5 Production and management aspect (please add detailed information, if necessary, on the general cultivation practices; appropriate input and moisture regimes):

5.5.1 Land Preparation: Land preparation can be followed as farmers practice. The ploughing can be done either by bullock drawn country plough or tractor drawn plough followed by two harrowing. Fields are then leveled, smoothened and puddle well.

5.5.2. Seed rate: Seed rate depends on germination percentage.
For quality seed 50 kg $ha^{-1}$is sufficient to have better crop stand.

5.5.3. Sowing methods: Transplanting- 24-30 days old seedlings are transplanted. The transplanting dept can be used as farmers practice

5.5.4. Fertilizer application: The fertilizer response trials conducted in 2004 revealed that 100:30:30 NPK kg ha^{-1} is recommended for Barkhe 3004 cultivation. We recommend applying 25 kg of N as top dressing at booting (panicle initiation) and 25 kg at flowering stages. Rest of the fertilizer is recommended to apply as basal dose. However, the application of fertilizer depends on soil fertility status and availability of fertilizer. If fertilizer is available for only one top dressing, then we recommend applying it at booting (panicle initiation) stage.

5.5.5 Spacing: We recommend 20 cm x 20 cm spacing for manual transplanting of Barkhe 3004.

5.5.6 Irrigation: Barkhe 3004 is bred for rainfed intermediate lowland and therefore is tolerant to drought stress. Kalinga III, one of the parents of this variety has contributed the drought tolerance to this variety. Also this variety is suited for poorly drained rice fields. However, we recommend irrigation at active tillering stage, panicle initiation and flowering stages if available. If only one irrigation is available, then one should irrigated Barkhe 3004 during booting (panicle initiation-50 to 60 days after transplanting) stage.

5.5.7 Disease: Barkhe 3004 is a progeny of Kalinga III/IR 64, therefore, it has multiples disease resistance. This variety is resistant to leaf blast, neck blast, bacterial leaf blight and tolerant to Sheath blight (App). IR 64 has contributed the multiple disease resistance to this variety. The resistance to BLB make this variety suitable in lowland and poorly drained soils where the blast and bacterial leaf blight is the major problem in Nepal.

5.5.8 Insect: There is no specific insect problem associated to this variety. Generally, Carbofuran 1 kg a.i. ha^{-1} equivalent to 33 kg of Furadan ha ¯1can be applied for controlling stem borer.

5.5.9 Intercultural operation: Two weedings are recommended for Barkhe 3004 cultivation. The first weeding is preferably recommended at 20-30 days after transplanting whereas second weeding would be better at 60 days after transplanting.

5.5.10 Harvesting: Technically harvesting of Barkhe 3004 is recommended when 95% of the grain in the panicles reach at physiological maturity. When 20-25% moisture in the grain is attained and crop reaches at physiological maturity, we recommend the crop for harvesting. Since, Barkhe 3004 is non shattering, manual or mechanical harvesting could be done. Farmers practice of harvesting and threshing such as sun drying for 2 days in the field and then manual threshing or by mechanical threshing can be followed.

5.5.11 Storage: Sun drying of harvested grain is a common practice in Nepal which could be followed for Barkhe 3004 too. The grain should be sufficiently dried in sun to bring grain moisture at 14-16% before grain is stored in Jute sacks under cool and dry storage.

5.6 Reasons for release of Barkhe 3004

- higher grain yield potential (22% higher yield than existing improved standard checks in CVT (Ram Bilas, Masuli, Radha 11) and on-farm trials (Masuli) (App);
- lodging tolerant under lowland condition where crop is grown under long standing water conditions. Also it suitable for high soil fertility conditions and do not lodge under these circumstances. The existing popular variety Masuli is severely lodged under these condition severely affecting the grain yield and post harvest grain qualities.
- "stay green" traits of leaves (plants remain green even after plant reaches physiological maturity. This trait helps farmers to harvest quality fodder for to feed their farm animals.
- it is resistant to blast (leaf and neck) and bacterial leaf blight (BLB). Furthermore, Barkhe 3004 is tolerant to many disease and insect pest (see App.)
- preferred by many farmers over their existing varieties (mainly over Masuli, and Kanchi Masuli-Aus 781) (App mother baby trials)
- proved to be a better variety than their existing varieties (Ram Bilas, Masuli, Kanchi Masuli and Sworna) in both on-station and on-farm situations.
- preferred by farmers for its ease of threshability and no shattering problem for manual as well as mechanical threshing;
- preferred and selected for good post harvest qualities such as milling traits high head rice recovery, good cooking and eating quality, and good market price (App);
- farmer need varietal choices to satisfy their multiple needs under their varied agro-climatic conditions. For example, one variety may perform better under one circumstance/location but may perform poorly when it is taken to another location within the same altitude.

6. Seed supply situation:

Table Seed supply status of Barkhe 3004 in Nepal.

CATEGORY OF SEED	AMOUNT AVAILABLE (KG)	YEAR	WHERE AVAILABLE	PERSON HANDLING
Breeder	50 kg<br>50 kg<br>50 kg	2003,<br>2004,<br>2005	LI-BIRD	Mr. Sanjaya Gyawali
Foundation	1000 kg<br>1000 kg<br>1000 kg	2003,<br>2004,<br>2005	LI-BIRD	Mr. Krishna P Devkota
Truthfully labeled seed	6000 kg<br>7000 kg<br>7000 kg	2003,<br>2004,<br>2005	LI-BIRD	Sharmila Sunwar<br>(Seed Unit of LI-BIRD)
Truthfully labeled seed		2003,<br>2004,<br>2005	Dev Ujjwal Agri. Cooperatives	Mr. Dev Raj Sapkota<br>Mr. Krishna P Chapagain
Truthfully labeled seed		2004<br>2005	Unnat Seed Producer Group, Chitwan	Mr. Prakash Poudel
Truthfully labeled seed		2004<br>2005	Sri Ram Seed Producer Groups, Phulbari, Chitwan	Mr. Chiranjibi Neupane

Proposed by

…………………………………
Signature

Name: Sanjaya Gyawali
Designation: Plant Breeder
Organization: Local Initiatives for Biodiversity, Research and Development (LI-BIRD)
PO Box No. 324, Pokhara, Kaski, Nepal
Telephone: 977 61 526834/532912/535357
Fax: 977 61 539956
Email: sgyawali@libird.org
URL: www.libird.org

2004 Yield Components

Coordinated varietal trial rainfed low land medium (CVT RLM), 2004

The coordinated varietal trials were conducted by National Rice Research Program in collaboration with various regional and agriculture research stations of NARC. LI-BIRD and NARC formally signed an LoA to initiated the evaluations of PPB bred rice varieties for the multilocational testing, disease screening and other advanced evolutions within Nepal. Similarly LI-BIRD contributed evaluation of various NRRP bred rice varieties in on-farm as well as other participatory evaluations such as micro milling and organoleptic assessments. The summary of performances of Barkhe 3004 in CVT and Disease Screening Nurseries are summarized for 2002, 2003 and 2004.

App.1. Table Coordinated varietal trail rainfed lowland medium (CVT RLM) 2004.

SN	VAREITIES	DAYS TO MATURITY		
		NRRP/H	RARS/P	LOC Mean
1	SUGANDA 2002	151	153	152
2	NR 268-4-6-1-4	146	148	147
3	NR 1190-24-4	142	141	142
4	BARKHE 2001	134	134	134
5	PSBRC 70	158	158	158
**6**	**BARKHE 3004**	**148**	**154**	**151**
7	NR 1893-17-2-3	148	151	150
8	NR 1894-10-3-2-3	158	159	159
9	BARKHE 2045	136	135	136
10	NR 1892-20-21-1-1-2	148	151	150
11	NR 1887-4-3-1-1-2	143	141	142
12	IR 62558 – SRN-17-2	149	146	148
13	NR 1887-8-1-1-2-2-2	141	146	144
14	MASULI	147	146	147
15	MAKAWANPUR – 1	159	160	160
**16**	**LOCAL CHECK**	**160**	**144**	**152**
	F test	**	**	
	CV %	112	0.9	
	GRAND MEAN	119	147	
	FLSD 0.05	2.3	1.9	

NOTE : Local check- In NRRP/Hardinath = Ram Bilash; In RARS/Parwanipur = Radha 11

Source: Chaudhary et al., 2005.

The maturity of Barkhe 3004 was found the most ideal for rainfed lowland and long standing water conditions because this variety matured in 152 days in 2004 and 148 days in 2003 on an average (App 1 and 9). The maturity of this variety was found the most suitable to farmers in on-farm trials (mother and baby trials) because it matured at the same time of farmers' popular variety Masuli. The test weight of Barkhe 3004 was 22.9 g in and 23.9 in 2004 and 2003 respectively. Therefore we concluded that this variety is regarded as medium coarse (similar to Sabitri) as described by farmers as well as rice merchant.

App.2. Coordinated varietal trial rainfed low land medium (CVT RLM), 2004

S.No.	VARIETIES	PLANT HEIGHT			PAN / M^2		
		NRRP/H	RARS/P	LOC Mean	NRRP/H	NMRP/P	LOC Mean
1	SUGANDA 2002	105.5	97	101.2	294	274	284
2	NR 268-4-6-1-4	128.5	123	125.7	246	214	230
3	NR 1190-24-4	125.0	116	120.5	279	241	260
4	BARKHE 2001	104.0	108	106	261	301	281
5	PSBRC 70	90.5	89	89.7	265	228	246
**6**	**BARKHE 3004**	**88.7**	**79**	**83.8**	**316**	**301**	**308**
7	NR 1893-17-2-3	91.7	83	87.3	302	285	293
8	NR 1894-10-3-2-3	103.7	94	98.8	329	257	293
9	BARKHE 2045	135.5	104	119.7	275	138	206
10	NR 1892-20-21-1-1-2	89.4	84	86.7	306	276	291
11	NR 1887-4-3-1-1-2	149	136	142.5	235	185	210
12	IR 62558 – SRN-17-2	103.7	94	98.8	287	288	287
13	NR 1887-8-1-1-2-2-2	102.5	97	99.7	273	345	309
14	MASULI	117	116	116.5	321	251	286
15	MAKAWANPUR – 1	86	85	85.5	297	273	285
**16**	**LOCAL CHECK**	**108**	**109**	**108.5**	**365**	**271**	**318**
	F test	**	**		**	**	
	CV %	4.6	5.9		14	10.3	
	GRAND MEAN	108.7	102		290	265	
	FLSD 0.05	7.1	8.3		57	12	

NOTE : Local check- In NRRP/Hardinath = Ram Bilash; In RARS/Parwanipur = Radha 11

Source: Chaudhary et al., 2005.

The plant height of Barkhe 3004 is medium dwarf (ranged from 84-90 cm) in different CVT and mother and baby trials (App 2 and 10). This variety is very much sturdy and resistant to lodging. Due to its shorter plant height it can tolerate higher fertility and long standing water condition. The plant stature of this variety is contributed by its one the parents IR 64 which is also medium dwarf with high level of resistance to lodging and high fertility.

The number of fertile panicles per square meter is one of the important characters of this variety (App 2 and 10). We recorded higher panicle per square meter for Barkhe 3004 as compared other advanced lines. This trait has attributed to the higher grain yield of this variety. The grain per panicle of this variety was comparable and higher than Standard Checks.

The grain yield of Barkhe 3004 is always higher than Standard Checks in CVT in 2004 and 2003. We found the Barkhe 3004 yield 20% more grain yield than Standard Checks (Masuli, Ram Bilas and Radha 11) (App 3 and 11).

App.3. Coordinated varietal trial rainfed lowland medium (CVT RLM), 2004

SN	VARIETIES	1000 g wt (gm)		GRAIN YIELD kg/ha		
		NRRP/H	LOC Mean	NRRP/H	NMRP/P	LOC Mean
1	SUGANDHA 2002	20	20	2620	2704	2662
2	NR 268-4-6-1-4	17.9	17.9	3222	3244	3233
3	NR 1190-24-4	24.1	24.1	3357	3165	3261
4	BARKHE 2001	21.2	21.2	2911	2879	2895
5	PSBRC 70	27.9	27.9	2128	2008	2068
**6**	**BARKHE 3004**	**22.9**	**22.9**	**3470**	**2900**	**3185**
7	NR 1893-17-2-3	21.5	21.5	3089	3131	3110
8	NR 1894-10-3-2-3	26.5	26.5	3215	3103	3159
9	BARKHE 2045	19	19	1821	1094	1457
10	NR 1892-20-21-1-1-2	21.4	21.4	2563	3160	2862
11	NR 1887-4-3-1-1-2	19.2	19.2	2579	1482	2030
12	IR 62558 – SRN-17-2	25.7	25.7	2487	3062	2774
13	NR 1887-8-1-1-2-2-2	18.7	18.7	3022	2973	2998
14	MASULI	16.9	16.9	3187	2479	2833
15	MAKAWANPUR – 1	25.1	25.1	3847	3431	3639
**16**	**LOCAL CHECK**	**20**	**20**	**2954**	**2478**	**2716**
	F test	**		**	**	
	CV %	6.16		17.8	13.8	
	GRAND MEAN	21.7		2904	2705	
	FLSD 0.05	1.89		732	531	

NOTE : Local check- In NRRP/Hardinath = Ram Bilash; In RARS/Parwanipur = Radha 11

Source: Chaudhary et al., 2005.

Rainfed Intermediate lowland Mother Trial 2004

The ANOVA of agronomic traits and overall preference ranking of rice varieties in lowland mother trials revealed that plant height, days to maturity, tiller hills and grain yield significantly ($p \leq 0.05$) differed for varieties (App.4).

App.4. Table Mean squares of plant height, days to maturity, tiller hill and grain yield measured for COB bred rice varieties in mother trials conducted in rainfed intermediate lowland in main season in 2004.

Source of variation	df	Plant height (cm)	Maturity (Days)	Tillers $hill^{-1}$	Grain yield ($t\ ha^{-1}$)	Preference ranking
Replication	5	211.79	216.45	66.569	2.4256	0.028
Treatment	5	1504.56**	181.65**	4.964*	3.0281**	5.228 ^{ns}
Error	25	31.49	18.49	1.811	0.6307	3.308

* , ** Significantly different at 0.05 and 0.01 probability level
Source: LI-BIRD's mother trials, 2003

We found that Barkhe 3004 and Super 3004 were dwarfer than other varieties but Super 3004 was 8 cm taller than Barkhe 3004 (App.5). Sugandha 2002 had intermediate plant height whereas Barkhe 3015 was the tallest among all. Barkhe 3017 and Masuli had similar plant height. Barkhe 3004, Super 3004, Sugandha 2002 and Masuli matured around 156 days whereas other two varieties i.e. Barkhe 3015 and Barkhe 3017 matured 10-12 days earlier than other varieties.

We found that COB bred varieties were always higher tillering capacity as compared to Standard Check Masuli (App5). Super 3004 and Barkhe 3015 recorded significantly higher tillering capacity than Masuli. Barkhe 3004, Sugandha 2002 and Super 3004 out yielded Masuli but only Super 3004 was found significantly different than Masuli for grain yield. Farmer mostly preferred Masuli and Super 3004 in mother trials but these were non significant with Barkhe 3004 and Sugandha 2002.

App.5. Means of plant height, days to maturity, tiller hill and grain yield measured for COB bred rice varieties in mother trials conducted in rainfed intermediate lowland in main season in 2004.

Varieties	Plant height (cm)	Maturity (Days)	Tillers $hill^{-1}$	Grain Yield ($t\ ha^{-1}$)	Preference ranking
Barkhe 3004	104	157	8	2.9	3.00
Barkhe 3015	145	146	10	1.9	2.33
Barkhe 3017	136	146	8	1.8	3.00
Sugandha 2002	121	156	8	3.0	3.33
Super 3004	112	158	9	3.7	4.33
Masuli	135	155	7	2.7	4.82
FLSD at 0.05 probability level	6.6	5.1	1.6	0.94	2.16

Source: LI-BIRD's mother trials, 2004

We analyzed grain yield and preference ranking of each mother trials in the context of soil fertility and crop managed by the farmers (App. 6). LF2 and LF3 trials were recorded for poor soil fertility and poor crop management. Under poor soil fertility condition in LF2 and LF3 trials, Masuli, Barkhe 3015 and Barkhe 3017 performed extremely poor as compared to Barkhe 3004 and Super 3004. Therefore, we concluded that Super 3004 and Barkhe 3004 were most stable as compared to other varieties including Standard Check Masuli. Another important observation we noticed for Super 3004 was that this variety was always higher yielding except in LF4. Also, Super 3004 performed extremely well under high fertility and good management conditions in LF1 and LF5.

App.6. Grain yield measured and preference ranking scored for COB bred rice varieties in mother trials in rainfed intermediate lowland in main season in 2004.

Variety	LF1		LF2		LF3		LF4		LF5		LF6	
	GY	Score	GY	Score	GY	Score	GY	Score	GY	Score	GY	Score
Barkhe 3004	2.24	4	2.80	1	2.27	2	3.36	1	2.66	4	3.99	6
Barkhe 3015	2.37	1	1.29	2	1.27	3	3.67	5	0.54	1	2.09	2
Barkhe 3017	3.11	3	1.09	3	†0.00	5	3.32	3	1.32	3	2.11	1
Sugandha 2002	2.40	2	3.78	4	2.42	1	2.85	4	3.38	6	3.41	3
Super 3004	4.63	5	4.35	5	2.87	4	3.11	2	2.75	5	4.27	5
Masuli	2.61	6	1.40	6	2.37	6	4.53	6	1.91	1	3.44	4

Score: 1 = Least preferred and 6= Most preferred, † Grain could not be harvested due to lodging
Source: LI-BIRD's mother trials, 2004

The analysis of 87 baby trials revealed that farmers recorded higher grain yield of Barkhe 3004 as compared to Sabitri and Masuli (Figure). We found that 70% farmers reported higher grain yield of Barkhe 3004 compared to Sabitri whereas 17% found its grain yield similar to Sabitri. A similar response of Barkhe 3004 was recorded when this variety was compared to Masuli. We found that 25% farmers found higher grain yield of Barkhe 3004 and 60% experienced similar grain yield as compared to Masuli.

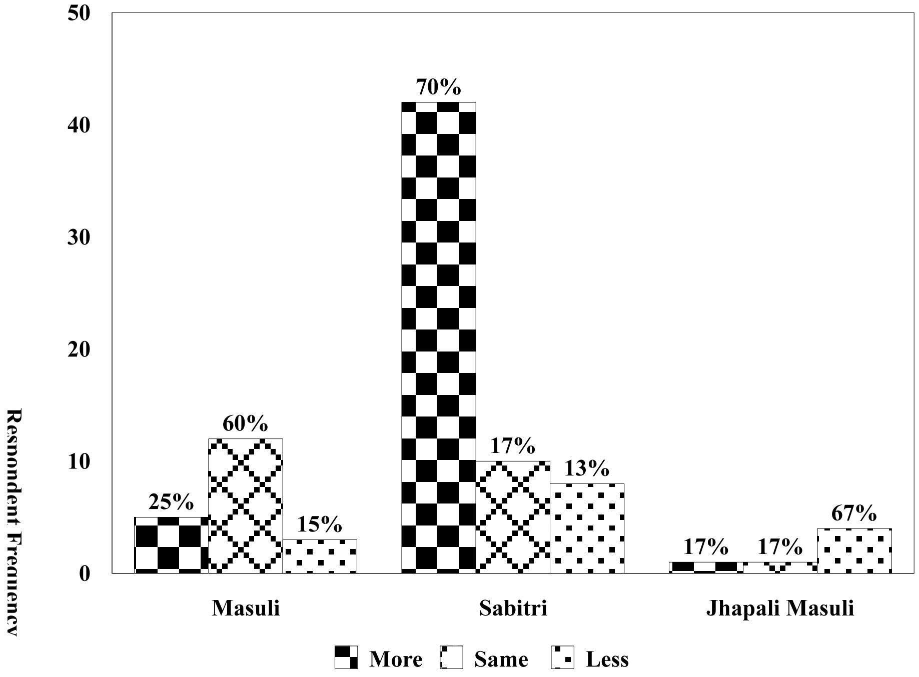

Figure Farmers response on grain yield of Barkhe 3004 compared with popular rice varieties in 87 baby trials in 2004.

Agronomic trials on Fertilizer response in 2004

We analyzed the response of Barkhe 3004 to chemical fertilizer in a replicated trial (App.7). We found that Barkhe 3004 gives higher response to applied fertilizer. The highest grain yield was recorded in 120:30:30 NPK with 18 kg Zn So_4 ha $^{-2}$ fertilizer application which was 54.6% higher than the control (no application of fertilizer) (App.8). We also noticed that the Zinc has least effect to grain yield of Barkhe 3004. This was true from the field observation of many hundred of baby trial where we have never noticed Zinc deficiency in this variety. Therefore we concluded that 120:30:30 NPK ha $^{-2}$ fertilizers are recommended for cultivation of Barkhe 3004 in Nepalese conditions.

App.7. Mean squares of grain yield measured for Barkhe 3004 in response of different combination of Chemical fertilizers in 2004.

Source of variation	df	Grain yield (t ha^{-1})
Replication	5	0.59939
Treatment	5	1.88051**
Error	25	0.09511

Source: LI-BIRD's fertilizer experiment, 2004.

App.8. Means of grain yield measured for Barkhe 3004 in response of different combination of chemical fertilizers evaluated in an experiment during main season rice in Chitwan in 2004.

Fertilizer	Description of fertilizer combinations	Grain yield (t ha^{-1})
0:0:0 NPK	Control (without Chemical Fertilizer)	2.47
25:0:0 NPK	One top dressing at booting stage	2.45
50:30:0 NPK	1/2 N at basal and 1/2 top dressing at booting, others as basal dose	3.40
100:30:30 NPK	50 kg N top dressing two times and rest as basal	3.79

	dose	
120:30:30 NPK	80 kg N top dressed at two times, rest as basal dose with Zinc†	3.82
FLSD at 0.05 probability level		0.47
CV (%)		9.7

† Zinc @ 18 kg Zn SO_4 ha^{-1} applied with 120:30:30 NPK treatment
Source: LI-BIRD's fertilizer experiment, 2004.

We analyzed farmers adoption of Barkhe 3004 in Chitwan in 2004 using household level questionnaires. Farmers growing rice in rainfed lowland and medium land as well as irrigated conditions reported that they have adopted (>60%) this variety (Figure). More interestingly, farmers preferred this variety in irrigated condition because of its higher grain yield, less diseases and insect pest incidence, non-lodging, good milling recovery and higher market price. We have found that Barkhe 3004 has replaced Sabitri in most of the cases.

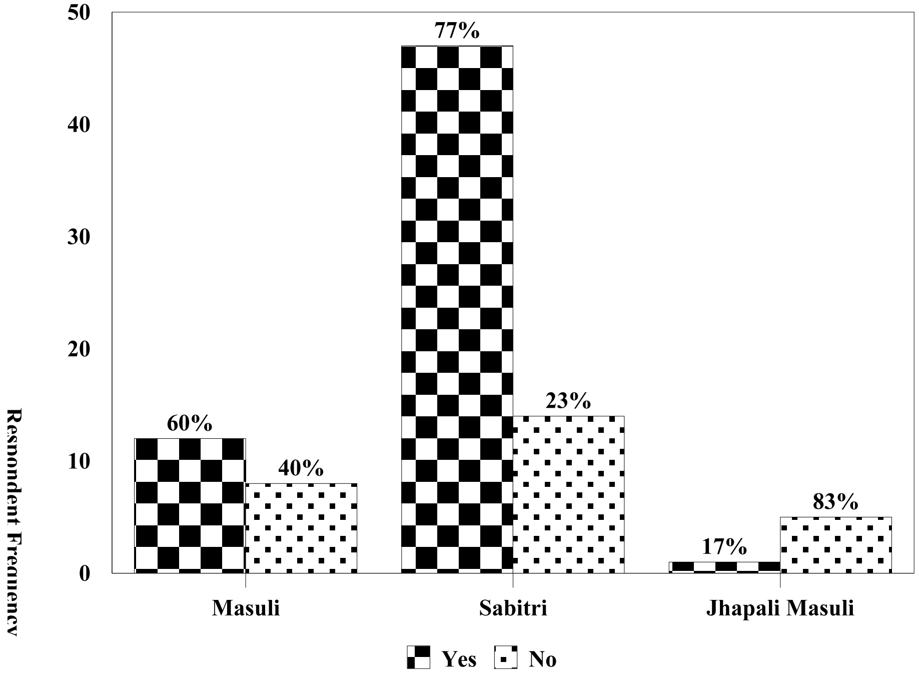

Figure Farmers response on adoption of Barkhe 3004 against popular rice varieties 87 baby trials in 2004.

Baby (on-farm trials), 2004

In 2004, we conducted more than 200 baby trials of Barkhe 3004 in Chitwan and Nawalparasi districts during main season rice. Farmers' responses on various traits of Barkhe 3004 collected using household level questionnaires (HLQs) at the end of season so that farmers could compare its performance during both standing field conditions as well as post harvest quality and market traits. We analyzed 105 baby trials data and summarized the results in figure. We found that 60% of the respondent farmers compared Barkhe 3004 with Sabitri, 20% with Masuli, 6% with Kanchi Masuli (Aus 781) and rest with other varieties. We found that more than 80% farmers experienced less insect pests and diseases in Barkhe 3004 and was less lodging prone. Also we found more than 70% farmers could harvest higher grain and straw yield of Barkhe 3004 as compared to Sabitri, Masuli and other check varieties. Most of

the farmers who had compared Barkhe 3004 reported that they experienced either same of better milling recovery and eating quality which resulted in higher price of milled rice in the market. We recorded that more than 65% farmers have saved the seed of Barkhe 3004 to scale up the area under this variety in 2005.

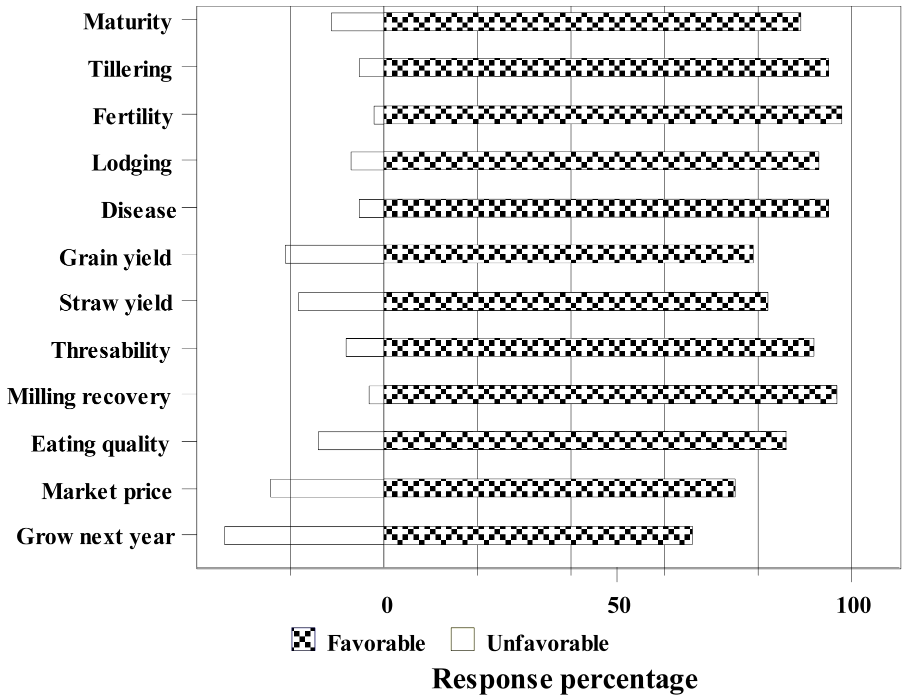

Figure . Farmer response on various traits of Barkhe 3004 measured on 105 baby (on-farm) trials conducted in Chitwan in 2004.

2003 Yield Components

Coordinated Varietal Trial Rainfed lowland Medium (CVTRLM)

App.9. Coordinated Varietal Trial Rainfed lowland Medium (CVTRLM) in 2003.

SN	Varieties	Days to heading		Loc. Mean	Days to maturity		Loc. Mean
		NRRP/H	RARS/P		NRRP/H	RARS/P	
1	NR 1736-4-6-2-1	118	125	122	148	153	151
2	BR 268-4-6-1-4-3-3-5	117	123	120	142	151	147
3	NR 1190-24-4-2-2-2-2-3	112	116	114	136	144	140
4	IR 9925	115	126	121	142	154	148
5	PSBRC 68	115	127	121	142	155	149
6	PSBRC 70	120	130	125	146	159	153
7	Barkhe 2001	102	108	105	121	137	129
**8**	**Barkhe 3004**	**117**	**125**	**121**	**143**	**153**	**148**
9	NR 1748-12-1-1-4-3	126	134	130	150	163	157
10	NR 1893-17-2-3	116	127	122	141	155	148
11	NR 1894-10-3-2-3	122	129	126	149	158	154
12	NR 1887-8-1-1-1-3-3	103	109	106	125	137	131
13	NR 1769-34-2-1-3-1-2	107	123	115	132	150	141
14	***Masuli (St. check)***	***117***	***123***	***120***	***136***	***151***	***144***
15	***Mak-1 (St check)***	***122***	***132***	***127***	***150***	***160***	***155***
16	***Local check***	***122***	***130***	***126***	***153***	***159***	***156***
F test		**	**		**	**	
CV%		1.29	1.48		2.18	1.31	
Grand Mean		115	124		141	152	
FLSD0.05		2.1	2.5		4.2	2.8	

Source: Choudhary *et al.*, 2004.

App.10. Coordinated Varietal Trial Rainfed lowland Medium (CVTRLM) in 2003.

SN	Varieties	Plant height (cm)		Loc Mean	Panicle m^{-2}		Loc Mean	Grains $panicle^{-1}$
		**NRRP/H**	**RARS/P**		**NRRP/H**	**RARS/P**		**NRRP/H**
1	NR 1736-4-6-2-1	121.9	117	119.4	235	231	233	193
2	BR 268-4-6-1-4-3-3-5	139.4	120	129.7	202	236	219	234
3	NR 1190-24-4-2-2-2-2-3	124.9	126	125.4	217	225	221	199
4	IR 9925	83.3	89	86.1	241	208	225	136
5	PSBRC 68	101.5	109	105.2	214	234	224	138
6	PSBRC 70	98.3	101	99.6	257	233	245	122
7	Barkhe 2001	98.5	108	103.2	234	294	264	113
**8**	**Barkhe 3004**	**56**	**93**	**89.5**	**257**	**271**	**264**	**113**
9	NR 1748-12-1-1-4-3	128.6	121	124.8	227	246	265	210
10	NR 1893-17-2-3	86.2	88	87.1	239	252	246	182
11	NR 1894-10-3-2-3	108.9	105	106.9	250	263	257	154
12	NR 1887-8-1-1-1-3-3	85	91	88	265	324	299	139
13	NR 1769-34-2-1-3-1-2	86.2	98	92.1	255	241	248	106
14	***Masuli (St. check)***	***125.5***	***115***	***120.2***	***271***	***272***	***272***	***203***
15	***Mak-1***	***89.1***	***93***	***91.10***	***279***	***263***	***271***	***123***
16	***Local check***	***134.3***	***126***	***130.1***	***320***	***279***	***310***	***115***
F test		**	**		**	*		
CV%		5.62	4.49		15	13.4		17.7
Grand Mean		106	107		247	254		154.8
FLSD 0.05		8.4	6.7		52	48		39

Source: Choudhary *et al.*, 2004.

App.11. Coordinated Varietal Trial Rainfed lowland Medium (CVTRLM) in 2003.

SN	VARIETIES	1000 Grain wt. (gm)	L/B ratio (mm)	Grain yield Kg ha^{-1}		Loc. Mean
		NRRP/H	NRRP/H	NRRP/H	RARS/P	
1	NR 1736-4-6-2-1	23.6	2.4	4325	4412	4368
2	BR 268-4-6-1-4-3-3-5	19.4	2.0	4288	4622	4455
3	NR 1190-24-4-2-2-2-2-3	23.3	2.38	4741	4443	4592
4	IR 9925	28.8	2.57	3268	3844	3556
5	PSBRC 68	29.5	3.05	3815	4436	4125
6	PSBRC 70	31.7	3.15	3994	3741	3843
7	Barkhe 2001	20.8	3.13	2729	3689	3209
**8**	**Barkhe 3004**	**23.9**	**2.99**	**3616**	**4177**	**3896**
9	NR 1748-12-1-1-4-3	21	2.66	2817	2122	2470
10	NR 1893-17-2-3	21.7	2.48	4113	4322	4227
11	NR 1894-10-3-2-3	27.9	2.42	4313	4439	4376
12	NR 1887-8-1-1-1-3-3	18.7	2.79	3168	3651	3410
13	NR 1769-34-2-1-3-1-2	24.1	3.1	2301	3232	2767
14	***Masuli (St. check)***	***17.7***	***2.41***	***3798***	***3468***	***3633***
15	***Mak-1***	***26.7***	***2.24***	***3314***	***4169***	***3742***
16	***Local check)***	***18.2***	***2.96***	***2188***	***3172***	***2680***
F test		**	**	**	**	
CV%		6.4	5.8	16.7	12.7	
Grand Mean		23.5	2.6	3350	3871	
FLSD 0.05		2.1	0.2	838	699	

Source: Choudhary *et al.*, 2004.

<u>Rainfed Intermediate lowland Mother Trial 2003</u>

App.12. Mean squares of plant height, days to maturity, tiller hill and grain yield measured for rice varieties in mother trials conducted in lowland in main season in 2003.

Source of variance	df	Plant height cm	Days to maturity	Tiller hill^{-1}	Grain yield t ha^{-1}	Preference ranking
Replication	4	659.94	410.25	14.50	4.604	0.00
Variety	5	658.42***	4.700 ^{ns}	2.54 ^{ns}	0.087 ^{ns}	1.82 ^{ns}
Error	20	28.26	2.750	1.34	0.405	3.92

Source: LI-BIRD's mother trials, 2003

App.13. Means of plant height, days to maturity, tiller hill and grain yield measured for rice varieties in mother trials lowland in main season in 2003.

**Variety**	**Plant height (cm)**	**Maturity (days)**	**Tiller hill^{-1}**	**Grain yield (t ha^{-1})**	**Preference ranking**
Barkhe 3004	97.6	158.6	8.0	3.17	4.2
Barkhe 3010	103.2	156.6	9.2	2.92	3.6
Barkhe 3014	100.8	159.2	8.4	3.06	2.6
Barkhe 3012	101.4	158.6	8.8	3.06	3.6
Barkhe 3013	100.2	159.2	9.2	3.25	3.0
Masuli	128.4	158.8	7.4	3.26	4.0
FLSD at 0.05 prob.	7.01	2.18	1.5	0.84	2.61
CV%	5.00	1.00	13.6	20.4	56

Source: LI-BIRD's mother trials, 2003

App.14. Preference ranking scores (S) and grain yield (GY t ha^{-1}) measured for PPB bred varieties in mother trials in lowland production domain in 2003.

Variety	LF1		LF2		LF3		LF4		LF5	
	**S**	**GY**	**S**	**GY**	**S**	**GY**	**S**	**GY**	**S**	**GY**
B 3004	4	2.94	6	4.97	5	3.65	3	2.50	3	1.79
B 3010	2	3.02	5	4.07	1	2.66	5	3.02	5	1.78
B 3012	3	3.90	4	4.95	4	2.83	1	1.85	1	1.75
B 3013	6	3.20	3	3.66	3	4.19	2	2.54	4	1.72
B 3014	5	3.37	2	4.20	2	4.00	4	2.75	2	1.92
Mansuli	1	2.60	1	3.26	6	4.80	6	2.75	6	2.89

Source: LI-BIRD's mother trials, 2003, LF= Farmers code

2004 Pest (Blast and Bacterial Leaf Blight)

National Rice Disease Nurseries (NRRP/Hardinath)

App.15. National Rice Disease Nurseries for blast and bacterial leaf blight, 2004.

SN	DESIGNATION	SOURCE	NRRP/H	NRRP/H
			BLAST	BLB
1	*R. Check*	.	*3*	*3*
2	*S. Check*	.	*7*	*9*
3	IAASR-16	CVT	0	7
4	CNTLR85085-78-1-1-1	,,	NG	7
5	RATO BASMATI	IET-A	0	5
6	PUSHA SUGANDHA-2	,,	0	9
7	Gautam	,,	PG	9
8	Sugandha 2002	CVT-		3
9	NR 268-4-6-1-4-3-3-5	,,	2	3
10	NR 1910-10-3-2	IET-	0	7
11	NR 1916-12-1-3	IET	0	3
12	IR 55435-5	CVT-UP	NG	7
13	Vandana	,,	0	5
14	CAN 4196	IET-UP	0	7
15	NR 1824-21-1-1-2-1-2	FFT	0	5
16	NR 1485-3-3-2-3-2-1	FFTRLM	0	3
17	BR 4962-12-4	FFT RLM	NG	3
18	BPI 3-2	,,	0	5
19	OR-367	,,	1	1
20	CNTLR 85033-9-3-1-1	FFT-A	0	7
21	GAN-WAN-XIAN 22	,,	0	3
22	PUSA-834	,,	0	3
23	Khumal-4	,,	0	3
24	Khumal-11	,,	0	1
25	*R.Check*	*,,*	*0*	*5*
26	*S.Check*	.	*9*	*9*
27	Barkhe 3017	LI-BIRD	2	3
28	Barkhe 2044	,,	0	3
29	Barkhe 2001 (Bulk)	,,	0	3
30	Barkhe 2045	,,	3	5
31	Barkhe 2024	,,	0	5
**32**	**Barkhe 3004**	**,,**	**0**	**3**
33	Barkhe 1027	,,	0	5
34	*R.Check*	.	*0*	*1*
35	*S.Check*	.	*9*	*9*

Source: Bedananda *et al,* 2005.
NRRP/H= National Rice Research Program, Hardinath; NG=Not germinated; PG=Poor germination

2003 Pest (Blast and Bacterial Leaf Blight)

App.16. National Rice Disease Nurseries for blast, 2003.

EN	DESIGNATION	LEAF BLAST SCORE (0-9)			
		NRRP/H	NORP/N	PPD/K	RARS/T
24	CNTLR85085-78-1-1-1	0	1	1	0
36	Rato Basmati	0	1	2	1
129	Ghaiya 2	0	1	2	0
155	Pant-10	-	1	2	0
156	Judi-102	0	1	-	0
157	Judi-565	-	1	1	0
158	Judi-566	-	1	2	0
166	IR 36	-	1	2	0
167	Judi 572	0	2	2	0
170	BPI 3-2	-	1	2	0
210	Barkhe-2001	0	1	1	0
211	Barkhe-2014	0	1	1	1
**226**	**Barkhe-3004**	**0**	**1**	**2**	**0**
228	Barkhe-3010	0	1	2	0
229	Barkhe-3012	0	1	1	1
230	Barkhe-3013	-	1	2	0
231	Barkhe-3014	-	1	1	0
232	Sugandha-1	0	1	1	0
233	Sugandha-2002	-	1	2	1
262	IR57893-10	-	2	2	0
	Laxmi (Resistant Check)	*1*			
	Masuli (Susceptible Check)	*9*			

Source: Chaudhary *et al.*, 2004.

App.17. Response of Barkhe 3004, Judi 572 and Barkhe 2014 to major rice diseases in on-farm trials assesses by Plant Pathologist in 2003.

Variety	Varietal Response to disease					
	Leaf Blast	Neck blast	Bacterial Leaf blight	Brown Spot	Sheath Blight	Glume Discoloration
Judi 572	R	R	R	-	MR	MS
Barkhe 2014	R	R	R	R	MR	R
Barkhe 3004	R	R	R	R	MR	R

R= Resistant, MR= Moderately Resistant, MS= Moderately Susceptible

App.18. Response of Barkhe 3004 and Masuli to rice diseases observed in mother trials in on-farm trials in Chitwan in 2003.

Variety/Lines	Farmer' Name	Village	GS	**LB**	**NB**	**BS**	NBLS	**ShB**	ShR	FSm	GD	**BLB**	LS	RSV	MB	**Borer**	LF
Masuli	Nirmala Aryal	Ujalnagar	6	0	-	3	5	0	0	-	1	1	0	0	2	0	0
**Barkhe 3004**	**Nirmala Aryal**	**Ujalnagar**	**7**	**0**	**-**	**1**	**5**	**0**	**0**	**-**	**-**	**0**	**0**	**1**	**3**	**0**	**0**
Masuli	Hari Pandit	Champanagar	7	0	-	0	5	0	0	0	1	0	0	0	1	0	0
**Barkhe 3004**	**Hari Pandit**	**Champanagar**	**8**	**0**	**0**	**1**	**5**	**0**	**0**	**0**	**3**	**0**	**0**	**1**	**1**	**0**	**0**
Masuli	Surya Kumari Raut	Amarbasti	8	0	0	0	5	0	0	0	5	3	0	0	0	1	1
**Barkhe3004**	**Sarswati Ghimire**	**Krishnamandir**	**4**	**0**	**-**	**0**	**5**	**0**	**-**	**-**	**-**	**5**	**0**	**0**	**0**	**0**	**0**
Masuli	Sarswati Ghimire	Krishnamandir	5	0	-	0	1	0	-	-_	-_	3	0	0	0	0	1

-Disease was not scored, GS=Grwoth Stage, **LB= Leaf Blast, NB= Neck Blast,** BS+ Brwon Spot, NBLS=Narrow Brown leaf Spot, **ShB= Sheath Blight,** ShR= Sheath Rot, FSm= False Smut, GD=Glume Discoloration, **BLB= Bacterial Leaf Blight,** RSV=Ragged Stunt Virus, LS=Leaf Scald, LF= Leaf folder.

2002 Pest (Blast and Bacterial Leaf Blight)

App.19. National Rice Disease Nursery for blast, 2002.

EN	DESIGNATION	LEAF BLAST SCORE			
		NRRP/H	RARS/L	NMRP/R	RARS/T
4	BG 1442	2	0	0	1
44	CNTLR 85085-78-1-1-1	1	0	0	0
207	Barkhe 1027	0	0	-	0
**217**	**Barkhe 3004**	**1**	**0**	**-**	**0**
218	Barkhe 3005	1	2	0	1
221	Barkhe 3006	2	0	-	2
222	Barkhe 3007	1	2	0	0
223	Barkhe 3008	1	1	0	1
242	NR 1748-12-1-1-4-3-4-3-2-1-2-3	2	2	0	0
244	NR 1863-63-1-1-1-1-2-1	1	0	0	0
245	NR 1892-12-2-6-1-3-1	1	0	0	0
246	NR 1892-12-2-1-3-2	1	0	0	0
250	NR 1898-5-1-2-1-1-2	2	0	0	0
255	IR 68851-27-1-B-1-2-1	2	2	0	0
	**Laxmi (Resistant Check)**	1	1	1	1
	**Shankharika (Susceptible Check)**	9	9	9	9

Source: Chaudhary *et al.*, 2003.

App.20. National Rice Disease Nursery for bacterial leaf blight, 2002.

EN	DESIGNATION	BACTERIAL LEAF BLIGHT SCORE (0-9)		
		NRRP, HARDINATH	RARS, PARWANIPUR	NWRP, BHAIRAHAWA
9	IR 55435-05	3	3	3
11	IR 70219-35-2-1-1-1-3	3	0	3
56	PSBRC 2	3	0	3
58	RHS379-25CX-1CX-2CX-OZA	3	0	3
61	IR 6179-138-1-3-2-2	3	0	3
127	Judi 102	3	1	3
138	Judi 503	3	1	3
**217**	**Barkhe 3004**	**3**	**0**	**3**
218	Barkhe 3005	3	0	3
233	PSBRC 70	3	0	3
241	NR 1748-12-1-1-4-3-4-3-2-1-2-1	3	0	3
247	NR 1893-17-2-3-1-1-2	3	0	3
248	NR 1894-10-3-2-3-1-1	3	0	3
249	NR 1894-10-3-2-3-1-6-3	3	0	3
250	NR 1898-5-1-2-1-1-2	3	0	3
251	NR 1855-1-1-2-1-1-1	3	0	3
253	NR 1887-8-1-2-1-1-1	3	0	3
257	NR 1891-58-3-2-2-3-1-2	3	0	3
258	NR 1891-58-3-2-2-3-1-3	3	0	3
	Janaki (Resistant Check)	1	1	1
	TN1 (Susceptible Ckheck)	9	9	9

Source: Chaudhary *et al.*, 2004.

References

B. Chaudhary, M. Yadav, RB. Yadaw, DK. Choudhary, PCP. Chaurasiya, RN. Chaudhary, BN. Mahato and DN. Sah. 2004a. Evaluation of rice genotypes for resistance to blast disease. Paper presented in 24th Summer Crop Workshop, NARC, Kathmandu.

B. Chaudhary, M. Yadav, T. Akhtar, RB. Yadaw, DB. Gharti and D. Bhandari. 2004b Evaluation of rice genotypes for resistance to bacterial blight. Paper presented in 24th Summer Crop Workshop, NARC, Kathmandu.

S Gyawali, K D Joshi and J R Witcombe. 2002. Participatory plant breeding in rice in low altitude production system in Nepal. In: Witcombe J R, Parr L B, Atlin G N (eds.) (2002) Breeding rainfed rice for drought prone environments: Integrating conventional and participatory plant breeding in South and Southeast Asia: Proceedings of a DFID Plant Sciences Research Programme/IRRI Conference, 12-15 March 2002, IRRI, Los Banos, Laguna, Philippines. Department for International Development (DFID) Plant Sciences Research programme (PSP), Center for Arid Zone Studies (CAZS) and International Rice Research Institute (IRRI), Bangor and Manila.

Central Bureau of Statistics (CBS). 2003. Statistical Year Book of Nepal. National Planning Commission Secretariat, HMG/N.

FAO. 2004. FAO Statistics Nepal. [http://www.riceweb.org/countries/nepal.htm]
