# Supplementary material for: Plant breeding can be made more efficient by having fewer, better crosses
Source: BMC Plant Biol. 2013 Feb 7;13:22. doi: 10.1186/1471-2229-13-22 (PMC3621587; doi:10.1186/1471-2229-13-22)
Supplement: Additional file 5 — New Upland Rice Varieties for India. Rainfed Agriculture Impact Study No. 1. Monitoring Impact Assessment and Learning Component (MIL) of the Research into Use Programme (RiU); 2008. [file 1471-2229-13-22-S5.pdf]

Impact of New Upland Rice Varieties in Eastern India from Client-Oriented Breeding: Evidence from Whole Village Surveys

A. Mottram

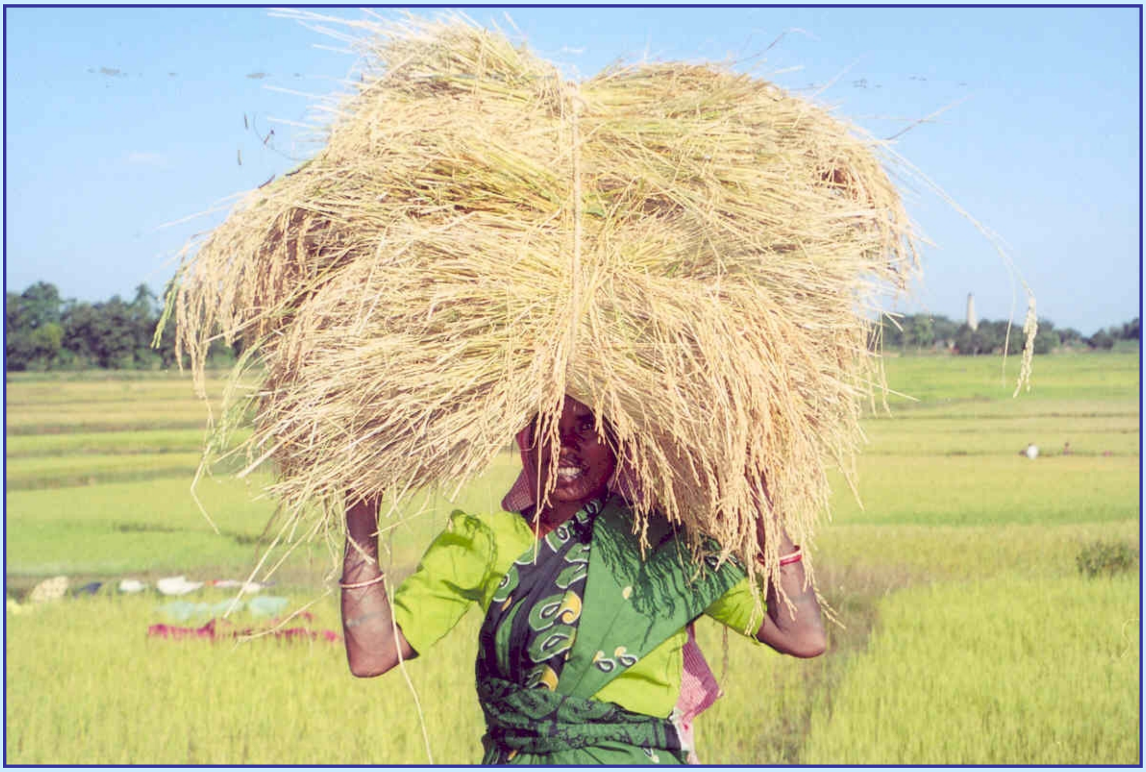

PRIFYSGOL CYMRU
UNIVERSITY OF WALES
BANGOR
1884
CATC | Canolfan Astudiaethau Tir Cras
CAZS | Centre for Arid Zone Studies

Impact of New Upland Rice Varieties in Eastern India from Client-Oriented Breeding:
Evidence from Whole Village Surveys

A. Mottram

Centre for Arid Zone Studies (CAZS), University of Wales, Bangor, UK.

Summary

A collaborative client-oriented breeding programme between the Gramin Vikas Trust (GVT), Birsa Agricultural University (BAU) and Centre for Arid Zone Studies (CAZS) resulted in the production of two new upland rice varieties, Ashoka 228 and Ashoka 200F. Since 2001, these varieties have been distributed to a number of farmers in Western and Eastern India.

Whole village surveys of a sample of the villages in Eastern India, that had been supplied with seed by GVT, showed adoption levels of up to 63% of the current upland rice production areas within three years, with a potential to increase further into other fallow areas of the upland. Farmers are adopting the Ashoka varieties because they are high yielding, mature early, are drought resistant and have a good taste and short cooking time. Farmers, as well as increasing the area under these varieties on their own farms, sold or exchanged seed to other farmers

Cultivation of Ashoka has had a large impact on farmers' livelihoods particularly in terms of food security where often this has been increased by 1-2 months, in some cases making grain-deficit households self-sufficient.

This survey was done to triangulate the results of two previous surveys. It confirmed the results that farmers like the new varieties, adopt them on a high proportion of their upland, and distribute the seed to others. The proportion of upland rice areas devoted to the new varieties was uniformly high whatever the method of assessment employed.

Introduction

Triangulation of impact assessments

This report is an independent assessment of the impact of an upland rice breeding programme in eastern India that used participatory methods to improve its client-orientation. The assessment was made in October 2004 by a CAZS member of staff who was external both to all of the projects up to and including the current one 'R8099 '*Participatory plant breeding in rice and maize in eastern India - its scaling up and evaluation of products and methods*' and external to the Plant Sciences Research Programme that had supported these projects. The purpose of this assessment was to use a different method to those of previous assessments in order to triangulate the results. Two previous surveys had been conducted at the household level where many farmers were interviewed who had received seed from GVT. In contrast, in this assessment, the survey was conducted at a village level. Land maps were used to quantify the village adoption rates and a number of participatory rural appraisal methods were used with groups of farmers to assess the reasons for adopting the new upland rice varieties and their effects on livelihoods.

The two previous surveys were made in 2002 and 2004. In the first, approximately 15% of the 1000 households that received seed from GVT (56 in Jharkhand, 29 in Orissa, and 41 in W. Bengal) were surveyed (Bourai *et al.*, 2003)[1]. Each farmer completed a questionnaire, providing information on adoption rates from 2001 to 2002, varietal traits and seed transactions. In 2004, this process was repeated for later years in a second survey with 150 households. Virk *et al*. (2004)[2] includes a summary of the results from 36 of those households (7 in Jharkhand, 15 in West Bengal and 14 in Orissa).

---

[1] Bourai, V.A., Choudary, A., Misra, M. (2003). Participatory crop improvement in Eastern India: A first impact assessment. Plant Sciences Research Programme Annual Report for 2002.

[2] Virk, D.S., Bourai, V.A., Choudary, A., Misra, M. & Witcombe, J.R. (2004). Participatory crop improvement in Eastern India: An impact assessment. Plant Sciences Research Programme Annual Report for 2003.

Background

The proportion of the population living below the poverty line in India is greatest in the eastern States. In Jharkhand, Orissa and West Bengal nearly half the rural population is below the poverty line (>26 million people). Although rice accounts for approximately half the total cropped area, only 20% in West Bengal, and 40% in Jharkhand and Orissa is irrigated. The poorest farmers grow upland rainfed rice, and have benefited little from high-yielding ‘green revolution varieties’, most are limited to growing local low-yielding varieties (Bourai *et al*., 2003).

A collaborative project, which began in 1997, was established for a breeding programme in upland rice, funded by the DFID Plant Sciences Research Programme, DFID India and the Government of India. This included three main partners:

1. Gramin Vikas Trust (GVT) Ranchi, India – a non-governmental organisation (NGO) working in six states of India currently active through two projects: Eastern India Rainfed Farming Project (EIRFP) and Western India Rainfed Farming Project (WIRFP). The EIRFP covers nine districts within Orissa, Jharkhand and West Bengal States.
2. Birsa Agricultural University (BAU), Ranchi, India – a state agricultural university in Ranchi responsible for agricultural research in Jharkhand state.
3. Centre for Arid Zone Studies (CAZS), Bangor, UK – a semi-autonomous, self-funding centre within the University of Wales, Bangor.

The plant-breeding project used participatory approaches to improve the client orientation of the breeding programme. This approach is called participatory plant breeding (PPB) or client-oriented breeding (COB). It used simple breeding methods in a carefully selected cross of Kalinga III (early maturing, high grain and fodder yield, fine grain, good cooking quality) and IR64 (high-quality, high-yielding irrigated variety). This involved a high degree of farmer participation either as active collaborators (they selected among the offspring of the cross in their own fields) or in a more consultative role (brought to the research station and asked to select among lines grown by scientists). Ashoka 200F and Ashoka 228 were identified by the Birsa Agricultural University in 2001 and released by the State Varietal Release Committee (SVRC) in 2003 (with GVT and CAZS) for Jharkhand as BVD 109 and BVD 110.

Since 2001 GVT's self-help groups in Orissa have been producing seed of Ashoka 200F and Ashoka 228, which has been distributed to many farmers, government organisations (GOs) and non-government organisations (NGOs) through the WIRFP and EIRFP.

Methods

In order to establish the uptake and impact of Ashoka varieties distributed to GVT project clusters and villages[3] a total of 17 villages/hamlets were selected from one district in each of the three states within EIRFP; Purulia in West Bengal, Ranchi in Jharkhand and Mayurbhanj in Orissa (Figure 1 and Table 1). Where possible hamlets/villages were selected that had been provided with seed of the Ashoka varieties for at least two seasons. Information on the percentage of upland area cultivated with Ashoka, reasons for adoption, farmer-to-farmer seed transactions and impact on farmers' livelihoods was collated with farmers using a number of participatory rural appraisal methods (PRAs) including; crop cultivation maps using land maps constructed in the early 1900s (Appendix 1), preference ranking (Figure 2), impact diagrams and group discussions.

[3] GVT works with self-help groups in clusters of villages (approximately 4 villages per cluster). Clusters are initially selected based on meeting certain criteria that place the villages in a low poverty status. GVT community organisers (COs) then establish rapport building with the community, conduct Participatory Rural Appraisals (PRAs) to understand the village situation, and facilitate group formations. Project activities e.g. Ashoka seed distribution, are then implemented through the groups and a group selected 'Janker' from the community who is a facilitator, trainer, disseminator and innovator.

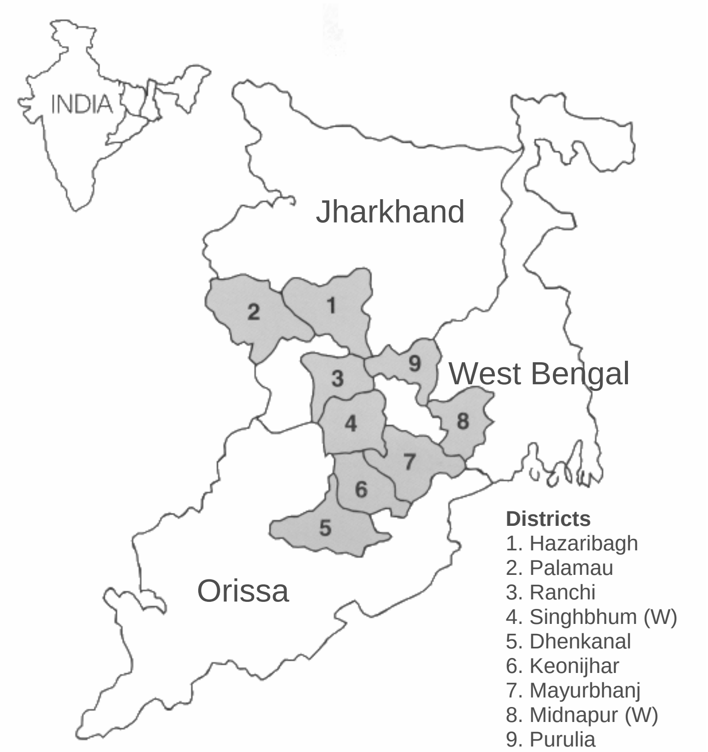

Figure 1. Map showing the GVT operational districts in Eastern India (districts 1 – 3 in Jharkhand; 4 – 7 in Orissa and 8 – 9 in West Bengal). Source: adapted from GVT Eastern India rainfed farming project information brochure.

Table 1. Villages selected for the impact assessment studies.

District/ State	Cluster	Villages selected
Purulia, West Bengal	Mayur Jangalpur	Asanbori & Dumkadih
	Bahukata Ranipur	Bogdisha
		Ranipur and Koldhi
Ranchi, Jharkhand	Maharu	Dighiya
	Muramu	Hulsi
	Rajhir	Jhalenria & Bhilwasie hamlets, Jamueria
	Okhargarha	New Masjid & Mahudar hamlets, Madanpur
	Okhargarha	Ohdar hamlet, Okhargarha
	Uruguttu	Uruguttu
Mayurbhanj,Orissa	Bodabilla Kalajhinel	Bodabilla
	Udali	Kalajhinel & Diblabeda
		Udali

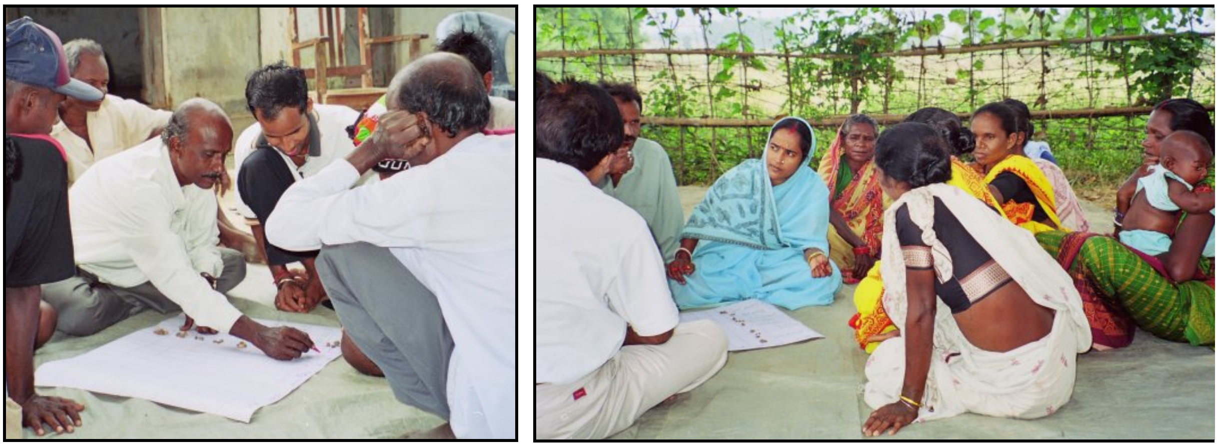

Figure 2. Male group of farmers completing preference rankings of local and Ashoka upland rice varieties, and female group discussing preference ranking with extension agents, Kalajhinel, Mayurbhanj district, Orissa, October 2004.

Seed distribution

Formal seed distribution by GVT

There is currently no commercial seed production of Ashoka varieties therefore GVT self-help groups in Orissa state produce Ashoka 228 and Ashoka 200F seed during the off-season (*Rabi*), for distribution to GVT project villages during the main season (*Kharif*). GVT procure most of this seed and distribute it to villages and other agencies, NGOs and State Departments of Agriculture. Since 2002, about 168 tonnes of Ashoka seed has been procured and distributed to farmers (Table 2).

Table 2. Seed distribution (tonnes) by GVT during kharif 2002-2004.

		2002		2003		2004	
State	Group type	Ashoka 228	Ashoka 200F	Ashoka 228	Ashoka 200F	Ashoka 228	Ashoka 200F
Jharkhand	GVT clusters	8.0	4.0	8.0	1.1	4.7	4.7
	Other†	2.1	2.4	5.9	2.0	4.8	4.8
	Sub-total	*10.1*	*6.4*	*13.9*	*3.0*	*9.5*	*9.5*
Orissa	GVT clusters	8.0	4.0	5.5	3.2	n.d.	n.d.
	Other†	1.5	0.9	1.7	0.9	n.d.	n.d.
	Sub-total	*9.5*	*4.9*	*7.2*	*4.1*	*18.4*	*27.8*
West Bengal	GVT clusters	8.0	4.0	6.0	3.0	7.4	7.5
	Other†	1.5	0.9	0	0	2.6	2.5
	Sub-total	*9.5*	*4.9*	*6.0*	*3.0*	*10.0*	*10.0*
	Total	29.1	16.2	27.1	10.1	37.9	47.3

†NGOs, GOs and private companies; n.d. = no data available

Farmer to farmer seed distribution

In addition to the formal seed distributed by GVT, farmers have distributed seed of Ashoka to each other. Village surveys showed that the amount of seed distributed varied from farmer to farmer depending on the area cultivated and the amount of seed harvested. However, unless farmers had only cultivated a very small area and harvested less than 50 kg, each farmer or group distributed seed to at least one other person. They distributed between 5 kg to 60 kg and between 1 to 9 farmers, usually family members. The distances ranged from distributions within the same village to villages up to 30 km away including non-GVT project villages. Farmers either distributed this seed freely, sold it or exchanged it for other seed. Some farmers, such as Mr Mahoto of Asanbori village, also sold seed in the market or to seed traders (Figure 3). He is typical of farmers who had been cultivating Ashoka for at least two seasons and who had, therefore, acquired a larger seed reserve.

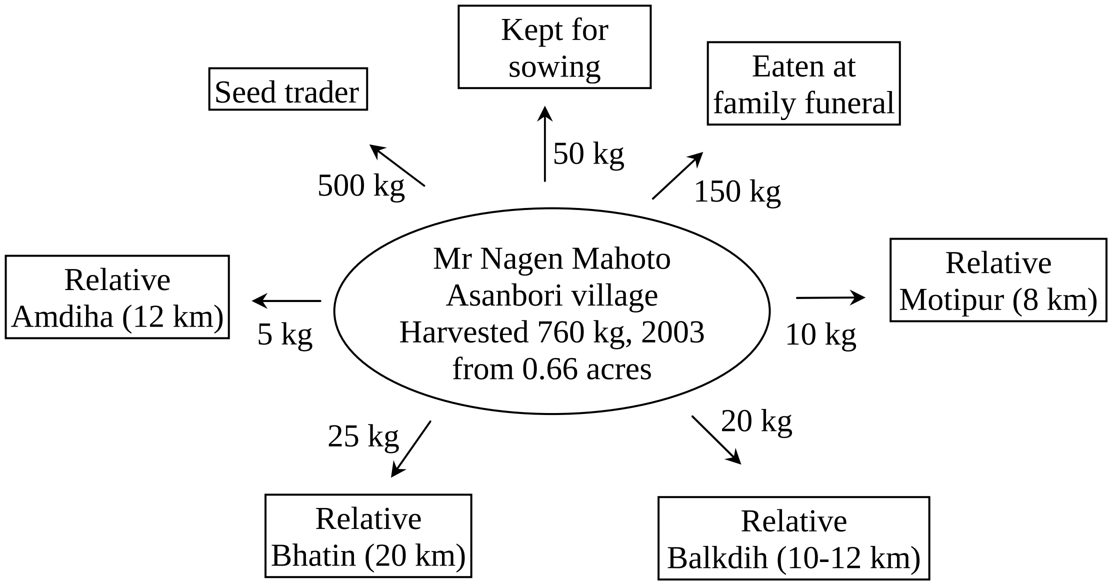

Figure 3. Example of farmer-to-farmer seed distribution. Mr Mahoto, Asanbori village, Purulia district, West Bengal, October 2004.

Adoption

In 10 of the villages surveyed, where detailed data was available for the areas of upland, medium and lowland within the village, the upland area accounted for a mean

of 41±5.6%[4] of the total cultivable area (not including homestead land). Generally this area is cultivated with local rice varieties, which are low yielding, other crops such as blackgram (*Vigna mungo*), groundnut (*Arachis hypogaea*), pigeon pea (*Cajanus cajan*), horsegram (*Dolichos biflorus*) and some vegetables, or is left fallow. Ashoka 228 and 200F have been bred for the purpose of being cultivated in this less fertile upland area.

Adoption rates of Ashoka 228 and Ashoka 200F are calculated for 11 villages where seed had been distributed for at least two years. The adoption rates vary from 8 to 63% of the upland rice area (Figure 4 and see appendix 2 for further details on upland areas). In just two to three years since the introduction of Ashoka seed, on average 30±6% of the upland rice previously cultivated, has been replaced with Ashoka 228 or Ashoka 200F. The variation in adoption was largely dependent on the amount and number of farmers that seed had been distributed to by GVT in previous years.

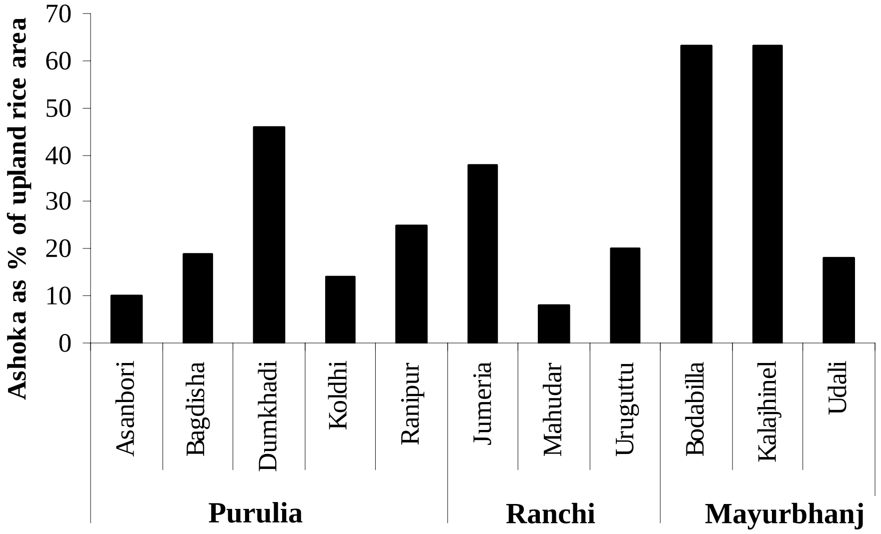

Figure 4. Adoption levels of Ashoka expressed as percentage of upland rice area, in kharif season 2004 from a survey in October 2004.

Adoption can also be considered as the percentage of total upland area rather than upland rice area (Figure 5), and this gives an estimate of the potential for increasing cultivation of the Ashoka varieties in the future. The cultivation of Ashoka can increase much further, if seed is available, both by replacing other rice varieties and,

---

[4] Mean ± standard error

as some farmers had already done, by extending into fallow areas that are generally not utilised due to a lack of suitable varieties.

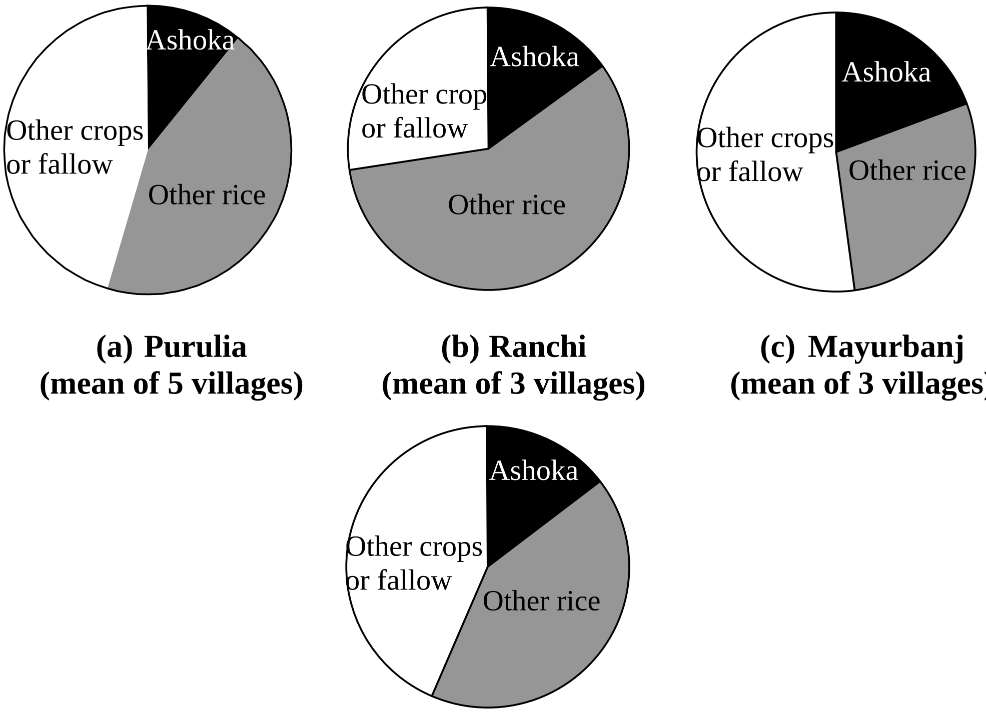

(a) Purulia (mean of 5 villages) **(b) Ranchi (mean of 3 villages)** **(c) Mayurbanj (mean of 3 villages)**

(d) Mean of all districts

Figure 5. Upland cultivation of Ashoka, other rice varieties and other crops in each of the three districts and a mean of them in kharif 2004.

Reasons for adoption

Preference ranking was conducted with groups of farmers in 8 villages. In all cases the Ashoka varieties were preferred for most traits and resulting in much higher overall scores compared to other upland rice varieties. A typical example is shown for Bagdisha village, Purulia (Table 3).

Table 3. Preference ranking by farmers in Bagdisha village, Purulia, October 2004.

Rice variety	Yield	Time to maturity	Drought resist.	Taste	Cooking time	Straw quality	Market price	Disease resist.	Total score
Simulkundi	5	4	3	3	3	5	5	6	34
Chauria	5	4	3	3	3	5	5	4	32
Karna	4	3	2	5	3	5	5	4	31
Halsanga	4	4	4	6	3	6	5	3	35
Tusku	3	6	4	5	4	4	4	3	33
Asu	7	7	7	6	5	3	5	6	46
Dahijeera	4	4	6	4	5	5	5	5	38
Ashoka	8	8	11	8	14	7	6	9	71

* Farmers distributed 40 stones between the varieties for each of the points they considered to be important for rice; the highest number was given to the best variety.

Farmers have adopted Ashoka 228 and 200F so readily because they are high yielding, mature early (90 days) (Figure 6), are drought resistant, have a good taste and short cooking time and good straw quality particularly for fodder. Due to these factors they also demand a high market price compared to local varieties (Rs 4 – 4.5 kg^{-1} compared with Rs 3.5 – 3.8 kg^{-1} for local varieties).

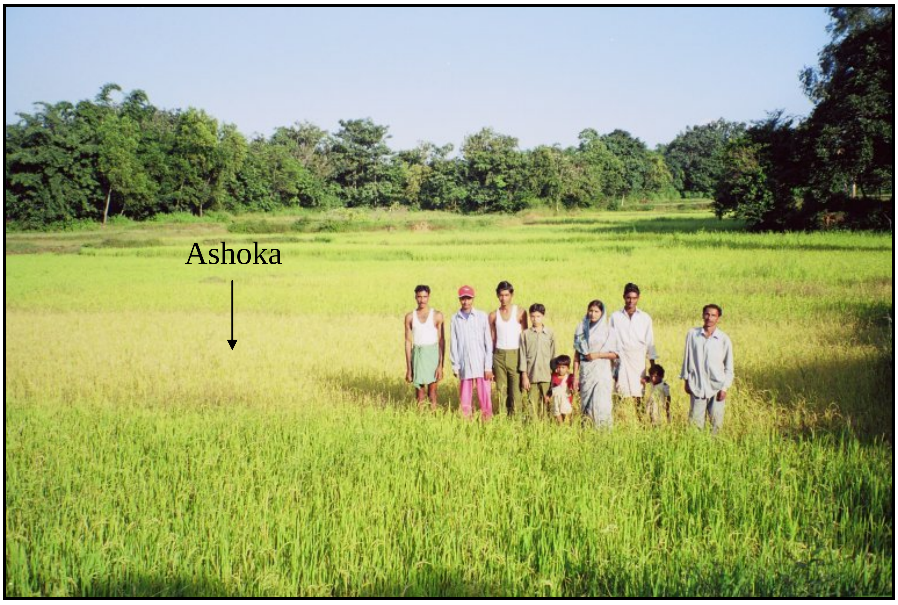

Figure 6. Ashoka maturing earlier than surrounding upland rice varieties in Sanga village, Jharkhand, October 2004.

Effect on livelihoods

Farmers reported that the benefits of cultivating Ashoka have had a major impact on their livelihoods (Figure 7). They said that food is available in years of poor rainfall and during the lean periods of the year, and straw is available for fodder earlier in the season. Additional cash from the sale of surplus grain, or because grain no longer has to be purchased for household needs, can be used for various purposes such as children's education, food and clothes. However, the most important impact, reported by a majority of farmers, was that cultivation of the Ashoka varieties results in increased household food security – on average by 1-2 months, in some cases, enabling grain-deficit households to become self-sufficient.

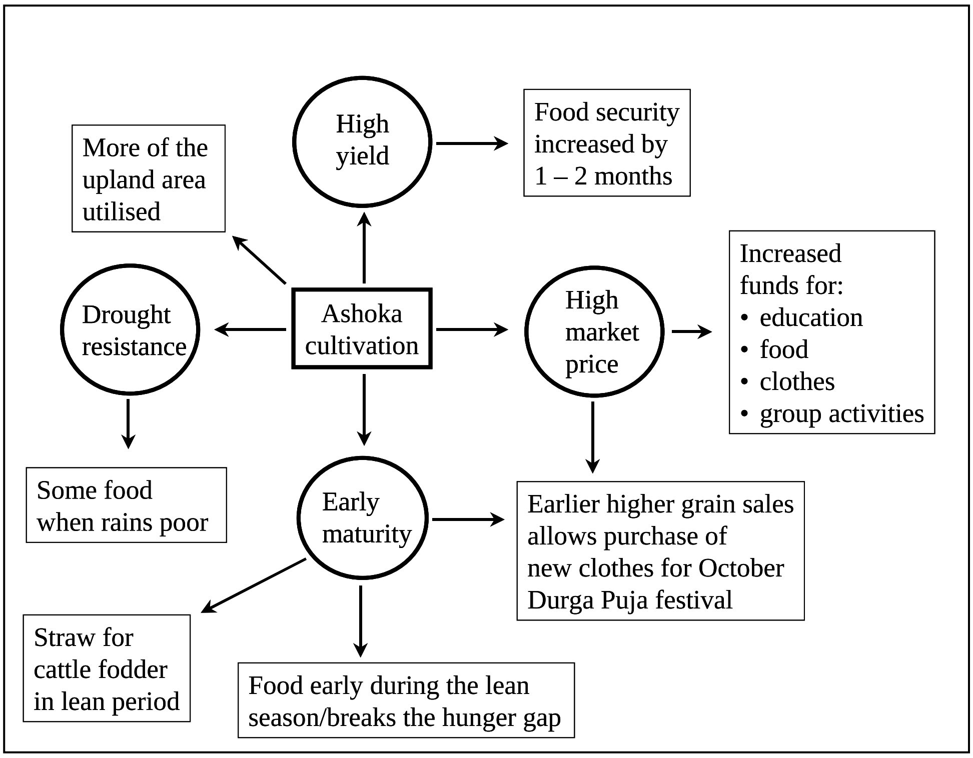

Figure 7. Diagram of the effects cultivating Ashoka can have on farmers' livelihoods, as determined in farmer group discussions. The diagram is a summary of group responses from 14 villages in three districts, October 2004.

Conclusions

This village level evaluation confirms the findings of the households surveys conducted in 2002 and 2004. The evaluation shows that there are high adoption rates of Ashoka 200F and 228; within 2 years of Ashoka being introduced to a village on average it is being cultivated on approximately 30% of the upland rice area. The area of Ashoka cultivation is increasing every year and its cultivation has enabled some farmers to increase their total area of cultivation by utilising fallow upland areas. Ashoka is being adopted at such high rates because it has a number of benefits over local varieties: high yielding, early maturity (90 days), drought resistance, good taste and short cooking time, good straw quality and high market price compared to local varieties.

Farmer-to-farmer seed exchange is occurring both within the same village and outside through seed traders, markets and relatives/friends. Triangulating with previous surveys, each farmer on average gives seed to at least one other farmer. This seed distribution activity is likely to increase. Initially surplus seed is mainly used to increase the on-farm area before farmers can distribute surplus seed to others.

However the informal seed spread is slow, at least in the initial stages, as farmers are limited in the amount of seed they can sell/exchange and the distances that they travel, additionally many of the poor farmers tend to eat the produce before they can disseminate it to other farmers.

To meet the required seed demand by farmers, increased production and dissemination of seed is therefore required. As the yield of upland varieties is lower than the transplanted rice, seed production is not as attractive to the private sector. Increased seed production is therefore required by NGOs, GOs and public companies, and large-scale seed production of truthfully labelled seed by farmer groups and NGOs needs to be supported. In conjunction with this, farmers require training on how to select for breeding seed to maintain quality. Awareness of the variety needs to be raised in order that dissemination is increased through NGOs, GOs, public and private companies (farmers are willing to pay up to 10Rs/kg). Dissemination could be enhanced further through the distribution of small seed packets to many farmers, thereby increasing the dissemination foci, and encouraging farmer-to-farmer spread.

The success of Ashoka production and cultivation highlights the impact that new varieties, produced through client orientated breeding, can have. To further enhance the opportunities of farmers in Eastern India development of additional varieties suitable to medium lands is also required.

Acknowledgements

I would personally like to thank Dr S. C. Prasad who was invaluable in facilitating the overall visit. I would also like to thank Mr V. K. Vij (GVT project manager) Mr S. L. Yadav, Mr P. K. Pathak and Mr P. K. Mishra (GVT State Coordinators of West Bengal, Jharkhand and Orrisa), Mr N.K. Sinah, Mr A. G. Das, and all other GVT staff including Mr A. Choudary, Mr Vikas Kumar and the community officers, for their cooperation and assistance in the village visits. Ultimately, grateful thanks to all the farmers who gave up their valuable time, enabling us to collate this information.

Appendix 1

Land map completed by farmers in Bagdisha, Purulia, October 2004

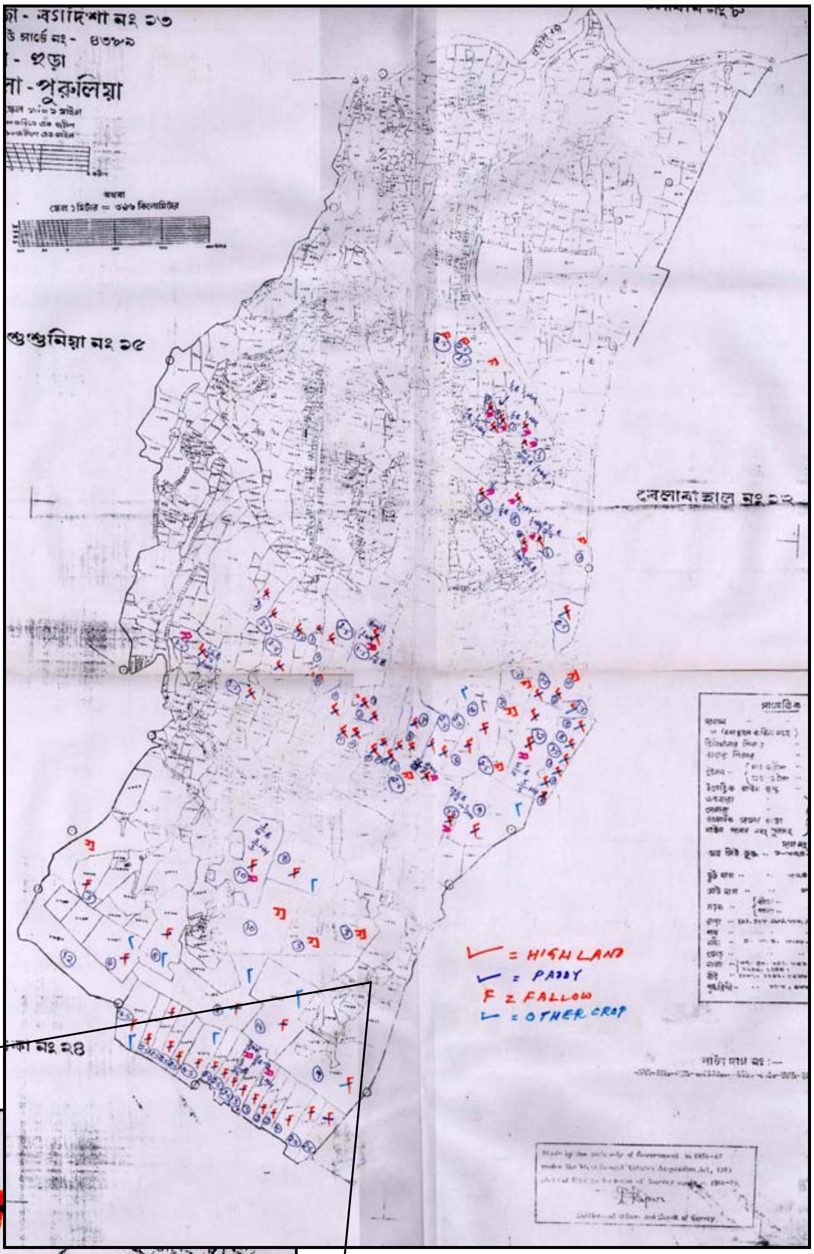

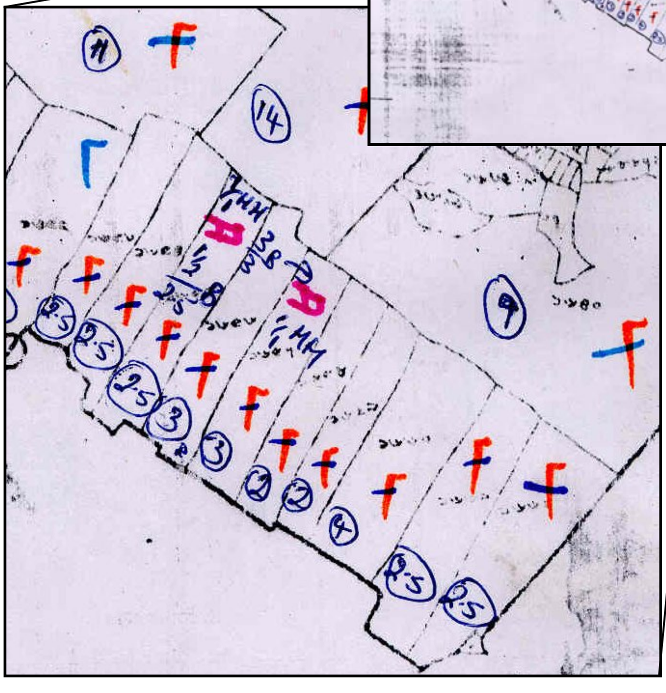

Appendix 2

Ashoka cultivation as a percentage of the total upland rice area in all villages that had been provided with seed for at least two years studied within the three districts.

District/State	Village	Total upland area (ha)	Upland rice area (ha) (% of upland)	Other crops/ fallow (ha) (% of upland)	Ashoka as % of upland rice area
Purulia, West Bengal	Asanbori	17.6	9.2 (52)	8.4 (48)	10
	Bagdisha	37.1	17.2 (46)	19.9 (54)	19
	Dumkadih	6.1	4.2 (69)	1.9 (31)	46
	Koldhi	38.2	19.5 (51)	18.7 (49)	14
	Ranipur	40.4	26.1 (65)	14.3 (35)	25
Ranchi, Jharkhand	Jamueria	10.1	5.5 (54)	4.6 (46)	38
	Mahudar hamlet	6.5	3.7 (57)	2.8 (43)	8
	Uruguttu	50.2	39.2 (78)	11 (22)	20
Mayurbhanj, Orissa	Bodabilla	37.5	15 (40)	22.5 (60)	63
	Kalajhinel	22.1	8.8 (40)	13.2 (60)	63
	Udali	37.8	22.7 (60)	15.1 (40)	18

* In the villages of Orissa state over 50% of the ‘other crops’ was Sabia grass
