# Supplementary material for: Plant breeding can be made more efficient by having fewer, better crosses
Source: BMC Plant Biol. 2013 Feb 7;13:22. doi: 10.1186/1471-2229-13-22 (PMC3621587; doi:10.1186/1471-2229-13-22)
Supplement: Additional file 6 — New Rice Varieties for Nepal. Rainfed Agriculture Impact Assessment Study No. 2. Monitoring Impact Assessment and Learning Component (MIL) of the Research into Use Programme (RiU); 2008. [file 1471-2229-13-22-S6.pdf]

A proposal for the release of an aromatic rice variety Suanulo Sugandha

(A rice variety developed using client-oriented breeding (COB) approaches)

Jointly Submitted by

Local Initiatives for Biodiversity, Research and Development (LI-BIRD)

CAZS-Natural Resources (CAZS-NR), Bangor University, Bangor, UK

National Rice Research Programme (NRRP), Hardinath, Dhanusha, Nepal

Jaskelo Youth Club (JYC), Chitwan

J. R. Witcombe[†], K. P. Devkota[§], M. P. Tripathi[§], S. Gyawali[§], M Subedi[§], P. K. Shrestha[§], B. N. Chaudhary[‡], R. B. Yadav[‡], M. Yadav[‡], D. Chaudhary[‡], A.P. Gautam[‡], T. Akhtar[‡], S. P. Khatiwada[‡], N P Adhikari[‡], K. Chapagain[¶] and K. D. Joshi[†§]

[§]*Local Initiatives for Biodiversity, Research and Development (LI-BIRD)*
[†]*CAZS-Natural Resources, Bangor University, UK*
[‡]*National Rice Research Programme (NRRP), Nepal Agricultural Research council (NARC) Hardinath, Dhanusha, Nepal*
[¶]*Jaskelo Youth Club (JYC), Chitwan*

January 2008

Application to be made for approval and release of crop varieties

Government of Nepal
Ministry of Agriculture and Cooperatives
National Seed Board
Format for submission of proposal for the release of a crop variety

The Variety Approval, Release and Registration Sub-committee (VARRC)
National Seed Board Secretariat
Seed Quality Control Centre
Harihar Bhawan, Lalitpur
Tel. 977-01-5521359

Sub: Application for the release of a rice variety Sunaulo Sugandha

As, we have selected new aromatic rice variety described below; we hereby make this application for the release of the seeds of rice variety as follows.

**Original entry name**	**Suggested name**
LIRI-BAS-77 (Plot # 65-1) Sugandha 2002	Sunaulo Sugandha

1. Background information

Rice (*Oryza sativa* L.) is the most important commodity in Nepalese agriculture and its economy as it is grown in about 1.55 m ha producing 4.3 m tons of rough rice with an average productivity of 2.8 t ha^{-1}. Share of rice is 20% to the agricultural Gross Domestic Product (GDP) and it contributes nearly 50% to the total calorie requirement of Nepalese people (CBS, 2003).

Basmati (aromatic) rice is a group of rice varieties that emit aroma on cooking and are considered very high quality rice. Most consumers in South Asia prefer long grain (length 6-7.5 mm), slender (2.5-3 length/breadth ratio), translucent, soft and aromatic grains. *Basmati* have superior eating quality with softness of cooked rice, remarkable expansion on cooking and pleasant aroma. These are grown in special niches of the terai, inner terai, valleys and foot hills of Nepal. Of these, Jetho Budho grown in Pokhara valley is most preferred, while *Seto* (white) and *Rato* (red) Basmati and *Kala Namak* are other preferred rice varieties grown in the terai. Jarneli and Pokhreli Masino, although, do not have aroma but are popular fine quality rice landraces (Joshi et al., 1997). Aromatic and fine grain rice landraces are photosensitive late maturing but with low yield potential, tall, non-responsive to nitrogenous fertilizers and prone to lodging. Some of these are also susceptible to rice diseases, e.g. blast. Although, there have been some efforts over last two decades to improve fine and aromatic rice through introduction and testing and also through landrace enhancement, but no modern aromatic rice variety has been released so far in the country. Introduced fine grain and aromatic varieties have had limited success. A stakeholders' analysis revealed that low net return from existing aromatic and fine rice landraces due to low productivity was the major for reason farmers not being very enthusiastic to expand area under fine and aromatic landraces (Sah, 2002).

Nepal used to export about 200 tonne of fine quality *Basmati* rice per annum earning 41 million rupees at 1977 price (Giri and Shahi, 1978) but due to population growth, Nepal stopped exporting rice. Production of aromatic and fine rice even today is limited in the country and with the improvement in the living conditions of the people, there is an increasing demand for better quality rice. It is assumed that major proportion of this is met by import from India, although, there is a lack of data on import and export of aromatic rice in the country.

It is estimated that aromatic and fine rice in Nepal may occupy around 10% of the total rice area i.e. 150, 000 ha (Sah, 2002). A postal survey and rapid rural appraisal conducted in Nepal during mid-1990s indicated a potential for over 30% increase in the area under aromatic rice if suitable technologies are available (Joshi et al., 1997). It was also found that over 60% cases the grain is used both for consumption and sale. The premium price for indigenous aromatic and fine quality rice varieties ranged from Rs. 2 to 20 per kg extra at 1997 price (2-30 cents) (Joshi et al., 1997).

Sunaulo Sugandha was bred using Client-Oriented Breeding (COB)[1].Several professionals from many organizations and farmers contributed in the development of Sunaulo Sugandha (Annexure 1). The details of the breeding procedures employed for the development of Sunaulo Sugandha are presented in Table 1. This involved screening large irradiated population of Pusa Basmati 1 that had outcrossed to other unknown parents. The selection focussed on identifying best aromatic and high grain quality rice lines under farmers' customary management and input levels and biotic and abiotic conditions of target population of environment (TPE) with active involvement of farmers. This proposal summarises the performance of Sunaulo Sugandha from a number of trials.

A comprehensive participatory evaluation for quality traits was done before advancing rice lines from this cross to any on-station or on-farm evaluation. This included assessment for physical qualities, e.g. aroma, qualities of unmilled and milled rice, cooking and eating qualities. The disease screening and yield testing of the rice lines was done by NRRP. A summary of all the trials with Sunaulo Sugandha as one of the test entries by NRRP and NARC research stations, LI-BIRD and DADO network are summarised in Table 2.

2. General Information

2.1. Common name: Rice
2.2. Botanical name: *Oryza sativa* L.
2.3. Original designation/Symbolic name used in testing: Sugandha 2002 subsequently evaluated as Sunaulo Sugandha in all the trials.
2.4. Pedigree/selection history: (Please see Table 1) below

[1] COB, previously known as participatory plant breeding (PPB) was implemented by LI-BIRD with technical support form CAZS Natural Resources (CAZS-NR), Bangor University, UK and in collaboration with National Rice Research Programme (NRRP), NARC, Jaskelo Yuba Club, Chitwan, District Agricultural Development Offices (DADOs) and the farming community of Chitwan and many other districts in Nepal. Department for International Development (DFID) Plant Sciences Research Programme (PSP) provided the financial support for COB

Table 1. Summary of breeding procedures of Sunaulo Sugandha, 1998-2007

Year	Season	Procedure
1998	Winter	Pusa Basmati 1 treated by Y-rays (done in the UK arranged by CAZS-NR).
1998	Main	Grown bulk seeds in 700 m^2 area and harvested selected 97 M_1 families having taller awnless and disease free plants. Farmers were involved in the selection process.
1999	*Chaite*	Grown individual families and selected 53 individual plants including LIRI-BAS-77 (Plot # 65-1).
1999	Main	Modified bulk harvesting after roguing
2000	Main	Bulk harvested after roguing
2001	Main	Bulk harvested after roguing
2002	Main	Plot # 65 was grown in $10m^2$ area with roguing
2003	Main	Disease screening nurseries at NRRP; organoleptic assessment in Chitwan by stakeholders
2004	Main	Disease screening nurseries at NRRP, CVT in RLM trial, Mother and Baby trials (LI-BIRD and other NGOs and DADOs across terai), organoleptic by stakeholders
2005	Main	Panicle selection for breeder seed production from 59 best hills from about 8000 IET and disease screening nurseries at NRRP and, Mother and Baby trials (LI-BIRD and NGOs and DADOs across terai) .
2006	Main	Disease screening nurseries at NRRP, CVT in RLM, Mother and Baby trials (LI-BIRD and other NGOs and DADOs)
2007	Main	Mother and Baby trials (LI-BIRD, other NGOs and DADOs), seed production.

2.5. Cross (parents)[2] Irradiation of Pusa Basmati-1 with gamma radiation at University of Nottingham arranged by CAZS-Natural Resources. However, this population was subsequently determined to have outcrossed to other unknown parents.

2.6. Country of Origin: Nepal

2.7. Source of material (Name of the institution, farmer/farming community, experiment and the year in which it was first introduced, etc): Irradiated seeds of Pusa Basmati 1 introduced from CAZS-NR, Bangor University, UK by LI-BIRD in 1998 and the entire breeding was done in Chitwan, Nepal.

2.8. Years and location of testing of the varieties in Nepal (see Table 2)

[2] Cross (parents) may not be applicable to landraces while for the hybrids, the proposing Company may or may not disclose the parentage

Table 2. Summary of years and locations of testing Sunaulo Sugandha in Nepal

Year	Experiment	Location	Source/ References[3]
2004 &2006	Coordinated varietal trial rainfed lowland medium (CVT RLM)	NRRP, Hardinath, RARS Parwanipur	Annexure 2
2004 to 2006	Yield (t ha^{-1}) performance of Sunaulo Sugandha in Mother trials in low land environment of Chitwan and Nawalparasi	Chitwan and Nawalparasi	Annexure 3
2006	Yield (t ha^{-1}) performance of Sunaulo Sugandha in aromatic Mother trials in Chitwan and Nawalparasi	Chitwan	Annexure 4
2004 to 2006	Agronomic traits of Sunaulo Sugandha and other main season rice varieties in CVT and Mother trials rainfed lowland medium (RLM)	Hardinath, Parwanipur, Jhapa, Morang, Sunsari, Saptari, Siraha, Chitwan, Nawalparasi and Kapilvastu	Annexure 5
2006	Grain yield (t ha -1) of Sunaulo Sugandha from crop cut survey of Baby trials from various districts of Nepal	Chitwan, Makwanpur, Rupandehi, Mahottari, Morang, Lamjung, Tanahun and Udaypur	Annexure 6
2006	Various rice varieties being replaced by Sunaulo Sugandha in 8 districts (as mentioned in Annexure 6) of Nepal.	Chitwan, Makwanpur, Rupandehi, Mahottari, Morang, Lamjung, Tanahun and Udaypur	Annexure 7
2004 to 2006	Cost-benefit analysis of Sunaulo Sugandha and Masuli Based on CVT, Mother and Baby trials	Various districts	Annexure 8
2003-2006	Disease data from National Bacterial Leaf Blight Screening Nursery and National Blast Screening Nurseries, other studies done at IAAS and LI-BIRD	NRRP, Hardinath, LI-BIRD Chitwan and Nawalparasi, IAAS, Rampur	Annexure 9-11
2003	Response of Sunaulo Sugandha and Masuli to rice insects observed in mother trials in on-farm trials in Chitwan district by plant pathologist from IAAS, Rampur, Chitwan	IAAS, Rampur	Annexure 12

Performance in Coordinated Varietal trials:

Table 3. Regression parameters of Sunaulo Sugandha and Masuli in rainfed lowland medium trials from CVT (n=11) and mother trial (n=25) where variety mean grain yield was regressed on trial mean yield of the test entry and Masuli from 2004 to 2007

Variety	Mean grain yield (t/ha)	b	SE of b	R^2 (%)	Range (t/ha)
Masuli	2.89 ± 0.145	1.02	0.1334***	63	1.39 – 4.91
Sunaulo Sugandha	3.05 ± 0.144	0.92	0.1517***	50	1.38 - 5.78

Sunaulo Sugandha is suitable from medium to low lands with rainfed to irrigated conditions having a potential of covering over 0.3 million ha from the terai to the river basins. It has exceptional combination of high yield potential, e.g. > 4 t ha^{-1} under farmers' management (which is not very common with high quality rice varieties), good aroma and excellent eating quality. It is non-lodging, responsive to applied nitrogenous fertilizers and has all the yield

[3] Details of experimental results have been presented at the end of the report and only Annexure numbers have been referred here.

components to support high yield. Because of good aroma and excellent eating quality, farm gate price of unmilled Sunalo Sugandha rice is Rs. 18 kg^{-1} which is 33% more than the price of Masuli, a most popular rice variety among Nepalese consumers. Remarkable thing about Sunaulo Sugandha is that it offers two fold advantages; higher yield and premium price thus nearly trebling the total returns to farmers compared to Masuli.

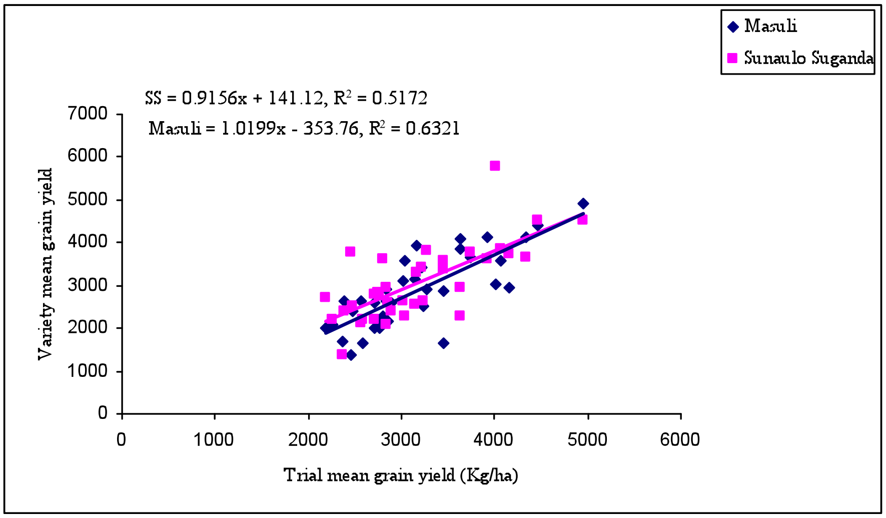

Figure 1. Stability (joint regression analysis) of Sunaulo Sugandha over standard check variety Masuli in CVT (n=11) and mother trials (n=25) from 2004 to 2007 in Chitwan, Nawalparasi, Bara and Dhanusha, districts of Nepal

Mother trials:

2.9. Farmers Field Trials (FFTs), participatory trials data/information and stakeholders'[4] preferences and major traits[5] of stakeholders' interest

Table 4. Summary of years and locations of testing Sunaulo Sugandha in Baby trials, participatory rice quality assessment and organoleptic assessment in Nepal

Year	Experiments/trials	Location	Source References
2005<br>2006	Paired comparison of rice genotypes in Baby trials	Jhapa, Morang, Sunsari, Saptari, Shirha, Dhanusha, Mahottari, Sarlahi, Rautahat, Bara, Parsa, Makwanpur, Chitwan, Nawalparasi, Rupandehi, Kapilvastu, Dang, Banke, Bardiya, Kailali, Kanchanpur	Table 4
2003<br>2004	Participatory rice quality assessment (physical, milled and cooked rice qualities)	Chitwan	Table 9

[3] Stakeholders refer to farmers, consumers, processing industry, merchants, and seed companies/Agrovets.
[4]Major traits of stakeholders' interest include (1) agronomic data (2) Stress tolerance (3) quality traits (4) Economic value (5) Others specify if any. Please attach the details of experimental results at the end and refer only Annexure numbers here.

Performance in Baby trial

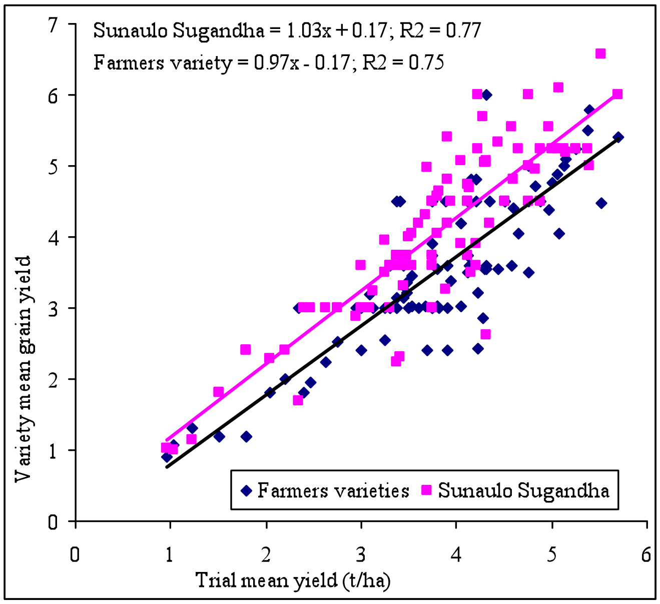

Figure 2. Stability (joint regression analysis) of Sunaulo Sugandha over farmers variety from Baby trial crop cut data (n=101) in 2006 from Chitwan (52), Makawanpur (7), Rupandehi (3), Mahottari (3), Morang (9), Lamjung (15), Tanahun (2) and Udayapur (10) districts of Nepal

Table 5. Grain yield of Sunaulo Sugandha from crop cut survey from Baby trials in 5 terai districts and 3 hilly districts, 2006

**Region‡**	**N**	**Farmers' varieties‡ ($t\ ha^{-1}$)§**	**Sunaulo Sugandha ($t\ ha^{-1}$)§**	**Yield advantage over farmers' varieties (%)**	**t at 0.05**
Eastern Terai	12	3.56±0.19	4.30±0.27	21.0	*
Central Terai	62	3.80±0.15	4.09±0.16	7.5	*
Hilly area	27	2.74±0.19	3.77±0.25	38.0	***
Overall mean	101	3.55±0.20	4.09±0.12	15.0	***

‡ See Annexure 6 for details of locations and farmers' varieties
§Mean ± SE,

The performance of Sunaulo Sugandha was compared to the yield of the best available aromatic varieties in some of the Mother trials (Annexure 4). Sunaulo Sugandha yielded more than 1.5 t ha^{-1} than these aromatic landraces while having better BLB resistance and lodging resistance.

2.10 Farmers' perceptions and preferences:

Summary of farmers preferences for Sunaulo Sugandha (SS) have been presented in Table 6 Based on results from 32 baby trials conducted in 2005 and 160 Baby trials in 2006 in several districts of Nepal, Sunaulo Sugandha was preferred variety for its excellent eating quality with aroma, higher market price and higher grain yield with higher milling recovery than the farmer's popular varieties. Higher tillering, lodging tolerance, easy threshing are other desirable traits (Table 6). Willingness of over 80% respondents in both the years to continue growing this variety indicates its very high preference as it was also highly rated for overall performance (Table 6).

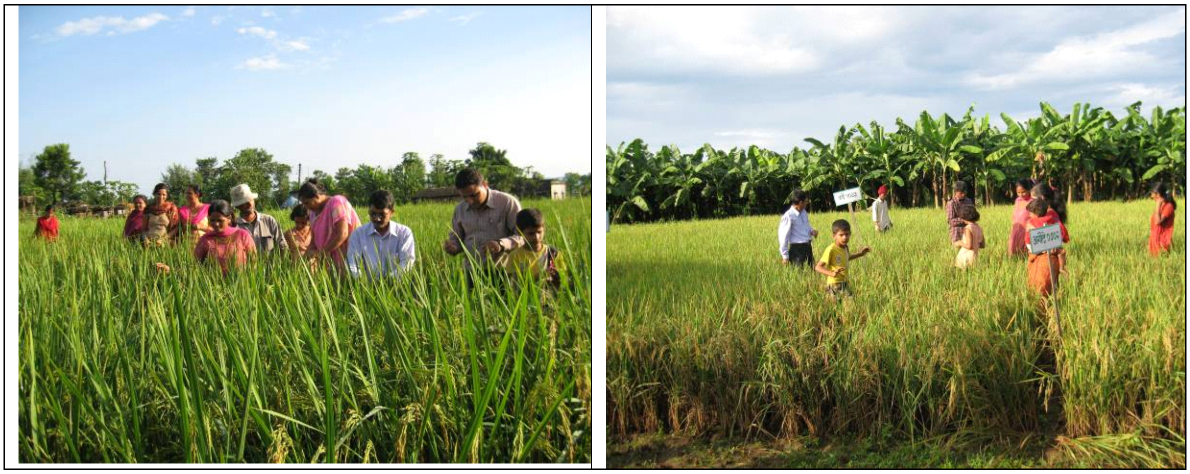

Farmers evaluating Sunaulo Sugandha (left) and preference ranking at a Mother Trial (right)

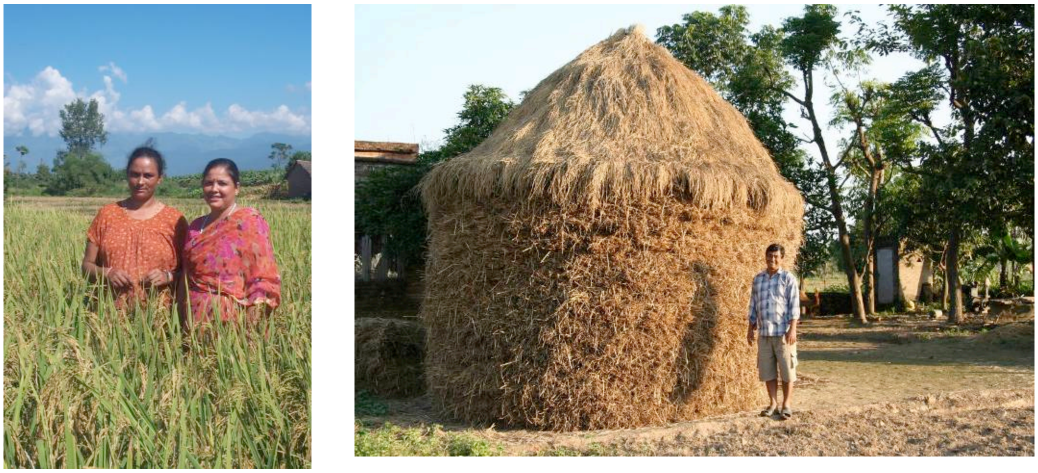

Sunaulo Sugandha near maturity (left) and Stack of harvested Sunalulo Sugandha (right)

Table 6. Farmers' perceptions from 32 Baby trials from seven districts in 2005 and 160 baby trials from 14 districts of Nepal, 2006‡

Trait	2005			2006		
	N	Preferring Sunaulo Sugandha (%)	Signif-icance	N	Preferring Sunaulo Sugandha (%)	Signif-icance
Maturity	30	30	0.439ns	145	21	0.001***
Tillering capacity	31	39	0.020*	152	55	0.000***
Lodging	30	17	0.000**	145	87	0.000***
Disease	32	50	0.102ns	154	33	0.345ns
Threshing	32	53	0.001**	142	51	0.000***
Milling recovery	32	44	0.008**	136	38	0.08ns
Eating quality	32	84	0.000**	138	88	0.000***
Market price	22	41	0.011*	126	77	0.000***
Straw yield	32	41	0.275ns	154	50	0.000***
Grain yield	32	47	0.005*	142	55	0.000***
Will grow again	29	86	0.002***	142	84	0.000***
Overall performance	30	83	0.000***	148	78	0.000***

‡ In 2005 Baby trials were conducted, with number in the parenthesis represents number of trials, in: Kailali (2), Kanchanpur (1), Makawanpur (10), Morang (2), Saptari (3), Siraha (5) and Sunsari (9)] while in 2006 baby trials were conducted in Bara (1), Chitwan (57), Jhapa (6), Lamjung (20), Mahottari (9), Makawanpur (8), Morang (23), Nawalparasi (2), Rupandehi (10), Parsa (3), Tanahun (5), Syangja (1), Sunsari (2), Udaypur (13).

Stakeholder's perceptions

Farmer's perception about Sunaulo Sugandha were received from seven districts Kailali, Kanchanpur, Makwanpur, Morang, Saptari, Siraha and Sunsari districts in main season 2005.

Preferred traits

1. Aromatic and fine grains
2. High grain and straw yields
3. Long Panicle
4. High milling percentage
5. Good eating quality
6. Fertilizer responsive
7. Non-lodging

3. Summary of Varietal Characteristics

3.1. Agronomic[6]

Agronomic data are summarised from three years' averages from CVT trials except for 3.1.4 which is the mean of all the trials in which yield was measured from CVT, Mother trials and crop cut of Baby trials (Table 7). Overall, yield advantage of Sunaulo Sugandha over Masuli is 13% considering the results of CVT, Mother and Baby trials (n=137) from various districts of Nepal.

Table 7. Agronomic performance of Sunaulo Sugandha relative to Masuli based on the CVT, Mother trial and Baby trial combined mean data from 2004 to 2006

S. No.	Characteristics‡	Sunaulo Sugandha	Masuli
**3.1.1.**	**Morphological traits**		
3.1.1.1	Plant height (cm)	105±1.3	117±1.4
3.1.1.2	Days to maturity	151±1.0	151±1.6
**3.1.2**	**Yield components**		
3.1.2.1	Yield (t ha^{-1}) (n = 137)	3.82±1.2	3.38±1.1
3.1.2.2	Yield range (t ha^{-1})	1.38-5.77	1.39-4.90
3.1.2.3	Panicle m^2 (Number)	272±5.3	260±8.3
3.1.2.4	Panicle length (cm)	27±0.4	23±0.4
3.1.2.5	Number of grains per panicles	141±13.9	136±77.1
3.1.2.6	1000 rough rice grain weight (g)	19±0.4	18±0.8
3.1.2.7	1000 milled rice grain weight (g)	14.75	

Nutrient responsiveness: From case studies of Hari Khadka, Shankar Bhatta and other farmers

3.2. Response to stresses:

3.2.1 Biotic stresses

(a) *Diseases:* Sunaulo Sugandha has field tolerance to blast as the disease scores recorded in all the National Blast Screening Nurseries from 2003 to 2006 it never scored more than 4 in a scale of 0-9 (Table 8, Annexure 9-11). Remarkable thing about the reaction of Sunaulo Sugandha to BLB is that although, in artificially inoculated trials it scored between 5 to 7 in a 0-9 scale (Table 8), but in spite of that its yield performance was extremely good, which indicated its field tolerance. A comparison of Sunaulo Sugandha with other aromatic rice varieties and landraces for BLB revealed that its BLB scores were similar to other popular aromatic varieties except Pusa Sugandha-2 which is a released variety in India (Table 9), again the its yield level is much higher than the Indian aromatic varieties. It is very well acknowledged fact that BLB is a severe constraint in aromatic rice production. All the existing aromatic varieties are highly susceptible

[6] These traits may vary depending up on the crop species

and the disease is prevalent in the entire aromatic growing regions (Joseph et al., 2004).

Table 8. Summary of blast scores from 2003 to 2006

Variety	Blast scores (0-9)				Maximum Score (0-9) In those set of trials
	2003	2004	2005	2006	
Sunaulo Sugandha	0	0	0	4	4
Resistant check (Laxmi)		3	0	0	3
Susceptible Check (Masuli/Sankharika)		9	8	7.5	9
Maximum score recorded (0-9)	4	9	8	8	9

Source: NRRP unpublished data 2003 to 2006

§ Leaf and neck blast scoring was done using IRRI Standard Evaluation System (SES) for rice in 0-9 scale, mean of three replicates. For blast on the basis of severity of infection on panicle, 0 = no incidence, 0 = no lesions 3= Significant number of roundish to slightly elongated necrotic grey spots about 1-2 mm in diameter with a distinct brown margin 5= Typical blast lesions infecting 4-10% of leaf area, 7= Typical blast lesions infecting 26-50% of leaf area, 9= Typical blast lesions infecting >75% of leaf area

Table 9. Summary of BLB scores of aromatic rice varieties from 2003 to 2006

Variety	BLB scores (0-9)				Maximum Score (0-9) In those set of trials
	2003	2004	2005	2006	
Sunaulo Sugandha	7	5	7	7	7
Rato Basmati		5			
Sugandha 2-2		7			
Sugandha 3-1		7			
Pusa Sugandha-2		9			
Basmati 101				5	
Resistant check (Laxmi)		3	3	3	3
Susceptible check (TN1)		7	9	9	9
Maximum score recorded (0-9)	7	7	9	9	9

Source: NRRP unpublished data 2003 to 2006, BLB= Bacterial leaf blight

Where 0 = no lesions 3= Significant number of small brown specks of pin-point size or large brown specks without sporulating centre 5= Typical BLB lesions infecting 4-10% of leaf area 7= Typical BLB lesions infecting 26-50% of leaf area 9= Typical BLB lesions infecting >75% of leaf area

Table 10. Summary of insect scores for Sunaulo Sugandha at NRRP in 2005

Varieties	Brown plant hopper (BPH)	Stem borer (dead heart)	Stem borer (white head)
Sunaulo Sugandha	MS†	R	MR
TN 1 (susceptible check)	HS	MS	MS
Sabitri (Resistant check)	MR	R	R

† MS = moderately susceptible, HS= highly susceptible, MR=moderately resistant, R= resistant
Source: P. Thakur, NRRP; unpublished data 2005

(b) *Weeds:* None of the specific weed problems were associated with this variety.

3.2.2 Abiotic stresses

For best results this variety should not be exposed to drought stress from booting to maturity.

3.3 Quality of economically important parts of the crop

3.3.1 Nutritional quality:

Table 11. Nutritional quality of selected rice varieties, 2006

Samples	Percentage of parameters						Energy Kcal/ 100g	Zinc (ppm)	Iron (ppm)
	Mois ture	Fat	Protein	Total Ash	Crude Fibre	Carbo Hydrate			
Sunaulo Sugandha	14.3	2.5	9.8	6.4	12.8	69	336	13	83
CNTLR	13.0	2.1	7.8	3.1	13.3	74	345	25	203
Sugandha 1	13.5	2.1	7.3	3.2	11.7	76	351	15	83
Masuli	11.9	2.3	10.1	3.1	12.3	72	350	22	213
Ram (OR367)	13.2	2.5	9.7	6.8	11.4	70	340	19	410
Sabitri	12.1	2.5	9.7	4.4	11.0	72	351	15	129
Barkhe 3004	13.4	2.2	10.9	5.2	11.9	70	343	18	77
Mithila (BPI-3-2)	12.1	2.1	9.4	5.9	12.5	70	337	13	153
Kanchhi Masuli	13.0	2.1	9.2	4.7	10.7	73	349	16	62
Radha 4	11.5	2.1	8.8	3.2	10.2	76	357	17	145

Source: LI-BIRD unpublished data, 2006

3.3.2 *Processing quality:*

Un-milled rice

Table 12. Participatory visual assessment of grain quality of new and popular rice varieties of Nepal Chitwan in 2003 and 2004.

Variety	Husk colour	Grain type		Rice grade
		Width	Length	
Sunaulo Sugandha	Dull brown	Fine	Medium	Good
CNTLR	Straw	Bold	Long	Good
Pusa 834	Straw	Bold	Long	Fair
Masuli	Golden	Medium fine	Medium	Excellent
Sabitri	Straw	Medium	Medium	Good
Mithila (BPI 3-2)	Lighter than Masuli	Same as Masuli	Same as Masuli	Good
Swarna	Like Masuli	Medium	Short	Fair
Radha 4	Straw	Bold	Short	Poor

Milled rice

Table 13. Participatory visual assessment of grain quality of new and popular rice varieties of Nepal Chitwan in 2003 and 2004.

Variety	Length	Width	Colour	Grain Breakage	Grading rice for the quality of *bhat*	White belly	Remarks
Sunaulo Sugandha	Long	Medium	Ghee white	Slightly broken	Excellent	Translucent	Aroma present
CNTLR	Long	Medium fine	Ghee	Not broken	Excellent	Translucent	Special aroma
Pusa Basmati-1	long	Fine	Ghee	Few broken	Excellent	Translucent	Aroma
Masuli	Medium	Medium fine	Ghee	Not broken	Excellent	Translucent	
Sabitri	Medium	Medium	Ghee	Not broken	Excellent	Translucent	
Mithila (BPI 3-2)	Medium	Medium fine	Ghee white	Not broken	Good	<10% area of grain	
Swarna	Medium	Medium	White ghee	Not broken	Good	<10% area of grain	
Kanchhi Masuli	Short	Bold	Ghee	Slightly broken	Poor	11-20% area of grain	
Radha 4	Short	Bold	White	Slightly broken	Poor	11-20% area of grain	

3.3.3 *Organoleptic test (cooking quality, taste, aroma/flavour, etc):*

Cooked rice

Table 14.a. Organoleptic assessment of cooked rice of new rice and popular rice varieties of Nepal Chitwan in 2003 and 2004

Variety	Softness	Flakiness	Taste	Water Absorption	Inner Hardiness	Remarks
Sunaulo Sugandha	Medium	Good	Tasty	High	Absent	Aroma
CNTLR	Soft	Good	Tasty	High	Absent	Aroma
Pusa Basmati-1	Soft	Good	Tasty	High	Absent	Aroma
Masuli	Soft	Good	Tasty	Medium	Absent	
Sabitri	Medium	Good	Medium	High	Present	
Mithila (BPI 3-2)	Medium	Good	Testy	Medium	Absent	
Kanchhi Masuli	Medium	Medium	Low	High	Present	
Swarna	Medium	Medium	Medium	Low	Absent	

Table 14.b. Post harvest evaluation of rough paddy, milled rice and cooked rice along with popular rice varieties in Chitwan during 2007

SN	Genotypes	Rough paddy			Milled rice			Cooked rice		
		Length	Breath	L/B	Length	Breath	L/B	Length	Breath	L/B
1	Sabitri	0.762	0.177	4.311	0.572	0.162	3.536	0.920	0.234	3.932
2	Pusa Basmati 1	1.120	0.178	6.280	0.730	0.152	4.813	1.196	0.216	5.537
3	Masuli	0.792	0.177	4.481	0.507	0.145	3.494	0.916	0.248	3.694
**4**	**Sunaulo Sugandha**	**0.975**	**0.172**	**5.680**	**0.683**	**0.158**	**4.316**	**1.132**	**0.214**	**5.290**
5	Lalka Basmati	0.842	0.183	4.591	0.605	0.165	3.667	0.922	0.238	3.874
6	Kanchhi Masuli	0.728	0.188	3.867	0.517	0.163	3.163	0.686	0.260	2.638
7	Swarna	0.763	0.180	4.241	0.553	0.158	3.495	0.840	0.242	3.471
8	CNTLR				0.695	0.163	4.255	0.946	0.270	3.504
9	Mithila (BPI 3-2)				0.605	0.152	3.989	0.896	0.242	3.702

3.4. *Other characteristics (threshing, storability, market potential etc.):*

3.4 Economic returns:

Market price: A survey in 2007 of various aromatic and fine grain rice varieties with the rice millers, traders (wholesalers and retailers) and the super markets in Chitwan revealed that Sunaulo Sugandha has established a milled grain price that is 33% higher than Masuli and similar to fine-grained non aromatic Jira Masino and Rato Basmati (Table 15a).

Table 15a. Market price of milled rice marketed in 5 kg bags of some leading rice varieties in Naryangadh, Chitwan, September, 2007

**Brand name**	**Price (Rs./kg)**
Shyam Bhog‡	28
Masuli (Pure Masuli)‡	30
Sugandha Long†	33
Jira Masino‡	36
**Sunaulo Sugandha***	**40**
Rato Basmati (Lalka Basmati- without aroma) ‡	45
Rato Basmati (Lalka Basmati)§	48
Supriya Hulas*	52
Arati*	53
Gauri Basmati*	55
Supriya long*	55
Premium Basmati*	58
Pemium Idli rice (Basmati) *	58
Makhan*	58
Hulas Basmati*	59
Jasmine (Shahnai Brand) *	74

‡Fine grain rice without aroma.
§Has light aroma,
†Indian variety without aroma
*Aromatic variety

But this is without any promotional effort and in the context that variety is yet to be popular among rice millers, traders and major super markets in the country. In December 2007, a subsequent survey in Chitwan showed that the price advantage of the un-milled rice (paddy) of Sunaulo Sugandha was now 33% higher than that of Masuli. Hence, it is anticipated that in 2008 the price advantage of the milled rice of Sunaulo Sugandha will increase further to values in excess of 33%.

Table 15b. Purchase and selling prices (Rs/kg) of unmilled (paddy) rice of Sunaulo Sugandha and Masuli achieved by two seed Producer Groups in Chitwan, December, 2007

	**Paddy**				**Milled rice**	
Variety	Shree Ram SPG		Dev Ujjawal SPG		Shree Ram SPG	Farmer** (Umanath Kandel)
	Purchase	Selling	Purchase	Selling	Selling	Selling
Masuli	15.50	16.50	15.50	16.50	28-32	28-30
Sunaulo Sugandha	20.00	22.00	20.00	21.00	40-60*	37-40

* market price depend on season (during Dhasain &Tihar the price was highest)
** Umanath Kandel is a farmer growing SS for last four years in 2 ha land and selling paddy and milled rice independently in the market.

4. Morphological characteristics (size, shape, colour, etc):

Traits were measured in 2004 in Gitanagar, Chitwan from a lowland irrigated environment

Characteristics	Value	Characteristics	Value
4.1. Leaf characteristics		***4.4 Panicle characteristics***	
Length (cm)	21.8	Length (cm)	28.97
Width (cm)	1.0	Type	Intermediate
Blade Pubescence	Intermediate	Secondary branching	Heavy
Blade colour	Green	Exertion	Well exerted
Basal leaf sheath colour	Green	Axis	Droopy
Angle	Erect	Shattering	Low (1-5%)
Flag leaf angle	Intermediate	Threshability	Intermediate
Leaf senescence	Late and slow	***4.5. Grain characteristics***	
4.2. Ligule Characters		Awning	Awnless
Length (cm)	2.2	Apiculus colour	White
Colour	White	Stigma colour	White
Shape	Cleft	Lemma and palea colour	Brown
Collar colour	Pale green	Lemma and palea pubescence	Hairs on lemma keel
Auricle colour	Pale green	Sterile lemma colour	Straw (yellow)
4.3. Culm characteristics		Sterile lemma length (mm)	0.5
Length (cm)	103.7	Spikelet sterility (%)	Fertile (75-90%)
Number per plant	8	Length (mm)	0.98
Angle	Intermediate	Width (mm)	0.17
Culm diameter (cm)	0.55	Length breadth ratio	4.0
Internode colour	Green	Seed coat (bran) colour	Brown
Culm strength	Strong (non-lodging)	Endosperm type	Non-glutinous (non waxy)
		Aroma	Lightly scented

4.1 Major identifying characteristics of a crop variety for its authenticity (distinctness)

Sturdy plants with stay green leaves, erect flag leaf, long panicles, medium fine grains and light golden husk colour.

4.2. Molecular characteristics:
Molecular data do not exist.

5. Recommendation domain:

5.1 Agro-ecological zone: Terai and inner terai.

The variety does very well in *terai* (east to west) and inner *terai.* Not formally evaluated in the river basins (however, farmers in the low hills and river basins have already successfully grown this variety). The variety could also be recommended for the river basins of Nepal up to 500 m.

5.2 *Moisture regime:* Rainfed medium land to lowland and irrigated conditions. The variety is bred for medium to semi-deep rainfed and irrigated lowland production environment as classified by IRRI. It is not recommended for deep water conditions.

5.3. Growing seasons and conditions: Main season from May June to October-November. It is best suited for rainfed or irrigated intermediate lowland and medium lands and it is best adapted in irrigated and high input conditions.

5.4 Cropping patterns:

This variety is growing in a number of rice-based cropping patterns from Jhapa to Rupandehi including some of the hilly districts, e.g. Tanahun and Lamjung. The most common patterns where this variety could be grown are as follows:

- Rice-winter vegetables-mungbean or maize or *Chaite* rice, vegetables
- Rice-fallow- mungbean or *Chaite* rice or spring maize
- Rice-potato-maize or *Chaite* rice
- Rice-wheat-fallow
- Rice-mustard-maize or *Chaite* rice or mungbean

5.5 Production and management aspect (please add detailed information on package of practices of this variety that may differ from those recommended for the crop):

5.5. Production and management aspects (package of practices)

5.5.1 Land Preparation: Farmers customary practice of land preparation is appropriate for this variety.

5.52 Seed rate: 50-60 kg ha^{-1} based on germination percentage.

5.5.3 Fertilizer application: From the stability analyses (Figs 1 and 2) it is clear that Sunaulo Sugandha does not differ significantly from Masuli or other commonly grown varieties in its responsive to improved environmental conditions. Many of the farmers that conducted the Mother and Baby trials reported that it is quite responsive to added fertilizers particularly as it is highly resistant to lodging compared with other aromatic varieties and Masuli. Nitrogenous

fertilisers should be given in split doses to reduce any incidence of BLB and also to increase nitrogen use efficiency. Other important aspects of nutrient management in this variety in relation to BLB management and fertiliser use efficiency are as follows:

- Avoid the use of un-decomposed farm yard manure; high dose of nitrogenous fertilisers
- Do not grow this variety close to biogas slurry, compost heap and under shade
- Increase the application of potassium fertilisers

5.5.4 Seedling age and transplanting time: Transplant younger seedlings, preferably <20 days old, under controlled irrigation. Because it has similar duration to popular varieties such as Masuli normal farmers' practice can be followed for transplanting time. However, if possible it is advantageous to transplant about 10 days to two weeks earlier than the current planting time to allow for earlier harvesting of the crop and timely planting of winter crops. This also makes sure that delayed transplanting is not done as, like all aromatic varieties, this leads to a higher incidence of insect pests.

5.5.5 Spacing: Maintaining specific row to row and plant to plant spacing under manual transplanting may be less practicable. However, make sure to have a minimum of 25 hills per m^2 to get good harvest.

5.5.6 Irrigation: This variety prefers alternate wetting and drying conditions to give best yield, i.e. it does very well under controlled irrigation condition. To get best return from this variety the crop should not suffer from drought stress from booting to maturity.

5.5.7. Disease: This variety is resistant to leaf and neck blast. Although, a score between 5-7 on a 0-9 scale has been reported for bacterial leaf blight on artificially inoculated condition (Annexure 9-11), it gave very good grain and straw yield performance indicating field tolerance to BLB. Moreover, there are no other aromatic and high quality rice varieties that have lower score of BLB than Sunaulo Sugandha with good combination of yield and quality.

Following management practices can help reduce the incidence of this disease:

- Double rice cropping may increase the disease pressure.
- Diseased rice stubbles, voluntary plants may act as the source of disease inoculum.
- Drain water from the rice paddies in case of disease symptoms in the crop field.
- Treat rice seeds with antibiotic e.g. Streptomycin

5.5.8. Insect: Since it has aroma in the standing condition the infestation of stem borer is relatively more which would need to be addressed in time to control the possible loss of grain due to the stem borer attack.

5.5.9. Intercultural operation: Two weeding are recommended. The first weeding should be preferably done at 20-30 days after transplanting whereas the second weeding at 60 days after transplanting.

5.5.10 Harvesting: Since, it is non-shattering, manual or mechanical harvesting could be done. Farmers practice of harvesting, drying and threshing such as sun drying for 2 days in the field and then manual threshing or by mechanical threshing can be followed.

5.5.11 Storage: Sun drying of harvested grain for several days is a common practice in Nepal which could be followed for Sunaulo Sugandha too. To reduce the loss of grains in the storage from moths and weevil, grains should be sufficiently sun dried to bring grain moisture near to 12% before grain is stored in Jute sacks under cool and dry storage.

5.6. Reasons for release:

- Although a few aromatic and high quality rice landraces are grown in the country, they are low yielding. Not a single modern aromatic and high grain quality of rice is released in Nepal (Jetho Budho, a landrace released in 2006 has very localised adaptation in the areas irrigated with cold water in Pokhara valley and so is unlikely to be widely grown). A large volume of aromatic and high quality rice (worth many millions of rupees) is imported from other countries particularly from India. There is ever increasing demand for aromatic and high quality of rice in the country which can only be fulfilled by developing and recommending suitable modern, high yielding, farmer preferred aromatic and high quality rice varieties for various rice domains in the country.
- Sunaulo Sugandha has a great combination of high yield along with quality traits, e.g. aroma, very good taste and high market price. Unlike many other aromatic varieties, it has sturdy plants and is non-lodging and highly input responsive.
- The variety has similar yield with most popular variety Masuli but has considerably better grain quality and higher market price. Farmers, millers and traders reported that there is a price advantage of at least Rs. 15 per kg for milled rice of Sunaulo Sugandha over Masuli (Table 15). In addition, the agronomic advantage of the variety over Masuli is that it performs extremely well in the low lying *Ghol*/*Khalla* lands where Masuli does not do well because of lodging problem and excessive blast infestation. This meant that combined added advantage of this variety over Masuli is enormous.
- Preferred by farmers for its ease of threshing.
- It is resistant to blast (leaf and neck), and has filed tolerance to bacterial leaf blight (BLB). There are no other aromatic varieties in the rice gene pool of the country which have better disease resistance.
- There are no other aromatic rice varieties with high yield potential, aroma and very good grain quality and good combination of other traits as that of Sunaulo Sugandha. Therefore there is a strong justification to release this variety without any delay to provide the potential benefit from this variety to the numerous farming communities across the country.

5.6.1 Recommendation by VARRC members:

In the third week of October 2005, near the maturity period of rice crop, a monitoring visit of the members of VARRC was organized for them to see the field performance of Sunaulo Sugandha (Sugandha 2002) and other potential varieties for release e.g. Barkhe 3004 under farmers' management and interact with the farming communities to have first hand information regarding the performance and preference of Sunaulo Sugandha by the farmers. The team also visited the plots of some of the rice varieties proposed by NRRP for release. The team visited several Mother, Baby trials, seed and grain production plots and held discussions with several farmers groups and individual farmers and traders in east and west Chitwan and Nawalparasi. The team members were very positive about the performance of proposed rice varieties. The team after

considering direct observation, farmers and traders interest recommended to submit variety release proposals for three rice varieties developed by LI-BIRD-CAZS-NR and NRRP collaboration, e.g. Sunaulo Sugandha, Barkhe 2014, Barkhe 3004. Of these, Barkhe 3004 has already been released by National Seed Board (NSB) in 2006. Following the recommendation of VARRC, the variety release proposal for Sunaulo Sugandha has been prepared and submitted.

The Technical Committee of National Seed Board on its decision of 24 December 2007 approved this variety to be proposed to VARRC for final hearing after incorporating all the comments raised by the committee members.

5.6.2 Adoption by the farmers:

Adoption and uptake of this variety was assessed through crop monitoring studies, household level questionnaires and in-depth interviews. Acceptance and uptake of this variety is increasing in rainfed lowland and medium lands and in irrigated high input and good management. Farmers preferred this variety because of its higher grain yield, input responsiveness, non-lodging and less diseases particularly blast and sheath blight. Good milling recovery and higher market price both for rough rice and milled rice make it really attractive option for growing in the low-lying areas replacing Masuli, which is increasingly becoming susceptible to a range of diseases. It is replacing several of the farmers varieties followed by Sabitri, Masuli and Jhapali Masuli. Although, seed distribution of this variety started in 2004 it was not in any significant quantity until 2005. In last three years, a total of over 20 tonne seeds were sold or distributed to farmers in several districts (see seed supply situation below). This amount is adequate to cover about 400 ha of land. This is ignoring seed production and distribution by seed producers groups and farmer-to-farmer spread of a seed which is a minimum of 1:3, i.e. one farmer giving out seeds to at least three farmers. This indicates the variety has already covered considerable area in the terai.

6. Seed supply situation:

Seed category	Seed quantity (t)				Institution
	2005	2006	2007	Total	
Breeder	0.5	0.5	3	4	LI-BIRD, Chitwan
Truthfully labelled	5	8	5	18	LI-BIRD, Chitwan
Truthfully labelled	2	2	3	7	SRSPG, Chitwan
Truthfully labelled			6	6	NASPG, Jhapa
Truthfully labelled			7	7	KPWSPG, Rautahat
Truthfully labelled			2	2	DADO Mahottari
Truthfully labelled			3	3	SBSPG, Bara
Truthfully labelled			5	5	DADO Rupandehi
Total	7.5	10.5	34	52	

SRSPG= Shree Ram Seed Producers' Group, NASPG= Nawa Adarsa Seed Producers Group, KPSPG= Krishna Pranami Women Seed Producers Group, SBSPG= Satobhai Seed Producers Group

Both LI-BIRD and NRRP will be responsible for the production of breeder seed as per this joint proposal. LI-BIRD can produce foundation seed, while certified or truthfully labelled seed production can be undertaken by any registered seed producer.

Proposed by

Signature:

Name: Krishna P Devkota
Main proposing organisation:
Local Initiatives for Biodiversity, Research and Development (LI-BIRD)
Designation: Plant Breeder
Corresponding address:
P.O. Box 324, Pokhara, Nepal

Tel. 061 526834/532912/535357
Fax: 061 539956
Email: info@libird.org
Website if applicable: www.libird.org

Signature:

Name: Prof J R Witcombe
Collaborating organization:
CAZS-Natural Resources, Bangor University

Designation: Director
Corresponding address:
CAZS-Natural Resources, Bangor University, Gwynedd, LL 57 2UW, UK
Tel. +44 1248 382116
Fax: +44 1248 371533
Email: j.r.witcombe@bangor.ac.uk
Website if applicable: www.cazs.bangor.ac.uk

Signature:

Name: Dr Ram, B Prasad
Collaborating organization:
National Rice Research program (NRRP)
Designation: Plant Breeder
Corresponding address:
Hardinath, Dhanusha, Nepal
Tel. 041 523000
Fax: 041
Email:
Website if applicable:www.narc-nepal.org

Signature:

Name: Krishna Chapagain
Collaborating organization:
Jaskelo Youth Club, Chitwan
Designation: Collaborator farmer
Corresponding address:
Ujwalnagar, Chitwan, Nepal
Tel. 056 620126
Fax:
Email:
Website if applicable:

7. Acknowledgements

This document is an output from projects R6748, R 7412 and R 8071 funded by Plant Sciences Research Programme of the UK Department for International Development for the benefit of developing countries. The views expressed are not necessarily those of the DFID. Farmers of Chitwan, Nawalparasi and all the other districts of Nepal who directly participated in various activities of COB, Mother, Baby trials, IRD and community-based seed production and distribution and dissemination activities have been duly acknowledged. Special thanks go to District Agriculture Development Offices of all the terai and selected hill districts for collaboration in field research. Staff of LI-BIRD and NRRP and members of Jaskelo Youth Club, who conducted these research activities over a long period, are highly appreciated. We thank Drs D. S. Virk, K.A. Steele and D. Harris of CAZS NR for their help in analysis and interpretation.

8. References

Central Bureau of Statistics (CBS). 2003. Statistical Year Book of Nepal. National Planning Commission Secretariat, HMG/N.

Chaudhary, B., M. Yadav, RB. Yadav, DK. Chaudhary, PCP. Chaurasiya, RN. Chaudhary, BN. Mahato and DN. Sah. 2004a. Evaluation of rice genotypes for resistance to blast disease. Paper presented in 24th Summer Crop Workshop, NARC, Kathmandu.

Chaudhary, B., M. Yadav, T. Akhtar, RB. Yadav, DB. Gharti and D. Bhandari. 2004b Evaluation of rice genotypes for resistance to bacterial blight. Paper presented in 24th Summer Crop Workshop, NARC, Kathmandu.

FAO, 2004. FAO Statistics Nepal. [http://www.riceweb.org/countries/nepal.htm]

Giri, T.P. and Shahi, B.B. 1978. Performance of aromatic rice varieties in Nepal, pp. 35-36. In: Proceedings of 5th Rice Improvement Workshop, 27 February to 2 March 1978pp. Parwanipur, Nepal: National Rice Improvement Programme, Department of Agriculture, Ministry of Food, Agriculture and Irrigation.

Gyawali, S., Joshi, K.D. and J R Witcombe 2002. Participatory plant breeding in rice in low altitude production system in Nepal. In: Witcombe J R, Parr L B, Atlin G N (eds.) (2002) Breeding rainfed rice for drought prone environments: Integrating conventional and participatory plant breeding in South and Southeast Asia: Proceedings of a DFID Plant Sciences Research Programme/IRRI Conference, 12-15 March 2002, IRRI, Los Banos, Laguna, Philippines. Department for International Development (DFID) Plant Sciences Research programme (PSP), Centre for Arid Zone Studies (CAZS) and International Rice Research Institute (IRRI), Bangor and Manila.

IRRI 2005 World rice facts Los Baños, Philippines: International Rice Research Institute.

IRRI, 2005. World rice facts. International Rice Research Institute. Los Baños, Laguna, Philippines.

Joshi, K.D, Sthapit, B.R., Vaidya, A., Kadayat, K.B., Tuladhar, J.K., Subedi, K.D. and Lohar, D.P. 1997. Findings of postal survey and rapid rural appraisal on aromatic and fine grain rice in Pokhara valley of Nepal: Issues for future research. LARC Working Paper no. 97/2. Pokhara, Kaski, Nepal: Lumle Agricultural Research Centre.

M. Joseph, S. Gopalakrishnan, R.K. Sharma, V.P. Singh, A.K. Singh, N.K. Singh and T. Mohapatra (2004) Combining bacterial blight resistance and Basmati quality characteristics by phenotypic and molecular marker-assisted selection in rice. Molecular Breeding 13 (4):377-387

MoAC 2004. Statistical information on Nepalese Agriculture (2003-04). His Majesty's Government of Nepal, Ministry of Agriculture and Cooperatives, Agri-Business Promotion and Statistics Division, Singh Durbar, Kathmandu, Nepal.

NRRP 1997. *25 years of Rice Research in Nepal (1972-1977)*. Parwanipur Bara, Nepal: National Rice Research Programme, Nepal Agricultural Research Council.

NRRP, 1997. 25 years of Rice Research in Nepal (1972-1977*)*. Parwanipur Bara, Nepal: National Rice Research Programme, Nepal Agricultural Research Council.

Puri, K.D. and Shrestha, S.M. 2005. Reaction of breeding lines/cultivars of rice against blast (*Magnaporthe grisea*) in field and laboratory conditions. Technical Report submitted to LI-BIRD by a Masters's Degree student at IAAS as a part of Mater's Thesis.

Rana R B, Joshi K D, Gyawali S and Witcombe J R 2004. Participatory crop improvement project of Chitwan and Nawalparasi districts of Nepal. A baseline report. Discussion Paper no. 5. CAZS Natural Resources and Local Initiatives for Biodiversity, Research and Development (LI-BIRD)

Sah, R.P. (Ed) 2002. Development of fine and aromatic rice varieties for the hills of Nepal. Final Technical Report, HARP PP-09/99. Pokhara, Kaski, Nepal: Agricultural Research Station, Lumle.

Snapp, S., 1999. Mother and baby trials: a novel trial design being tried out in Malawi. In TARGET. The Newsletter of the Soil Fertility Research Network for Maize-Based Cropping Systems in Malawi and Zimbabwe. Jan. 1999 issue. CIMMYT, Zimbabwe.

Steele, K. A., Edwards, G., Zhu, J., and Witcombe, J.R. 2004. *Theor. Appl. Genet.* **109**, 1247

Witcombe J R, Joshi K D, Rana R B and Virk D S 2001. Increasing genetic diversity by participatory varietal selection in high potential production systems in Nepal and India *Euphytica* 122 575-588

Annexure 1. Professionals and farmers involved in the development of Sunaulo Sugandha

Institutions	Professionals and farmers	Designation
LI-BIRD	Mr. KP Devkota, Mr. MP Tripathi, Mr. S Gyawali, , Mr. M Chaudhary, Mr. M Gurung, Mr. Hari Poudel, Mr. K Rawal, Mr. Dev R Karki, Mr. D Poudel, Mr. P Shrestha, Mr. KB Limbu, Mrs R. Kadariya, Mr. N. Chaudhary, Mr. M Subedi, Ms S Sunwar, Ms Durga Pandit, Ms. Parbati Neupane, Mr. Bhim Thanet, Mr. Hari Ghimire, Mr. S Khanal, Mr. B Gadal, Dr. A Subedi and Dr. PK Shrestha,	Plant Breeder, Asst. Plant Breeder, Project Coordinator, Community Organizers, Field Motivators
CAZS Natural Resources, University of Wales Bangor, UK	Prof. John R Witcombe, Dr. Krishna D Joshi; Prof. Dr. Daljit Singh Virk	Plant Breeder, Biometrical Geneticist
NRRP/NARC	Mr. B Chaudhary, Mr. D Chaudhary, Mr. T Akhtar, Dr. N Adhikari, Dr SP Khatiwada, Mr. RB Yadav	Plant Pathologists, Plant Breeders, Agronomists, Plant Breeders, Scientists
DADO, Chitwan	Mr. Chandra K Devkota, Dr. Suroj Pokhrel, Mr. Mahesh Regmi, Pawan Giri, Ishowari Neupane	Extension Specialists
IAAS, Rampur	Mr. Krishna Datta Puri, Dipak Sharma Poudel	Lecturer and Plant Pathologist
DADOs of terai and inner terai districts	Chitwan, Nawalparasi, Bara, Rautahat, Kailali, Kanchanpur, Dang, Saptari, Dhanusha, Mahottari, Jhapa, Morang, Sunsari, Siraha, Sarlahi, Parsa, Makwanpur, Kapilbastu, Banke, Bardiya, Kaski, Palpa and Baglung.	Agronomists, Extension Officers, JT and JTAs
Chitwan	Mr. Dev R Sapkota, Mr. Chudamani Sapkota, Mr. Krishna Chapagain, Mr. Megh Prasad Devkota, Mr. Chirinjibi Pokhrel, Mr. Punya Prasad Adhikari, Krishna Bhatta, Hari Khadka, Mr. Ganga Neuapne.	Leader farmers
Chitwan	Mr. Dev Raj Sapkota, Mr. Chudamani Sapkota, Mr. Krishna Chapagain, Mr. Deepak Subedi	Jaskelo Youth Club and Dev Ujjal Seed Producers Group

Annexure 2. Coordinated Varietal Trial (CVT)-Rainfed Lowland Medium 2004 and 2006 Yield (kg/ha)

	2004			2006		Mean	
Genotype	Hardinath	Parwanipur	Mean	Hardinath	Parwanipur		Grand mean
Barkhe 2001	2912	2879	2895				2895
Barkhe 2014				2949	2307	2673	2673
Barkhe 2045	1821	1094	1458				1458
Barkhe 3004	3470	2900	3185				3185
Barkhe 3019				3887	1856	3016	3016
IR 62558-SRN-17-2	2488	3062	2775				2775
IR 73435-66				2568	2119	2375	2375
IR 73435-83				2516	3331	2865	2865
Makwanpur 1	3847	3432	3639				3639
**Masuli**	**3187**	**2479**	**2833**		**1927**	**1927**	**2586**
NR 1190-24-4-2	3357	3166	3261				3261
NR 1887-4-3-1-1	2579	1483	2031				2031
NR 1887-81-1-2-2	3023	2973	2998				2998
NR 1887-8-1-1-2-2				3625	1748	2821	2821
NR 1892-20-21-1-1	2814	3160	2987				2987
NR 1893-17-2-3	3090	3126	3108	3682	2178	3037	3075
NR 1894-10-3-2-3	3225	3103	3164	3664	3036	3395	3272
NR 1988-2-3-5				2732	2377	2580	2580
NR 1988-9-1-2-4-3				3111	1727	2518	2518
NR 1988-9-1-2-4-4				2428	1932	2215	2215
NR 268-4-6-4	2430	3247	2839				2839
PSBRC 70	2129	2008	2068				2068
Radha 11					2048	2048	2048
Radha 4				4058		4058	4058
Rambilas	2955	2478	2716	3463		3463	2965
**Sunaulo Suganda**	**2620**	**2704**	**2662**	**4112**	**2101**	**3250**	**2936**
Super 3004				3436	2933	3220	3220
WAR 120-1-5				3111	1848	2570	2570
WITA 4				3060	2957	3016	3016
Grand mean	2872	2706	2789	3275	2277	2847	2816
LSD (p=0.05)	998	534	Ns	1057	926	997	
p level	0.023	<0.001	0.101	0.023	0.015	ns	

Annexure 3. Yield (t ha-1) performance of Sunalulo Sugandha in Mother trials in low land environment of Chitwan and Nawalparasi, 2004 to 2006

Genotype	2004	2005	2006	Grand mean
Barkhe 3004	3.011	4.241	3.968	3.920
Barkhe 3015	1.917			1.917
Barkhe 3017	2.102	2.552		2.417
Barkhe 3018		4.284	4.071	4.159
Barkhe 3019		4.662	4.249	4.419
Masuli	2.481	3.315	3.280	3.172
NR 1190			3.575	3.575
Sugandha 2002	3.197	3.782	3.138	3.372
Super 3004	4.419	4.221	4.138	4.209
Grand mean	2.855	3.865	3.774	3.686
LSD (p=0.05)	1.37	0.72	0.503	
p level	0.02	<0.001	<0.001	

Note: Data from 3, 7 and 10 mother trials in 2004, 2005 and 2006 respectively in Chitwan and Nawalparasi districts

Annexure 4. Yield (t ha-^{1}) performance of Sunalulo Sugandha in aromatic Mother trials in Chitwan and Nawalparasi, 2006

Table: Mean performance of Sunalulo Sugandha in aromatic mother trials in Chitwan and Nawalparasi districts during main season 2006 and 2007 (n=9)

Genotype	Maturity (days)	Tillers (no/hill)	Plant height(cm)	Grain yield (t ha-^{1})
Barkhe 3019	151.40	8.91	106.0	3.59
Lalka Basmati	159.60	7.77	141.2	2.04
Masuli	152.33	6.97	134.0	2.87
Sunaulo Sugandha	155.67	6.73	110.0	3.57
Super 3004	152.33	9.07	106.2	3.74
Grand mean	3.762	7.89	119.5	3.16
F test	**	**	**	**
CV (%)	3.5	26.6	9.0	39.9
LSD	154.27	0.944	4.08	0.891

Annexure 5. Agronomic traits of Sunaulo Sugandha and other main season rice varieties in CVT rainfed lowland medium (RLM) in Hardinath, Parawanipur and Itahari since 2004-2006 and mother trial data from the trials conducted by LI-BIRD, FORWARD and CDRC in Jhapa (5), Morang (3), Sunsari (5), Saptari (6), Siraha (5), Chitwan (26), Nawalparasi (15) and Kapilbastu (3) from 2004 to 2006.

	No. of trials				Days to heading			Days to maturity				Plant height			
Genotype	2004	2005	2006	Total	2005	2006	Mean	2004	2005	2006	Mean	2004	2005	2006	Mean
Barkhe 2001	23			23				129			129	115			115
Barkhe 2014	11	20	21	52	121	112	118	136	148	144	143	123	112	106	112
Barkhe 2024	10	12	12	34				140	144	141	141	100	102	87	96
Barkhe 3004	19	18	10	47	111		111	155	156	155	155	98	95	96	96
Barkhe 3019	1	10	17	28	71	125	98	149	153	155	154	93	102	100	101
BPI-3-2		12	4	16					141	140	140		103	96	101
CNTLR		8		8					116		116		96		96
Gajale Masino			17	17						153	153			153	153
IR 62558-SRN-17-2	12			12				148			148	99			99
Jhapali Masuli		16	15	31	108		108		142	144	143		113	106	110
Lalka Basmati			15	15						159	159			144	144
**Masuli**	**15**	**24**	**18**	**57**	**126**	**116**	**123**	**150**	**152**	**150**	**151**	**132**	**125**	**124**	**125**
NR 1190		12	10	22					145	152	149		139	131	135
NR 1190-24-4-2	12			12				142			142	121			121
NR 1887-4-3-1-1	12			12				142			142	143			143
NR 1887-81-1-2-2	12			12				144			144	100			100
NR 1887-8-1-1-2-2		8	7	15	117	111	115		149	147	148		107	111	109
NR 1892-20-21-1-1	12			12				150			150	87			87
NR 1893-17-2-3	12	8	7	27	129	119	126	150	162	148	155	87	88	110	97
NR 1894-10-3-2-3	12	8	7	27	132	123	129	159	164	154	159	99	107	116	110
NR 268			14	14						149	149			125	125
NR 268-4-6-4	12			12				147			147	126			126
OR 367		12	14	26					143	145	144		100	85	93
PSBRC 70		8	3	11				158			158	90			90
Radha 17	17	8	15	40				149			149	126			126
Rambilas	11		3	14	140		140	152	167	154	159	109	114	106	110
Sabitri	17	7	1	25				139		140	139	103		98	102
**Sunaulo Sugandha**	**7**	**10**	**17**	**34**	**72**	**120**	**96**	**152**	**153**	**153**	**153**	**111**	**112**	**101**	**107**
Super 3004			7	7	74	123	99	156	152	153	154	109	104	101	103

Contd.

Genotype	Panicle length (cm)			
	2004	2005	2006	Mean
Barkhe 2001				
Barkhe 2014		21	24	22
Barkhe 2024				
Barkhe 3004	24	23		23
Barkhe 3019	24	24	26	25
BPI-3-2				
CNTLR				
Gajale Masino				
IR 62558-SRN-17-2				
Jhapali Masuli		21		21
Lalka Basmati				
**Masuli**		**22**	**24**	**23**
NR 1190				
NR 1190-24-4-2				
NR 1887-4-3-1-1				
NR 1887-81-1-2-2				
NR 1887-8-1-1-2-2		22	22	22
NR 1892-20-21-1-1				
NR 1893-17-2-3		22	25	23
NR 1894-10-3-2-3		23	27	24
NR 268				
NR 268-4-6-4				
OR 367				
PSBRC 70				
Radha 17				
Rambilas		21		21
Sabitri				
**Sunaulo Sugandha**	**26**	**26**	**28**	**27**
Super 3004	23	23	25	24

Cont.

Genotype	Filled grains /5 panicles 2005	Unfilled grains /5 panicles 2005	1000 grain weight (g) 2004	2005	Mean	Tillers/hill 2004	2005	2006	Mean
Barkhe 2001			21		21	9			9
Barkhe 2014	533	167		22	22	9	10	9	9
Barkhe 2024						10	9	8	9
Barkhe 3004	435	221	23	24	24	8	10	10	9
Barkhe 3019			22		22		9	10	10
BPI-3-2							10	7	9
CNTLR							9		9
Gajale Masino								8	8
IR 62558-SRN-17-2			26		26				
Jhapali Masuli	661	151		17	17		9	9	9
Lalka Basmati								8	8
**Masuli**	**740**	**187**	**17**	**18**	**18**	**7**	**7**	**9**	**8**
NR 1190							8	8	8
NR 1190-24-4-2			24		24				
NR 1887-4-3-1-1			19		19				
NR 1887-81-1-2-2			19		19				
NR 1887-8-1-1-2-2	449	263		20	20				
NR 1892-20-21-1-1			21		21				
NR 1893-17-2-3	587	232	22	22	22				
NR 1894-10-3-2-3	536	240	27	26	26				
NR 268								8	8
NR 268-4-6-4			18		18				
OR 367							10	9	9
PSBRC 70			28		28				
Radha 17						6			6
Rambilas	586	266	20	19	19				
Sabitri						9		8	9
**Sunaulo Sugandha**	**840**		**19**		**19**	**8**	**10**	**8**	**8**
Super 3004			23		23	9	10	12	10

Cont.

Genotype	Tillers/m2 2004	2005	2006	Mean
Barkhe 2001	281			281
Barkhe 2014		263	271	267
Barkhe 2024				
Barkhe 3004	293	250		259
Barkhe 3019	288	288	290	289
BPI-3-2				
CNTLR				
Gajale Masino				
IR 62558-SRN-17-2	288			288
Jhapali Masuli		306		306
Lalka Basmati				
**Masuli**	**286**	**262**	**231**	**259**
NR 1190				
NR 1190-24-4-2	260			260
NR 1887-4-3-1-1	210			210
NR 1887-81-1-2-2	309			309
NR 1887-8-1-1-2-2		269	251	260
NR 1892-20-21-1-1	291			291
NR 1893-17-2-3	294	258	283	272
NR 1894-10-3-2-3	293	253	285	271
NR 268				
NR 268-4-6-4	230			230
OR 367				
PSBRC 70	247			247
Radha 17				
Rambilas	318	267	269	278
Sabitri				
**Sunaulo Sugandha**	**277**	**284**	**266**	**272**
Super 3004	295	295	262	274

Annexure 6. Grain yield (t ha $^{-1}$) of Sunaulo Sugandha from crop cut survey of Baby trials from various districts of Nepal, 2006

Region	District	N	Farmer's variety	Farmer's variety†	Sunaulo Sugandha†	Yield advantage over farmer's variety (%)	t-at 0.05
Central Terai	Chitwan	54	Barkhe 2014, Barkhe 3004, BG 1442, Makawanpur 1, Masuli, OR 367, Sabitri, Samba Masuli, Swarna	3.99 ±0.17	4.31± 0.18	8	*
	Makawanpur	7	Makawanpur 1	3.09 ±0.20	3.39 ± 0.17	10	*
	Rupandehi	3	Gorakhnath 509, Masuli, Radha 4	4.03±0.33	3.57±0.65	-11	NS
	Mean	62		3.80±0.15	4.09±0.16	7.45	*
Eastern Terai	Mahotari	3	Not available	3.50±0.35	5.50±0.13	57	***
	Morang	9	Basmati, Kanchhi Masuli, Masuli	3.58±0.24	3.89±0.22	9	NS
	Mean	12		3.56± 0.19	4.30±0.27	21	*
Hilly area	Lamjung	15	Chhote, Ekle, Khumal-4, Madhesi, Pahele, Pokhreli Masino, Sabitri	3.22 ±0.15	4.71±0.23	46	***
	Tahanun	2	Madhesi, Masuli	3.75± 0.75	3.1250±0.12	-17	NS
	Udayapur	10	Bans Dhan, Basmati, Dumsi, Harda, Kalo Basmati, Khemti, Masuli, Radha 12	1.82±0.27	2.50±0.15	38	***
	Mean	27		2.74±0.19	3.77±0.25	38	***
	Grand mean	101		3.55±0.20	4.09±0.12	15	***

†Mean ± SE

Annexure 7. Various rice varieties being replaced by Sunaulo Sugandha in 8 districts (as mentioned in Annexure 6) of Nepal.

Variety replacing	Frequency	Variety replacing	Frequency
Farmers varieties	41	Pokhreli	2
Sabitri	28	Radha 12	2
Masuli	19	Barkhe 2001	1
OR 367	12	Barkhe 3004	1
Aus Masuli (Bansdhan)	9	Chaite 2	1
Radha 4	7	Dumsi	1
Barkhe 2014	4	Ekle	1
Basmati	4	Goraknath 509	1
Sona Masuli	4	Harda	1
Madeshe	3	Himali	1
Pahele	3	Jarneli	1
Samba Masuli	3	Khemti	1
Swarna	3	Khumal 4	1
BG 1442	2	Makawanpur 1	1
Kalo Basmati	2	Total	160

Annexure 8.0 Cost-benefit analysis of Sunaulo Sugandha and Masuli Based on CVT, Mother and Baby trial yield data

Cost items	Sunaulo Sugandha	Masuli
**Expenditure on cultural operations (Labour cost)**		
Nursery seed bed preparation	400	400
Fertilizer N, P and K for nursery bed	125	125
Land preparation	3640	3640
Fertilizer /Manure application	500	500
Transplanting	3000	3000
Weeding	3000	3000
Insecticide/pesticide spraying	600	600
Harvesting	3000	3000
Threshing	2500	2500
Cleaning, packaging	1250	1250
**Subtotal**	**18015**	**18015**
**Expenditure on inputs**		
Seed	1100	1100
Fertilizer		
DAP	2349	2349
UREA	1735.92	1735.92
MOP	900	900
Micro-nutrient	1000	300
Fungicide	500	500
Insecticide	1000	500
**Sub-total**	**8584.92**	**7384.92**
**Total cost**	**26599.92**	**25399.92**
**Return**		
Grain[§]	68580	43810
Straw	11875	11875
**Total return**	**80455**	**55685**
**Net profit**	**53855.08**	**30285.08**
**Benefit cost ratio**	**2.025**	**1.192**

[§] Average grain yield of Sunaulo Sugandha from 58 trials in CVT (n=11), mother trials (n=20) and baby trials (n=101) data.

Annexure 9. National Rice Disease Nurseries for blast, 2003.

EN	DESIGNATION	Leaf blast score (0-9)			
		NRRP/H	NORP/N	PPD/K	RARS/T
24	CNTLR85085-78-1-1-1	0	1	1	0
36	Rato Basmati	0	1	2	1
170	BPI 3-2	-	1	2	0
**226**	**Barkhe-3004**	**0**	**1**	**2**	**0**
232	Sugandha-1	0	1	1	0
233	Sugandha-2002	-	1	2	1
	Laxmi (Resistant Check)	*1*			
	Masuli (Susceptible Check)	*9*			

Source: Chaudhary *et al.*, 2004.

Annexure 10. National Rice Disease Nurseries for blast and bacterial leaf blight, 2004.

Variety	2004 Blast	2004 BLB	2005 Blast	2005 BLB
Sugandha 2002		3	0	7
Sugandha 1			0	7
CNTLR 85033-9-3-1-1	0	7		
CNTLR85085-78-1-1-1	NG	7		
Rato Basmati	0	5		
Pusa sugandha-2	0	9		
Gan-wan-xian 22	0	3		
Pusa 834	0	3		
Resistant Check (Laxmi)	1		1	
Resistant Check (??)		3		2
Susceptible Check (Masuli)	8		8	
Susceptible Check (Masuli??)		9		9

Source: Bedananda *et al,* 2005.

NRRP/H= National Rice Research Program, Hardinath; NG=Not germinated; PG=Poor germination

Annexure 11. Incidence and average blast scores in rice varieties screened in the PVS trials in Chitwan, 2005

Rice variety	Leaf blast score (0-9)[§]		Neck blast score (0-9)	Severity/incidence	
	Krishna Mandir	Gitanagar	Krishna Mandir	Leaf blast	Neck blast
Sugandha 2002	2	1	†	No/low	†
Masuli	5	6	4	High	High
Barkhe 3004	1	1	†	No/low	†

[§] Leaf and neck blast scoring was done using IRRI Standard Evaluation System (SES) for rice in 0-9 scale, mean of three replicates. For neck blast on the basis of severity of infection on panicle, 0 = no incidence, 1= <1%, 3 = 1-5%, 5-6 =25%, 7= 26-50%, 9= 51-100%. For severity, varieties are categorised as no = zero infection, low = predominance of 1-3 score and low severity, moderate = 4-6 and high = 7-9, [†]= information not available.

Source: Puri and Shrestha 2005

Annexure 12. Response of Sunaulo Sugandha and Masuli to rice insects observed in mother trials in on-farm trials in Chitwan in 2003 in Chitwan district by plant pathologist from IAAS, Rampur, Chitwan

	Masuli			Suanulo Sugandha		
Trial	1	2	3	1	2	3
Mealy bug	2	1	0	3	1	0
Stem borer	0	0	1	0	0	0
Leaf folder	0	0	1	0	0	0
