# Supplementary material for: Plant breeding can be made more efficient by having fewer, better crosses
Source: BMC Plant Biol. 2013 Feb 7;13:22. doi: 10.1186/1471-2229-13-22 (PMC3621587; doi:10.1186/1471-2229-13-22)
Supplement: Additional file 7 — New Rice Varieties for Bangladesh from Client-Oriented Breeding. Rainfed Agriculture Impact Assessment Study No.3. Monitoring Impact and Learning (MIL) Component of the Research into Use (RiU) Programme; 2008. [file 1471-2229-13-22-S7.pdf]

The impact of new maize and rice varieties on the livelihoods of poor farmers in marginal agricultural areas of western India

Yadavendra[1], J.P., Patel[2], V.P. & Witcombe[3] J.R.

[1]Gramin Vikas Trust, Dahod, Gujarat, India, [2]Anand Agricultural University, Anand, Gujarat, India, [3]CAZS Natural Resources, University of Wales, Bangor, UK.

Limited participation of farmers in agriculture research and extension can result in low uptake of new farm technologies including the use of modern varieties. Participatory approaches can be used to rapidly and cost effectively identify the best of already existing varieties in a process termed participatory varietal selection (PVS). However, new highly client-oriented breeding (COB) approaches can produce varieties better than those found by PVS. In COB varieties are bred to specifically meet the requirements of the client farmers.

The impact of a new maize varieties on the livelihoods of farmers was surveyed in a sample of farmers in three states of western India that had previously been given seed. The varieties were GM-6 developed through highly client-oriented breeding in India and ZM-421 introduced from Zimbabwe. They liked the new varieties for their higher yields as well as other traits such as improved grain quality and earlier maturity. The improvement on livelihoods because of the adoption of GM-6 was high. The adopting farmers were able to sell more grain and their household food self sufficiency also increased. In rice, new varieties Ashoka 200F and Ashoka 228 bred by COB in eastern India were preferred by almost all farmers for many traits including their higher grain yield. Farmers reported that food grain self-sufficiency increased.

All these undoubted impacts on livelihoods were obtained by farmers who had received seed through rural development projects. However, the challenge still remains of producing a sustainable and affordable seed supply system for farmers in marginal areas.

The choice of research on improved seeds is discussed. The new varieties give direct advantages from their increased productivity as well as providing additional benefits for the farming system and the rural economy. Genetic improvement is a highly cost effective intervention but without water the benefits from new varieties will greatly decline in drought years. Mechanisation by two-wheeled tractors has also been researched in the project and it can improve water availability.

Keywords: client oriented breeding, livelihoods, impact assessment, rice, maize

Introduction

The Gramin Vikas Trust (GVT) has a research component that includes the improvement of crops grown by the indigenous (tribal) populations in the hill districts of western India in Gujarat, Madhya Pradesh and Rajasthan. The rainy season crops in the research include maize (*Zea mays* L.), rice (*Oryza sativa* L.), blackgram (*Vigna mungo* L.) and horsegram (*Macrotyloma uniflorum* L.) that together occupy most of the cultivated area in project districts (Figure 1). Maize and rice are the two most important rainy season cereal crops. The crops are grown on low fertility soils on undulating land where agriculture is a risky enterprise with severe droughts occurring on average once in five years.

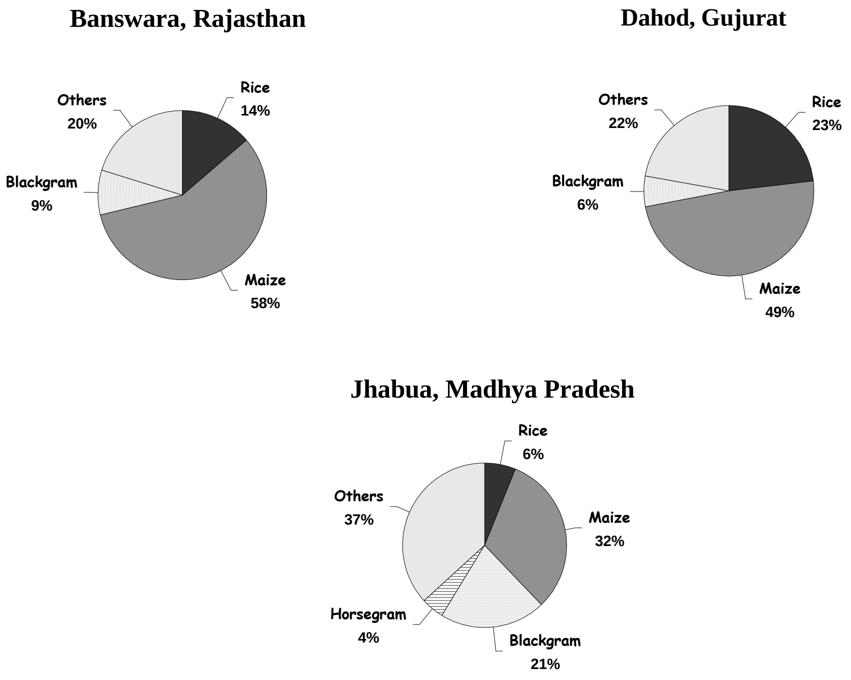

Figure 1. Crops (as percentage of total cropped area) in the three districts of the GVT project area, rainy season, 2004.

Existing research-extension approaches had not led to the use of modern varieties so most farmers were still growing landraces of these crops. A key assumption was made that the poor adoption of new varieties was not because of a lack of willingness to adopt new technologies, or even a lack of a good choice of varieties, but because low-resource farmers in remote areas had not had access to them. Participatory varietal selection is a farmer participatory approach for identifying improved crop cultivars or varieties (Witcombe et al., 1996). It was shown to be a rapid and cost effective process of identifying farmer-preferred cultivars in the GVT project area in western India (Joshi & Witcombe, 1996). However, varieties identified by PVS are pre-existing varieties that

have not been specifically bred to meet the requirements of the poor farmers in the target areas. A very good understanding of those requirements was gained from participatory rural appraisals and the PVS trials so maize and rice varieties were bred to meet those identified needs.

We report here on the impact of new maize and rice varieties in the Gramin Vikas Trust (GVT) project area.

Materials and Methods

The Gramin Vikas Trust is implementing the Western India Rainfed Farming Project funded by the Department for International Development (DFID), United Kingdom, Government of India, State Governments, Krishak Bharati Co-operative Limited (KRIBHCO). The project is working for sustainable livelihood enhancement of poor women and men in areas populated by indigenous (tribal) people covering about 1350 villages of 7 districts in Gujarat (Dahod and Panchmahals), Rajasthan (Banswara and Dungarpur) and Madhya Pradesh (Jhabua, Dhar and Ratlam).

In these districts, maize is cultivated on marginal soils under low-input management and rainfed conditions. Rainfed is low and erratic. The project area receives on an average 700 to 1100 mm of rains per year in an average of 30 to 40 rainy days. Droughts are frequent and on average in one year in five they are severe enough to cause widespread crop failure.

Surveys were conducted in the period from June to November 2004 (Table 1) by GVT staff or by staff of the State Agricultural Universities working in what is called Component C (the research component) of the GVT Western India Rainfed Farming Project. In some cases these surveys followed from initial ones that were made in February 2004.

The surveys were designed to study the take up by farmers of five project-developed varieties and only included farmers who were known to have had an opportunity to try them. Seed had been widely distributed by the project (Tables 2 and 3). Hence, surveys were made in villages of the GVT project where the project had been working for several years. In total, 294 farmers were interviewed in a total of 40 different villages (11 in Gujarat, 16 in MP and 13 in Rajasthan).

The farmers that were interviewed on maize (Table 4) were asked questions on two varieties: GM-6, bred in India (Witcombe et al., 2003); and ZM-421, introduced into India from the International Centre for Maize and Wheat Improvement (CIMMYT), Zimbabwe. Farmers were asked questions about the level of adoption, the variety’s traits and details on seed that they had distributed.

Farmers interviewed on rice (Table 5) were asked similar questions as to the maize. The rice varieties examined were Ashoka 200F and Ashoka 228 bred using client-oriented techniques (Virk et al., 2003) and Kalinga III that had been identified by PVS (Joshi and

Witcombe, 1996) and had spread rapidly from farmer-to-farmer in the project area (Witcombe et al., 1999).

The surveys were done by using a structured questionnaire and the data were entered and analysed in Excel spreadsheets.

Table 1. Summary of the surveys conducted in GVT project villages in 2004.

Name of intervention	State[1]	Organisation conducting survey[2]	Survey month	Farmers interviewed (number)	Villages (number)
Rice	Guj	AAU	July	52	5
		GVT	June	21	4
	MP	JNKVV	June-July	47	9
		GVT	June	15	5
	Raj	MPUAT	July	30	7
		Total		165	30
Maize	Guj	AAU	August	24	4
		GVT	June	22	6
	MP	JNKVV	June-Aug.	25	6
		GVT	August	26	4
	Raj	MPUAT	April-June	33	6
		Total		130	26

[1]Guj = Gujarat; MP = Madhya Pradesh; Raj = Rajasthan.
[2]AAU = Anand Agricultural University; JNKVV = Jawaharlal Nerhu Krishi Vishwa Vidhyalaya; MPUAT = Maharana Pratap University of Agriculture and Technology.

Table 2. Seed of maize varieties distributed in the GVT project area from 2001 to 2003.

	Seed distributed (t)	
Year	GM - 6	ZM - 421
2001	-	1.1
2002	15.3	1.4
2003	29.9	2.9
Total	45.2	4.4

Table 3. Seed of rice varieties distributed in the GVT project area from 2001 to 2004.
.

	Seed distributed (t)		
Year	Kalinga III	Ashoka 200F	Ashoka 228
2001	9.2	0.3	0.1
2002	10.4	22.4	4.3
2003	3.5	10.7	16.9
2004	0	3.0	6.1
Total	23.1	16.1	28.0

Table 4. Summary of the maize surveys conducted in GVT project villages in 2004.

State	Organisation conducting survey	Villages (number) Common Villages	Individual Villages	2000	2001	2002	2003	Total
GM-6								
Guj	AAU	2	2	-	-	20	1	21
Guj	GVT	2	4	-	2	3	11	16
MP	JNKVV	2	2	-	23	2	-	25
MP	GVT	2	4	8	6	11	1	26
Raj	MPUAT	0	6	-	-	15	18	33
	Total	4	18	8	31	51	31	121
ZM-421								
Guj	AAU	2	2			14	1	15
Guj	GVT	2	3			5	3	8
MP	JNKVV	0	4			12	-	12
MP	GVT	0	1			3	-	3
Raj	MPUAT	0	2			0	9	9
	Total	2	12			34	13	47

Table 5. Summary of the rice surveys conducted in GVT project villages in 2004.

State	Organisation conducting survey	Villages (number) Common Villages	Individual Villages	1999	2000	2001	2002	2003	Total
Ashoka	200F								
Guj	AAU	1	1				10	3	13
Guj	GVT	1	1				3	0	3
MP	JNKVV	1	4				5	8	13
MP	GVT	1	0				1	1	2
Raj	MPUAT	0	5				13	13	26
	Total	2	11				32	25	57
Ashoka	228								
Guj	AAU	1	4				18	3	21
Guj	GVT	1	1				4	0	4
MP	JNKVV	2	6				8	14	22
MP	GVT	2	0				1	6	7
Raj	MPUAT	0	5				13	13	26
	Total	3	16				44	36	80
Kalinga	III†								
Guj	AAU	1	3	1	2	5	15	0	23
Guj	GVT	1	3		1	8	9	0	18
MP	JNKVV	4	1	2	6	2	7	0	17
MP	GVT	4	0		2	1	5	0	8
Raj	MPUAT	0	7		20	8	2	0	30
	Total	5	14	3	31	24	38	0	96

†For Kalinga III, adoption data was collected from 94 farmers and perception data from 90 farmers.

Results and Discussion

Farmers' perceptions of the varieties

Farmers perceptions on both of the maize varieties were favourable with significantly more farmer preferring both varieties for grain yield, fodder yield and eating quality (Figure 1) than those that preferred the local variety. However, the later variety ZM-421 was perceived by farmers to be of the same maturity as the local check as opinions were equally divided on it being later or earlier. Although a considerable proportion of the farmers reported that the varieties had better eating quality this was not reflected in market price where universally the varieties were reported to fetch the same price as the local variety. This reflects the market where grain merchants and consumers do not distinguish between different qualities of white maize.

All three rice varieties were markedly preferred by farmers for the important traits of early maturity, high grain yield and high grain quality (Figure 2). However, all three were inferior for fodder production. Surprisingly, given the greatly higher yield of the Ashoka varieties compared to Kalinga III they did not score much better for this trait – the only difference was that more farmers reported that Kalinga III yielded less grain than the local. However, the superiority of the Ashoka varieties was reflected in the adoption data (see below).

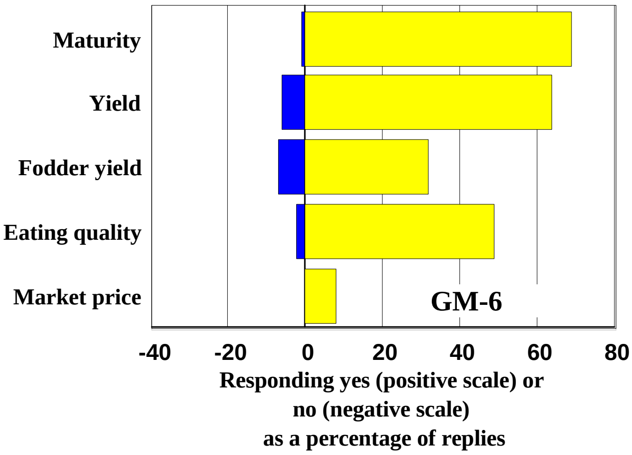

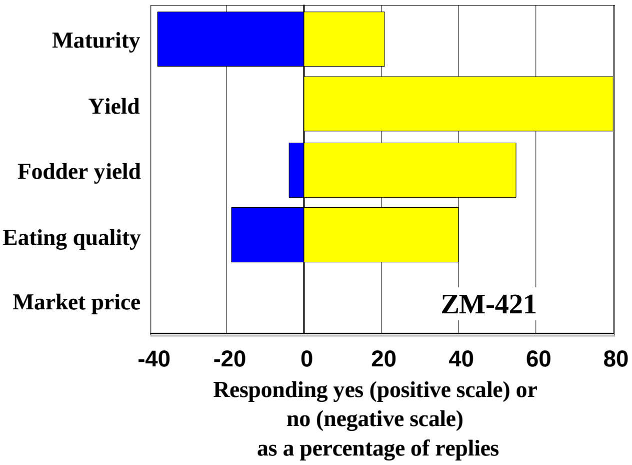

Figure 1. Summary of farmers' perceptions of two maize varieties GM-6 and ZM-421 relative to the local checks.

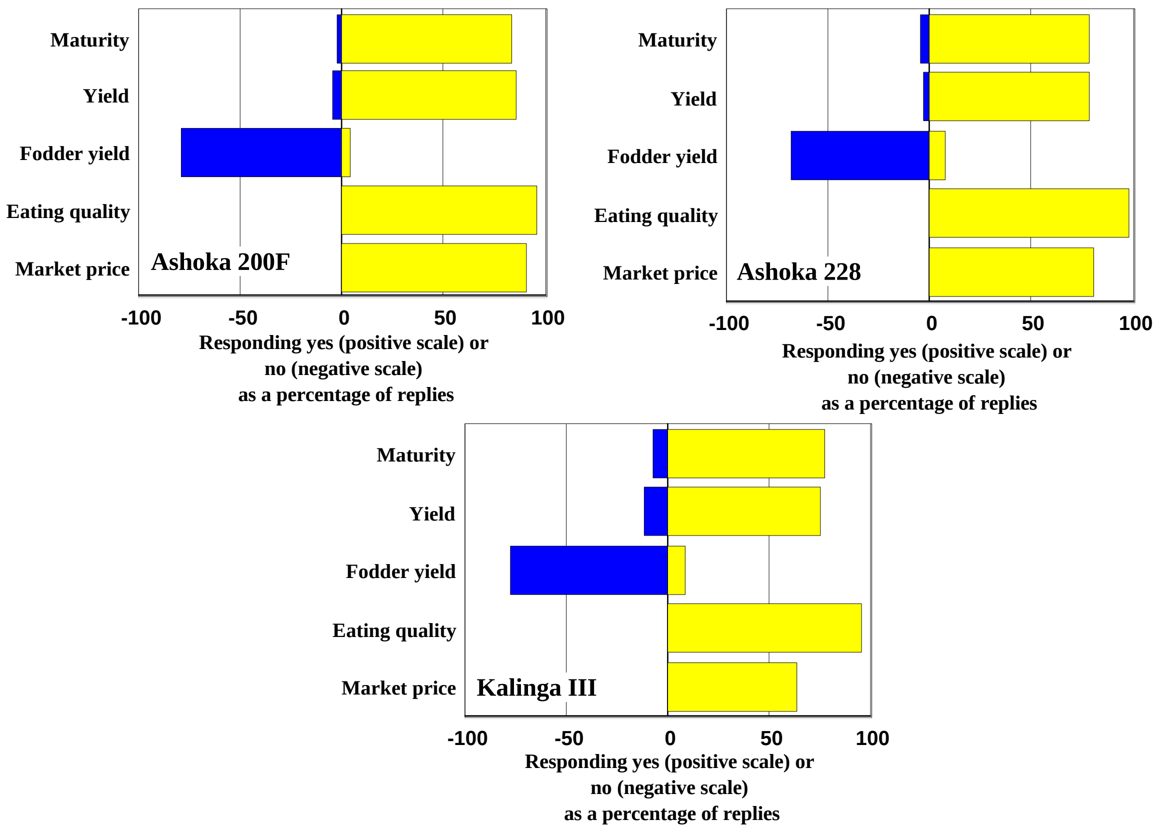

Figure 2. Summary of farmers' perceptions of three rice varieties, Ashoka 200F, Ashoka 228 and Kalinga III relative to the local checks.

Adoption of the new maize and rice varieties

All the farmers given access to seed of GM-6 grew it in the first year in 2001 (Figure 3). After the first year a proportion of the farmers decided to no longer grow the variety. However, those that did decide to grow the variety again became continuing adopters and grew it on an increasing proportion of their maize land (Figure 3). The most probable explanation for this pattern of adopters is that some farmers did not prefer the variety (Figure 1) and that some farmers who did prefer it had not saved seed. Once farmers had grown it for a second time, all were convinced of its advantages.

The acceptance of Ashoka 200F and Ashoka 228 was extraordinarily high with nearly all of the farmers continuing to grow it (Figure 4). They did so on a rapidly increasing proportion of their land. Farmers replaced Kalinga III with these new varieties so the proportion of continuing adopters fell and the rate of increase in the amount of land slowed.

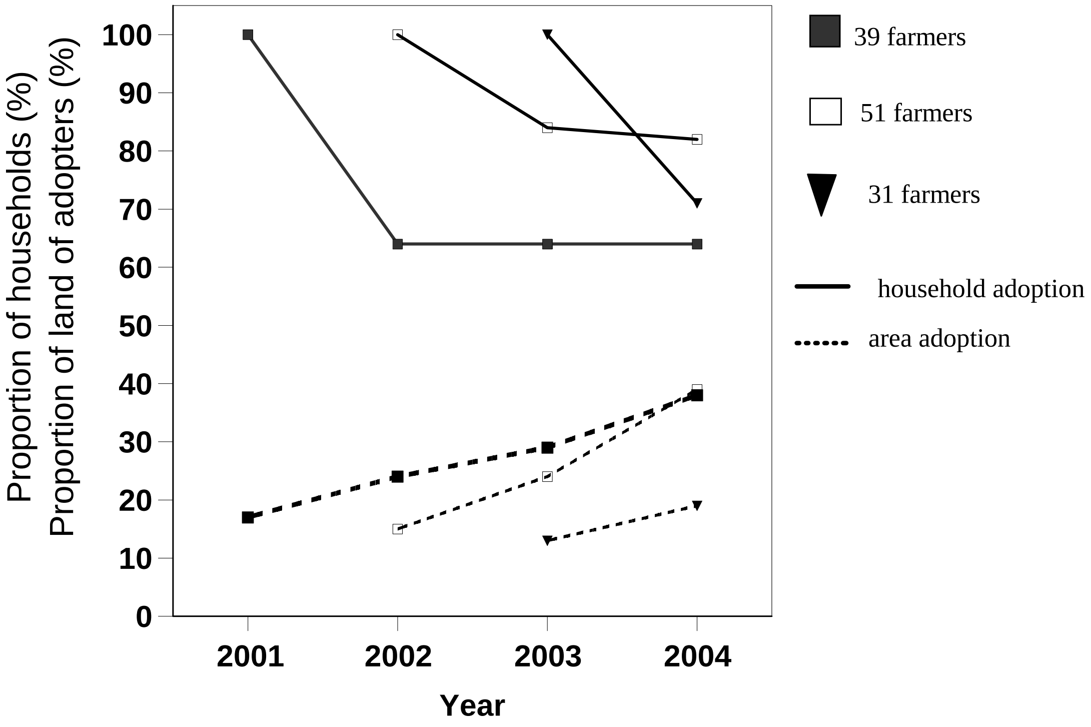

Figure 3. Adoption as a percentage of farmers who had been given seed of GM-6 and the proportion of maize land they devoted to this variety.

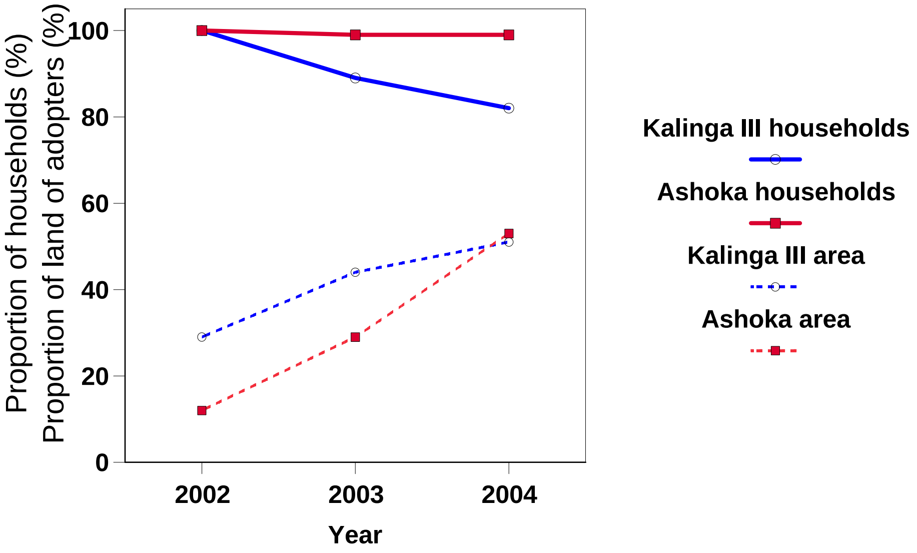

Figure 4. Adoption as a percentage of farmers who had been given seed of the Ashoka varieties (Ashoka 200F and Ashoka 228 combined) or Kalinga III and the proportion of rice land they devoted to this variety.

Impact of the new maize and rice varieties on livelihoods

Farmers reported considerable impacts on their livelihoods with seed sales in maize increasing by 51% and food self-sufficiency by more than one month. Over 70% of the farmers growing the new maize varieties reported an overall increase of more than 10% in their total income. The impact of the rice varieties was less as rice occupies, on average, a smaller proportion of the farmers' land. It is also more difficult for farmers to answer questions about food self sufficiency as rice is generally not the most important staple. However, this is a qualitative way of quickly examining the impact gave useful information without having to collect data on total household economics.

Table 5. Farmers' perceptions on the impact of the new maize varieties on their livelihoods.

Seed sales (t)		Food self sufficiency (months)		Impact on livelihoods (% total income)					
Before	After	Before	After	0 (no & %)	>0-10 (no & %)	10-20 (no & %)	20-30 (no & %)	30-40 (no & %)	>40 (no & %)
34	52	10.0	11.3	0	27	41	4	1	14
	+51%		+13%	0%	24%	47%	26%	<1%	12%

Table 6. Farmers' perceptions on the impact of the new rice varieties on their livelihoods.

Seed sales (t)		Food self sufficiency (months)		Impact on livelihoods (% total income)					
Before	After	Before	After	0 (no & %)	>0-10 (no & %)	10-20 (no & %)	20-30 (no & %)	30-40 (no & %)	>40 (no & %)
3.9	4.9	6.2	7.7	0	102	41	4	0	0
	+26%		+24%	0%	69%	28%	3%	0%	0%

Discussion

That the new varieties give benefits is beyond doubt. Farmers' favourable perceptions on the superiority of their traits, their rapid adoption by the majority of farmers that had access to seeds, and farmers' perceptions of the impacts on their livelihoods all confirm the benefits. This strongly suggests that the breeding of these new varieties and the provision of seed of them to farmers has been cot-effective development. Agriculture

remains important to the livelihoods of most households despite alternative sources of income such as the earnings from seasonal migration of some family members to large towns to work in the construction industry.

Increases in agricultural productivity can be achieved in many ways so is the provision of seed of improved varieties a sensible high priority? Ways of increasing benefits from agriculture are presented in Figure 5. This is a simplified illustration because, for example, improved agronomy includes many aspects such as mechanisation, plant protection, and agricultural practices such as intercropping and plant spacing. All these agronomic interventions require knowledge and many require the purchase of inputs although some, such as seed priming, can be very cheap and simple (Rashid et al. 2004; Harris et al. 2001).

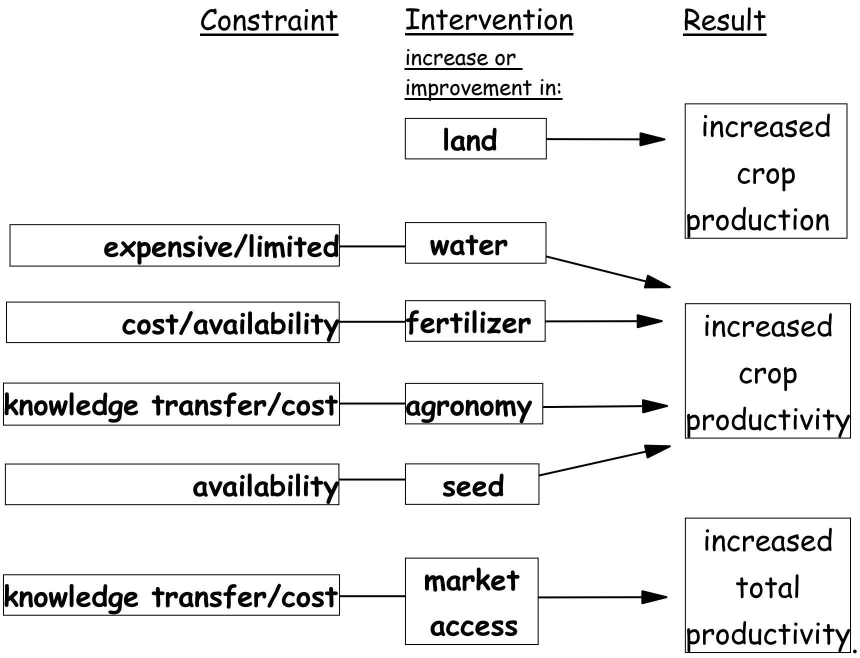

Figure 5. A simplified model of interventions that can improve crop production and productivity and their constraints to adoption.

In the GVT research component the choice of breeding new varieties was made because:

- The expenditure on breeding new varieties is much less than the cost of, for example, soil and water conservation work or the cost of providing improved agricultural knowledge to many farmers.
- Plant breeding research has a high potential cost benefit ratio (breeding and selecting new varieties is very inexpensive compared to the potential benefits).

- The cost benefit ratio of adopting new varieties is very high for farmers (the additional cost of seed can be zero if farmed-saved seed is used and the gains in yield can be substantial).
- The knowledge required to grow the new varieties is limited as they do not require changes in management practices.
- Increasing adoption of the technology (a new variety) can occur without the need of external interventions if the seed spreads from farmer to farmer.

Nonetheless, the price of food grains has been in continual decline so the value of increased crop productivity is less than in the past. However, the benefits of a new variety are not restricted to the value of the additional harvest (Figure 6). Additional benefits, i.e. multipliers, are obtained when increased cash contributes to off-farm economy or provides additional labour opportunities in agriculture. Farming system effects are also multipliers when it allows additional income from crop diversification. Farmers tend to prioritise production of their staple food and increased productivity can free up land for alternative, more profitable crops. Earlier maturing varieties can facilitate the timely harvest of post-rainy season (*rabi*) crops.

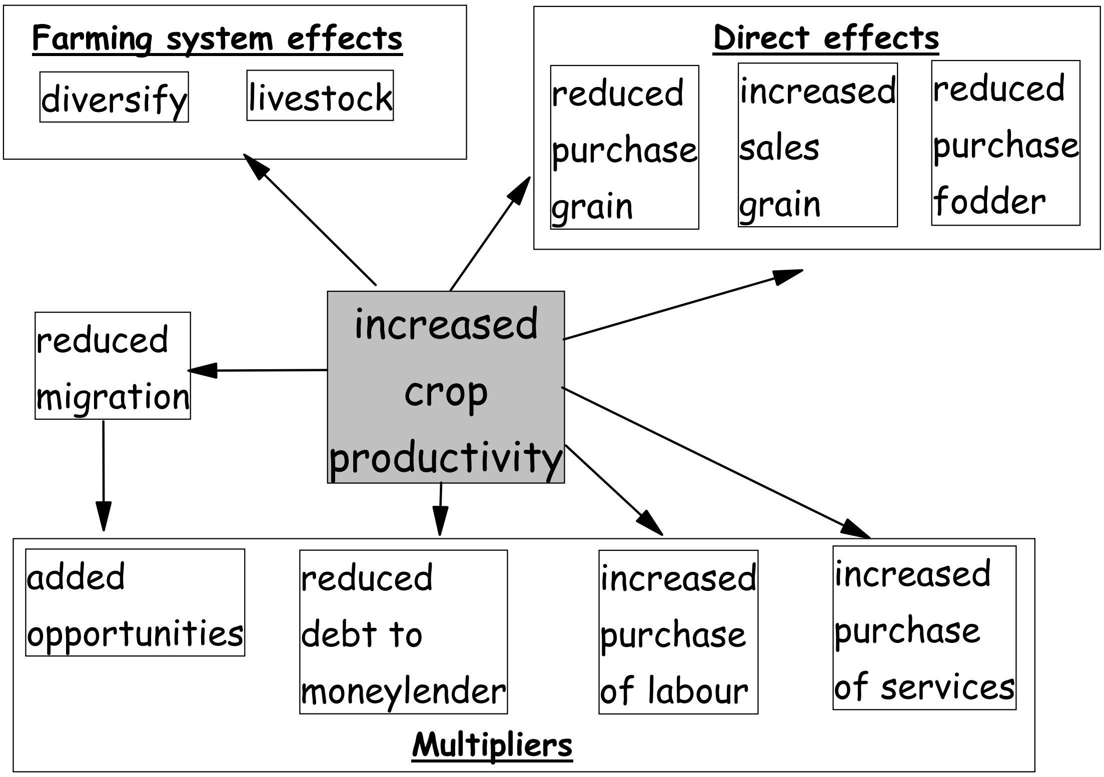

Figure 6. Impacts of increased productivity.

In a study on the impact of Ashoka 200F and Ashoka 228 in eastern India Mottram (2005) found their adoption had unexpected benefits such as reduced need to borrow money (Figure 7).

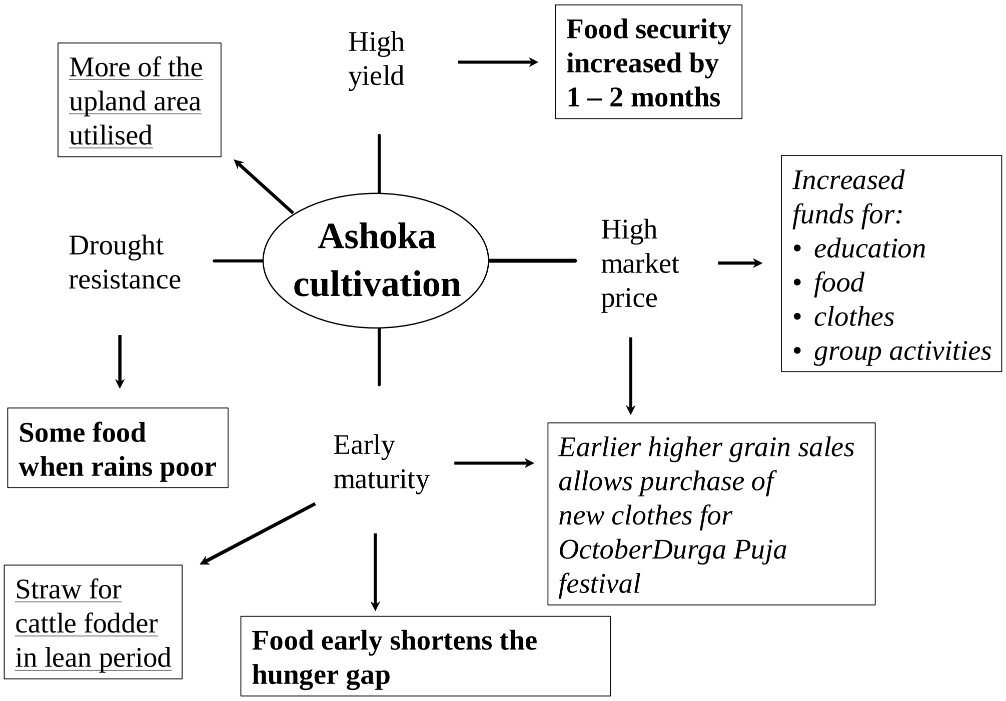

Figure 7. Impacts (shown in rectangles) of the adoption of Ashoka 200F and Ashoka 228 in eastern India. Direct effects (**bold**) *multiplier effects (italics) farming system effects* (<u>underlined</u>)*. Source A Mottram 2005.*

Even without multipliers, farmers in the study reported here have reported substantial improvements in their livelihoods. However, these impacts were reported only by farmers that had adopted a new variety. Clearly, if impact is to be large and widespread then adequate availability of seed is essential. Even though farmers reported distributing seed to others they only did so once they had satisfied the need of increasing the area on their own farms. Additional seed sources to farm-saved seed are needed if impact is to be rapid and is not to be set back by decreased seed availability resulting from poor harvests in drought years. Seed supply from the formal seed sector is not a solution as it does not serve the interests of poor farmers in remote villages. The formal sector:

- mainly produces high value certified seed and poor farmers have difficulty in affording its purchase,
- markets seed in areas remote from those of poorer farmers, and
- does not place priority on producing seed of varieties that are most appropriate for poor farmers e.g., open-pollinated varieties of maize or upland varieties of rice.

Additional forms of seed supply to the formal sector are required. GVT has facilitated community-based seed production and supply in the project area and, in the cases documented so far, it has been successful. This approach needs to be further developed and expanded as an alternative to conventional seed supply.

The impacts on livelihoods reported by farmers were in non-drought years. Drought is a major factor in productivity and the new varieties are more tolerant to droughted conditions. However, no matter how drought tolerant a new variety is there are biological limits to what breeding can achieve. In really severe droughts crop production is only possible if water is provided. Unfortunately, the rate of construction of water harvesting structures has been held back by an over-reliance on human labour because priority was given to providing waged employment over increased productivity. Mechanisations can greatly increase the speed and cost-effectiveness of constructing water harvesting structures such as check dams and tanks and greatly increase the productivity of human labour.

In the GVT research component two-wheeled tractors have been introduced into villages and, by using a trolley attachment, they can be used to shift stones and earth for water resource development work. The two-wheeled tractor offers a second way of increasing the availability of water. Farmer groups report that the water pump attachment to the two-wheeled tractor is much more easily transported so it can be used more widely in the village. It also provides more effective pumping than the common 5 hp diesel pump set. Hence, the villagers have been able to expand the area under irrigated crops and, where perennial water has been available, they have cultivated three crops a year. The tractor has thus produced the opposite of what is commonly feared; it has increased labour demand because of the increased cropped area and has reduced migration. In combination with higher yielding, higher quality crops mechanisation can increase overall productivity in a stable and sustainable way.

Acknowledgments

We wish to thank the many people who have made this work possible. A. Prasad, K.S. Sandhu, S.S. Rana, R. Sharma, S.K. Yadav, B.S. Raghuwanshi and D. Agrawal of GVT. S.N. Goyal, S. V. Sanghani, A.N. Metha and V.P. Patel of Anand Agricultural University. S.B.S. Tikka, N.B. Patel of Sardarkrishinagar Dantiwada Agricultural University. M. Billore, V.P. Rajput, H.C. Singhal of JNKVV. R. Pandya, P. Rokadiya, P. Choudhary and S.K. Kaushik of MPUAT.

References

Harris, D., B.S. Raghuwanshi, J. S. Gangwar, S. C. Singh, K. D. Joshi, A. Rashid and P.A. Hollington, 2001. Participatory evaluation by farmers of ‘on-farm’ seed priming in wheat in India, Nepal and Pakistan. Expl Agric. 37: 403-415.

Joshi, A. and J. R. Witcombe, 1996. Farmer participatory crop improvement II: Participatory varietal selection in India. Expl Agric. 32:461:477.

Mottram, A. 2005. Impact of New Upland Rice Varieties in Eastern India from Client-Oriented Breeding: Evidence from Whole Village Surveys. CAZS Natural Resources (CAZS-NR), University of Wales, Bangor, UK.

Rashid, A., D. Harris, P. A. Hollington and M. Rafiq, 2004. Improving the yield of mungbean (*Vigna radiata*) in the North West Frontier Province of Pakistan using On-Farm Seed Priming. Expl Agric. 40: 233-244.

Virk, D.S., D. N. Singh, R. Kumar, S. C. Prasad, J. S. Gangwar and J.R. Witcombe, 2003. Collaborative and consultative participatory plant breeding or rice for the rainfed uplands of eastern India. Euphytica 132: 95-108.

Witcombe, J.R., R. Petre, S. Jones and A. Joshi, 1999. Farmer participatory crop improvement. IV. The spread and impact of a rice variety identified by participatory varietal selection. Expl. Agric. 35: 471-487.

Witcombe, J.R., A. Joshi and S.N. Goyal, 2003. Participatory plant breeding in maize: A case study from Gujarat, India. Euphytica 130: 413-422.
